# Supplementary material for: Superplume mantle tracked isotopically the length of Africa from the Indian Ocean to the Red Sea
Source: Nat Commun. 2019 Dec 2;10:5493. doi: 10.1038/s41467-019-13181-7 (PMC6889401; doi:10.1038/s41467-019-13181-7)
Supplement: Supplementary file 2 — Supplementary Information [file 41467_2019_13181_MOESM2_ESM.pdf]

Supplementary Information for “Superplume mantle tracked isotopically the length of Africa from the Indian Ocean to the Red Sea”

John M. O’Connor, Wilfried Jokat, Marcel Regelous, Klaudia F. Kuiper, Daniel P. Miggins, Anthony A.P. Koppers

This PDF file includes:

Supplementary Table 1: Sample coordinates and information

Supplementary Figure 1: Photographic images of TV-grab samples

Supplementary Figure 2: Incremental heating $^{40}Ar/^{39}Ar$ age spectra

Supplementary Figure 3: Seaward extensions of the EARS.

Table 1. Summary of SO230 TV-grab locations and samples used in this study

Station	Date	Dredge	Time (UTC)	Longitude (°E)	Latitude (°S)	Depth
**DL2**	16/2/2014	On bottom	15:45	034° 45,23'	26° 8,12'	925 m
		Off bottom	17:21	034° 45,44'	26° 7,83'	959 m
**Sample**	**Size**	**Description**				
2	**26 x 22 x 22 cm**	**Mn- encrusted fine-grained basalt. Mn-crust is up to 5 cm thick, very fined grained and laminated; in places it appears to infill conical depressions up to 6 cm diameter and up to 10 cm deep**				
3	**22 x 17 x 13 cm**	**Mn-encrusted fine-grained basalt. Basalt peppered by very small holes, sometimes lined with a pea green to yellowish brown coating. Mn-crust is up to 4 cm thick, very fine-grained and laminated.**				

Station	Date	Dredge	Time (UTC)	Longitude (°E)	Latitude (°S)	Depth
**DL3**	16/2/2014	On bottom	18:28	034° 45,50'	26° 7,80'	943 m
		Off bottom	19:37	034° 45,45'	26° 7,84'	956.6
**Sample**	**Size**	**Description**				
**5**	**90 x 80 x 35 cm**	**Vesicular fine-grained basalt, partially encrusted by Mn, with coral attached**				

A

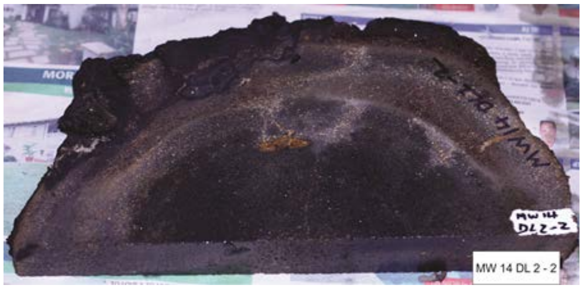

B

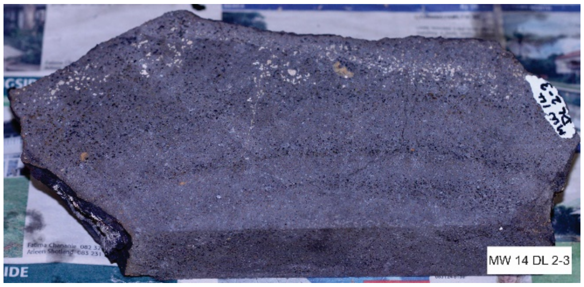

C

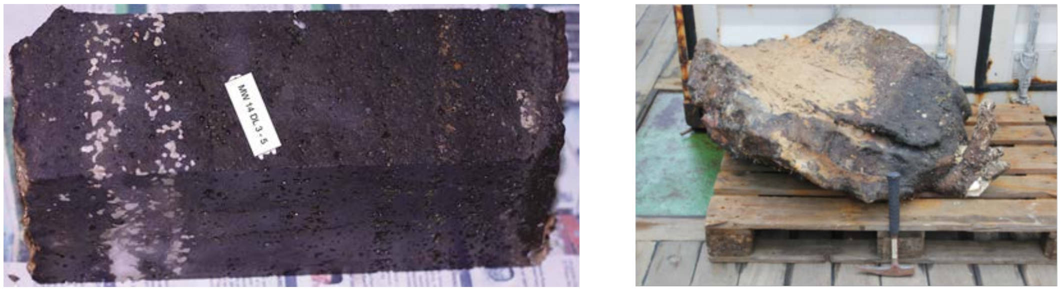

Supplementary Figure 1. Images of SO230 TV-grab samples. a) cobble sample MW 14 DL2-2. b) cobble sample MW 14 DL2-3. c) Boulder sample MW 14 DL3-5

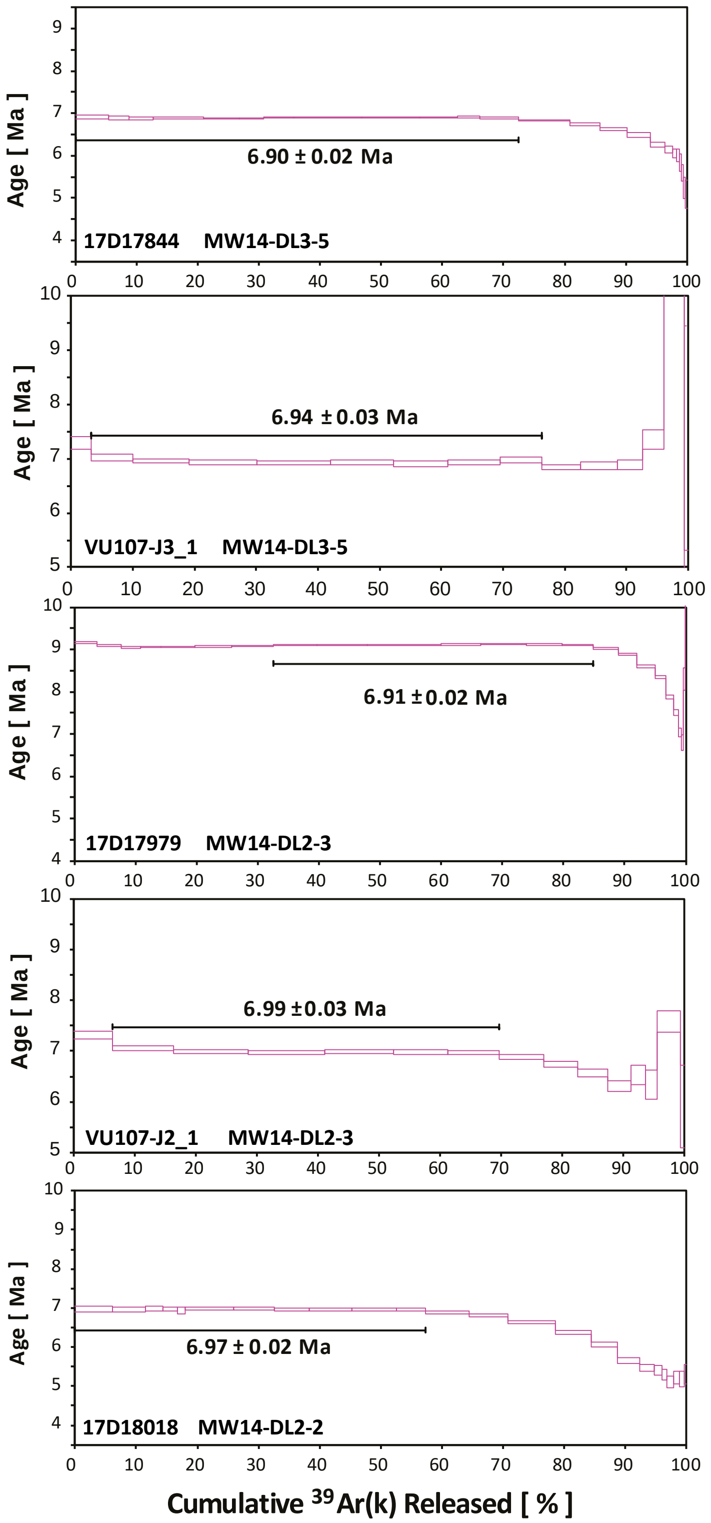

Supplementary Figure 2. Incremental heating $^{40}Ar/^{39}Ar$ age spectra.

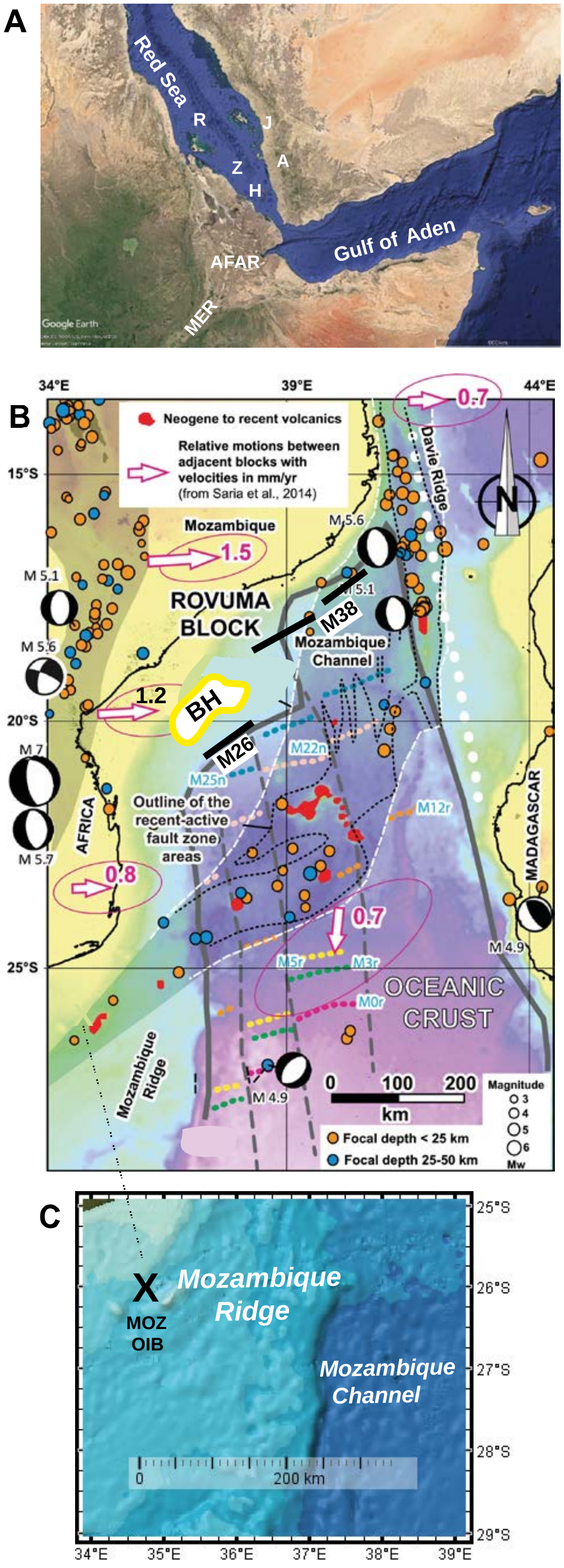

Supplementary Figure 3. Seaward extensions of the EARS. a) Google image showing the Red Sea-Gulf of Aden. Locations of samples discussed in the text: R = Ramad seamount; Z = Zubair island group; H = Hanish-Zukur island group; A = Hamdan volcanic field; J =Jizan volcanic field. See ref. 1 for further information. b) Structural sketch-map of the Mozambique channel area from ref. 2 showing the EARS extending offshore along the Comoros-Mayotte and Madagascar system and as far south as the Davie Ridge where it trends NNE-SSW across the Mozambique Channel and the Mozambique Ridge. The main corridors of recent to active faults are bounded by white dotted lines. Map location is indicated on Figure 1. OIB sample site on the Mozambique Ridge is indicate by X. Spreading anomalies (thick black lines) M38 (164.1 Ma).and M26 157.1 Ma are from ref. 3. Grey areas show location of recent/active faults corresponding to the western and eastern branch of the EARS. Red indicates schematically the associated seamounts, dykes and lava flows in the Mozambique Channel[4,5] and the volcanic domes on the Mozambique Ridge and in the northern Natal Valley. The Beira High (BH) represents a continental block, which was detached from Antarctica by 157 Ma at the latest[3]. Earthquake depths from the NEIC catalog (USGS). Earthquake focal mechanisms from the Global Centroid Moment Tensor database[6]. Pink arrows indicate relative motions between the African continent (Nubia Plate) and Madagascar (Somalia Plate)[7]. Elevation/bathymetry grid from GEBCO. Location of the oceanic crust and oceanic fracture zones compiled from refs[3,8,9]. Magnetic anomalies are from ref. 10. BH: Beira High. c) Enlarged map of the OIB sample site on the Mozambique Ridge. GEBCO (2014) gridded bathymetry prepared in GeoMapApp (www.geomapapp.org).

Supplementary References

1. Volker, F., Altherr, R., Jochum, K.-P. & McCulloch, M.T. Quaternary volcanic activity of the southern Red Sea: new data and assessment of models on magma sources and Afar plume-lithosphere interaction. *Tectonophysics* **278**, 15–29 (1997).

2. Deville, E. et al. Active fault system across the oceanic lithosphere of the Mozambique Channel: Implications for the Nubia–Somalia southern plate boundary. *Earth Planet. Sci. Lett.* **502**, 210–220 (2018).

3. Mueller, C.O., Jokat, W. Geophysical evidence for the crustal and distribution of magmatism along the central coast of Mozambique. Tectonophysics **712–713**, 684–703 (2017).

4. Courgeon, S. et al. Growth and demise of Cenozoic isolated carbonate platforms: new insights from the Mozambique Channel seamounts (SW Indian Ocean). *Mar. Geol.* **380**, 90–105 (2016).

5. Courgeon, S. et al. Impact of tectonic and volcanism on the Neogene evolution of isolated carbonate platforms (SW Indian Ocean). *Sediment. Geol.* **355**, 114–131m (2017).

6. Ekström, G., Nettles, M. & Dziewonski, A.M. The global CMT project 2004–2010: centroid-moment tensors for 13,017 earthquakes. *Phys. Earth Planet. Inter*. **200–201**, 1-9 (2012).

7. Saria, E., Calais, E., Altamimi, Z., Willis, P. & Farah, H. A new velocity field for Africa from combined GPS and DORIS space geodetic solutions: contribution to the definition of the African reference frame(AFREF). *J. Geophys. Res.* **118**, 1677–1697 (2013).

8. König, M. & Jokat, W. Advanced insights into magmatism and volcanism of the Mozambique Ridge and Mozambique Basin in the view of new potential field data. *Geophys. J. Int.* **180**, 158–180 (2010).

9. Leinweber, V.T. & Jokat, W. The Jurassic history of the Africa–Antarctica Corridor —new constraints from magnetic data on the conjugate continental margins. *Tectonophysics* **530**, 87–101 (2012).

10. Davis, J.K., Lawver, L.A., Norton, I.O. & Gahagan, L.G. New Somali Basin magnetic anomalies and plate model for the early Indian Ocean. *Gondwana. Res.* **34**, 16–28 (2016).
