# Supplementary material for: Superplume mantle tracked isotopically the length of Africa from the Indian Ocean to the Red Sea
Source: Nat Commun. 2019 Dec 2;10:5493. doi: 10.1038/s41467-019-13181-7 (PMC6889401; doi:10.1038/s41467-019-13181-7)
Supplement: Supplementary file 3 — Description of Additional Supplementary Files [file 41467_2019_13181_MOESM3_ESM.pdf]

Descriptions of Additional Supplementary Files

File Name: Supplementary Data 1
Description: Summary versions of ArArCalc age files

File Name: Supplementary Data 2
Description: Full versions of ArArCalc age files

File Name: Supplementary Data 3
Description: Geochemical data
