# Supplementary material for: Superplume mantle tracked isotopically the length of Africa from the Indian Ocean to the Red Sea
Source: Nat Commun. 2019 Dec 2;10:5493. doi: 10.1038/s41467-019-13181-7 (PMC6889401; doi:10.1038/s41467-019-13181-7)

EXP#17D17844 > MW14-DL3-5 > Groundmass > O-CONNOR (16-23)
INDIAN OCEAN > MOZAMBIQUE RIDGE
17-OSU-01 (1B35-17) > Incremental Heating > Dan Miggins

Information on Analysis and Constants Used in Calculations

Project = **O-CONNOR (16-23)**
Sample = **MW14-DL3-5**
Material = **Groundmass**
Location = **Mozambique Ridge**
Region = **Indian Ocean**
Analyst = **Dan Miggins**
Irradiation = **17-OSU-01 (1B35-17)**
Position = **X: 0 | Y: 0 | Z/H: 55.52779 mm**
FCT-NM Age = **28.201 ± 0.023 Ma**
FCT-NM Reference = **Kuiper et al (2008)**
FCT-NM 40Ar/39Ar Ratio = **10.16502 ± 0.01017**
FCT-NM J-value = **0.00154622 ± 0.00000155**
Air Shot 40Ar/36Ar = **302.5400 ± 0.2783**
Air Shot MDF = **0.99417507 ± 0.00062159 (LIN)**
Experiment Type = **Incremental Heating**
Extraction Method = **Bulk Laser Heating**
Heating = **77 sec**
Isolation = **3.00 min**
Instrument = **ARGUS-VI-D**
Preferred Age = **Plateau Age**
Age Classification = **Crystallization Age**
IGSN = Undefined
Rock Class = Undefined
Lithology = Undefined
Lat-Lon = Undefined - Undefined
Age Equations = **Min et al. (2000)**
Negative Intensities = **Allowed**
Collector Calibrations = **36Ar**
Decay 40K = **5.530 ± 0.048 E-10 1/a**
Decay 39Ar = **2.940 ± 0.016 E-07 1/h**
Decay 37Ar = **8.230 ± 0.012 E-04 1/h**
Decay 36Cl = **2.257 ± 0.015 E-06 1/a**
Decay 40K(EC,β^+) = **0.580 ± 0.009 E-10 1/a**
Decay 40K(β^-) = **4.950 ± 0.043 E-10 1/a**
Atmospheric 40/36(a) = **287.03 ± 3.67**
Atmospheric 38/36(a) = **0.1869**
Production 39/37(ca) = **0.0006756 ± 0.0000089**
Production 38/37(ca) = **0.0000718 ± 0.0000092**
Production 36/37(ca) = **0.0002663 ± 0.0000004**
Production 40/39(k) = **0.003823 ± 0.000102**
Production 38/39(k) = **0.012031 ± 0.000019**
Production 36/38(cl) = **262.80 ± 1.71**
Scaling Ratio K/Ca = **0.430**
Abundance Ratio 40K/K = **1.1700 ± 0.0100 E-04**
Atomic Weight K = **39.0983 ± 0.0001 g**

Subatmospheric Initial 40Ar/36Ar = 287.03 ± 1.28 (%SD).

Results

Results	40(a)/36(a) ± 2σ		40(r)/39(k) ± 2σ		Age ± 2σ (Ma)		MSWD	39Ar(k) (%,n)	K/Ca ± 2σ
**Age Plateau**			2.47193	± 0.00250	**6.90**	**± 0.02**	1.35	72.46	0.357 ± 0.036
				± 0.10%		**± 0.22%**	19%	12	
					Full External Error	± 0.16	1.85	2σ Confidence Limit	
					Analytical Error	± 0.01	1.1618	Error Magnification	
**Total Fusion Age**			2.43874	± 0.00238	**6.81**	**± 0.02**		24	0.311 ± 0.001
				± 0.10%		**± 0.22%**			
					Full External Error	± 0.15			
					Analytical Error	± 0.01			
**Normal Isochron**	**285.30**	**± 7.48**	2.47333	± 0.00634	**6.90**	**± 0.02**	3.13	72.46	
Error Chron		**± 2.62%**		± 0.26%		**± 0.32%**	0%	12	
					Full External Error	± 0.16	1.89	2σ Confidence Limit	
					Analytical Error	± 0.02	1.7691	Error Magnification	
**Inverse Isochron**	**287.03**	**± 7.33**	2.47186	± 0.00623	**6.90**	**± 0.02**	3.02	72.46	
Error Chron		**± 2.56%**		± 0.25%		**± 0.32%**	0%	12	
					Full External Error	± 0.16	1.89	2σ Confidence Limit	
					Analytical Error	± 0.02	1.7383	Error Magnification	
							13%	Spreading Factor	

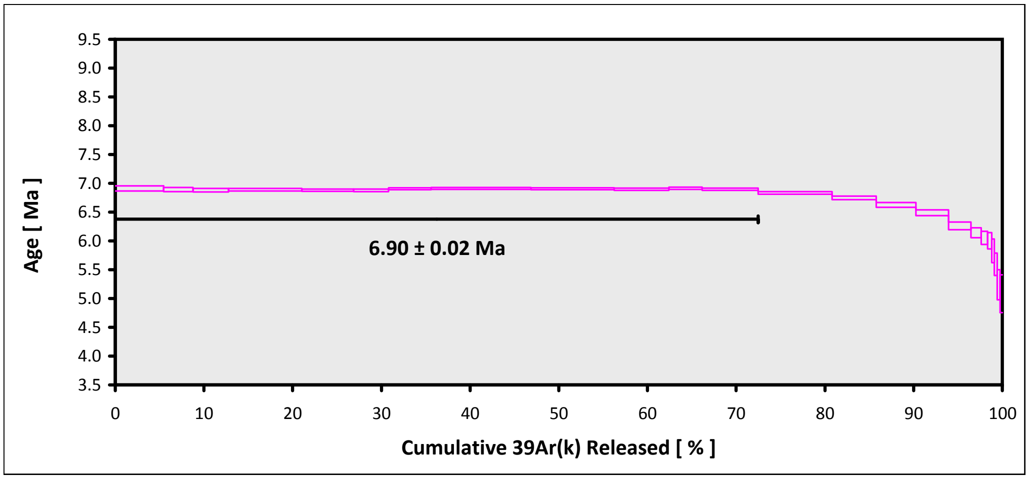

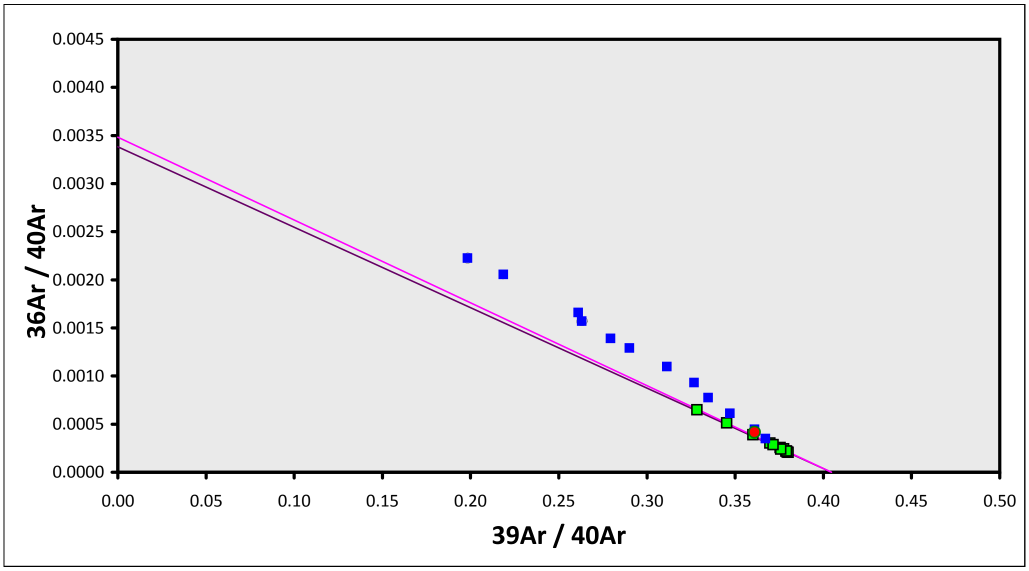

EXP#VU107-J3_1 > MW14-DL3-5 > Groundmass
INDIAN OCEAN > MOZAMBIQUE RIDGE
Incremental Heating > Klaudia Kuiper

Information on Analysis and Constants Used in Calculations

Sample = 055_VU107-J-3
Material = groundmass
Location = MW14 DL3-5
Analyst = Klaudia Kuiper
Project = VU107
Mass Discrimination Law = LIN
Irradiation = VU107
J = 0.00468970 ± 0.00000469
FCs = 28.201 ± 0.023 Ma
IGSN = Undefined
Preferred Age = Undefined
Classification = Undefined
Experiment Type = Undefined
Extraction Method = Undefined
Heating = 720 sec
Isolation = 18.00 min
Instrument = HELIX
Lithology = Undefined
Lat-Lon = Undefined - Undefined
Feature = Undefined
Age Equations = Min et al. (2000)
Negative Intensities = Allowed
Decay Constant 40K = 5.460 ± 0.053 E-10 1/a
Decay Constant 39Ar = 2.940 ± 0.016 E-07 1/h
Decay Constant 37Ar = 8.230 ± 0.012 E-04 1/h
Decay Constant 36Cl = 2.257 ± 0.015 E-06 1/a
Decay Activity 40K(EC,β⁺) = 3.310 ± 0.030 1/gs
Decay Activity 40K(β⁻) = 27.890 ± 0.150 1/gs
Atmospheric Ratio 40/36(a) = 298.56 ± 0.31
Atmospheric Ratio 38/36(a) = 0.1885 ± 0.0003
Production Ratio 39/37(ca) = 0.000673 ± 0.000004
Production Ratio 36/37(ca) = 0.000264 ± 0.000002
Production Ratio 40/39(k) = 0.000860 ± 0.000070
Production Ratio 38/39(k) = 0.012110 ± 0.000030
Production Ratio 36/38(cl) = 262.80 ± 1.71
Scaling Ratio K/Ca = 0.430
Abundance Ratio 40K/K = 1.1700 ± 0.0100 E-04
Atomic Weight K = 39.0983 ± 0.0001 g

Results	40(a)/36(a) ± 2σ		40(r)/39(k) ± 2σ		Age ± 2σ (Ma)		MSWD	39Ar(k) (%,n)	K/Ca ± 2σ
Age Plateau			0.81002	± 0.00255<br>± 0.32%	6.94	± 0.03<br>± 0.37%	1.87<br>7%	73.01<br>8	0.325 ± 0.054
					Full External Error	± 0.15	2.07	2σ Confidence Limit	
					Analytical Error	± 0.02	1.3685	Error Magnification	
Total Fusion Age			0.82451	± 0.00214<br>± 0.26%	7.07	± 0.02<br>± 0.33%		15	0.185 ± 0.001
					Full External Error	± 0.15			
					Analytical Error	± 0.02			
Normal Isochron<br>Overestimated Error	317.09	± 10.88<br>± 3.43%	0.80319	± 0.00440<br>± 0.55%	6.89	± 0.04<br>± 0.58%	0.29<br>94%	73.01<br>8	
					Full External Error	± 0.15	2.15	2σ Confidence Limit	
					Analytical Error	± 0.04	1.0000	Error Magnification	
Inverse Isochron<br>Overestimated Error	316.22	± 10.88<br>± 3.44%	0.80360	± 0.00439<br>± 0.55%	6.89	± 0.04<br>± 0.58%	0.28<br>95%	73.01<br>8	
					Full External Error	± 0.15	2.15	2σ Confidence Limit	
					Analytical Error	± 0.04	1.0000	Error Magnification	
							19%	Spreading Factor	

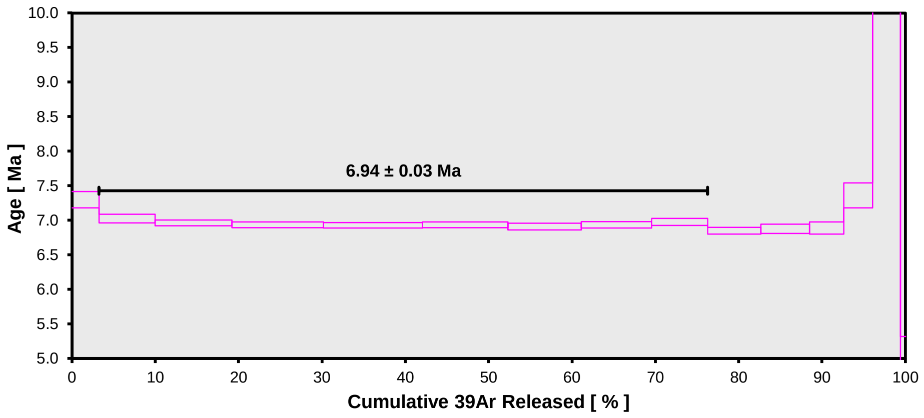

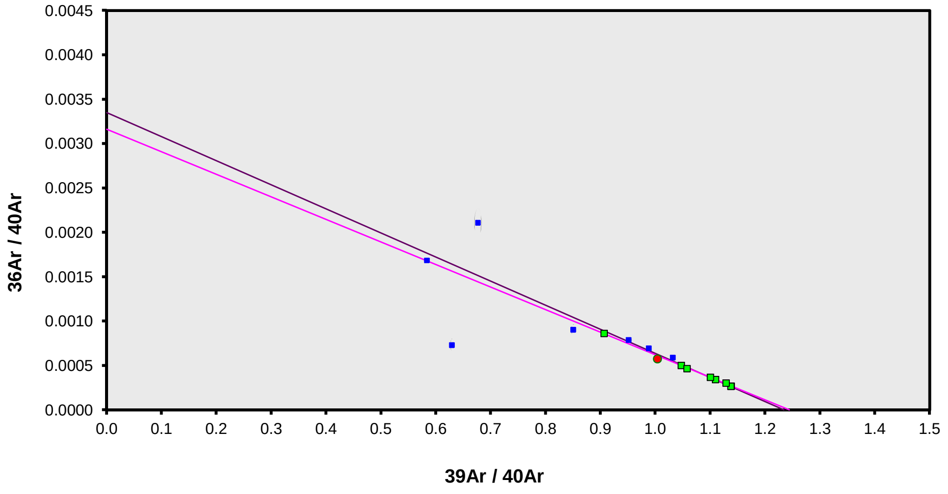

EXP#VU107-J2_1_corr > MW14-DL2-3 > Groundmass > INDIAN OCEAN > MOZAMBIQUE RIDGE > Incremental Heating > Klaudia Kuiper

Information on Analysis and Constants Used in Calculations

Sample = 031_VU107-J-2
Material = groundmass
Location = MW14 DL2-3
Analyst = Klaudia Kuiper
Project = VU107
Mass Discrimination Law = LIN
Irradiation = VU107
J = 0.00468970 ± 0.00000469
FCs = 28.201 ± 0.023 Ma
IGSN = Undefined
Preferred Age = Undefined
Classification = Undefined
Experiment Type = Undefined
Extraction Method = Undefined
Heating = 720 sec
Isolation = 18.00 min
Instrument = HELIX
Lithology = Undefined
Lat-Lon = Undefined - Undefined
Feature = Undefined
Age Equations = Min et al. (2000)
Negative Intensities = Allowed
Decay Constant 40K = 5.460 ± 0.053 E-10 1/a
Decay Constant 39Ar = 2.940 ± 0.016 E-07 1/h
Decay Constant 37Ar = 8.230 ± 0.012 E-04 1/h
Decay Constant 36Cl = 2.257 ± 0.015 E-06 1/a
Decay Activity 40K(EC,β^+) = 3.310 ± 0.030 1/gs
Decay Activity 40K(β^-) = 27.890 ± 0.150 1/gs
Atmospheric Ratio 40/36(a) = 298.56 ± 0.31
Atmospheric Ratio 38/36(a) = 0.1885 ± 0.0003
Production Ratio 39/37(ca) = 0.000673 ± 0.000004
Production Ratio 36/37(ca) = 0.000264 ± 0.000002
Production Ratio 40/39(k) = 0.000860 ± 0.000070
Production Ratio 38/39(k) = 0.012110 ± 0.000030
Production Ratio 36/38(cl) = 262.80 ± 1.71
Scaling Ratio K/Ca = 0.430
Abundance Ratio 40K/K = 1.1700 ± 0.0100 E-04
Atomic Weight K = 39.0983 ± 0.0001 g

Results	40(a)/36(a) ± 2s		40(r)/39(k) ± 2s		Age ± 2s (Ma)		MSWD	39Ar(k) (%,n)	K/Ca ± 2s
Age Plateau			0.81588	± 0.00301<br>± 0.37%	6.99	± 0.03<br>± 0.42%	2.07<br>7%	63.23<br>6	0.326 ± 0.054
					Full External Error	± 0.15	2.26	2σ Confidence Limit	
					Analytical Error	± 0.03	1.4404	Error Magnification	
Total Fusion Age			0.80928	± 0.00218<br>± 0.27%	6.94	± 0.02<br>± 0.33%		15	0.204 ± 0.001
					Full External Error	± 0.15			
					Analytical Error	± 0.02			
Normal Isochron	320.57	± 15.82<br>± 4.93%	0.80761	± 0.00633<br>± 0.78%	6.92	± 0.06<br>± 0.81%	0.35<br>84%	63.23<br>6	
					Full External Error	± 0.16	2.41	2σ Confidence Limit	
					Analytical Error	± 0.05	1.0000	Error Magnification	
Inverse Isochron	321.16	± 15.79<br>± 4.92%	0.80740	± 0.00632<br>± 0.78%	6.92	± 0.06<br>± 0.81%	0.35<br>84%	63.23<br>6	
					Full External Error	± 0.16	2.41	2σ Confidence Limit	
					Analytical Error	± 0.05	1.0000	Error Magnification	
							12%	Spreading Factor	

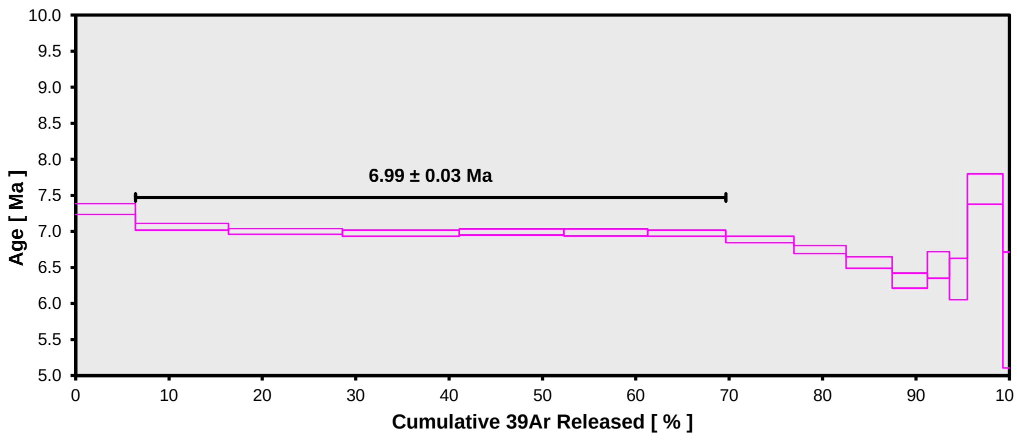

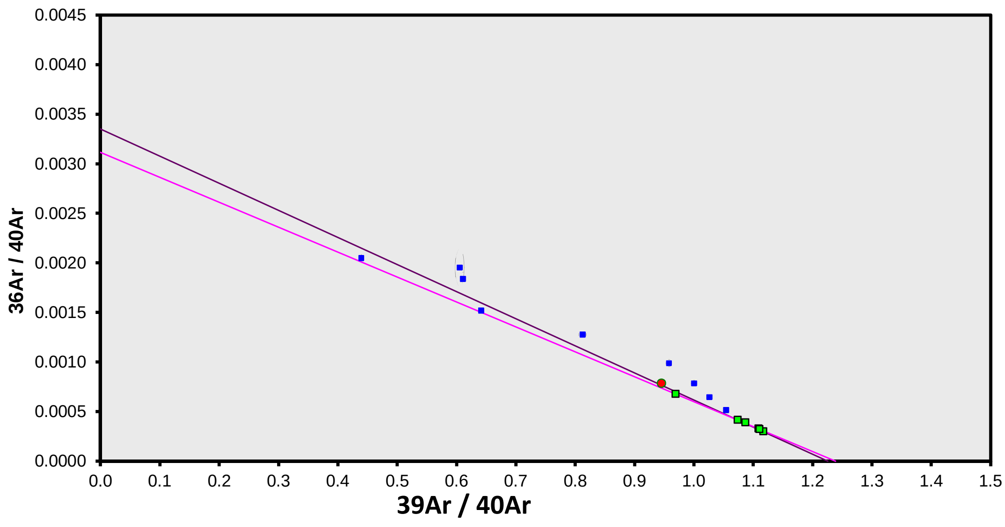

EXP#17D17979 > MW14-DL2-3 > Groundmass > O-CONNOR (16-23)
INDIAN OCEAN > MOZAMBIQUE RIDGE
17-OSU-01 (1B32-17) > Incremental Heating > Dan Miggins

Information on Analysis and Constants Used in Calculations

Project = **O-CONNOR (16-23)**
Sample = **MW14-DL2-3**
Material = **Groundmass**
Location = **Mozambique Ridge**
Region = **Indian Ocean**
Analyst = **Dan Miggins**
Irradiation = **17-OSU-01 (1B32-17)**
Position = **X: 0 | Y: 0 | Z/H: 50.93376 mm**
FCT-NM Age = **28.201 ± 0.023 Ma**
FCT-NM Reference = **Kuiper et al (2008)**
FCT-NM 40Ar/39Ar Ratio = **10.04942 ± 0.01015**
FCT-NM J-value = **0.00156401 ± 0.00000158**
Air Shot 40Ar/36Ar = **302.5270 ± 0.2844**
Air Shot MDF = **0.99418558 ± 0.00062340 (LIN)**
Experiment Type = **Incremental Heating**
Extraction Method = **Bulk Laser Heating**
Heating = **77 sec**
Isolation = **3.00 min**
Instrument = **ARGUS-VI-D**
Preferred Age = **Plateau Age**
Age Classification = **Crystallization Age**
IGSN = Undefined
Rock Class = Undefined
Lithology = Undefined
Lat-Lon = Undefined - Undefined
Age Equations = **Min et al. (2000)**
Negative Intensities = **Allowed**
Collector Calibrations = **36Ar**
Decay 40K = **5.530 ± 0.048 E-10 1/a**
Decay 39Ar = **2.940 ± 0.016 E-07 1/h**
Decay 37Ar = **8.230 ± 0.012 E-04 1/h**
Decay 36Cl = **2.257 ± 0.015 E-06 1/a**
Decay 40K(EC,β^+) = **0.580 ± 0.009 E-10 1/a**
Decay 40K(β^-) = **4.950 ± 0.043 E-10 1/a**
Atmospheric 40/36(a) = **295.50**
Atmospheric 38/36(a) = **0.1869**
Production 39/37(ca) = **0.0006756 ± 0.0000089**
Production 38/37(ca) = **0.0000718 ± 0.0000092**
Production 36/37(ca) = **0.0002663 ± 0.0000004**
Production 40/39(k) = **0.003823 ± 0.000102**
Production 38/39(k) = **0.012031 ± 0.000019**
Production 36/38(cl) = **262.80 ± 1.71**
Scaling Ratio K/Ca = **0.430**
Abundance Ratio 40K/K = **1.1700 ± 0.0100 E-04**
Atomic Weight K = **39.0983 ± 0.0001 g**

Results

Results	40(a)/36(a) ± 2σ		40(r)/39(k) ± 2σ		Age ± 2σ (Ma)		MSWD	39Ar(k) (%,n)	K/Ca ± 2σ
**Age Plateau**			2.44919	± 0.00345	**6.91**	**± 0.02**	3.26	52.27	0.267 ± 0.041
Error Mean				± 0.14%		**± 0.25%**	0%	7	
				Full External Error		± 0.16	2.15	2σ Confidence Limit	
				Analytical Error		± 0.01	1.8068	Error Magnification	
**Total Fusion Age**			2.42143	± 0.00147	**6.84**	**± 0.01**		24	0.284 ± 0.001
				± 0.06%		**± 0.21%**			
				Full External Error		± 0.15			
				Analytical Error		± 0.00			
**Normal Isochron**	**316.99**	**± 31.52**	2.43851	± 0.01575	**6.88**	**± 0.05**	2.90	52.27	
Error Chron		**± 9.94%**		± 0.65%		**± 0.68%**	1%	7	
				Full External Error		± 0.16	2.26	2σ Confidence Limit	
				Analytical Error		± 0.04	1.7038	Error Magnification	
**Inverse Isochron**	**315.46**	**± 31.97**	2.43944	± 0.01594	**6.89**	**± 0.05**	2.95	52.27	
Error Chron		**± 10.13%**		± 0.65%		**± 0.68%**	1%	7	
				Full External Error		± 0.16	2.26	2σ Confidence Limit	
				Analytical Error		± 0.04	1.7178	Error Magnification	
							4%	Spreading Factor	

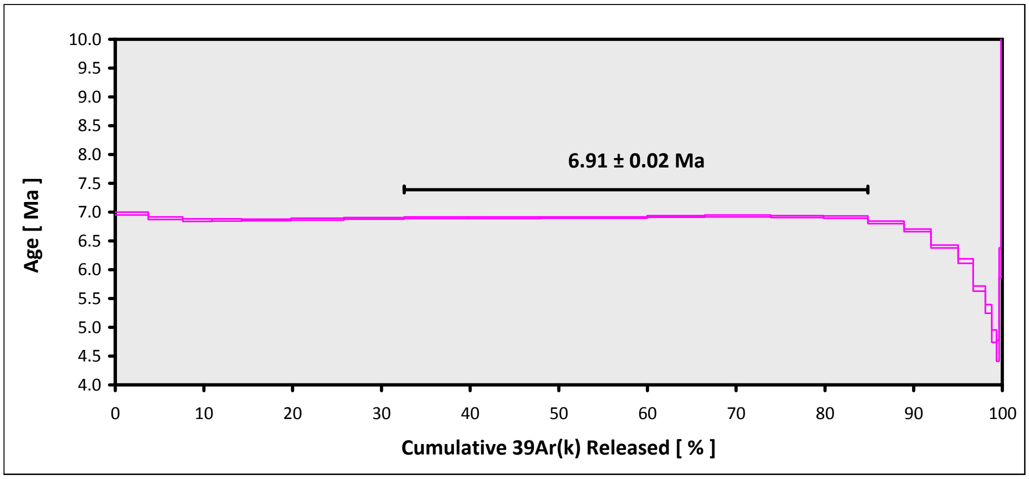

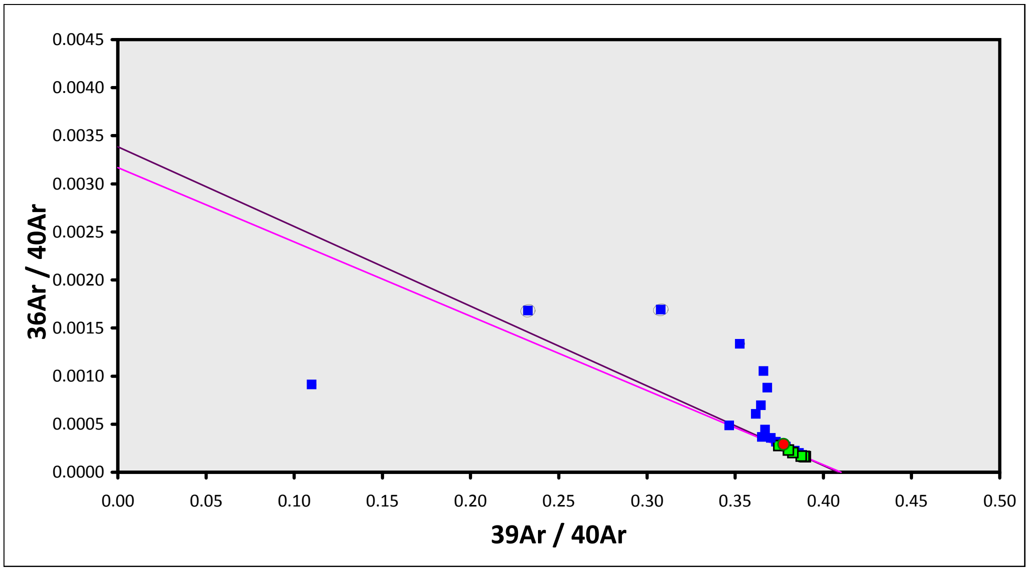

EXP#17D18018 > MW14-DL2-2 > Groundmass > O-CONNOR (16-23)
INDIAN OCEAN > MOZAMBIQUE RIDGE
17-OSU-01 (1B38-17) > Incremental Heating > Dan Miggins

Information on Analysis and Constants Used in Calculations

Project = **O-CONNOR (16-23)**
Sample = **MW14-DL2-2**
Material = **Groundmass**
Location = **Mozambique Ridge**
Region = **Indian Ocean**
Analyst = **Dan Miggins**
Irradiation = **17-OSU-01 (1B38-17)**
Position = **X: 0 | Y: 0 | Z/H: 59.89021 mm**
FCT-NM Age = **28.201 ± 0.023 Ma**
FCT-NM Reference = **Kuiper et al (2008)**
FCT-NM 40Ar/39Ar Ratio = **10.28448 ± 0.01018**
FCT-NM J-value = **0.00152826 ± 0.00000151**
Air Shot 40Ar/36Ar = **302.7200 ± 0.4268**
Air Shot MDF = **0.99402969 ± 0.00067386 (LIN)**
Experiment Type = **Incremental Heating**
Extraction Method = **Bulk Laser Heating**
Heating = **77 sec**
Isolation = **3.00 min**
Instrument = **ARGUS-VI-D**
Preferred Age = **Plateau Age**
Age Classification = **Crystallization Age**
IGSN = Undefined
Rock Class = Undefined
Lithology = Undefined
Lat-Lon = Undefined - Undefined
Age Equations = **Min et al. (2000)**
Negative Intensities = **Allowed**
Collector Calibrations = **36Ar**
Decay 40K = **5.530 ± 0.048 E-10 1/a**
Decay 39Ar = **2.940 ± 0.016 E-07 1/h**
Decay 37Ar = **8.230 ± 0.012 E-04 1/h**
Decay 36Cl = **2.257 ± 0.015 E-06 1/a**
Decay 40K(EC,β^+) = **0.580 ± 0.009 E-10 1/a**
Decay 40K(β^-) = **4.950 ± 0.043 E-10 1/a**
Atmospheric 40/36(a) = **354.12 ± 3.99**
Atmospheric 38/36(a) = **0.1869**
Production 39/37(ca) = **0.0006756 ± 0.0000089**
Production 38/37(ca) = **0.0000718 ± 0.0000092**
Production 36/37(ca) = **0.0002663 ± 0.0000004**
Production 40/39(k) = **0.003823 ± 0.000102**
Production 38/39(k) = **0.012031 ± 0.000019**
Production 36/38(cl) = **262.80 ± 1.71**
Scaling Ratio K/Ca = **0.430**
Abundance Ratio 40K/K = **1.1700 ± 0.0100 E-04**
Atomic Weight K = **39.0983 ± 0.0001 g**

Excess Initial 40Ar/36Ar = 354.12 ± 1.13 (%SD).

Results

Results	40(a)/36(a) ± 2σ		40(r)/39(k) ± 2σ		Age ± 2σ (Ma)		MSWD	39Ar(k) (%,n)	K/Ca ± 2σ
**Age Plateau**			2.52589	± 0.00465<br>± 0.18%	**6.97**	**± 0.02<br>± 0.27%**	0.40<br>95%	57.32<br>11	0.173 ± 0.025
				Full External Error		± 0.16	1.89	2σ Confidence Limit	
				Analytical Error		± 0.01	1.0000	Error Magnification	
**Total Fusion Age**			2.42154	± 0.00424<br>± 0.18%	**6.68**	**± 0.02<br>± 0.26%**		24	0.120 ± 0.000
				Full External Error		± 0.15			
				Analytical Error		± 0.01			
**Normal Isochron**	**354.70**	**± 7.97<br>± 2.25%**	2.52532	± 0.00989<br>± 0.39%	**6.97**	**± 0.03<br>± 0.44%**	0.71<br>70%	57.32<br>11	
				Full External Error		± 0.16	1.94	2σ Confidence Limit	
				Analytical Error		± 0.03	1.0000	Error Magnification	
**Inverse Isochron**	**354.12**	**± 7.97<br>± 2.25%**	2.52619	± 0.00990<br>± 0.39%	**6.97**	**± 0.03<br>± 0.44%**	0.71<br>70%	57.32<br>11	
				Full External Error		± 0.16	1.94	2σ Confidence Limit	
				Analytical Error		± 0.03	1.0000	Error Magnification	
							18%	Spreading Factor	

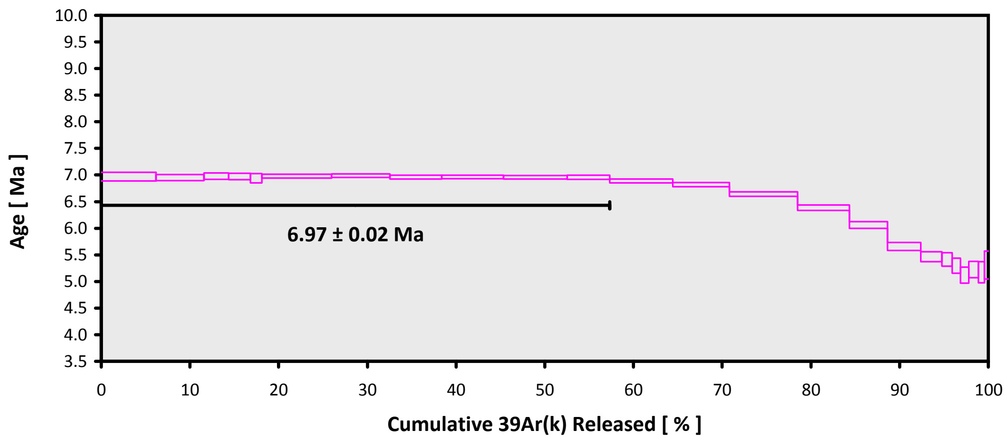

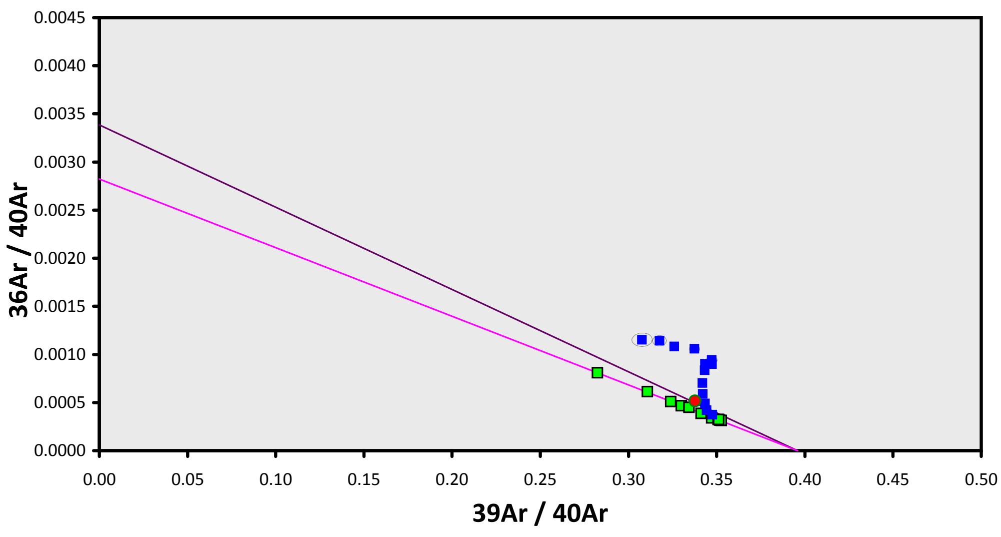

EXP#VU107-J1_1_corr_ > MW14-DL2-2 > Groundmass > INDIAN OCEAN > MOZAMBIQUE RIDGE > Incremental Heating > Klaudia Kuiper

Information on Analysis and Constants Used in Calculations

Sample = 011_VU107-J-1
Material = groundmass
Location = MW14 DL2-2
Analyst = Klaudia Kuiper
Project = VU107
Mass Discrimination Law = LIN
Irradiation = VU107
J = 0.00468970 ± 0.00000469
FCs = 28.201 ± 0.023 Ma
IGSN = Undefined
Preferred Age = Undefined
Classification = Undefined
Experiment Type = Undefined
Extraction Method = Undefined
Heating = 720 sec
Isolation = 18.00 min
Instrument = HELIX
Lithology = Undefined
Lat-Lon = Undefined - Undefined
Feature = Undefined
Age Equations = Min et al. (2000)
Negative Intensities = Allowed
Decay Constant 40K = 5.460 ± 0.053 E-10 1/a
Decay Constant 39Ar = 2.940 ± 0.016 E-07 1/h
Decay Constant 37Ar = 8.230 ± 0.012 E-04 1/h
Decay Constant 36Cl = 2.257 ± 0.015 E-06 1/a
Decay Activity 40K(EC,β^+) = 3.310 ± 0.030 1/gs
Decay Activity 40K(β^-) = 27.890 ± 0.150 1/gs
Atmospheric Ratio 40/36(a) = 298.56 ± 0.31
Atmospheric Ratio 38/36(a) = 0.1885 ± 0.0003
Production Ratio 39/37(ca) = 0.000673 ± 0.000004
Production Ratio 36/37(ca) = 0.000264 ± 0.000002
Production Ratio 40/39(k) = 0.000860 ± 0.000070
Production Ratio 38/39(k) = 0.012110 ± 0.000030
Production Ratio 36/38(cl) = 262.80 ± 1.71
Scaling Ratio K/Ca = 0.430
Abundance Ratio 40K/K = 1.1700 ± 0.0100 E-04
Atomic Weight K = 39.0983 ± 0.0001 g

Results	40(a)/36(a) ± 2s		40(r)/39(k) ± 2s		Age ± 2s (Ma)		MSWD	39Ar(k) (%,n)	K/Ca ± 2s
Age Plateau Error Mean			0.84748	± 0.01424 ± 1.68%	7.26	± 0.12 ± 1.69%	7.94 0%	43.44 4	0.168 ± 0.021
					Full External Error	± 0.20	2.63	2σ Confidence Limit	
					Analytical Error	± 0.12	2.8176	Error Magnification	
Total Fusion Age			0.82813	± 0.00546 ± 0.66%	7.10	± 0.05 ± 0.69%		15	0.100 ± 0.000
					Full External Error	± 0.16			
					Analytical Error	± 0.05			
Normal Isochron	334.01	± 74.30 ± 22.24%	0.79763	± 0.10427 ± 13.07%	6.84	± 0.89 ± 13.05%	7.99 0%	43.44 4	
					Full External Error	± 0.90	3.00	2σ Confidence Limit	
					Analytical Error	± 0.89	2.8267	Error Magnification	
Inverse Isochron Error Chron	332.07	± 74.98 ± 22.58%	0.80114	± 0.10238 ± 12.78%	6.87	± 0.88 ± 12.76%	8.13 0%	43.44 4	
					Full External Error	± 0.89	3.00	2σ Confidence Limit	
					Analytical Error	± 0.88	2.8506	Error Magnification	
								8%	Spreading Factor

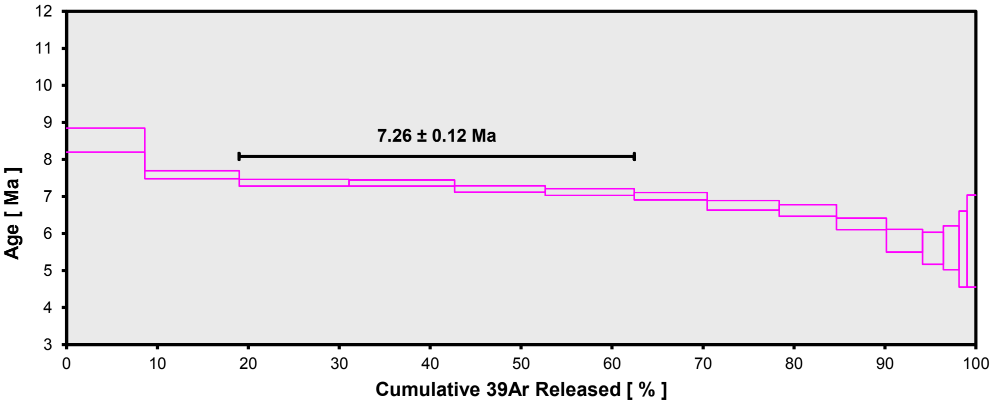

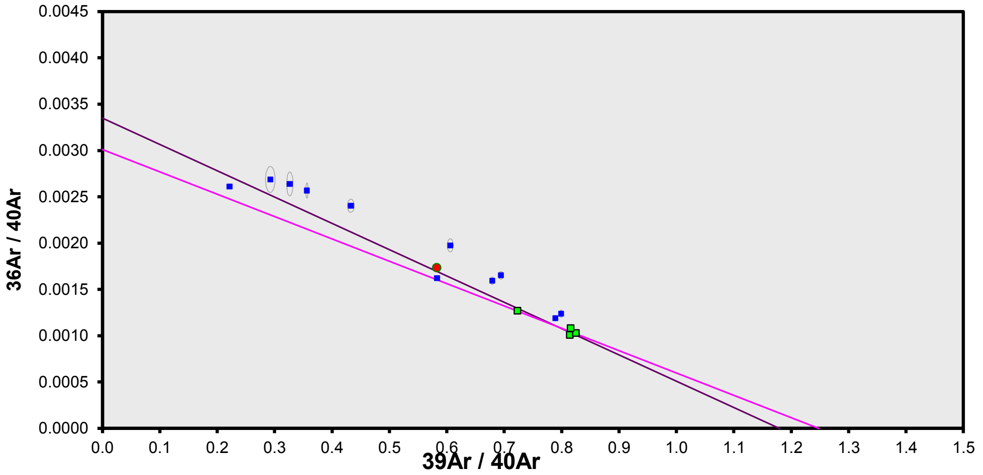

Supplement: Supplementary file 4 — Supplementary Data 1 [file 41467_2019_13181_MOESM4_ESM.pdf]
