# Supplementary material for: Superplume mantle tracked isotopically the length of Africa from the Indian Ocean to the Red Sea
Source: Nat Commun. 2019 Dec 2;10:5493. doi: 10.1038/s41467-019-13181-7 (PMC6889401; doi:10.1038/s41467-019-13181-7)
Supplement: Supplementary file 5 — Supplementary Data 2 [file 41467_2019_13181_MOESM5_ESM.pdf]

Relative Abundances			36Ar [fA]	%1σ	37Ar [fA]	%1σ	38Ar [fA]	%1σ	39Ar [fA]	%1σ	40Ar [fA]	%1σ	40(r)/39(k) ± 2σ	Age ± 2σ (Ma)	40Ar(r) (%)	39Ar(k) (%)	K/Ca ± 2σ
17D17848	1.8 %	✓	0.1998093	0.438	71.1661	0.582	1.125745	2.077	91.5036	0.068	278.8088	0.017	2.47713 ± 0.01593	6.91 ± 0.04	81.26	5.45	0.553 ± 0.006
17D17850	1.9 %	✓	0.0951668	0.579	47.0418	0.782	0.694021	3.365	55.6852	0.076	161.3911	0.028	2.46990 ± 0.01299	6.89 ± 0.04	85.17	3.31	0.509 ± 0.008
17D17851	2.0 %	✓	0.0894355	0.673	61.1380	0.654	0.795528	2.921	67.5473	0.073	187.6868	0.023	2.46543 ± 0.01023	6.88 ± 0.03	88.67	4.02	0.475 ± 0.006
17D17853	2.2 %	✓	0.1517442	0.502	141.0503	0.454	1.659861	1.432	138.8378	0.065	375.8654	0.013	2.46904 ± 0.00761	6.89 ± 0.02	91.14	8.26	0.423 ± 0.004
17D17854	2.4 %	✓	0.0979875	0.584	116.4327	0.480	1.175479	1.945	97.8482	0.068	260.6872	0.018	2.46588 ± 0.00705	6.88 ± 0.02	92.48	5.82	0.361 ± 0.004
17D17856	2.7 %	✓	0.0652398	0.770	86.1335	0.534	0.804280	2.843	66.1748	0.073	175.3637	0.026	2.46487 ± 0.00757	6.88 ± 0.02	92.93	3.94	0.330 ± 0.004
17D17857	3.0 %	✓	0.0764243	0.636	111.3732	0.490	0.969016	2.471	81.1207	0.070	214.3031	0.021	2.47479 ± 0.00666	6.91 ± 0.02	93.59	4.83	0.313 ± 0.003
17D17859	3.4 %	✓	0.1747086	0.474	263.5261	0.418	2.279616	1.057	188.6455	0.065	497.3068	0.010	2.47567 ± 0.00586	6.91 ± 0.02	93.82	11.22	0.308 ± 0.003
17D17860	3.9 %	✓	0.1425484	0.503	213.8780	0.427	1.892348	1.226	157.8137	0.065	415.3594	0.012	2.47473 ± 0.00587	6.91 ± 0.02	93.94	9.39	0.317 ± 0.003
17D17862	4.5 %	✓	0.0938120	0.605	127.7742	0.472	1.259148	1.754	103.7091	0.068	273.6596	0.018	2.47150 ± 0.00640	6.90 ± 0.02	93.58	6.17	0.349 ± 0.003
17D17863	5.2 %	✓	0.0589102	0.743	70.5059	0.591	0.731534	3.081	63.0967	0.073	167.9734	0.027	2.47764 ± 0.00734	6.91 ± 0.02	93.00	3.75	0.385 ± 0.005
17D17865	6.0 %	✓	0.1088545	0.575	103.8292	0.502	1.253569	1.878	106.0255	0.067	285.6191	0.016	2.47185 ± 0.00745	6.90 ± 0.02	91.70	6.31	0.439 ± 0.004
17D17866	6.9 %		0.1652993	0.482	118.5025	0.471	1.694897	1.401	140.1678	0.066	381.9581	0.012	2.44871 ± 0.00843	6.83 ± 0.02	89.81	8.34	0.508 ± 0.005
17D17868	7.9 %		0.1206357	0.604	65.1574	0.630	1.033972	2.182	83.9747	0.070	232.8157	0.020	2.41687 ± 0.01094	6.75 ± 0.03	87.13	5.00	0.554 ± 0.007
17D17869	9.0 %		0.1496825	0.530	65.8917	0.611	0.927906	2.551	74.8597	0.071	215.8122	0.022	2.37384 ± 0.01479	6.63 ± 0.04	82.29	4.45	0.488 ± 0.006
17D17871	10.3 %		0.1644891	0.456	78.7940	0.562	0.835683	2.736	62.1406	0.075	185.7654	0.025	2.32480 ± 0.01874	6.49 ± 0.05	77.70	3.70	0.339 ± 0.004
17D17872	11.6 %		0.1425918	0.509	85.6230	0.534	0.583937	3.955	42.1028	0.085	128.8280	0.035	2.24252 ± 0.02361	6.26 ± 0.07	73.19	2.50	0.211 ± 0.002
17D17873	12.5 %		0.0837744	0.647	54.1549	0.690	0.239910	9.589	19.6947	0.132	63.2350	0.066	2.20028 ± 0.03133	6.14 ± 0.09	68.40	1.17	0.156 ± 0.002
17D17875	13.4 %		0.0639726	0.679	41.5902	0.841	0.169706	13.563	11.9140	0.199	41.0059	0.101	2.16882 ± 0.04063	6.05 ± 0.11	62.86	0.71	0.123 ± 0.002
17D17876	14.6 %		0.0518486	0.887	40.9529	0.814	0.142117	16.334	8.2469	0.282	29.4564	0.137	2.15044 ± 0.05149	6.00 ± 0.14	60.00	0.49	0.086 ± 0.001
17D17877	16.0 %		0.0358584	1.078	35.5526	0.956	0.045343	51.854	4.4484	0.536	16.8306	0.246	2.08813 ± 0.07368	5.83 ± 0.21	54.89	0.26	0.054 ± 0.001
17D17879	17.6 %		0.0589807	0.791	83.1926	0.557	0.113113	20.653	5.8414	0.397	22.1861	0.184	2.00434 ± 0.07029	5.60 ± 0.20	52.26	0.34	0.030 ± 0.000
17D17880	19.3 %		0.0786909	0.664	109.5442	0.497	0.111236	22.769	5.3433	0.423	24.1226	0.172	1.87707 ± 0.09364	5.24 ± 0.26	41.00	0.31	0.021 ± 0.000
17D17882	21.0 %		0.0823556	0.686	129.6297	0.466	0.091403	26.300	4.3509	0.519	21.5074	0.188	1.82071 ± 0.11755	5.08 ± 0.33	36.09	0.25	0.014 ± 0.000
		Σ	2.5528208	0.121	2322.4347	0.114	20.629368	0.555	1681.0932	0.017	4657.5480	0.005					

Information on Analysis and Constants Used in Calculations

Project = **O-CONNOR (16-23)**
Sample = **MW14-DL3-5**
Material = **Groundmass**
Location = **Mozambique Ridge**
Region = **Indian Ocean**
Analyst = **Dan Miggins**
Irradiation = **17-OSU-01 (1B35-17)**
Position = **X: 0 | Y: 0 | Z/H: 55.52779 mm**
FCT-NM Age = **28.201 ± 0.023 Ma**
FCT-NM Reference = **Kuiper et al (2008)**
FCT-NM 40Ar/39Ar Ratio = **10.16502 ± 0.01017**
FCT-NM J-value = **0.00154622 ± 0.00000155**
Air Shot 40Ar/36Ar = **302.5400 ± 0.2783**
Air Shot MDF = **0.99417507 ± 0.00062159 (LIN)**
Experiment Type = **Incremental Heating**
Extraction Method = **Bulk Laser Heating**
Heating = **77 sec**
Isolation = **3.00 min**
Instrument = **ARGUS-VI-D**
Preferred Age = **Plateau Age**
Age Classification = **Crystallization Age**
IGSN = Undefined
Rock Class = Undefined
Lithology = Undefined
Lat-Lon = Undefined - Undefined

Age Equations = **Min et al. (2000)**
Negative Intensities = **Allowed**
Collector Calibrations = **36Ar**
Decay 40K = **5.530 ± 0.048 E-10 1/a**
Decay 39Ar = **2.940 ± 0.016 E-07 1/h**
Decay 37Ar = **8.230 ± 0.012 E-04 1/h**
Decay 36Cl = **2.257 ± 0.015 E-06 1/a**
Decay 40K(EC,β^+) = **0.580 ± 0.009 E-10 1/a**
Decay 40K(β^-) = **4.950 ± 0.043 E-10 1/a**
Atmospheric 40/36(a) = **287.03 ± 3.67**
Atmospheric 38/36(a) = **0.1869**
Production 39/37(ca) = **0.0006756 ± 0.0000089**
Production 38/37(ca) = **0.0000718 ± 0.0000092**
Production 36/37(ca) = **0.0002663 ± 0.0000004**
Production 40/39(k) = **0.003823 ± 0.000102**
Production 38/39(k) = **0.012031 ± 0.000019**
Production 36/38(cl) = **262.80 ± 1.71**
Scaling Ratio K/Ca = **0.430**
Abundance Ratio 40K/K = **1.1700 ± 0.0100 E-04**
Atomic Weight K = **39.0983 ± 0.0001 g**

Results	40(a)/36(a) ± 2σ	40(r)/39(k) ± 2σ	Age ± 2σ (Ma)	MSWD	39Ar(k) (%,n)	K/Ca ± 2σ
**Age Plateau**		2.47193 ± 0.00250 ± 0.10%	**6.90 ± 0.02 ± 0.22%**	1.35	72.46	0.357 ± 0.036
				19%	12	
			Full External Error ± 0.16	1.85	2σ Confidence Limit	
			Analytical Error ± 0.01	1.1618	Error Magnification	
**Total Fusion Age**		2.43874 ± 0.00238 ± 0.10%	**6.81 ± 0.02 ± 0.22%**		24	0.311 ± 0.001
			Full External Error ± 0.15			
			Analytical Error ± 0.01			
**Normal Isochron** Error Chron	**285.30 ± 7.48 ± 2.62%**	2.47333 ± 0.00634 ± 0.26%	**6.90 ± 0.02 ± 0.32%**	3.13	72.46	
				0%	12	
			Full External Error ± 0.16	1.89	2σ Confidence Limit	
			Analytical Error ± 0.02	1.7691	Error Magnification	
				54	Number of Iterations	
				0.0000247184	Convergence	
**Inverse Isochron** Error Chron	**287.03 ± 7.33 ± 2.56%**	2.47186 ± 0.00623 ± 0.25%	**6.90 ± 0.02 ± 0.32%**	3.02	72.46	
				0%	12	
			Full External Error ± 0.16	1.89	2σ Confidence Limit	
			Analytical Error ± 0.02	1.7383	Error Magnification	
				3	Number of Iterations	
				0.0000124079	Convergence	
				13%	Spreading Factor	

Notes

Subatmospheric Initial 40Ar/36Ar = 287.03 ± 1.28 (%SD).

Incremental Heating			36Ar(a) [fA]	37Ar(ca) [fA]	38Ar(cl) [fA]	39Ar(k) [fA]	40Ar(r) [fA]	Age ± 2σ (Ma)	40Ar(r) (%)	39Ar(k) (%)	K/Ca ± 2σ
17D17848	1.8 %	✓	0.1808578	71.1661	0.0000000	91.4555	226.5476	6.91 ± 0.04	81.26	5.45	0.553 ± 0.006
17D17850	1.9 %	✓	0.0826384	47.0418	0.0056316	55.6535	137.4586	6.89 ± 0.04	85.17	3.31	0.509 ± 0.008
17D17851	2.0 %	✓	0.0731545	61.1380	0.0000000	67.5060	166.4312	6.88 ± 0.03	88.67	4.02	0.475 ± 0.006
17D17853	2.2 %	✓	0.1141825	141.0503	0.0000000	138.7425	342.5612	6.89 ± 0.02	91.14	8.26	0.423 ± 0.004
17D17854	2.4 %	✓	0.0669815	116.4327	0.0000000	97.7695	241.0877	6.88 ± 0.02	92.48	5.82	0.361 ± 0.004
17D17856	2.7 %	✓	0.0423025	86.1335	0.0000000	66.1167	162.9688	6.88 ± 0.02	92.93	3.94	0.330 ± 0.004
17D17857	3.0 %	✓	0.0467656	111.3732	0.0000000	81.0454	200.5701	6.91 ± 0.02	93.59	4.83	0.313 ± 0.003
17D17859	3.4 %	✓	0.1045316	263.5261	0.0000000	188.4674	466.5825	6.91 ± 0.02	93.82	11.22	0.308 ± 0.003
17D17860	3.9 %	✓	0.0855927	213.8780	0.0000000	157.6692	390.1890	6.91 ± 0.02	93.94	9.39	0.317 ± 0.003
17D17862	4.5 %	✓	0.0597857	127.7742	0.0000000	103.6227	256.1032	6.90 ± 0.02	93.58	6.17	0.349 ± 0.003
17D17863	5.2 %	✓	0.0401345	70.5059	0.0000000	63.0490	156.2126	6.91 ± 0.02	93.00	3.75	0.385 ± 0.005
17D17865	6.0 %	✓	0.0812047	103.8292	0.0000000	105.9554	261.9058	6.90 ± 0.02	91.70	6.31	0.439 ± 0.004
17D17866	6.9 %		0.1337421	118.5025	0.0000000	140.0877	343.0346	6.83 ± 0.02	89.81	8.34	0.508 ± 0.005
17D17868	7.9 %		0.1032843	65.1574	0.0002196	83.9307	202.8492	6.75 ± 0.03	87.13	5.00	0.554 ± 0.007
17D17869	9.0 %		0.1321356	65.8917	0.0000000	74.8152	177.5993	6.63 ± 0.04	82.29	4.45	0.488 ± 0.006
17D17871	10.3 %		0.1434948	78.7940	0.0562331	62.0874	144.3407	6.49 ± 0.05	77.70	3.70	0.339 ± 0.004
17D17872	11.6 %		0.1197804	85.6230	0.0495594	42.0450	94.2867	6.26 ± 0.07	73.19	2.50	0.211 ± 0.002
17D17873	12.5 %		0.0693530	54.1549	0.0000000	19.6582	43.2534	6.14 ± 0.09	68.40	1.17	0.156 ± 0.002
17D17875	13.4 %		0.0528943	41.5902	0.0138349	11.8859	25.7783	6.05 ± 0.11	62.86	0.71	0.123 ± 0.002
17D17876	14.6 %		0.0409362	40.9529	0.0326394	8.2193	17.6750	6.00 ± 0.14	60.00	0.49	0.086 ± 0.001
17D17877	16.0 %		0.0263907	35.5526	0.0000000	4.4244	9.2388	5.83 ± 0.21	54.89	0.26	0.054 ± 0.001
17D17879	17.6 %		0.0368203	83.1926	0.0306560	5.7852	11.5955	5.60 ± 0.20	52.26	0.34	0.030 ± 0.000
17D17880	19.3 %		0.0495130	109.5442	0.0307223	5.2693	9.8908	5.24 ± 0.26	41.00	0.31	0.021 ± 0.000
17D17882	21.0 %		0.0478307	129.6297	0.0218647	4.2633	7.7622	5.08 ± 0.33	36.09	0.25	0.014 ± 0.000
		Σ	1.9343074	2322.4347	0.2413609	1679.5242	4095.9229				

Information on Analysis

Project = **O-CONNOR (16-23)**
Sample = **MW14-DL3-5**
Material = **Groundmass**
Location = **Mozambique Ridge**
Region = **Indian Ocean**
Analyst = **Dan Miggins**
Irradiation = **17-OSU-01 (1B35-17)**
J = **0.00154622 ± 0.00000155**
FCT-NM = **28.201 ± 0.023 Ma**

Results	40(r)/39(k)	± 2σ	Age (Ma)	± 2σ	MSWD	39Ar(k) (%,n)	K/Ca ± 2σ
Age Plateau	2.47193	± 0.00250<br>± 0.10%	**6.90**	**± 0.02**<br>**± 0.22%**	1.35<br>19%	72.46<br>12	0.357 ± 0.036
			Full External Error	± 0.16	1.85	2σ Confidence Limit	
			Analytical Error	± 0.01	1.1618	Error Magnification	
Total Fusion Age	2.43874	± 0.00238<br>± 0.10%	**6.81**	**± 0.02**<br>**± 0.22%**		24	0.311 ± 0.001
			Full External Error	± 0.15			
			Analytical Error	± 0.01			

Normal Isochron			39(k)/36(a) ± 2σ	40(a+r)/36(a) ± 2σ	r.i.
17D17848	1.8 %	✓	505.68 ± 4.98	1539.66 ± 15.03	0.9897
17D17850	1.9 %	✓	673.46 ± 9.18	1950.40 ± 26.45	0.9929
17D17851	2.0 %	✓	922.79 ± 15.50	2562.10 ± 42.89	0.9959
17D17853	2.2 %	✓	1215.09 ± 16.73	3287.15 ± 45.06	0.9953
17D17854	2.4 %	✓	1459.65 ± 25.92	3886.35 ± 68.82	0.9968
17D17856	2.7 %	✓	1562.95 ± 38.35	4139.50 ± 101.42	0.9980
17D17857	3.0 %	✓	1733.02 ± 37.81	4575.87 ± 99.63	0.9977
17D17859	3.4 %	✓	1802.97 ± 30.60	4750.58 ± 80.39	0.9970
17D17860	3.9 %	✓	1842.09 ± 32.87	4845.70 ± 86.25	0.9972
17D17862	4.5 %	✓	1733.24 ± 34.41	4570.71 ± 90.53	0.9975
17D17863	5.2 %	✓	1570.95 ± 35.47	4179.26 ± 94.20	0.9976
17D17865	6.0 %	✓	1304.79 ± 20.73	3512.28 ± 55.60	0.9962
17D17866	6.9 %		1047.45 ± 12.80	2851.93 ± 34.64	0.9939
17D17868	7.9 %		812.62 ± 11.67	2251.02 ± 32.17	0.9949
17D17869	9.0 %		566.20 ± 6.91	1631.10 ± 19.78	0.9926
17D17871	10.3 %		432.68 ± 4.62	1292.92 ± 13.70	0.9891
17D17872	11.6 %		351.02 ± 4.36	1074.19 ± 13.24	0.9889
17D17873	12.5 %		283.45 ± 4.57	910.70 ± 14.54	0.9831
17D17875	13.4 %		224.71 ± 3.88	774.38 ± 13.11	0.9659
17D17876	14.6 %		200.78 ± 4.74	718.80 ± 16.58	0.9638
17D17877	16.0 %		167.65 ± 5.36	637.11 ± 19.43	0.9291
17D17879	17.6 %		157.12 ± 4.32	601.95 ± 15.97	0.9470
17D17880	19.3 %		106.42 ± 2.51	486.79 ± 10.82	0.9200
17D17882	21.0 %		89.13 ± 2.39	449.32 ± 11.21	0.9083

Results	40(a)/36(a) ± 2σ	40(r)/39(k) ± 2σ	Age ± 2σ (Ma)	MSWD
Normal Isochron<br>Error Chron	**285.30 ± 7.48<br>± 2.62%**	2.47333 ± 0.00634<br>± 0.26%	**6.90 ± 0.02<br>± 0.32%**<br>Full External Error ± 0.16<br>Analytical Error ± 0.02	3.13<br>0%

Statistics				
	2σ Confidence Limit	1.89	Convergence	0.000024718400
	Error Magnification	1.7691	Number of Iterations	54
	Number of Data Points	12	Calculated Line	Weighted York-2

Inverse Isochron			39(k)/40(a+r) ± 2σ	36(a)/40(a+r) ± 2σ	r.i.
17D17848	1.8 %	✓	0.3284342 ± 0.0004632	0.00064949 ± 0.00000634	0.0089
17D17850	1.9 %	✓	0.3452911 ± 0.0005590	0.00051271 ± 0.00000695	0.0142
17D17851	2.0 %	✓	0.3601688 ± 0.0005486	0.00039031 ± 0.00000653	0.0083
17D17853	2.2 %	✓	0.3696497 ± 0.0004947	0.00030421 ± 0.00000417	0.0040
17D17854	2.4 %	✓	0.3755839 ± 0.0005317	0.00025731 ± 0.00000456	0.0054
17D17856	2.7 %	✓	0.3775702 ± 0.0005893	0.00024158 ± 0.00000592	0.0073
17D17857	3.0 %	✓	0.3787290 ± 0.0005569	0.00021854 ± 0.00000476	0.0055
17D17859	3.4 %	✓	0.3795261 ± 0.0004976	0.00021050 ± 0.00000356	0.0022
17D17860	3.9 %	✓	0.3801486 ± 0.0005073	0.00020637 ± 0.00000367	0.0028
17D17862	4.5 %	✓	0.3792045 ± 0.0005341	0.00021878 ± 0.00000433	0.0048
17D17863	5.2 %	✓	0.3758907 ± 0.0005847	0.00023928 ± 0.00000539	0.0083
17D17865	6.0 %	✓	0.3714942 ± 0.0005139	0.00028472 ± 0.00000451	0.0048
17D17866	6.9 %		0.3672769 ± 0.0004947	0.00035064 ± 0.00000426	0.0040
17D17868	7.9 %		0.3610003 ± 0.0005252	0.00044424 ± 0.00000635	0.0077
17D17869	9.0 %		0.3471281 ± 0.0005160	0.00061308 ± 0.00000744	0.0112
17D17871	10.3 %		0.3346525 ± 0.0005274	0.00077344 ± 0.00000819	0.0147
17D17872	11.6 %		0.3267729 ± 0.0006038	0.00093093 ± 0.00001148	0.0212
17D17873	12.5 %		0.3112447 ± 0.0009202	0.00109806 ± 0.00001753	0.0374
17D17875	13.4 %		0.2901786 ± 0.0013008	0.00129135 ± 0.00002187	0.0542
17D17876	14.6 %		0.2793293 ± 0.0017582	0.00139121 ± 0.00003208	0.0520
17D17877	16.0 %		0.2631436 ± 0.0031198	0.00156960 ± 0.00004788	0.0669
17D17879	17.6 %		0.2610177 ± 0.0023062	0.00166127 ± 0.00004407	0.0578
17D17880	19.3 %		0.2186189 ± 0.0020243	0.00205427 ± 0.00004567	0.0577
17D17882	21.0 %		0.1983750 ± 0.0022314	0.00222561 ± 0.00005554	0.0504

Results	40(a)/36(a) ± 2σ	40(r)/39(k) ± 2σ	Age ± 2σ (Ma)	MSWD
Inverse Isochron<br>Error Chron	**287.03** **± 7.33**<br>**± 2.56%**	2.47186 ± 0.00623<br>± 0.25%	**6.90** **± 0.02**<br>**± 0.32%**<br>Full External Error ± 0.16<br>Analytical Error ± 0.02	3.02<br>0%

Statistics				
	2σ Confidence Limit	1.89	Convergence	0.0000124079
	Error Magnification	1.7383	Number of Iterations	3
	Number of Data Points	12	Calculated Line	Weighted York-2
	Spreading Factor	12.8%		

Degassing Patterns			36Ar(a) [fA]	%1σ	36Ar(c) [fA]	%1σ	36Ar(ca) [fA]	%1σ	36Ar(cl) [fA]	%1σ	37Ar(ca) [fA]	%1σ	38Ar(a) [fA]	%1σ	38Ar(c) [fA]	%1σ
17D17848	1.8 %	✓	0.1808578	0.49	0.0000000	0.00	0.0189515	0.60	0.0000000	0.00	71.1661	0.58	0.0338023	0.49	0.0000000	0.00
17D17850	1.9 %	✓	0.0826384	0.68	0.0000000	0.00	0.0125272	0.80	0.0000011	415.37	47.0418	0.78	0.0154451	0.68	0.0000000	0.00
17D17851	2.0 %	✓	0.0731545	0.84	0.0000000	0.00	0.0162810	0.67	0.0000000	0.00	61.1380	0.65	0.0136726	0.84	0.0000000	0.00
17D17853	2.2 %	✓	0.1141825	0.69	0.0000000	0.00	0.0375617	0.48	0.0000000	0.00	141.0503	0.45	0.0213407	0.69	0.0000000	0.00
17D17854	2.4 %	✓	0.0669815	0.89	0.0000000	0.00	0.0310060	0.50	0.0000000	0.00	116.4327	0.48	0.0125188	0.89	0.0000000	0.00
17D17856	2.7 %	✓	0.0423025	1.22	0.0000000	0.00	0.0229373	0.55	0.0000000	0.00	86.1335	0.53	0.0079063	1.22	0.0000000	0.00
17D17857	3.0 %	✓	0.0467656	1.09	0.0000000	0.00	0.0296587	0.51	0.0000000	0.00	111.3732	0.49	0.0087405	1.09	0.0000000	0.00
17D17859	3.4 %	✓	0.1045316	0.85	0.0000000	0.00	0.0701770	0.44	0.0000000	0.00	263.5261	0.42	0.0195370	0.85	0.0000000	0.00
17D17860	3.9 %	✓	0.0855927	0.89	0.0000000	0.00	0.0569557	0.45	0.0000000	0.00	213.8780	0.43	0.0159973	0.89	0.0000000	0.00
17D17862	4.5 %	✓	0.0597857	0.99	0.0000000	0.00	0.0340263	0.50	0.0000000	0.00	127.7742	0.47	0.0111740	0.99	0.0000000	0.00
17D17863	5.2 %	✓	0.0401345	1.13	0.0000000	0.00	0.0187757	0.61	0.0000000	0.00	70.5059	0.59	0.0075011	1.13	0.0000000	0.00
17D17865	6.0 %	✓	0.0812047	0.79	0.0000000	0.00	0.0276497	0.52	0.0000000	0.00	103.8292	0.50	0.0151772	0.79	0.0000000	0.00
17D17866	6.9 %		0.1337421	0.61	0.0000000	0.00	0.0315572	0.49	0.0000000	0.00	118.5025	0.47	0.0249964	0.61	0.0000000	0.00
17D17868	7.9 %		0.1032843	0.71	0.0000000	0.00	0.0173514	0.65	0.0000000	######	65.1574	0.63	0.0193038	0.71	0.0000000	0.00
17D17869	9.0 %		0.1321356	0.61	0.0000000	0.00	0.0175470	0.63	0.0000000	0.00	65.8917	0.61	0.0246961	0.61	0.0000000	0.00
17D17871	10.3 %		0.1434948	0.53	0.0000000	0.00	0.0209828	0.58	0.0000114	40.75	78.7940	0.56	0.0268192	0.53	0.0000000	0.00
17D17872	11.6 %		0.1197804	0.62	0.0000000	0.00	0.0228014	0.55	0.0000101	46.68	85.6230	0.53	0.0223870	0.62	0.0000000	0.00
17D17873	12.5 %		0.0693530	0.80	0.0000000	0.00	0.0144214	0.71	0.0000000	0.00	54.1549	0.69	0.0129621	0.80	0.0000000	0.00
17D17875	13.4 %		0.0528943	0.84	0.0000000	0.00	0.0110755	0.85	0.0000028	166.42	41.5902	0.84	0.0098859	0.84	0.0000000	0.00
17D17876	14.6 %		0.0409362	1.14	0.0000000	0.00	0.0109057	0.83	0.0000066	71.15	40.9529	0.81	0.0076510	1.14	0.0000000	0.00
17D17877	16.0 %		0.0263907	1.51	0.0000000	0.00	0.0094676	0.97	0.0000000	0.00	35.5526	0.96	0.0049324	1.51	0.0000000	0.00
17D17879	17.6 %		0.0368203	1.31	0.0000000	0.00	0.0221542	0.58	0.0000062	76.26	83.1926	0.56	0.0068817	1.31	0.0000000	0.00
17D17880	19.3 %		0.0495130	1.10	0.0000000	0.00	0.0291716	0.52	0.0000062	82.52	109.5442	0.50	0.0092540	1.10	0.0000000	0.00
17D17882	21.0 %		0.0478307	1.23	0.0000000	0.00	0.0345204	0.49	0.0000044	110.10	129.6297	0.47	0.0089396	1.23	0.0000000	0.00
		Σ	1.9343074	0.16	0.0000000	0.00	0.6184644	0.12	0.0000490	29.17	2322.4347	0.11	0.3615221	0.16	0.0000000	0.00
		Σ							2.5528208	0.13	2322.4347	0.11				

Degassing Patterns	38Ar(k) [fA]	%1σ	38Ar(ca) [fA]	%1σ	38Ar(cl) [fA]	%1σ	39Ar(k) [fA]	%1σ	39Ar(ca) [fA]	%1σ	40Ar(r) [fA]	%1σ	40Ar(a) [fA]	%1σ	40Ar(c) [fA]	%1σ	40Ar(k) [fA]	%1σ
17D17848	1.100301	0.17	0.0051097	12.83	0.0000000	0.00	91.4555	0.07	0.0480798	1.44	226.5476	0.31	51.91161	1.37	0.0000000	0.00	0.3496344	2.66
17D17850	0.669567	0.18	0.0033776	12.84	0.0056316	415.37	55.6535	0.08	0.0317814	1.53	137.4586	0.25	23.71971	1.45	0.0000000	0.00	0.2127631	2.66
17D17851	0.812164	0.18	0.0043897	12.84	0.0000000	0.00	67.5060	0.07	0.0413048	1.47	166.4312	0.19	20.99753	1.53	0.0000000	0.00	0.2580753	2.66
17D17853	1.669211	0.17	0.0101274	12.83	0.0000000	0.00	138.7425	0.07	0.0952936	1.40	342.5612	0.14	32.77380	1.45	0.0000000	0.00	0.5304124	2.66
17D17854	1.176265	0.17	0.0083599	12.83	0.0000000	0.00	97.7695	0.07	0.0786619	1.40	241.0877	0.13	19.22570	1.55	0.0000000	0.00	0.3737729	2.66
17D17856	0.795449	0.18	0.0061844	12.83	0.0000000	0.00	66.1167	0.07	0.0581918	1.42	162.9688	0.13	12.14207	1.77	0.0000000	0.00	0.2527640	2.66
17D17857	0.975058	0.17	0.0079966	12.83	0.0000000	0.00	81.0454	0.07	0.0752437	1.41	200.5701	0.11	13.42312	1.68	0.0000000	0.00	0.3098367	2.66
17D17859	2.267452	0.17	0.0189212	12.83	0.0000000	0.00	188.4674	0.06	0.1780383	1.38	466.5825	0.10	30.00371	1.53	0.0000000	0.00	0.7205110	2.66
17D17860	1.896918	0.17	0.0153564	12.83	0.0000000	0.00	157.6692	0.07	0.1444960	1.39	390.1890	0.10	24.56768	1.56	0.0000000	0.00	0.6027692	2.66
17D17862	1.246685	0.17	0.0091742	12.83	0.0000000	0.00	103.6227	0.07	0.0863243	1.40	256.1032	0.11	17.16030	1.62	0.0000000	0.00	0.3961497	2.66
17D17863	0.758543	0.18	0.0050623	12.83	0.0000000	0.00	63.0490	0.07	0.0476338	1.45	156.2126	0.13	11.51979	1.70	0.0000000	0.00	0.2410364	2.66
17D17865	1.274749	0.17	0.0074549	12.83	0.0000000	0.00	105.9554	0.07	0.0701470	1.41	261.9058	0.13	23.30820	1.50	0.0000000	0.00	0.4050674	2.66
17D17866	1.685395	0.17	0.0085085	12.83	0.0000000	0.00	140.0877	0.07	0.0800603	1.40	343.0346	0.16	38.38799	1.41	0.0000000	0.00	0.5355553	2.66
17D17868	1.009770	0.17	0.0046783	12.84	0.0002196	######	83.9307	0.07	0.0440204	1.46	202.8492	0.22	29.64568	1.46	0.0000000	0.00	0.3208671	2.66
17D17869	0.900102	0.17	0.0047310	12.83	0.0000000	0.00	74.8152	0.07	0.0445164	1.45	177.5993	0.30	37.92688	1.41	0.0000000	0.00	0.2860185	2.66
17D17871	0.746974	0.18	0.0056574	12.83	0.0562331	40.76	62.0874	0.07	0.0532332	1.43	144.3407	0.40	41.18733	1.38	0.0000000	0.00	0.2373602	2.66
17D17872	0.505843	0.18	0.0061477	12.83	0.0495594	46.69	42.0450	0.09	0.0578469	1.42	94.2867	0.52	34.38056	1.42	0.0000000	0.00	0.1607379	2.66
17D17873	0.236507	0.21	0.0038883	12.84	0.0000000	0.00	19.6582	0.13	0.0365870	1.49	43.2534	0.70	19.90639	1.51	0.0000000	0.00	0.0751531	2.66
17D17875	0.142999	0.26	0.0029862	12.85	0.0138349	166.42	11.8859	0.20	0.0280983	1.56	25.7783	0.92	15.18224	1.53	0.0000000	0.00	0.0454396	2.67
17D17876	0.098886	0.33	0.0029404	12.85	0.0326394	71.15	8.2193	0.28	0.0276677	1.55	17.6750	1.16	11.74993	1.72	0.0000000	0.00	0.0314222	2.68
17D17877	0.053230	0.56	0.0025527	12.86	0.0000000	0.00	4.4244	0.54	0.0240193	1.63	9.2388	1.68	7.57493	1.97	0.0000000	0.00	0.0169146	2.71
17D17879	0.069602	0.43	0.0059732	12.83	0.0306560	76.27	5.7852	0.40	0.0562049	1.43	11.5955	1.71	10.56854	1.83	0.0000000	0.00	0.0221168	2.69
17D17880	0.063395	0.46	0.0078653	12.83	0.0307223	82.52	5.2693	0.43	0.0740081	1.41	9.8908	2.46	14.21173	1.68	0.0000000	0.00	0.0201444	2.69
17D17882	0.051292	0.55	0.0093074	12.83	0.0218647	110.10	4.2633	0.53	0.0875778	1.40	7.7622	3.18	13.72885	1.78	0.0000000	0.00	0.0162986	2.71
Σ	20.206355	0.04	0.1667508	2.98	0.2413609	29.18	1679.5242	0.02	1.5690369	0.33	4095.9229	0.05	555.20426	0.33	0.0000000	0.00	6.4208209	0.67
Σ					20.975989	0.34			1681.0932	0.02							4657.5480	0.06

Additional Parameters			40Ar/39Ar	1σ	37Ar/39Ar	1σ	36Ar/39Ar	1σ	Time (days)	37Ar (decay)	39Ar (decay)	40Ar (moles)
17D17848	1.8 %	✓	3.046971	0.002145	0.777741	0.004560	0.002184	0.000010	124.887	11.813373	1.00088247	1.338E-11
17D17850	1.9 %	✓	2.898275	0.002342	0.844780	0.006635	0.001709	0.000010	124.901	11.816614	1.00088257	7.747E-12
17D17851	2.0 %	✓	2.778599	0.002112	0.905114	0.005954	0.001324	0.000009	124.908	11.818235	1.00088262	9.009E-12
17D17853	2.2 %	✓	2.707228	0.001807	1.015936	0.004661	0.001093	0.000006	124.922	11.821477	1.00088272	1.804E-11
17D17854	2.4 %	✓	2.664200	0.001881	1.189932	0.005771	0.001001	0.000006	124.928	11.823099	1.00088277	1.251E-11
17D17856	2.7 %	✓	2.650005	0.002063	1.301605	0.007010	0.000986	0.000008	124.942	11.826343	1.00088286	8.417E-12
17D17857	3.0 %	✓	2.641781	0.001938	1.372932	0.006797	0.000942	0.000006	124.949	11.827965	1.00088291	1.029E-11
17D17859	3.4 %	✓	2.636198	0.001723	1.396939	0.005912	0.000926	0.000004	124.963	11.831211	1.00088301	2.387E-11
17D17860	3.9 %	✓	2.631961	0.001751	1.355257	0.005852	0.000903	0.000005	124.970	11.832833	1.00088306	1.994E-11
17D17862	4.5 %	✓	2.638724	0.001854	1.232045	0.005877	0.000905	0.000006	124.984	11.836080	1.00088316	1.314E-11
17D17863	5.2 %	✓	2.662160	0.002066	1.117427	0.006653	0.000934	0.000007	124.991	11.837704	1.00088321	8.063E-12
17D17865	6.0 %	✓	2.693872	0.001859	0.979286	0.004960	0.001027	0.000006	125.005	11.840952	1.00088331	1.371E-11
17D17866	6.9 %		2.725007	0.001831	0.845434	0.004023	0.001179	0.000006	125.012	11.842576	1.00088336	1.833E-11
17D17868	7.9 %		2.772450	0.002013	0.775917	0.004917	0.001437	0.000009	125.026	11.845825	1.00088345	1.118E-11
17D17869	9.0 %		2.882889	0.002139	0.880202	0.005410	0.002000	0.000011	125.033	11.847450	1.00088350	1.036E-11
17D17871	10.3 %		2.989435	0.002351	1.267994	0.007185	0.002647	0.000012	125.047	11.850701	1.00088360	8.917E-12
17D17872	11.6 %		3.059843	0.002821	2.033664	0.011002	0.003387	0.000017	125.053	11.852326	1.00088365	6.184E-12
17D17873	12.5 %		3.210753	0.004736	2.749713	0.019311	0.004254	0.000028	125.060	11.853952	1.00088370	3.035E-12
17D17875	13.4 %		3.441840	0.007697	3.490881	0.030156	0.005370	0.000038	125.075	11.857367	1.00088380	1.968E-12
17D17876	14.6 %		3.571804	0.011206	4.965834	0.042783	0.006287	0.000059	125.082	11.858994	1.00088385	1.414E-12
17D17877	16.0 %		3.783489	0.022322	7.992136	0.087640	0.008061	0.000097	125.088	11.860458	1.00088389	8.079E-13
17D17879	17.6 %		3.798082	0.016634	14.241874	0.097412	0.010097	0.000089	125.103	11.863875	1.00088400	1.065E-12
17D17880	19.3 %		4.514585	0.020631	20.501340	0.133813	0.014727	0.000116	125.110	11.865502	1.00088405	1.158E-12
17D17882	21.0 %		4.943237	0.027267	29.793960	0.207771	0.018929	0.000163	125.124	11.868758	1.00088414	1.032E-12

Procedure Blanks		36Ar ± 1σ (SE) [fA]	37Ar ± 1σ (SE) [fA]	38Ar ± 1σ (SE) [fA]	39Ar ± 1σ (SE) [fA]	40Ar ± 1σ (SE) [fA]
17D17848	1.8 %	0.0041981 ± 0.0001559	0.1481332 ± 0.0180074	0.0473696 ± 0.0159277	0.0015760 ± 0.0161262	1.1355173 ± 0.0362646
17D17850	1.9 %	0.0041307 ± 0.0001559	0.1443825 ± 0.0180074	0.0510508 ± 0.0159277	0.0057788 ± 0.0161262	1.0991448 ± 0.0362646
17D17851	2.0 %	0.0040896 ± 0.0001559	0.1426761 ± 0.0180074	0.0522813 ± 0.0159277	0.0069819 ± 0.0161262	1.0853439 ± 0.0362646
17D17853	2.2 %	0.0040004 ± 0.0001559	0.1395558 ± 0.0180074	0.0533990 ± 0.0159277	0.0079127 ± 0.0161262	1.0647189 ± 0.0362646
17D17854	2.4 %	0.0039550 ± 0.0001559	0.1381265 ± 0.0180074	0.0532595 ± 0.0159277	0.0077611 ± 0.0161262	1.0572513 ± 0.0362646
17D17856	2.7 %	0.0038680 ± 0.0001559	0.1354990 ± 0.0180074	0.0515740 ± 0.0159277	0.0065059 ± 0.0161262	1.0465829 ± 0.0362646
17D17857	3.0 %	0.0038284 ± 0.0001559	0.1342913 ± 0.0180074	0.0500466 ± 0.0159277	0.0055075 ± 0.0161262	1.0428867 ± 0.0362646
17D17859	3.4 %	0.0037604 ± 0.0001559	0.1320719 ± 0.0180074	0.0457262 ± 0.0159277	0.0030129 ± 0.0161262	1.0377179 ± 0.0362646
17D17860	3.9 %	0.0037331 ± 0.0001559	0.1310567 ± 0.0180074	0.0429970 ± 0.0159277	0.0016063 ± 0.0161262	1.0358982 ± 0.0362646
17D17862	4.5 %	0.0036936 ± 0.0001559	0.1292134 ± 0.0180074	0.0366173 ± 0.0159277	0.0013205 ± 0.0161262	1.0331060 ± 0.0362646
17D17863	5.2 %	0.0036817 ± 0.0001559	0.1283878 ± 0.0180074	0.0330761 ± 0.0159277	0.0027666 ± 0.0161262	1.0319348 ± 0.0362646
17D17865	6.0 %	0.0036729 ± 0.0001559	0.1269416 ± 0.0180074	0.0256207 ± 0.0159277	0.0054580 ± 0.0161262	1.0297306 ± 0.0362646
17D17866	6.9 %	0.0036756 ± 0.0001559	0.1263292 ± 0.0180074	0.0218612 ± 0.0159277	0.0066447 ± 0.0161262	1.0286471 ± 0.0362646
17D17868	7.9 %	0.0036926 ± 0.0001559	0.1253537 ± 0.0180074	0.0147213 ± 0.0159277	0.0085723 ± 0.0161262	1.0265760 ± 0.0362646
17D17869	9.0 %	0.0037056 ± 0.0001559	0.1250046 ± 0.0180074	0.0115409 ± 0.0159277	0.0092701 ± 0.0161262	1.0256862 ± 0.0362646
17D17871	10.3 %	0.0037362 ± 0.0001559	0.1246263 ± 0.0180074	0.0065154 ± 0.0159277	0.0100451 ± 0.0161262	1.0246276 ± 0.0362646
17D17872	11.6 %	0.0037516 ± 0.0001559	0.1246169 ± 0.0180074	0.0049156 ± 0.0159277	0.0100946 ± 0.0161262	1.0247047 ± 0.0362646
17D17873	12.5 %	0.0037655 ± 0.0001559	0.1247424 ± 0.0180074	0.0041003 ± 0.0159277	0.0099045 ± 0.0161262	1.0253913 ± 0.0362646
17D17875	13.4 %	0.0037829 ± 0.0001559	0.1255010 ± 0.0180074	0.0055805 ± 0.0159277	0.0086959 ± 0.0161262	1.0296687 ± 0.0362646
17D17876	14.6 %	0.0037822 ± 0.0001559	0.1261275 ± 0.0180074	0.0081300 ± 0.0159277	0.0077277 ± 0.0161262	1.0335282 ± 0.0362646
17D17877	16.0 %	0.0037746 ± 0.0001559	0.1268549 ± 0.0180074	0.0116275 ± 0.0159277	0.0066395 ± 0.0161262	1.0382967 ± 0.0362646
17D17879	17.6 %	0.0037227 ± 0.0001559	0.1292247 ± 0.0180074	0.0249511 ± 0.0159277	0.0033175 ± 0.0161262	1.0554185 ± 0.0362646
17D17880	19.3 %	0.0036768 ± 0.0001559	0.1307211 ± 0.0180074	0.0342156 ± 0.0159277	0.0013648 ± 0.0161262	1.0671882 ± 0.0362646
17D17882	21.0 %	0.0035316 ± 0.0001559	0.1345242 ± 0.0180074	0.0593896 ± 0.0159277	0.0032029 ± 0.0161262	1.0995607 ± 0.0362646

**Intercept Values**		**36Ar ± 1σ (SE) [fA]**	**r2**	**Regression (type,n)**	**37Ar ± 1σ (SE) [fA]**	**r2**	**Regression (type,n)**	**38Ar ± 1σ (SE) [fA]**	**r2**	**Regression (type,n)**	**39Ar ± 1σ (SE) [fA]**	**r2**	**Regression (type,n)**	**40Ar ± 1σ (SE) [fA]**	**r2**	**Regression (type,n)**
17D17848	1.8 %	0.1966904 ± 0.0006685	0.2974	EXP 149 of 150	5.770945 ± 0.017361	0.7821	EXP 150 of 150	1.0652630 ± 0.0166903	0.1826	EXP 150 of 150	90.8929755 ± 0.0193258	0.9990	EXP 150 of 150	279.944319 ± 0.031257	0.9973	EXP 150 of 150
17D17850	1.9 %	0.0958125 ± 0.0004505	0.0899	EXP 150 of 150	3.767137 ± 0.019103	0.6635	EXP 150 of 150	0.6348863 ± 0.0166872	0.1076	EXP 150 of 150	55.3184572 ± 0.0176565	0.9976	EXP 150 of 150	162.490265 ± 0.026097	0.9678	EXP 150 of 150
17D17851	2.0 %	0.0902500 ± 0.0005141	0.1027	EXP 150 of 150	4.940246 ± 0.019113	0.6911	EXP 150 of 150	0.7339803 ± 0.0165136	0.0379	EXP 150 of 150	67.1023441 ± 0.0188936	0.9981	EXP 150 of 150	188.772161 ± 0.022252	0.9911	EXP 150 of 150
17D17853	2.2 %	0.1501877 ± 0.0006131	0.0130	EXP 150 of 150	11.583945 ± 0.017352	0.9364	EXP 150 of 150	1.5871284 ± 0.0171462	0.1944	EXP 150 of 150	137.9167743 ± 0.0220012	0.9994	EXP 150 of 150	376.930145 ± 0.033180	0.9989	EXP 150 of 150
17D17854	2.4 %	0.0983542 ± 0.0004710	0.2396	EXP 150 of 150	9.537934 ± 0.018173	0.9070	EXP 150 of 150	1.1085273 ± 0.0159611	0.1531	EXP 149 of 150	97.2012904 ± 0.0216567	0.9988	EXP 150 of 150	261.744433 ± 0.029411	0.9965	EXP 150 of 150
17D17856	2.7 %	0.0667187 ± 0.0004293	0.3689	EXP 149 of 150	7.020604 ± 0.017624	0.8475	EXP 150 of 150	0.7433375 ± 0.0160007	0.1108	EXP 150 of 150	65.7385962 ± 0.0195393	0.9979	EXP 150 of 150	176.410241 ± 0.027972	0.9818	EXP 150 of 150
17D17857	3.0 %	0.0774540 ± 0.0003996	0.4445	EXP 149 of 150	9.117495 ± 0.018854	0.8923	EXP 149 of 150	0.9076824 ± 0.0174654	0.0749	EXP 150 of 150	80.5834455 ± 0.0207267	0.9984	EXP 150 of 150	215.345966 ± 0.025026	0.9949	EXP 149 of 150
17D17859	3.4 %	0.1720712 ± 0.0006544	0.0040	EXP 150 of 150	21.753077 ± 0.018849	0.9779	EXP 150 of 150	2.2073374 ± 0.0174779	0.4004	EXP 150 of 150	187.3862984 ± 0.0263946	0.9995	EXP 150 of 150	498.344484 ± 0.036238	0.9994	EXP 150 of 150
17D17860	3.9 %	0.1410615 ± 0.0005750	0.0906	EXP 149 of 150	17.628514 ± 0.018719	0.9668	EXP 150 of 150	1.8273095 ± 0.0163387	0.3098	EXP 150 of 150	156.7593510 ± 0.0261219	0.9994	EXP 150 of 150	416.395320 ± 0.035523	0.9989	EXP 150 of 150
17D17862	4.5 %	0.0940703 ± 0.0004710	0.1692	EXP 150 of 150	10.477734 ± 0.019358	0.9100	EXP 150 of 150	1.2078646 ± 0.0148519	0.2632	EXP 149 of 150	103.0138279 ± 0.0225558	0.9989	EXP 150 of 150	274.692736 ± 0.032470	0.9968	EXP 150 of 150
17D17863	5.2 %	0.0604346 ± 0.0003640	0.5767	EXP 150 of 150	5.723732 ± 0.017909	0.7965	EXP 150 of 150	0.6899375 ± 0.0155417	0.0255	EXP 150 of 150	62.6717126 ± 0.0172628	0.9982	EXP 150 of 150	169.005317 ± 0.026180	0.9715	EXP 150 of 150
17D17865	6.0 %	0.1085412 ± 0.0005181	0.1810	EXP 150 of 150	8.488710 ± 0.018779	0.8610	EXP 150 of 150	1.2133469 ± 0.0168930	0.0804	EXP 150 of 150	105.3106263 ± 0.0206938	0.9991	EXP 150 of 150	286.648841 ± 0.026514	0.9983	EXP 150 of 150
17D17866	6.9 %	0.1629217 ± 0.0006335	0.0039	EXP 150 of 150	9.705547 ± 0.016374	0.9190	EXP 150 of 150	1.6532939 ± 0.0171160	0.1855	EXP 150 of 150	139.2232289 ± 0.0256210	0.9992	EXP 150 of 150	382.986755 ± 0.028986	0.9991	EXP 150 of 150
17D17868	7.9 %	0.1199107 ± 0.0006180	0.0059	EXP 150 of 150	5.279122 ± 0.019095	0.7402	EXP 150 of 150	1.0072072 ± 0.0155472	0.1101	EXP 150 of 150	83.4042615 ± 0.0207561	0.9986	EXP 150 of 150	233.842315 ± 0.027725	0.9956	EXP 150 of 150
17D17869	9.0 %	0.1479068 ± 0.0006521	0.0833	EXP 150 of 150	5.339625 ± 0.017566	0.7557	EXP 150 of 150	0.9055567 ± 0.0171000	0.0393	EXP 150 of 150	74.3495345 ± 0.0189735	0.9985	EXP 150 of 150	216.837920 ± 0.031018	0.9920	EXP 150 of 150
17D17871	10.3 %	0.1622017 ± 0.0005784	0.2948	EXP 149 of 150	6.408244 ± 0.018316	0.8052	EXP 150 of 150	0.8194340 ± 0.0159914	0.1132	EXP 150 of 150	61.7147938 ± 0.0195875	0.9976	EXP 150 of 150	186.790019 ± 0.027595	0.9898	EXP 150 of 150
17D17872	11.6 %	0.1411218 ± 0.0005858	0.2060	EXP 150 of 150	6.973478 ± 0.017401	0.8479	EXP 150 of 150	0.5722200 ± 0.0163376	0.0431	EXP 150 of 150	41.8109994 ± 0.0183291	0.9953	EXP 150 of 150	129.852720 ± 0.025872	0.7205	EXP 150 of 150
17D17873	12.5 %	0.0844721 ± 0.0004544	0.0181	EXP 150 of 150	4.364051 ± 0.017613	0.6822	EXP 150 of 150	0.2330156 ± 0.0162222	0.0011	EXP 150 of 150	19.5530511 ± 0.0159827	0.9828	EXP 150 of 150	64.260353 ± 0.021022	0.9902	EXP 150 of 150
17D17875	13.4 %	0.0654128 ± 0.0003554	0.0856	EXP 150 of 150	3.320837 ± 0.017990	0.5237	EXP 150 of 150	0.1621486 ± 0.0162410	0.0089	EXP 150 of 150	11.8255378 ± 0.0155598	0.9503	EXP 150 of 150	42.035606 ± 0.020231	0.9945	EXP 150 of 150
17D17876	14.6 %	0.0537321 ± 0.0003948	0.2378	EXP 150 of 150	3.266931 ± 0.015914	0.5973	EXP 149 of 150	0.1323313 ± 0.0165122	0.0221	EXP 150 of 150	8.1840093 ± 0.0157527	0.8901	EXP 150 of 150	30.489918 ± 0.017806	0.9968	EXP 150 of 150
17D17877	16.0 %	0.0383199 ± 0.0003266	0.4957	EXP 150 of 150	2.818411 ± 0.018155	0.4359	EXP 150 of 150	0.0331875 ± 0.0169213	0.0033	EXP 150 of 150	4.4120364 ± 0.0171498	0.6354	EXP 149 of 150	17.868935 ± 0.019862	0.9970	EXP 150 of 150
17D17879	17.6 %	0.0605436 ± 0.0003959	0.1915	EXP 150 of 150	6.760675 ± 0.019549	0.7953	EXP 150 of 150	0.0868440 ± 0.0167150	0.0260	EXP 150 of 150	5.7989982 ± 0.0160851	0.7990	EXP 150 of 150	23.241554 ± 0.018695	0.9966	EXP 150 of 150
17D17880	19.3 %	0.0794860 ± 0.0004377	0.0218	EXP 150 of 150	8.940345 ± 0.019533	0.8854	EXP 149 of 150	0.0757248 ± 0.0193101	0.0058	EXP 150 of 150	5.3061501 ± 0.0152902	0.7728	EXP 150 of 150	25.189838 ± 0.020217	0.9962	EXP 150 of 150
17D17882	21.0 %	0.0828713 ± 0.0004812	0.0243	EXP 150 of 150	10.596825 ± 0.018000	0.9197	EXP 150 of 150	0.0309491 ± 0.0176294	0.0015	EXP 150 of 150	4.3249595 ± 0.0153299	0.6347	EXP 150 of 150	22.606952 ± 0.017849	0.9972	EXP 150 of 150

Project Info		Analyst	Irradiation	X-pos	Y-pos	Z/H-pos	Project	Experiment	Nmb
17D17848	1.8 %	Dan Miggins	17-OSU-01	0.00	0.00	55.53	Mozambique Ridge\O-Connor (16-23)	17D17844	01
17D17850	1.9 %	Dan Miggins	17-OSU-01	0.00	0.00	55.53	Mozambique Ridge\O-Connor (16-23)	17D17844	01
17D17851	2.0 %	Dan Miggins	17-OSU-01	0.00	0.00	55.53	Mozambique Ridge\O-Connor (16-23)	17D17844	01
17D17853	2.2 %	Dan Miggins	17-OSU-01	0.00	0.00	55.53	Mozambique Ridge\O-Connor (16-23)	17D17844	01
17D17854	2.4 %	Dan Miggins	17-OSU-01	0.00	0.00	55.53	Mozambique Ridge\O-Connor (16-23)	17D17844	01
17D17856	2.7 %	Dan Miggins	17-OSU-01	0.00	0.00	55.53	Mozambique Ridge\O-Connor (16-23)	17D17844	01
17D17857	3.0 %	Dan Miggins	17-OSU-01	0.00	0.00	55.53	Mozambique Ridge\O-Connor (16-23)	17D17844	01
17D17859	3.4 %	Dan Miggins	17-OSU-01	0.00	0.00	55.53	Mozambique Ridge\O-Connor (16-23)	17D17844	01
17D17860	3.9 %	Dan Miggins	17-OSU-01	0.00	0.00	55.53	Mozambique Ridge\O-Connor (16-23)	17D17844	01
17D17862	4.5 %	Dan Miggins	17-OSU-01	0.00	0.00	55.53	Mozambique Ridge\O-Connor (16-23)	17D17844	01
17D17863	5.2 %	Dan Miggins	17-OSU-01	0.00	0.00	55.53	Mozambique Ridge\O-Connor (16-23)	17D17844	01
17D17865	6.0 %	Dan Miggins	17-OSU-01	0.00	0.00	55.53	Mozambique Ridge\O-Connor (16-23)	17D17844	01
17D17866	6.9 %	Dan Miggins	17-OSU-01	0.00	0.00	55.53	Mozambique Ridge\O-Connor (16-23)	17D17844	01
17D17868	7.9 %	Dan Miggins	17-OSU-01	0.00	0.00	55.53	Mozambique Ridge\O-Connor (16-23)	17D17844	01
17D17869	9.0 %	Dan Miggins	17-OSU-01	0.00	0.00	55.53	Mozambique Ridge\O-Connor (16-23)	17D17844	01
17D17871	10.3 %	Dan Miggins	17-OSU-01	0.00	0.00	55.53	Mozambique Ridge\O-Connor (16-23)	17D17844	01
17D17872	11.6 %	Dan Miggins	17-OSU-01	0.00	0.00	55.53	Mozambique Ridge\O-Connor (16-23)	17D17844	01
17D17873	12.5 %	Dan Miggins	17-OSU-01	0.00	0.00	55.53	Mozambique Ridge\O-Connor (16-23)	17D17844	01
17D17875	13.4 %	Dan Miggins	17-OSU-01	0.00	0.00	55.53	Mozambique Ridge\O-Connor (16-23)	17D17844	01
17D17876	14.6 %	Dan Miggins	17-OSU-01	0.00	0.00	55.53	Mozambique Ridge\O-Connor (16-23)	17D17844	01
17D17877	16.0 %	Dan Miggins	17-OSU-01	0.00	0.00	55.53	Mozambique Ridge\O-Connor (16-23)	17D17844	01
17D17879	17.6 %	Dan Miggins	17-OSU-01	0.00	0.00	55.53	Mozambique Ridge\O-Connor (16-23)	17D17844	01
17D17880	19.3 %	Dan Miggins	17-OSU-01	0.00	0.00	55.53	Mozambique Ridge\O-Connor (16-23)	17D17844	01
17D17882	21.0 %	Dan Miggins	17-OSU-01	0.00	0.00	55.53	Mozambique Ridge\O-Connor (16-23)	17D17844	01

**Sample Parameters**		Sample	Material	Location	Standard Name	Standard (in Ma)	%1σ	Standard Reference	Standard 40Ar/39Ar	%1σ	J	%1σ	Air 40Ar/36Ar	%1σ	MDF (lin)	%1σ	Volume Ratio	Sensitivity (mol/volt)	Day	Month	Year	Hour	Min	Resist
17D17848	1.8 %	MW14-DL3-5	Groundmass	Mozambique Ridge	FCT-NM (1B35-17)	28.201	0.082	Kuiper et al (2008)	10.16502	0.1	0.00154622	0.100	302.54	0.092	0.9941751	0.063	1	4.8E-14	24	MAY	2017	12	56	1
17D17850	1.9 %	MW14-DL3-5	Groundmass	Mozambique Ridge	FCT-NM (1B35-17)	28.201	0.082	Kuiper et al (2008)	10.16502	0.1	0.00154622	0.100	302.54	0.092	0.9941751	0.063	1	4.8E-14	24	MAY	2017	13	16	1
17D17851	2.0 %	MW14-DL3-5	Groundmass	Mozambique Ridge	FCT-NM (1B35-17)	28.201	0.082	Kuiper et al (2008)	10.16502	0.1	0.00154622	0.100	302.54	0.092	0.9941751	0.063	1	4.8E-14	24	MAY	2017	13	26	1
17D17853	2.2 %	MW14-DL3-5	Groundmass	Mozambique Ridge	FCT-NM (1B35-17)	28.201	0.082	Kuiper et al (2008)	10.16502	0.1	0.00154622	0.100	302.54	0.092	0.9941751	0.063	1	4.8E-14	24	MAY	2017	13	46	1
17D17854	2.4 %	MW14-DL3-5	Groundmass	Mozambique Ridge	FCT-NM (1B35-17)	28.201	0.082	Kuiper et al (2008)	10.16502	0.1	0.00154622	0.100	302.54	0.092	0.9941751	0.063	1	4.8E-14	24	MAY	2017	13	56	1
17D17856	2.7 %	MW14-DL3-5	Groundmass	Mozambique Ridge	FCT-NM (1B35-17)	28.201	0.082	Kuiper et al (2008)	10.16502	0.1	0.00154622	0.100	302.54	0.092	0.9941751	0.063	1	4.8E-14	24	MAY	2017	14	16	1
17D17857	3.0 %	MW14-DL3-5	Groundmass	Mozambique Ridge	FCT-NM (1B35-17)	28.201	0.082	Kuiper et al (2008)	10.16502	0.1	0.00154622	0.100	302.54	0.092	0.9941751	0.063	1	4.8E-14	24	MAY	2017	14	26	1
17D17859	3.4 %	MW14-DL3-5	Groundmass	Mozambique Ridge	FCT-NM (1B35-17)	28.201	0.082	Kuiper et al (2008)	10.16502	0.1	0.00154622	0.100	302.54	0.092	0.9941751	0.063	1	4.8E-14	24	MAY	2017	14	46	1
17D17860	3.9 %	MW14-DL3-5	Groundmass	Mozambique Ridge	FCT-NM (1B35-17)	28.201	0.082	Kuiper et al (2008)	10.16502	0.1	0.00154622	0.100	302.54	0.092	0.9941751	0.063	1	4.8E-14	24	MAY	2017	14	56	1
17D17862	4.5 %	MW14-DL3-5	Groundmass	Mozambique Ridge	FCT-NM (1B35-17)	28.201	0.082	Kuiper et al (2008)	10.16502	0.1	0.00154622	0.100	302.54	0.092	0.9941751	0.063	1	4.8E-14	24	MAY	2017	15	16	1
17D17863	5.2 %	MW14-DL3-5	Groundmass	Mozambique Ridge	FCT-NM (1B35-17)	28.201	0.082	Kuiper et al (2008)	10.16502	0.1	0.00154622	0.100	302.54	0.092	0.9941751	0.063	1	4.8E-14	24	MAY	2017	15	26	1
17D17865	6.0 %	MW14-DL3-5	Groundmass	Mozambique Ridge	FCT-NM (1B35-17)	28.201	0.082	Kuiper et al (2008)	10.16502	0.1	0.00154622	0.100	302.54	0.092	0.9941751	0.063	1	4.8E-14	24	MAY	2017	15	46	1
17D17866	6.9 %	MW14-DL3-5	Groundmass	Mozambique Ridge	FCT-NM (1B35-17)	28.201	0.082	Kuiper et al (2008)	10.16502	0.1	0.00154622	0.100	302.54	0.092	0.9941751	0.063	1	4.8E-14	24	MAY	2017	15	56	1
17D17868	7.9 %	MW14-DL3-5	Groundmass	Mozambique Ridge	FCT-NM (1B35-17)	28.201	0.082	Kuiper et al (2008)	10.16502	0.1	0.00154622	0.100	302.54	0.092	0.9941751	0.063	1	4.8E-14	24	MAY	2017	16	16	1
17D17869	9.0 %	MW14-DL3-5	Groundmass	Mozambique Ridge	FCT-NM (1B35-17)	28.201	0.082	Kuiper et al (2008)	10.16502	0.1	0.00154622	0.100	302.54	0.092	0.9941751	0.063	1	4.8E-14	24	MAY	2017	16	26	1
17D17871	10.3 %	MW14-DL3-5	Groundmass	Mozambique Ridge	FCT-NM (1B35-17)	28.201	0.082	Kuiper et al (2008)	10.16502	0.1	0.00154622	0.100	302.54	0.092	0.9941751	0.063	1	4.8E-14	24	MAY	2017	16	46	1
17D17872	11.6 %	MW14-DL3-5	Groundmass	Mozambique Ridge	FCT-NM (1B35-17)	28.201	0.082	Kuiper et al (2008)	10.16502	0.1	0.00154622	0.100	302.54	0.092	0.9941751	0.063	1	4.8E-14	24	MAY	2017	16	56	1
17D17873	12.5 %	MW14-DL3-5	Groundmass	Mozambique Ridge	FCT-NM (1B35-17)	28.201	0.082	Kuiper et al (2008)	10.16502	0.1	0.00154622	0.100	302.54	0.092	0.9941751	0.063	1	4.8E-14	24	MAY	2017	17	6	1
17D17875	13.4 %	MW14-DL3-5	Groundmass	Mozambique Ridge	FCT-NM (1B35-17)	28.201	0.082	Kuiper et al (2008)	10.16502	0.1	0.00154622	0.100	302.54	0.092	0.9941751	0.063	1	4.8E-14	24	MAY	2017	17	27	1
17D17876	14.6 %	MW14-DL3-5	Groundmass	Mozambique Ridge	FCT-NM (1B35-17)	28.201	0.082	Kuiper et al (2008)	10.16502	0.1	0.00154622	0.100	302.54	0.092	0.9941751	0.063	1	4.8E-14	24	MAY	2017	17	37	1
17D17877	16.0 %	MW14-DL3-5	Groundmass	Mozambique Ridge	FCT-NM (1B35-17)	28.201	0.082	Kuiper et al (2008)	10.16502	0.1	0.00154622	0.100	302.54	0.092	0.9941751	0.063	1	4.8E-14	24	MAY	2017	17	46	1
17D17879	17.6 %	MW14-DL3-5	Groundmass	Mozambique Ridge	FCT-NM (1B35-17)	28.201	0.082	Kuiper et al (2008)	10.16502	0.1	0.00154622	0.100	302.54	0.092	0.9941751	0.063	1	4.8E-14	24	MAY	2017	18	7	1
17D17880	19.3 %	MW14-DL3-5	Groundmass	Mozambique Ridge	FCT-NM (1B35-17)	28.201	0.082	Kuiper et al (2008)	10.16502	0.1	0.00154622	0.100	302.54	0.092	0.9941751	0.063	1	4.8E-14	24	MAY	2017	18	17	1
17D17882	21.0 %	MW14-DL3-5	Groundmass	Mozambique Ridge	FCT-NM (1B35-17)	28.201	0.082	Kuiper et al (2008)	10.16502	0.1	0.00154622	0.100	302.54	0.092	0.9941751	0.063	1	4.8E-14	24	MAY	2017	18	37	1

Irradiation Constants		40/36(a)	%1σ	40/36(c)	%1σ	38/36(a)	%1σ	38/36(c)	%1σ	39/37(ca)	%1σ	38/37(ca)	%1σ	36/37(ca)	%1σ	40/39(k)	%1σ	38/39(k)	%1σ	36/38(cl)	%1σ	K/Ca	%1σ	K/Cl	%1σ	Ca/Cl	%1σ
17D17848	1.8 %	287.03	1.278	0.018	35	0.1869	0	1.493	3	0.000676	1.32	7.18E-05	12.82	0.000266	0.15	0.003823	2.66	0.012031	0.16	0	0	0.43	0	0	0	0	0
17D17850	1.9 %	287.03	1.278	0.018	35	0.1869	0	1.493	3	0.000676	1.32	7.18E-05	12.82	0.000266	0.15	0.003823	2.66	0.012031	0.16	0	0	0.43	0	0	0	0	0
17D17851	2.0 %	287.03	1.278	0.018	35	0.1869	0	1.493	3	0.000676	1.32	7.18E-05	12.82	0.000266	0.15	0.003823	2.66	0.012031	0.16	0	0	0.43	0	0	0	0	0
17D17853	2.2 %	287.03	1.278	0.018	35	0.1869	0	1.493	3	0.000676	1.32	7.18E-05	12.82	0.000266	0.15	0.003823	2.66	0.012031	0.16	0	0	0.43	0	0	0	0	0
17D17854	2.4 %	287.03	1.278	0.018	35	0.1869	0	1.493	3	0.000676	1.32	7.18E-05	12.82	0.000266	0.15	0.003823	2.66	0.012031	0.16	0	0	0.43	0	0	0	0	0
17D17856	2.7 %	287.03	1.278	0.018	35	0.1869	0	1.493	3	0.000676	1.32	7.18E-05	12.82	0.000266	0.15	0.003823	2.66	0.012031	0.16	0	0	0.43	0	0	0	0	0
17D17857	3.0 %	287.03	1.278	0.018	35	0.1869	0	1.493	3	0.000676	1.32	7.18E-05	12.82	0.000266	0.15	0.003823	2.66	0.012031	0.16	0	0	0.43	0	0	0	0	0
17D17859	3.4 %	287.03	1.278	0.018	35	0.1869	0	1.493	3	0.000676	1.32	7.18E-05	12.82	0.000266	0.15	0.003823	2.66	0.012031	0.16	0	0	0.43	0	0	0	0	0
17D17860	3.9 %	287.03	1.278	0.018	35	0.1869	0	1.493	3	0.000676	1.32	7.18E-05	12.82	0.000266	0.15	0.003823	2.66	0.012031	0.16	0	0	0.43	0	0	0	0	0
17D17862	4.5 %	287.03	1.278	0.018	35	0.1869	0	1.493	3	0.000676	1.32	7.18E-05	12.82	0.000266	0.15	0.003823	2.66	0.012031	0.16	0	0	0.43	0	0	0	0	0
17D17863	5.2 %	287.03	1.278	0.018	35	0.1869	0	1.493	3	0.000676	1.32	7.18E-05	12.82	0.000266	0.15	0.003823	2.66	0.012031	0.16	0	0	0.43	0	0	0	0	0
17D17865	6.0 %	287.03	1.278	0.018	35	0.1869	0	1.493	3	0.000676	1.32	7.18E-05	12.82	0.000266	0.15	0.003823	2.66	0.012031	0.16	0	0	0.43	0	0	0	0	0
17D17866	6.9 %	287.03	1.278	0.018	35	0.1869	0	1.493	3	0.000676	1.32	7.18E-05	12.82	0.000266	0.15	0.003823	2.66	0.012031	0.16	0	0	0.43	0	0	0	0	0
17D17868	7.9 %	287.03	1.278	0.018	35	0.1869	0	1.493	3	0.000676	1.32	7.18E-05	12.82	0.000266	0.15	0.003823	2.66	0.012031	0.16	0	0	0.43	0	0	0	0	0
17D17869	9.0 %	287.03	1.278	0.018	35	0.1869	0	1.493	3	0.000676	1.32	7.18E-05	12.82	0.000266	0.15	0.003823	2.66	0.012031	0.16	0	0	0.43	0	0	0	0	0
17D17871	10.3 %	287.03	1.278	0.018	35	0.1869	0	1.493	3	0.000676	1.32	7.18E-05	12.82	0.000266	0.15	0.003823	2.66	0.012031	0.16	0	0	0.43	0	0	0	0	0
17D17872	11.6 %	287.03	1.278	0.018	35	0.1869	0	1.493	3	0.000676	1.32	7.18E-05	12.82	0.000266	0.15	0.003823	2.66	0.012031	0.16	0	0	0.43	0	0	0	0	0
17D17873	12.5 %	287.03	1.278	0.018	35	0.1869	0	1.493	3	0.000676	1.32	7.18E-05	12.82	0.000266	0.15	0.003823	2.66	0.012031	0.16	0	0	0.43	0	0	0	0	0
17D17875	13.4 %	287.03	1.278	0.018	35	0.1869	0	1.493	3	0.000676	1.32	7.18E-05	12.82	0.000266	0.15	0.003823	2.66	0.012031	0.16	0	0	0.43	0	0	0	0	0
17D17876	14.6 %	287.03	1.278	0.018	35	0.1869	0	1.493	3	0.000676	1.32	7.18E-05	12.82	0.000266	0.15	0.003823	2.66	0.012031	0.16	0	0	0.43	0	0	0	0	0
17D17877	16.0 %	287.03	1.278	0.018	35	0.1869	0	1.493	3	0.000676	1.32	7.18E-05	12.82	0.000266	0.15	0.003823	2.66	0.012031	0.16	0	0	0.43	0	0	0	0	0
17D17879	17.6 %	287.03	1.278	0.018	35	0.1869	0	1.493	3	0.000676	1.32	7.18E-05	12.82	0.000266	0.15	0.003823	2.66	0.012031	0.16	0	0	0.43	0	0	0	0	0
17D17880	19.3 %	287.03	1.278	0.018	35	0.1869	0	1.493	3	0.000676	1.32	7.18E-05	12.82	0.000266	0.15	0.003823	2.66	0.012031	0.16	0	0	0.43	0	0	0	0	0
17D17882	21.0 %	287.03	1.278	0.018	35	0.1869	0	1.493	3	0.000676	1.32	7.18E-05	12.82	0.000266	0.15	0.003823	2.66	0.012031	0.16	0	0	0.43	0	0	0	0	0

17D17844.AGE >>> MW14-DL3-5 >>> MOZAMBIQUE RIDGE | O-CONNOR (16-23) PROJECT

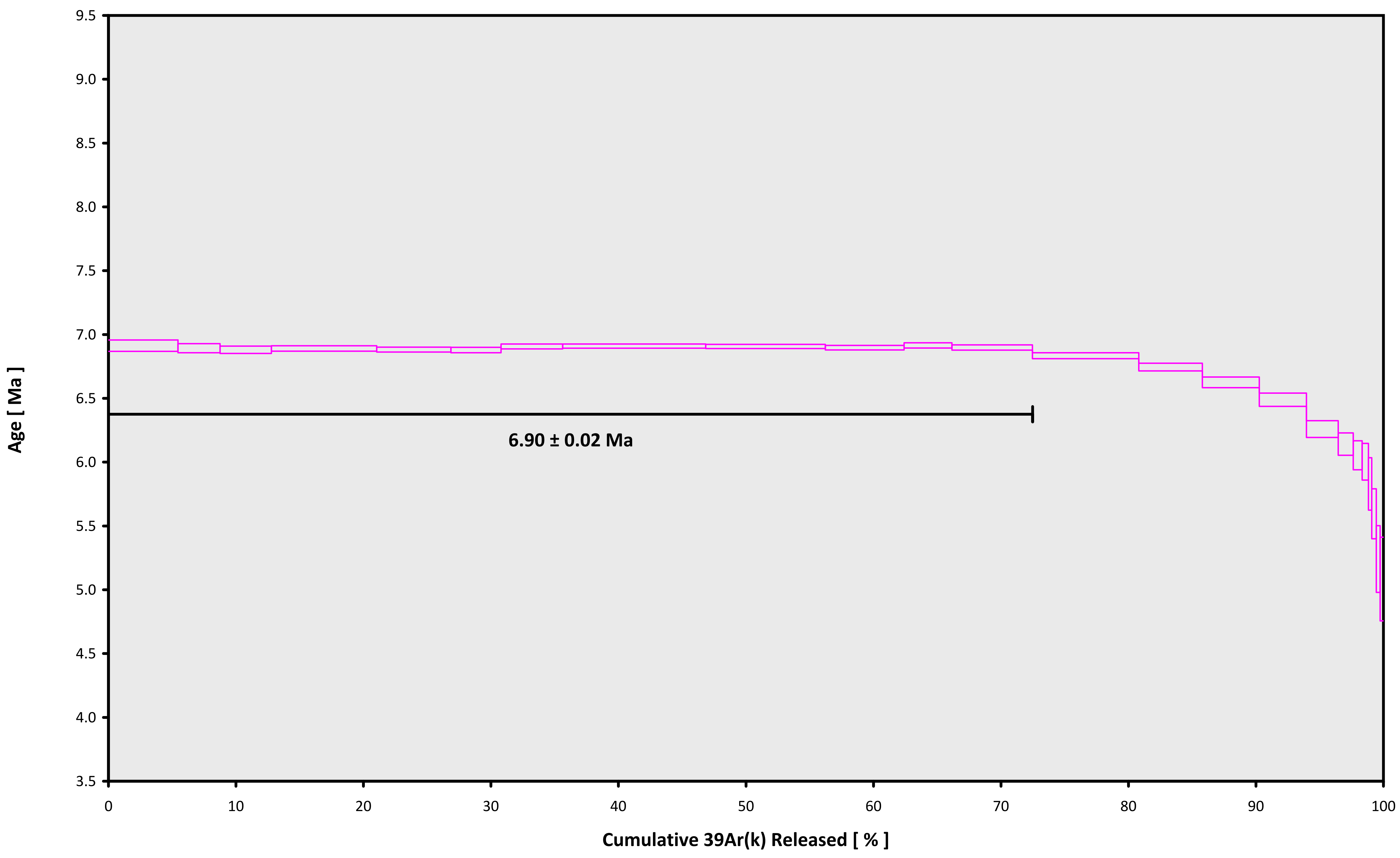

Ar-Ages in Ma

WEIGHTED PLATEAU
6.90 ± 0.02
TOTAL FUSION
6.81 ± 0.02
NORMAL ISOCHRON
6.90 ± 0.02
INVERSE ISOCHRON
6.90 ± 0.02

MSWD (PROBABILITY)
1.35 (19%)

Sample Info

Groundmass
Mozambique Ridge
Dan Miggins

IRR = 17-OSU-01 (1B35-17)
J = 0.00154622 ± 0.00000155

17D17844.AGE >>> MW14-DL3-5 >>> MOZAMBIQUE RIDGE | O-CONNOR (16-23) PROJECT

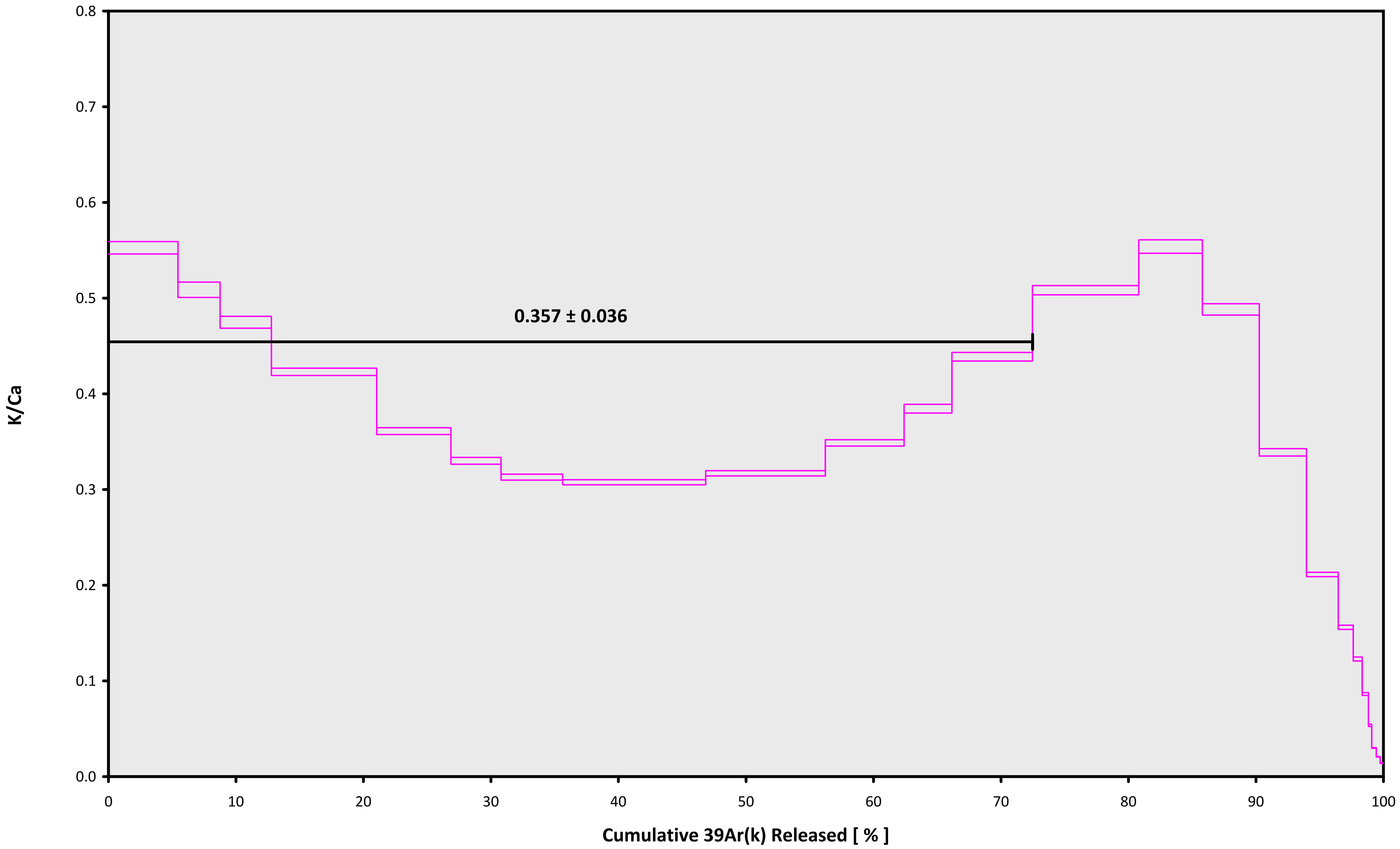

Ar-Ages in Ma

WEIGHTED PLATEAU
6.90 ± 0.02
TOTAL FUSION
6.81 ± 0.02
NORMAL ISOCHRON
6.90 ± 0.02
INVERSE ISOCHRON
6.90 ± 0.02

Sample Info

Groundmass
Mozambique Ridge
Dan Miggins

IRR = 17-OSU-01 (1B35-17)
J = 0.00154622 ± 0.00000155

17D17844.AGE >>> MW14-DL3-5 >>> MOZAMBIQUE RIDGE | O-CONNOR (16-23) PROJECT

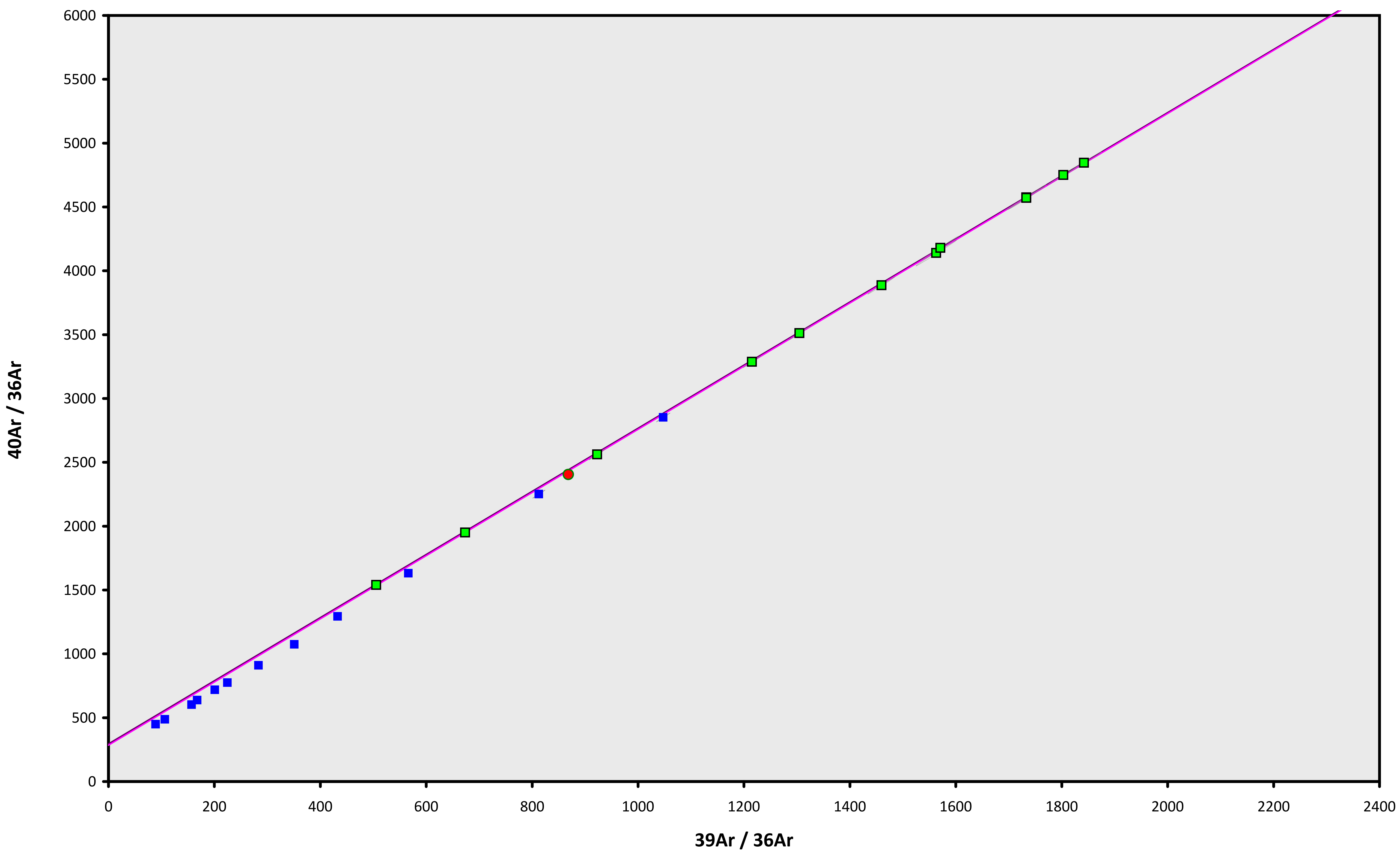

Ar-Ages in Ma

WEIGHTED PLATEAU
6.90 ± 0.02
TOTAL FUSION
6.81 ± 0.02
NORMAL ISOCHRON
6.90 ± 0.02
INVERSE ISOCHRON
6.90 ± 0.02

MSWD (PROBABILITY)
3.13 (0%)

40AR/36AR INTERCEPT
285.3 ± 7.5

Sample Info

Groundmass
Mozambique Ridge
Dan Miggins

IRR = 17-OSU-01 (1B35-17)
J = 0.00154622 ± 0.00000155

17D17844.AGE >>> MW14-DL3-5 >>> MOZAMBIQUE RIDGE | O-CONNOR (16-23) PROJECT

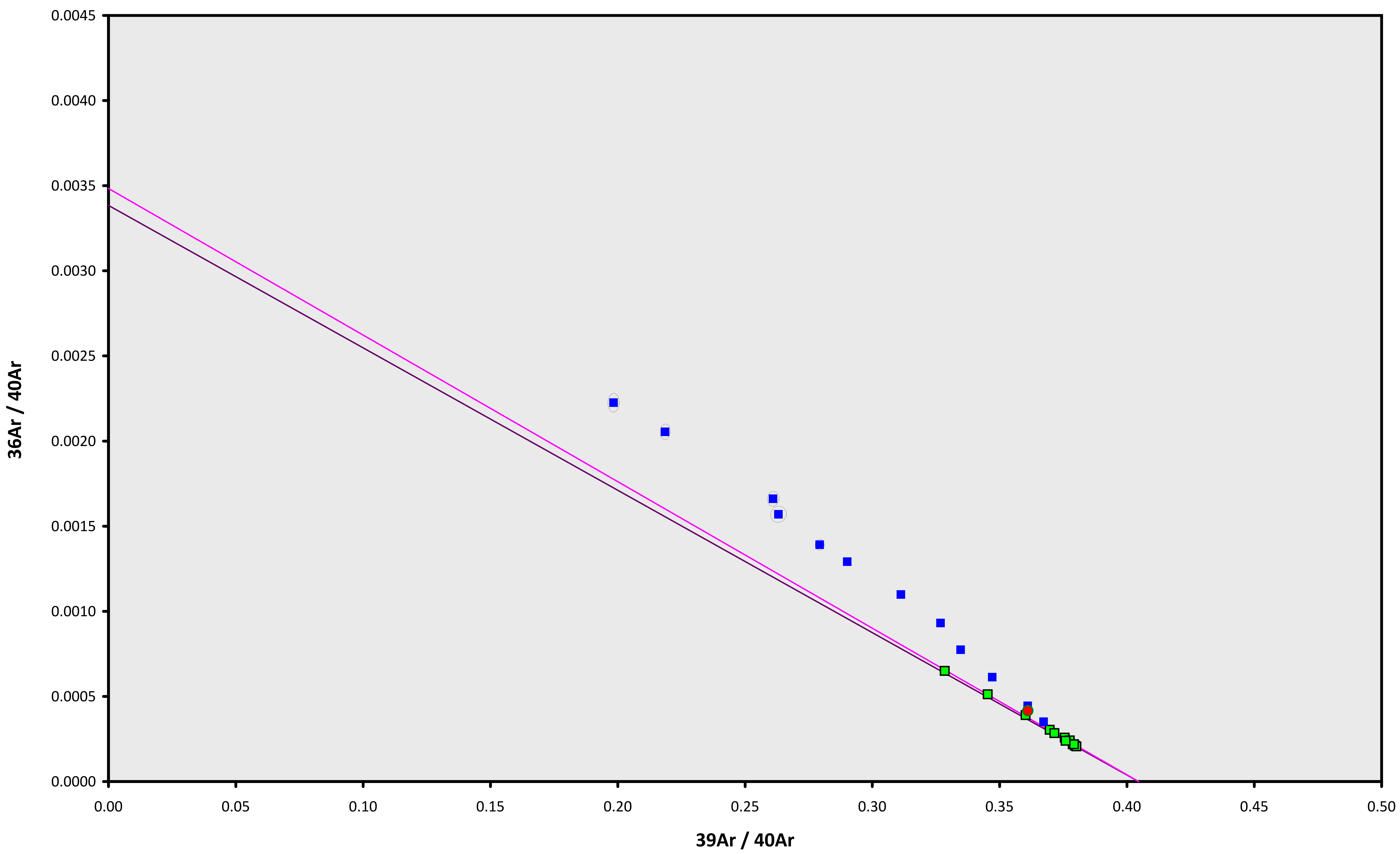

Ar-Ages in Ma

WEIGHTED PLATEAU
6.90 ± 0.02
TOTAL FUSION
6.81 ± 0.02
NORMAL ISOCHRON
6.90 ± 0.02
INVERSE ISOCHRON
6.90 ± 0.02

MSWD (PROBABILITY)
3.02 (0%)

SPREADING FACTOR
12.8%

40AR/36AR INTERCEPT
287.0 ± 7.3

Sample Info

Groundmass
Mozambique Ridge
Dan Miggins

IRR = 17-OSU-01 (1B35-17)
J = 0.00154622 ± 0.00000155

Incremental Heating			36Ar(a) [fA]	37Ar(ca) [fA]	38Ar(cl) [fA]	39Ar(k) [fA]	40Ar(r) [fA]	Age ± 2σ (Ma)	40Ar(r) (%)	39Ar(k) (%)	K/Ca ± 2σ
055_VU107-J-3	12 °C		0.0750532	16.8742	0.0000000	26.00875	22.14301	7.30 ± 0.12	49.68	3.25	0.663 ± 0.012
056_VU107-J-3	13 °C	4	0.0511301	39.8087	0.0000000	53.80048	44.08857	7.03 ± 0.06	74.22	6.73	0.581 ± 0.010
057_VU107-J-3	14 °C	4	0.0351359	66.4062	0.0000000	73.57887	59.75788	6.96 ± 0.04	84.99	9.20	0.476 ± 0.008
059_VU107-J-3	15 °C	4	0.0271474	100.8101	0.0000000	87.99133	71.18342	6.94 ± 0.04	89.69	11.00	0.375 ± 0.006
060_VU107-J-3	17 °C	4	0.0223841	126.2571	0.0000000	94.92703	76.69419	6.93 ± 0.04	91.89	11.87	0.323 ± 0.005
061_VU107-J-3	18 °C	4	0.0193923	118.6930	0.0000000	82.16472	66.44261	6.93 ± 0.04	91.89	10.27	0.298 ± 0.005
063_VU107-J-3	19 °C	4	0.0188004	105.1487	0.0000000	70.08148	56.47013	6.91 ± 0.05	90.87	8.76	0.287 ± 0.005
064_VU107-J-3	21 °C	4	0.0227033	98.4922	0.0000000	67.79766	54.84262	6.93 ± 0.05	88.92	8.48	0.296 ± 0.005
065_VU107-J-3	22 °C	4	0.0236431	83.1993	0.0014956	53.70576	43.70762	6.98 ± 0.05	86.02	6.71	0.278 ± 0.005
067_VU107-J-3	25 °C		0.0291117	76.0654	0.0138185	51.01319	40.74757	6.85 ± 0.05	82.35	6.38	0.288 ± 0.005
068_VU107-J-3	28 °C		0.0328395	116.6208	0.0265717	46.72203	37.48120	6.88 ± 0.07	79.20	5.84	0.172 ± 0.003
069_VU107-J-3	31 °C		0.0272288	133.0965	0.0299745	32.82535	26.36984	6.89 ± 0.09	76.37	4.10	0.106 ± 0.002
071_VU107-J-3	39 °C		0.0294184	238.2541	0.0306651	27.69840	23.78648	7.36 ± 0.18	72.98	3.46	0.050 ± 0.001
072_VU107-J-3	69 °C		0.0310022	420.4011	0.0245431	26.68054	33.13610	10.64 ± 0.28	78.12	3.34	0.027 ± 0.000
073_VU107-J-3	16 °C		0.0154178	122.9531	0.0114454	4.94762	2.70750	4.69 ± 0.62	37.01	0.62	0.017 ± 0.000
		Σ	0.4604081	1863.0806	0.1385138	799.94322	659.55875				

Information on Analysis

Sample = 055_VU107-J-3
Material = groundmass
Location = MW14 DL3-5
Analyst = Klaudia Kuiper
Project = VU107
Mass Discrimination Law = LIN
Irradiation = VU107
J = 0.00468970 ± 0.00000469
FCs = 28.201 ± 0.023 Ma

Results	40(r)/39(k)	± 2σ	Age (Ma)	± 2σ	MSWD	39Ar(k) (%,n)	K/Ca ± 2σ
**Age Plateau**	0.81002	± 0.00255<br>± 0.32%	6.94	± 0.03<br>± 0.37%	1.87<br>7%	73.01<br>8	0.325 ± 0.054
			Full External Error	± 0.15	2.07	2σ Confidence Limit	
			Analytical Error	± 0.02	1.3685	Error Magnification	
**Total Fusion Age**	0.82451	± 0.00214<br>± 0.26%	7.07	± 0.02<br>± 0.33%		15	0.185 ± 0.001
			Full External Error	± 0.15			
			Analytical Error	± 0.02			

Normal Isochron			39(k)/36(a) ± 2σ	40(a+r)/36(a) ± 2σ	r.i.
055_VU107-J-3	12 °C		346.54 ± 5.52	593.59 ± 9.26	0.9732
056_VU107-J-3	13 °C	4	1052.23 ± 24.70	1160.84 ± 27.02	0.9890
057_VU107-J-3	14 °C	4	2094.12 ± 60.73	1999.33 ± 57.69	0.9932
059_VU107-J-3	15 °C	4	3241.24 ± 139.99	2920.67 ± 125.86	0.9969
060_VU107-J-3	17 °C	4	4240.82 ± 205.39	3724.83 ± 180.07	0.9972
061_VU107-J-3	18 °C	4	4236.98 ± 242.38	3724.80 ± 212.81	0.9983
063_VU107-J-3	19 °C	4	3727.65 ± 216.48	3302.22 ± 191.47	0.9980
064_VU107-J-3	21 °C	4	2986.25 ± 139.81	2714.18 ± 126.80	0.9970
065_VU107-J-3	22 °C	4	2271.52 ± 87.01	2147.20 ± 81.98	0.9957
067_VU107-J-3	25 °C		1752.32 ± 52.07	1698.26 ± 50.20	0.9930
068_VU107-J-3	28 °C		1422.74 ± 49.38	1439.91 ± 49.73	0.9939
069_VU107-J-3	31 °C		1205.54 ± 46.24	1267.01 ± 48.23	0.9902
071_VU107-J-3	39 °C		941.53 ± 62.13	1107.12 ± 72.99	0.9985
072_VU107-J-3	69 °C		860.60 ± 81.31	1367.39 ± 129.13	0.9991
073_VU107-J-3	16 °C		320.90 ± 25.03	474.17 ± 36.81	0.9925

Results	40(a)/36(a) ± 2σ	40(r)/39(k) ± 2σ	Age ± 2σ (Ma)	MSWD
Normal Isochron<br>Overestimated Error	317.09 ± 10.88<br>± 3.43%	0.80319 ± 0.00440<br>± 0.55%	6.89 ± 0.04<br>± 0.58%<br>Full External Error ± 0.15<br>Analytical Error ± 0.04	0.29<br>94%

Statistics				
	2σ Confidence Limit	2.15	Convergence	0.000007885272
	Error Magnification	1.0000	Number of Iterations	5
	Number of Data Points	8	Calculated Line	Weighted York-2

Inverse Isochron			39(k)/40(a+r) ± 2σ	36(a)/40(a+r) ± 2σ	r.i.
055_VU107-J-3	12 °C		0.5837989 ± 0.0021384	0.00168466 ± 0.00002627	0.0286
056_VU107-J-3	13 °C	4	0.9064341 ± 0.0031481	0.00086144 ± 0.00002005	0.0165
057_VU107-J-3	14 °C	4	1.0474152 ± 0.0035373	0.00050017 ± 0.00001443	0.0155
059_VU107-J-3	15 °C	4	1.1097609 ± 0.0037617	0.00034239 ± 0.00001475	0.0100
060_VU107-J-3	17 °C	4	1.1385251 ± 0.0041286	0.00026847 ± 0.00001298	0.0128
061_VU107-J-3	18 °C	4	1.1375056 ± 0.0037739	0.00026847 ± 0.00001534	0.0072
063_VU107-J-3	19 °C	4	1.1288318 ± 0.0041909	0.00030283 ± 0.00001756	0.0074
064_VU107-J-3	21 °C	4	1.1002377 ± 0.0039924	0.00036844 ± 0.00001721	0.0111
065_VU107-J-3	22 °C	4	1.0578975 ± 0.0037466	0.00046572 ± 0.00001778	0.0110
067_VU107-J-3	25 °C		1.0318376 ± 0.0036347	0.00058884 ± 0.00001741	0.0148
068_VU107-J-3	28 °C		0.9880785 ± 0.0037862	0.00069449 ± 0.00002399	0.0110
069_VU107-J-3	31 °C		0.9514795 ± 0.0051007	0.00078926 ± 0.00003004	0.0165
071_VU107-J-3	39 °C		0.8504364 ± 0.0030449	0.00090325 ± 0.00005955	0.0097
072_VU107-J-3	69 °C		0.6293749 ± 0.0024727	0.00073132 ± 0.00006906	0.0103
073_VU107-J-3	16 °C		0.6767715 ± 0.0064337	0.00210895 ± 0.00016371	0.0214

Results	40(a)/36(a) ± 2σ	40(r)/39(k) ± 2σ	Age ± 2σ (Ma)	MSWD
Inverse Isochron<br>Overestimated Error	316.22 ± 10.88<br>± 3.44%	0.80360 ± 0.00439<br>± 0.55%	6.89 ± 0.04<br>± 0.58%<br>Full External Error ± 0.15<br>Analytical Error ± 0.04	0.28<br>95%

Statistics				
	2σ Confidence Limit	2.15	Convergence	0.0006344581
	Error Magnification	1.0000	Number of Iterations	2
	Number of Data Points	8	Calculated Line	Weighted York-2
	Spreading Factor	18.7%		

Relative Abundances			36Ar [fA]	%1σ	37Ar [fA]	%1σ	38Ar [fA]	%1σ	39Ar [fA]	%1σ	40Ar [fA]	%1σ	40(r)/39(k) ± 2σ	Age ± 2σ (Ma)	40Ar(r) (%)	39Ar(k) (%)	K/Ca ± 2σ
055_VU107-J-3	12 °C		0.0795079	0.731	16.8742	0.897	0.3150490	0.338	26.02011	0.172	44.57325	0.064	0.85137 ± 0.01399	7.30 ± 0.12	49.68	3.25	0.663 ± 0.012
056_VU107-J-3	13 °C	4	0.0616396	0.948	39.8087	0.825	0.6350693	0.265	53.82727	0.164	59.40025	0.057	0.81948 ± 0.00726	7.03 ± 0.06	74.22	6.73	0.581 ± 0.010
057_VU107-J-3	14 °C	4	0.0526671	0.897	66.4062	0.824	0.8653435	0.234	73.62356	0.157	70.31132	0.061	0.81216 ± 0.00499	6.96 ± 0.04	84.99	9.20	0.476 ± 0.008
059_VU107-J-3	15 °C	4	0.0537613	0.958	100.8101	0.820	1.0317613	0.237	88.05918	0.158	79.36422	0.060	0.80898 ± 0.00485	6.94 ± 0.04	89.69	11.00	0.375 ± 0.006
060_VU107-J-3	17 °C	4	0.0557160	0.747	126.2571	0.815	1.1203767	0.265	95.01200	0.165	83.45883	0.074	0.80793 ± 0.00452	6.93 ± 0.04	91.89	11.87	0.323 ± 0.005
061_VU107-J-3	18 °C	4	0.0507272	0.888	118.6930	0.805	0.9759515	0.251	82.24460	0.155	72.30303	0.058	0.80865 ± 0.00486	6.93 ± 0.04	91.89	10.27	0.298 ± 0.005
063_VU107-J-3	19 °C	4	0.0465597	0.996	105.1487	0.809	0.8375943	0.264	70.15225	0.174	62.14346	0.063	0.80578 ± 0.00555	6.91 ± 0.05	90.87	8.76	0.287 ± 0.005
064_VU107-J-3	21 °C	4	0.0487053	0.938	98.4922	0.812	0.8161564	0.230	67.86395	0.168	61.67923	0.068	0.80892 ± 0.00555	6.93 ± 0.05	88.92	8.48	0.296 ± 0.005
065_VU107-J-3	22 °C	4	0.0456084	0.854	83.1993	0.816	0.6563290	0.273	53.76175	0.166	50.81269	0.061	0.81383 ± 0.00582	6.98 ± 0.05	86.02	6.71	0.278 ± 0.005
067_VU107-J-3	25 °C		0.0491988	0.763	76.0654	0.822	0.6370758	0.265	51.06438	0.165	49.48304	0.062	0.79877 ± 0.00582	6.85 ± 0.05	82.35	6.38	0.288 ± 0.005
068_VU107-J-3	28 °C		0.0636386	0.734	116.6208	0.824	0.5985657	0.293	46.80052	0.182	47.32593	0.060	0.80222 ± 0.00792	6.88 ± 0.07	79.20	5.84	0.172 ± 0.003
069_VU107-J-3	31 °C		0.0623789	0.598	133.0965	0.797	0.4326222	0.249	32.91492	0.251	34.52750	0.092	0.80334 ± 0.01044	6.89 ± 0.09	76.37	4.10	0.106 ± 0.002
071_VU107-J-3	39 °C		0.0923304	0.780	238.2541	0.810	0.3716381	0.298	27.85874	0.161	32.59345	0.075	0.85877 ± 0.02117	7.36 ± 0.18	72.98	3.46	0.050 ± 0.001
072_VU107-J-3	69 °C		0.1419985	0.655	420.4011	0.792	0.3534883	0.304	26.96347	0.168	42.41506	0.097	1.24196 ± 0.03318	10.64 ± 0.28	78.12	3.34	0.027 ± 0.000
073_VU107-J-3	16 °C		0.0478822	1.034	122.9531	0.808	0.0742673	0.511	5.03037	0.424	7.31488	0.199	0.54723 ± 0.07254	4.69 ± 0.62	37.01	0.62	0.017 ± 0.000
		Σ	0.9523200	0.216	1863.0806	0.258	9.7212885	0.073	801.19707	0.048	797.70616	0.018					

Information on Analysis and Constants Used in Calculations

Sample = 055_VU107-J-3	Age Equations = Min et al. (2000)
Material = groundmass	Negative Intensities = Allowed
Location = MW14 DL3-5	Decay Constant 40K = 5.460 ± 0.053 E-10 1/a
Analyst = Klaudia Kuiper	Decay Constant 39Ar = 2.940 ± 0.016 E-07 1/h
Project = VU107	Decay Constant 37Ar = 8.230 ± 0.012 E-04 1/h
Mass Discrimination Law = LIN	Decay Constant 36Cl = 2.257 ± 0.015 E-06 1/a
Irradiation = VU107	Decay Activity 40K(EC,β⁺) = 3.310 ± 0.030 1/gs
J = 0.00468970 ± 0.00000469	Decay Activity 40K(β⁻) = 27.890 ± 0.150 1/gs
FCs = 28.201 ± 0.023 Ma	Atmospheric Ratio 40/36(a) = 298.56 ± 0.31
IGSN = Undefined	Atmospheric Ratio 38/36(a) = 0.1885 ± 0.0003
Preferred Age = Undefined	Production Ratio 39/37(ca) = 0.000673 ± 0.000004
Classification = Undefined	Production Ratio 36/37(ca) = 0.000264 ± 0.000002
Experiment Type = Undefined	Production Ratio 40/39(k) = 0.000860 ± 0.000070
Extraction Method = Undefined	Production Ratio 38/39(k) = 0.012110 ± 0.000030
Heating = 720 sec	Production Ratio 36/38(cl) = 262.80 ± 1.71
Isolation = 18.00 min	Scaling Ratio K/Ca = 0.430
Instrument = HELIX	Abundance Ratio 40K/K = 1.1700 ± 0.0100 E-04
Lithology = Undefined	Atomic Weight K = 39.0983 ± 0.0001 g
Lat-Lon = Undefined - Undefined	
Feature = Undefined	

Results

Results	40(a)/36(a) ± 2σ	40(r)/39(k) ± 2σ	Age ± 2σ (Ma)	MSWD	39Ar(k) (%,n)	K/Ca ± 2σ
**Age Plateau**		0.81002 ± 0.00255 ± 0.32%	6.94 ± 0.03 ± 0.37%	1.87 7%	73.01 8	0.325 ± 0.054
			Full External Error ± 0.15	2.07	2σ Confidence Limit	
			Analytical Error ± 0.02	1.3685	Error Magnification	
**Total Fusion Age**		0.82451 ± 0.00214 ± 0.26%	7.07 ± 0.02 ± 0.33%		15	0.185 ± 0.001
			Full External Error ± 0.15			
			Analytical Error ± 0.02			
**Normal Isochron** Overestimated Error	317.09 ± 10.88 ± 3.43%	0.80319 ± 0.00440 ± 0.55%	6.89 ± 0.04 ± 0.58%	0.29 94%	73.01 8	
			Full External Error ± 0.15	2.15	2σ Confidence Limit	
			Analytical Error ± 0.04	1.0000	Error Magnification	
				5	Number of Iterations	
				0.0000078853	Convergence	
**Inverse Isochron** Overestimated Error	316.22 ± 10.88 ± 3.44%	0.80360 ± 0.00439 ± 0.55%	6.89 ± 0.04 ± 0.58%	0.28 95%	73.01 8	
			Full External Error ± 0.15	2.15	2σ Confidence Limit	
			Analytical Error ± 0.04	1.0000	Error Magnification	
				2	Number of Iterations	
				0.0006344581	Convergence	
				19%	Spreading Factor	

Degassing Patterns			36Ar(a) [fA]	%1σ	36Ar(c) [fA]	%1σ	36Ar(ca) [fA]	%1σ	36Ar(cl) [fA]	%1σ	37Ar(ca) [fA]	%1σ	38Ar(a) [fA]	%1σ	38Ar(c) [fA]	%1σ
055_VU107-J-3	12 °C		0.0750532	0.78	0.0000000	0.00	0.0044548	1.10	0.0000000	0.00	16.8742	0.90	0.0141475	0.79	0.0000000	0.00
056_VU107-J-3	13 °C	4	0.0511301	1.16	0.0000000	0.00	0.0105095	1.04	0.0000000	0.00	39.8087	0.82	0.0096380	1.17	0.0000000	0.00
057_VU107-J-3	14 °C	4	0.0351359	1.44	0.0000000	0.00	0.0175312	1.04	0.0000000	0.00	66.4062	0.82	0.0066231	1.45	0.0000000	0.00
059_VU107-J-3	15 °C	4	0.0271474	2.15	0.0000000	0.00	0.0266139	1.04	0.0000000	0.00	100.8101	0.82	0.0051173	2.16	0.0000000	0.00
060_VU107-J-3	17 °C	4	0.0223841	2.42	0.0000000	0.00	0.0333319	1.04	0.0000000	0.00	126.2571	0.81	0.0042194	2.42	0.0000000	0.00
061_VU107-J-3	18 °C	4	0.0193923	2.86	0.0000000	0.00	0.0313350	1.03	0.0000000	0.00	118.6930	0.81	0.0036554	2.86	0.0000000	0.00
063_VU107-J-3	19 °C	4	0.0188004	2.90	0.0000000	0.00	0.0277593	1.03	0.0000000	0.00	105.1487	0.81	0.0035439	2.90	0.0000000	0.00
064_VU107-J-3	21 °C	4	0.0227033	2.33	0.0000000	0.00	0.0260020	1.03	0.0000000	0.00	98.4922	0.81	0.0042796	2.34	0.0000000	0.00
065_VU107-J-3	22 °C	4	0.0236431	1.91	0.0000000	0.00	0.0219646	1.04	0.0000006	202.77	83.1993	0.82	0.0044567	1.91	0.0000000	0.00
067_VU107-J-3	25 °C		0.0291117	1.48	0.0000000	0.00	0.0200813	1.04	0.0000058	20.84	76.0654	0.82	0.0054876	1.49	0.0000000	0.00
068_VU107-J-3	28 °C		0.0328395	1.73	0.0000000	0.00	0.0307879	1.04	0.0000112	10.59	116.6208	0.82	0.0061902	1.73	0.0000000	0.00
069_VU107-J-3	31 °C		0.0272288	1.90	0.0000000	0.00	0.0351375	1.02	0.0000127	6.76	133.0965	0.80	0.0051326	1.91	0.0000000	0.00
071_VU107-J-3	39 °C		0.0294184	3.30	0.0000000	0.00	0.0628991	1.03	0.0000130	5.63	238.2541	0.81	0.0055454	3.30	0.0000000	0.00
072_VU107-J-3	69 °C		0.0310022	4.72	0.0000000	0.00	0.1109859	1.02	0.0000104	6.84	420.4011	0.79	0.0058439	4.72	0.0000000	0.00
073_VU107-J-3	16 °C		0.0154178	3.88	0.0000000	0.00	0.0324596	1.03	0.0000048	4.63	122.9531	0.81	0.0029062	3.88	0.0000000	0.00
		Σ	0.4604081	0.57	0.0000000	0.00	0.4918533	0.33	0.0000585	4.31	1863.0806	0.26	0.0867869	0.57	0.0000000	0.00
		Σ							0.9523200	0.32	1863.0806	0.26				

Degassing Patterns			38Ar(k) [fA]	%1σ	38Ar(ca) [fA]	%1σ	38Ar(cl) [fA]	%1σ	39Ar(k) [fA]	%1σ	39Ar(ca) [fA]	%1σ	40Ar(r) [fA]	%1σ	40Ar(a) [fA]	%1σ	40Ar(c) [fA]	%1σ	40Ar(k) [fA]	%1σ
055_VU107-J-3	12 °C		0.3149660	0.30	0.0000000	0.00	0.0000000	0.00	26.00875	0.17	0.0113563	1.05	22.14301	0.80	22.40787	0.78	0.0000000	0.00	0.0223675	8.10
056_VU107-J-3	13 °C	4	0.6515238	0.30	0.0000000	0.00	0.0000000	0.00	53.80048	0.16	0.0267913	0.99	44.08857	0.41	15.26541	1.17	0.0000000	0.00	0.0462684	8.10
057_VU107-J-3	14 °C	4	0.8910402	0.30	0.0000000	0.00	0.0000000	0.00	73.57887	0.16	0.0446914	0.99	59.75788	0.26	10.49016	1.45	0.0000000	0.00	0.0632778	8.10
059_VU107-J-3	15 °C	4	1.0655750	0.30	0.0000000	0.00	0.0000000	0.00	87.99133	0.16	0.0678452	0.99	71.18342	0.25	8.10513	2.16	0.0000000	0.00	0.0756725	8.10
060_VU107-J-3	17 °C	4	1.1495663	0.30	0.0000000	0.00	0.0000000	0.00	94.92703	0.17	0.0849710	0.98	76.69419	0.23	6.68301	2.42	0.0000000	0.00	0.0816372	8.10
061_VU107-J-3	18 °C	4	0.9950147	0.29	0.0000000	0.00	0.0000000	0.00	82.16472	0.16	0.0798804	0.98	66.44261	0.26	5.78976	2.86	0.0000000	0.00	0.0706617	8.10
063_VU107-J-3	19 °C	4	0.8486868	0.30	0.0000000	0.00	0.0000000	0.00	70.08148	0.17	0.0707651	0.98	56.47013	0.30	5.61306	2.90	0.0000000	0.00	0.0602701	8.10
064_VU107-J-3	21 °C	4	0.8210297	0.30	0.0000000	0.00	0.0000000	0.00	67.79766	0.17	0.0662853	0.98	54.84262	0.30	6.77830	2.34	0.0000000	0.00	0.0583060	8.10
065_VU107-J-3	22 °C	4	0.6503768	0.30	0.0000000	0.00	0.0014956	202.78	53.70576	0.17	0.0559931	0.98	43.70762	0.32	7.05889	1.91	0.0000000	0.00	0.0461870	8.10
067_VU107-J-3	25 °C		0.6177698	0.30	0.0000000	0.00	0.0138185	20.86	51.01319	0.16	0.0511920	0.99	40.74757	0.32	8.69160	1.48	0.0000000	0.00	0.0438713	8.10
068_VU107-J-3	28 °C		0.5658038	0.31	0.0000000	0.00	0.0265717	10.63	46.72203	0.18	0.0784858	0.99	37.48120	0.46	9.80455	1.73	0.0000000	0.00	0.0401809	8.10
069_VU107-J-3	31 °C		0.3975150	0.35	0.0000000	0.00	0.0299745	6.83	32.82535	0.25	0.0895739	0.97	26.36984	0.60	8.12943	1.90	0.0000000	0.00	0.0282298	8.10
071_VU107-J-3	39 °C		0.3354276	0.30	0.0000000	0.00	0.0306651	5.70	27.69840	0.16	0.1603450	0.98	23.78648	1.22	8.78315	3.30	0.0000000	0.00	0.0238206	8.10
072_VU107-J-3	69 °C		0.3231013	0.30	0.0000000	0.00	0.0245431	6.91	26.68054	0.17	0.2829300	0.96	33.13610	1.32	9.25602	4.72	0.0000000	0.00	0.0229453	8.10
073_VU107-J-3	16 °C		0.0599157	0.50	0.0000000	0.00	0.0114454	4.72	4.94762	0.43	0.0827474	0.98	2.70750	6.61	4.60312	3.88	0.0000000	0.00	0.0042550	8.11
		Σ	9.6873124	0.09	0.0000000	0.00	0.1385138	4.33	799.94322	0.05	1.2538532	0.31	659.55875	0.12	137.45946	0.57	0.0000000	0.00	0.6879512	2.32
		Σ					9.9126132	0.10			801.19707	0.05							797.70616	0.14

Additional Parameters			40Ar/39Ar	1σ	37Ar/39Ar	1σ	36Ar/39Ar	1σ	Time (days)	37Ar (decay)	39Ar (decay)	40Ar (moles)
055_VU107-J-3	12 °C		1.713031	0.003135	0.648505	0.005925	0.003056	0.000023	259.919	174.285718	1.00184535	4.457E-12
056_VU107-J-3	13 °C	4	1.103535	0.001914	0.739564	0.006217	0.001145	0.000011	259.940	174.359843	1.00184550	5.940E-12
057_VU107-J-3	14 °C	4	0.955011	0.001610	0.901969	0.007569	0.000715	0.000007	259.962	174.433999	1.00184566	7.031E-12
059_VU107-J-3	15 °C	4	0.901260	0.001525	1.144800	0.009562	0.000611	0.000006	260.003	174.575223	1.00184595	7.936E-12
060_VU107-J-3	17 °C	4	0.878403	0.001590	1.328854	0.011044	0.000586	0.000004	260.024	174.649471	1.00184610	8.346E-12
061_VU107-J-3	18 °C	4	0.879122	0.001455	1.443171	0.011836	0.000617	0.000006	260.046	174.723751	1.00184625	7.230E-12
063_VU107-J-3	19 °C	4	0.885837	0.001641	1.498865	0.012402	0.000664	0.000007	260.088	174.867607	1.00184654	6.214E-12
064_VU107-J-3	21 °C	4	0.908866	0.001646	1.451319	0.012033	0.000718	0.000007	260.108	174.939580	1.00184669	6.168E-12
065_VU107-J-3	22 °C	4	0.945146	0.001670	1.547555	0.012888	0.000848	0.000007	260.130	175.013983	1.00184684	5.081E-12
067_VU107-J-3	25 °C		0.969032	0.001704	1.489597	0.012488	0.000963	0.000008	260.172	175.158079	1.00184714	4.948E-12
068_VU107-J-3	28 °C		1.011227	0.001933	2.491870	0.021028	0.001360	0.000010	260.192	175.230171	1.00184729	4.733E-12
069_VU107-J-3	31 °C		1.048992	0.002804	4.043652	0.033783	0.001895	0.000012	260.214	175.304698	1.00184744	3.453E-12
071_VU107-J-3	39 °C		1.169954	0.002082	8.552219	0.070603	0.003314	0.000026	260.256	175.449033	1.00184773	3.259E-12
072_VU107-J-3	69 °C		1.573057	0.003060	15.591510	0.126315	0.005266	0.000036	260.277	175.523653	1.00184788	4.242E-12
073_VU107-J-3	16 °C		1.454143	0.006813	24.442165	0.223157	0.009519	0.000106	260.298	175.595896	1.00184803	7.315E-13

Procedure Blanks		36Ar [fA]	1σ	37Ar [fA]	1σ	38Ar [fA]	1σ	39Ar [fA]	1σ	40Ar [fA]	1σ
055_VU107-J-3	12 °C	0.0091895	0.0001282	0.0129220	0.0001421	0.0055383	0.0000787	0.2270783	0.0175544	2.8195341	0.0031127
056_VU107-J-3	13 °C	0.0091895	0.0001282	0.0129220	0.0001421	0.0055383	0.0000787	0.2270783	0.0175544	2.8195341	0.0031127
057_VU107-J-3	14 °C	0.0091895	0.0001282	0.0129220	0.0001421	0.0055383	0.0000787	0.2270783	0.0175544	2.8195341	0.0031127
059_VU107-J-3	15 °C	0.0095636	0.0000737	0.0131462	0.0000931	0.0060505	0.0001372	0.2439519	0.0100183	2.8357870	0.0052424
060_VU107-J-3	17 °C	0.0095636	0.0000737	0.0131462	0.0000931	0.0060505	0.0001372	0.2439519	0.0100183	2.8357870	0.0052424
061_VU107-J-3	18 °C	0.0095636	0.0000737	0.0131462	0.0000931	0.0060505	0.0001372	0.2439519	0.0100183	2.8357870	0.0052424
063_VU107-J-3	19 °C	0.0097299	0.0001392	0.0131804	0.0000864	0.0062892	0.0000898	0.2436652	0.0276875	2.8392322	0.0047241
064_VU107-J-3	21 °C	0.0097299	0.0001392	0.0131804	0.0000864	0.0062892	0.0000898	0.2436652	0.0276875	2.8392322	0.0047241
065_VU107-J-3	22 °C	0.0097299	0.0001392	0.0131804	0.0000864	0.0062892	0.0000898	0.2436652	0.0276875	2.8392322	0.0047241
067_VU107-J-3	25 °C	0.0092697	0.0001025	0.0134981	0.0001013	0.0062782	0.0000810	0.1967272	0.0265422	2.7642648	0.0046521
068_VU107-J-3	28 °C	0.0092697	0.0001025	0.0134981	0.0001013	0.0062782	0.0000810	0.1967272	0.0265422	2.7642648	0.0046521
069_VU107-J-3	31 °C	0.0092697	0.0001025	0.0134981	0.0001013	0.0062782	0.0000810	0.1967272	0.0265422	2.7642648	0.0046521
071_VU107-J-3	39 °C	0.0086423	0.0001114	0.0136353	0.0000977	0.0057583	0.0001128	0.2379897	0.0049113	2.7413966	0.0054199
072_VU107-J-3	69 °C	0.0086423	0.0001114	0.0136353	0.0000977	0.0057583	0.0001128	0.2379897	0.0049113	2.7413966	0.0054199
073_VU107-J-3	16 °C	0.0086423	0.0001114	0.0136353	0.0000977	0.0057583	0.0001128	0.2379897	0.0049113	2.7413966	0.0054199

Intercept Values		36Ar [fA]	1σ	r2		37Ar [fA]	1σ	r2		38Ar [fA]	1σ	r2		39Ar [fA]	1σ	r2		40Ar [fA]	1σ	r2	
055_VU107-J-3	12 °C	0.0834370	0.0004249	0.8100	LIN 15 of 15	0.1049	0.0004	0.9600	LIN 15 of 15	0.3101534	0.0008169	0.9800	LIN 15 of 15	25.76994	0.03102	1.0000	EXP 15 of 15	47.39278	0.02822	1.0000	EXP 15 of 15
056_VU107-J-3	13 °C	0.0667509	0.0004716	0.6000	LIN 15 of 15	0.2299	0.0005	0.9800	LIN 15 of 15	0.6195752	0.0010364	0.9900	LIN 15 of 15	53.06706	0.06625	1.0000	EXP 15 of 15	62.21979	0.03396	1.0000	EXP 15 of 15
057_VU107-J-3	14 °C	0.0583720	0.0003678	0.5600	LIN 15 of 15	0.3747	0.0009	0.9800	LIN 15 of 15	0.8422232	0.0009587	1.0000	LIN 15 of 15	72.50025	0.08595	1.0000	EXP 15 of 15	73.13086	0.04281	1.0000	EXP 15 of 15
059_VU107-J-3	15 °C	0.0597679	0.0004257	0.3800	LIN 15 of 15	0.5620	0.0013	0.9800	LIN 15 of 15	1.0036417	0.0012123	1.0000	LIN 15 of 15	86.68794	0.10559	1.0000	EXP 15 of 15	82.20001	0.04734	1.0000	EXP 15 of 15
060_VU107-J-3	17 °C	0.0615933	0.0003126	0.3700	LIN 15 of 15	0.7002	0.0015	0.9900	LIN 15 of 15	1.0893223	0.0018390	0.9900	LIN 15 of 15	93.51321	0.12213	1.0000	EXP 15 of 15	86.29462	0.06183	1.0000	EXP 15 of 15
061_VU107-J-3	18 °C	0.0569346	0.0003630	0.4800	LIN 15 of 15	0.6588	0.0012	0.9900	LIN 15 of 15	0.9496802	0.0013800	0.9900	LIN 15 of 15	80.97998	0.09535	1.0000	EXP 15 of 15	75.13881	0.04144	1.0000	EXP 15 of 15
063_VU107-J-3	19 °C	0.0532091	0.0003669	0.5700	LIN 15 of 15	0.5847	0.0011	0.9900	LIN 15 of 15	0.8161439	0.0013631	0.9900	LIN 15 of 15	69.10913	0.09452	1.0000	EXP 15 of 15	64.98270	0.03856	1.0000	EXP 15 of 15
064_VU107-J-3	21 °C	0.0552127	0.0003550	0.1700	LIN 15 of 15	0.5483	0.0011	0.9800	LIN 15 of 15	0.7954160	0.0008506	1.0000	LIN 15 of 15	66.86280	0.08546	1.0000	EXP 15 of 15	64.51846	0.04180	1.0000	EXP 15 of 15
065_VU107-J-3	22 °C	0.0523207	0.0002841	0.3600	LIN 15 of 15	0.4650	0.0010	0.9900	LIN 15 of 15	0.6408818	0.0011549	0.9900	LIN 15 of 15	53.01927	0.06431	1.0000	EXP 15 of 15	53.65192	0.03043	1.0000	EXP 15 of 15
067_VU107-J-3	25 °C	0.0552134	0.0002741	0.6000	LIN 15 of 15	0.4262	0.0010	0.9800	LIN 15 of 15	0.6222552	0.0010457	0.9900	LIN 15 of 15	50.32442	0.06006	1.0000	EXP 15 of 15	52.24731	0.03009	1.0000	EXP 15 of 15
068_VU107-J-3	28 °C	0.0686978	0.0003422	0.8000	LIN 15 of 15	0.6460	0.0016	0.9800	LIN 15 of 15	0.5850205	0.0012177	0.9900	LIN 15 of 15	46.13876	0.06439	1.0000	EXP 15 of 15	50.09019	0.02800	1.0000	EXP 15 of 15
069_VU107-J-3	31 °C	0.0675215	0.0002250	0.9500	LIN 15 of 15	0.7351	0.0010	1.0000	LIN 15 of 15	0.4245727	0.0005942	0.9900	LIN 15 of 15	32.50787	0.06956	0.9900	EXP 15 of 15	37.29177	0.03125	1.0000	EXP 15 of 15
071_VU107-J-3	39 °C	0.0948639	0.0005552	0.8500	LIN 14 of 15	1.3042	0.0025	0.9900	LIN 14 of 15	0.3650884	0.0007750	0.9900	LIN 14 of 15	27.58570	0.03429	1.0000	EXP 14 of 15	35.33485	0.02392	1.0000	EXP 14 of 15
072_VU107-J-3	69 °C	0.1412458	0.0006555	0.8800	LIN 15 of 15	2.2899	0.0023	1.0000	LIN 15 of 15	0.3475397	0.0007651	0.9800	LIN 15 of 15	26.70684	0.03556	1.0000	EXP 15 of 15	45.15646	0.04098	1.0000	EXP 15 of 15
073_VU107-J-3	16 °C	0.0533565	0.0004074	0.8700	LIN 13 of 15	0.6791	0.0013	0.9900	LIN 15 of 15	0.0775660	0.0003170	0.9300	LIN 15 of 15	5.17608	0.01977	0.9600	EXP 15 of 15	10.05627	0.01348	0.8900	EXP 13 of 15

Sample Parameters		Sample	Material	Location	Analyst	Temp	Standard (in Ma)	%1σ	J	%1σ	MDF	%1σ	Volume Ratio	Sensitivity (mol/volt)	Day	Month	Year	Hour	Min	Resist	Irradiation	Project	Experiment	Nmb	Standard Name
055_VU107-J-3	12 °C	055_VU107-J-3	groundmass	MW14 DL3-5	Klaudia Kuiper	11.7	28.201	0.08	0.0046897	0.1	0.983438	0.1	1	1E-13	18	NOV	2016	22	2	1	VU107	VU107	VU107-J-3	01	FCs
056_VU107-J-3	13 °C	056_VU107-J-3	groundmass	MW14 DL3-5	Klaudia Kuiper	13.1	28.201	0.08	0.0046897	0.1	0.983438	0.1	1	1E-13	18	NOV	2016	22	33	1	VU107	VU107	VU107-J-3	01	FCs
057_VU107-J-3	14 °C	057_VU107-J-3	groundmass	MW14 DL3-5	Klaudia Kuiper	14.3	28.201	0.08	0.0046897	0.1	0.983438	0.1	1	1E-13	18	NOV	2016	23	4	1	VU107	VU107	VU107-J-3	01	FCs
059_VU107-J-3	15 °C	059_VU107-J-3	groundmass	MW14 DL3-5	Klaudia Kuiper	15.3	28.201	0.08	0.0046897	0.1	0.983438	0.1	1	1E-13	19	NOV	2016	0	3	1	VU107	VU107	VU107-J-3	01	FCs
060_VU107-J-3	17 °C	060_VU107-J-3	groundmass	MW14 DL3-5	Klaudia Kuiper	16.5	28.201	0.08	0.0046897	0.1	0.983438	0.1	1	1E-13	19	NOV	2016	0	34	1	VU107	VU107	VU107-J-3	01	FCs
061_VU107-J-3	18 °C	061_VU107-J-3	groundmass	MW14 DL3-5	Klaudia Kuiper	17.6	28.201	0.08	0.0046897	0.1	0.983438	0.1	1	1E-13	19	NOV	2016	1	5	1	VU107	VU107	VU107-J-3	01	FCs
063_VU107-J-3	19 °C	063_VU107-J-3	groundmass	MW14 DL3-5	Klaudia Kuiper	18.9	28.201	0.08	0.0046897	0.1	0.983438	0.1	1	1E-13	19	NOV	2016	2	5	1	VU107	VU107	VU107-J-3	01	FCs
064_VU107-J-3	21 °C	064_VU107-J-3	groundmass	MW14 DL3-5	Klaudia Kuiper	20.5	28.201	0.08	0.0046897	0.1	0.983438	0.1	1	1E-13	19	NOV	2016	2	35	1	VU107	VU107	VU107-J-3	01	FCs
065_VU107-J-3	22 °C	065_VU107-J-3	groundmass	MW14 DL3-5	Klaudia Kuiper	22.4	28.201	0.08	0.0046897	0.1	0.983438	0.1	1	1E-13	19	NOV	2016	3	6	1	VU107	VU107	VU107-J-3	01	FCs
067_VU107-J-3	25 °C	067_VU107-J-3	groundmass	MW14 DL3-5	Klaudia Kuiper	24.6	28.201	0.08	0.0046897	0.1	0.983438	0.1	1	1E-13	19	NOV	2016	4	6	1	VU107	VU107	VU107-J-3	01	FCs
068_VU107-J-3	28 °C	068_VU107-J-3	groundmass	MW14 DL3-5	Klaudia Kuiper	27.6	28.201	0.08	0.0046897	0.1	0.983438	0.1	1	1E-13	19	NOV	2016	4	36	1	VU107	VU107	VU107-J-3	01	FCs
069_VU107-J-3	31 °C	069_VU107-J-3	groundmass	MW14 DL3-5	Klaudia Kuiper	31.2	28.201	0.08	0.0046897	0.1	0.983438	0.1	1	1E-13	19	NOV	2016	5	7	1	VU107	VU107	VU107-J-3	01	FCs
071_VU107-J-3	39 °C	071_VU107-J-3	groundmass	MW14 DL3-5	Klaudia Kuiper	38.7	28.201	0.08	0.0046897	0.1	0.983438	0.1	1	1E-13	19	NOV	2016	6	7	1	VU107	VU107	VU107-J-3	01	FCs
072_VU107-J-3	69 °C	072_VU107-J-3	groundmass	MW14 DL3-5	Klaudia Kuiper	68.6	28.201	0.08	0.0046897	0.1	0.983438	0.1	1	1E-13	19	NOV	2016	6	38	1	VU107	VU107	VU107-J-3	01	FCs
073_VU107-J-3	16 °C	073_VU107-J-3	groundmass	MW14 DL3-5	Klaudia Kuiper	16	28.201	0.08	0.0046897	0.1	0.983438	0.1	1	1E-13	19	NOV	2016	7	8	1	VU107	VU107	VU107-J-3	01	FCs

Irradiation Constants		40/36(a)	%1σ	40/36(c)	%1σ	38/36(a)	%1σ	38/36(c)	%1σ	39/37(ca)	%1σ	38/37(ca)	%1σ	36/37(ca)	%1σ	40/39(k)	%1σ	38/39(k)	%1σ	36/38(cl)	%1σ	K/Ca	%1σ	K/Cl	%1σ	Ca/Cl	%1σ
055_VU107-J-3	12 °C	298.56	0.104	0.018	35	0.1885	0.159	1.493	3	0.000673	0.55	0	0	0.000264	0.64	0.00086	8.1	0.01211	0.25	0	0	0.43	0	0	0	0	0
056_VU107-J-3	13 °C	298.56	0.104	0.018	35	0.1885	0.159	1.493	3	0.000673	0.55	0	0	0.000264	0.64	0.00086	8.1	0.01211	0.25	0	0	0.43	0	0	0	0	0
057_VU107-J-3	14 °C	298.56	0.104	0.018	35	0.1885	0.159	1.493	3	0.000673	0.55	0	0	0.000264	0.64	0.00086	8.1	0.01211	0.25	0	0	0.43	0	0	0	0	0
059_VU107-J-3	15 °C	298.56	0.104	0.018	35	0.1885	0.159	1.493	3	0.000673	0.55	0	0	0.000264	0.64	0.00086	8.1	0.01211	0.25	0	0	0.43	0	0	0	0	0
060_VU107-J-3	17 °C	298.56	0.104	0.018	35	0.1885	0.159	1.493	3	0.000673	0.55	0	0	0.000264	0.64	0.00086	8.1	0.01211	0.25	0	0	0.43	0	0	0	0	0
061_VU107-J-3	18 °C	298.56	0.104	0.018	35	0.1885	0.159	1.493	3	0.000673	0.55	0	0	0.000264	0.64	0.00086	8.1	0.01211	0.25	0	0	0.43	0	0	0	0	0
063_VU107-J-3	19 °C	298.56	0.104	0.018	35	0.1885	0.159	1.493	3	0.000673	0.55	0	0	0.000264	0.64	0.00086	8.1	0.01211	0.25	0	0	0.43	0	0	0	0	0
064_VU107-J-3	21 °C	298.56	0.104	0.018	35	0.1885	0.159	1.493	3	0.000673	0.55	0	0	0.000264	0.64	0.00086	8.1	0.01211	0.25	0	0	0.43	0	0	0	0	0
065_VU107-J-3	22 °C	298.56	0.104	0.018	35	0.1885	0.159	1.493	3	0.000673	0.55	0	0	0.000264	0.64	0.00086	8.1	0.01211	0.25	0	0	0.43	0	0	0	0	0
067_VU107-J-3	25 °C	298.56	0.104	0.018	35	0.1885	0.159	1.493	3	0.000673	0.55	0	0	0.000264	0.64	0.00086	8.1	0.01211	0.25	0	0	0.43	0	0	0	0	0
068_VU107-J-3	28 °C	298.56	0.104	0.018	35	0.1885	0.159	1.493	3	0.000673	0.55	0	0	0.000264	0.64	0.00086	8.1	0.01211	0.25	0	0	0.43	0	0	0	0	0
069_VU107-J-3	31 °C	298.56	0.104	0.018	35	0.1885	0.159	1.493	3	0.000673	0.55	0	0	0.000264	0.64	0.00086	8.1	0.01211	0.25	0	0	0.43	0	0	0	0	0
071_VU107-J-3	39 °C	298.56	0.104	0.018	35	0.1885	0.159	1.493	3	0.000673	0.55	0	0	0.000264	0.64	0.00086	8.1	0.01211	0.25	0	0	0.43	0	0	0	0	0
072_VU107-J-3	69 °C	298.56	0.104	0.018	35	0.1885	0.159	1.493	3	0.000673	0.55	0	0	0.000264	0.64	0.00086	8.1	0.01211	0.25	0	0	0.43	0	0	0	0	0
073_VU107-J-3	16 °C	298.56	0.104	0.018	35	0.1885	0.159	1.493	3	0.000673	0.55	0	0	0.000264	0.64	0.00086	8.1	0.01211	0.25	0	0	0.43	0	0	0	0	0

Geochronology laboratory
Vrije Universiteit Amsterdam, Amsterdam, The Netherlands

VU107-J3_1_CORR_MW14 DL3-5.AGE >>> 055_VU107-J-3 >>> VU107 PROJECT

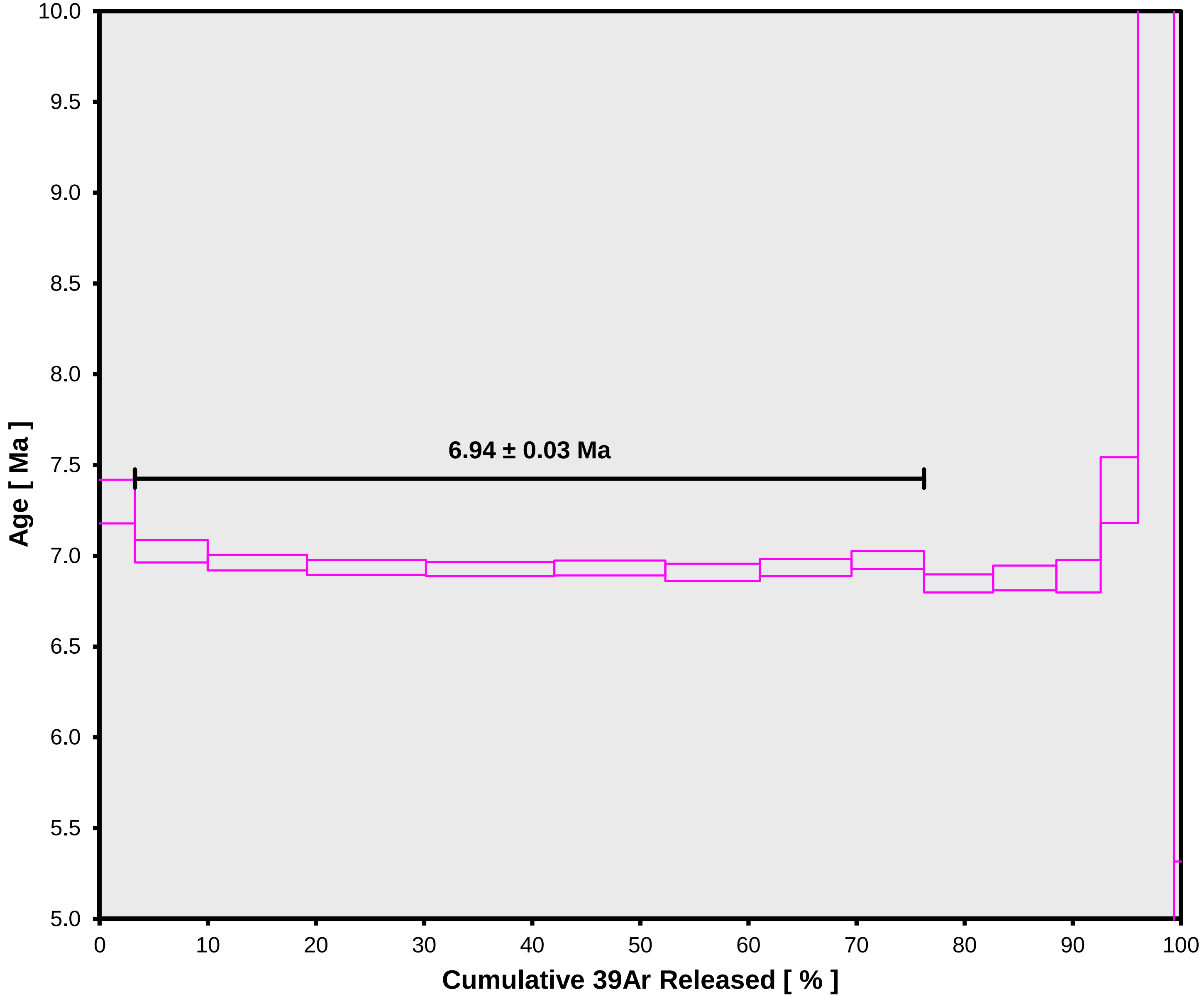

Ar-Ages in Ma

WEIGHTED PLATEAU
6.94 ± 0.03
TOTAL FUSION
7.07 ± 0.02
NORMAL ISOCHRON
6.89 ± 0.04
INVERSE ISOCHRON
6.89 ± 0.04

MSWD (PROBABILITY)
1.87 (7%)

Sample Info

groundmass
MW14 DL3-5
Klaudia Kuiper

IRR = VU107
J = 0.00468970 ± 0.00000469

RECALIBRATED AGE

VU107-J3_1_MW14 DL3-5_groundmass_full printed at 4/27/2018 (3:47 PM)
ArArCALC v2.5.2

VU107-J3_1_CORR_MW14 DL3-5.AGE >>> 055_VU107-J-3 >>> VU107 PROJECT

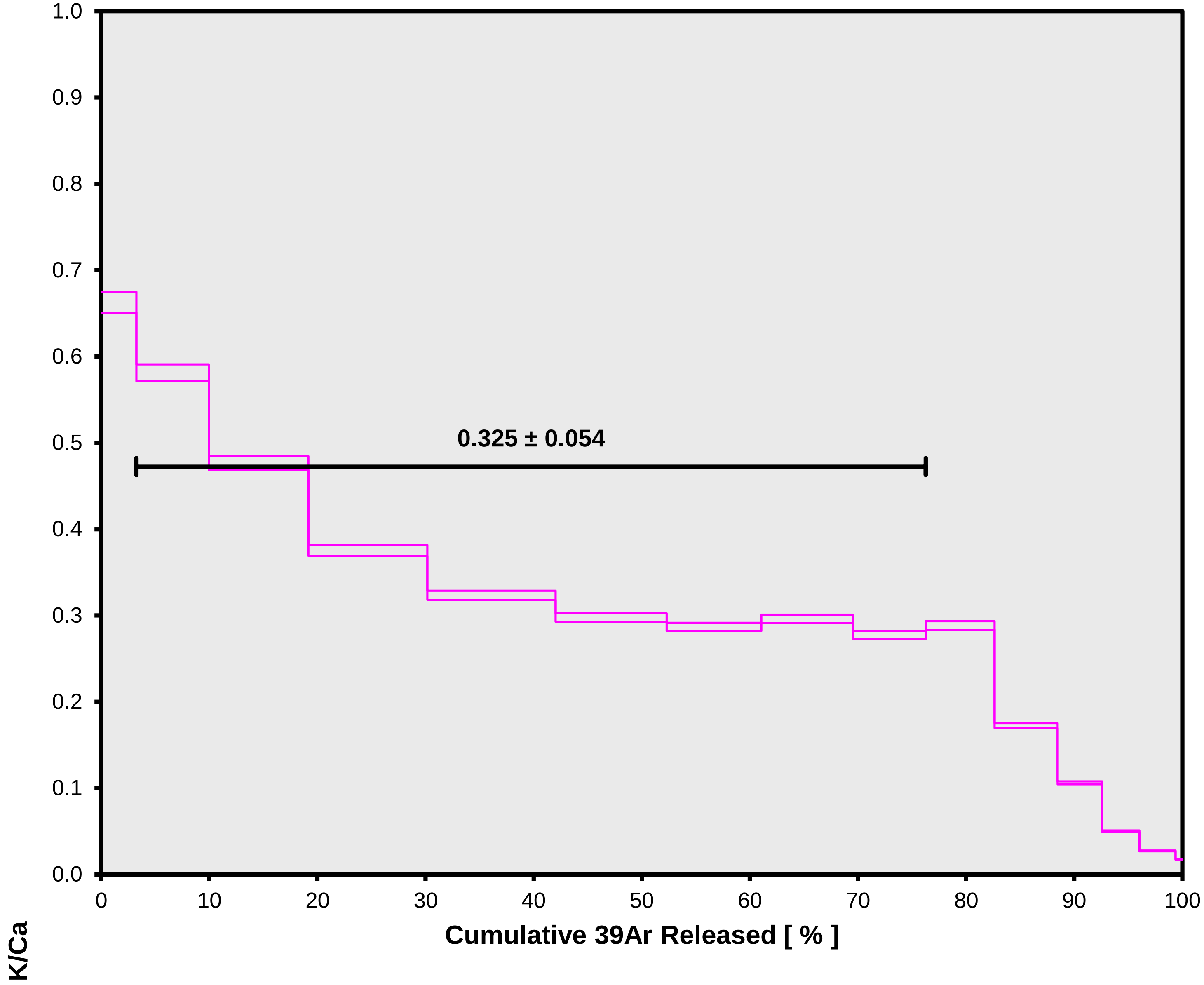

Ar-Ages in Ma

WEIGHTED PLATEAU
6.94 ± 0.03
TOTAL FUSION
7.07 ± 0.02
NORMAL ISOCHRON
6.89 ± 0.04
INVERSE ISOCHRON
6.89 ± 0.04

Sample Info

groundmass
MW14 DL3-5
Klaudia Kuiper

IRR = VU107
J = 0.00468970 ± 0.00000469

RECALIBRATED AGE

VU107-J3_1_CORR_MW14 DL3-5.AGE >>> 055_VU107-J-3 >>> VU107 PROJECT

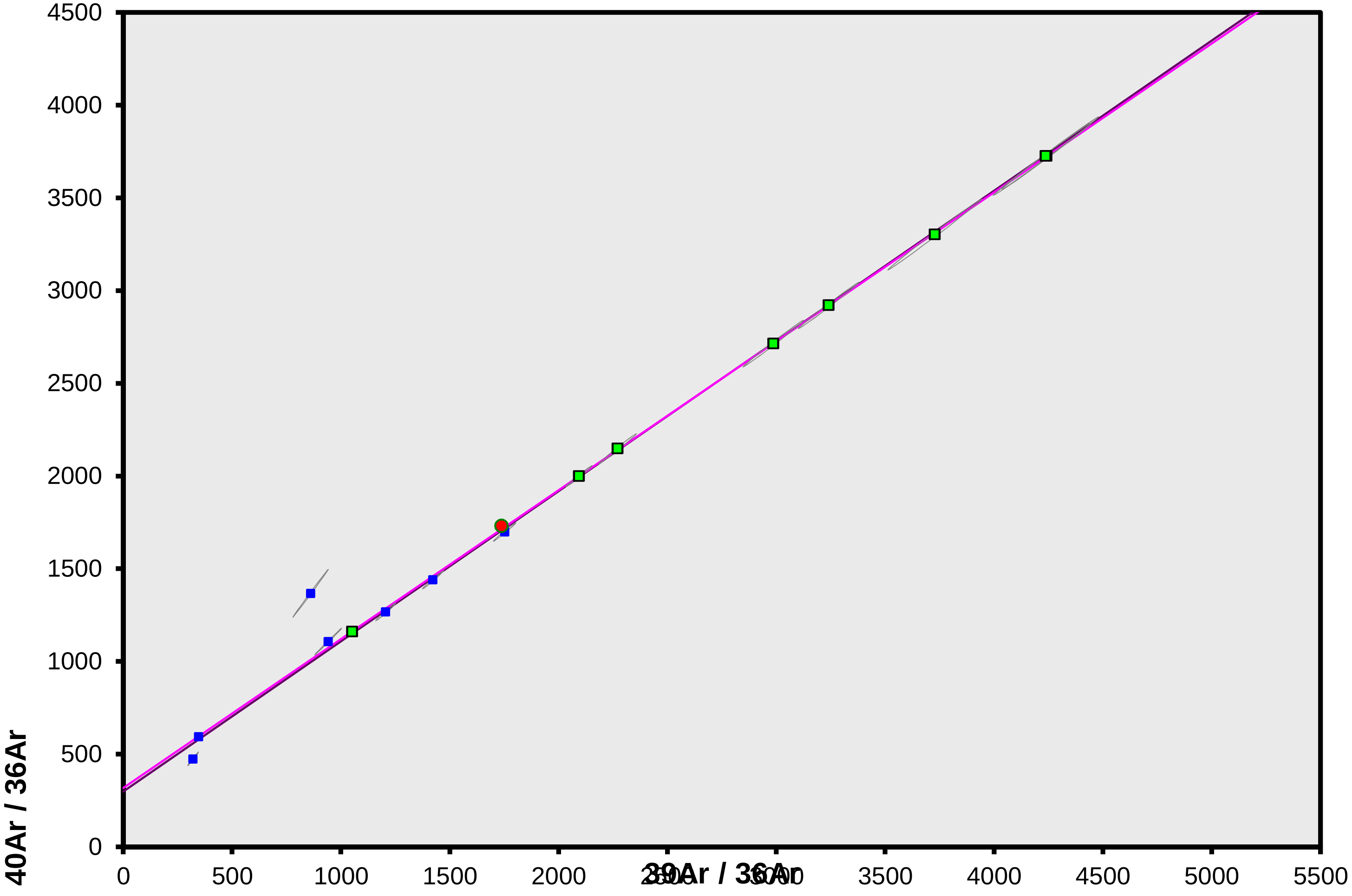

Ar-Ages in Ma

WEIGHTED PLATEAU
6.94 ± 0.03
TOTAL FUSION
7.07 ± 0.02
NORMAL ISOCHRON
6.89 ± 0.04
INVERSE ISOCHRON
6.89 ± 0.04

MSWD (PROBABILITY)
0.29 (94%)

40AR/36AR INTERCEPT
317.1 ± 10.9

Sample Info

groundmass
MW14 DL3-5
Klaudia Kuiper

IRR = VU107
J = 0.00468970 ± 0.00000469

RECALIBRATED AGE

VU107-J3_1_CORR_MW14 DL3-5.AGE >>> 055_VU107-J-3 >>> VU107 PROJECT

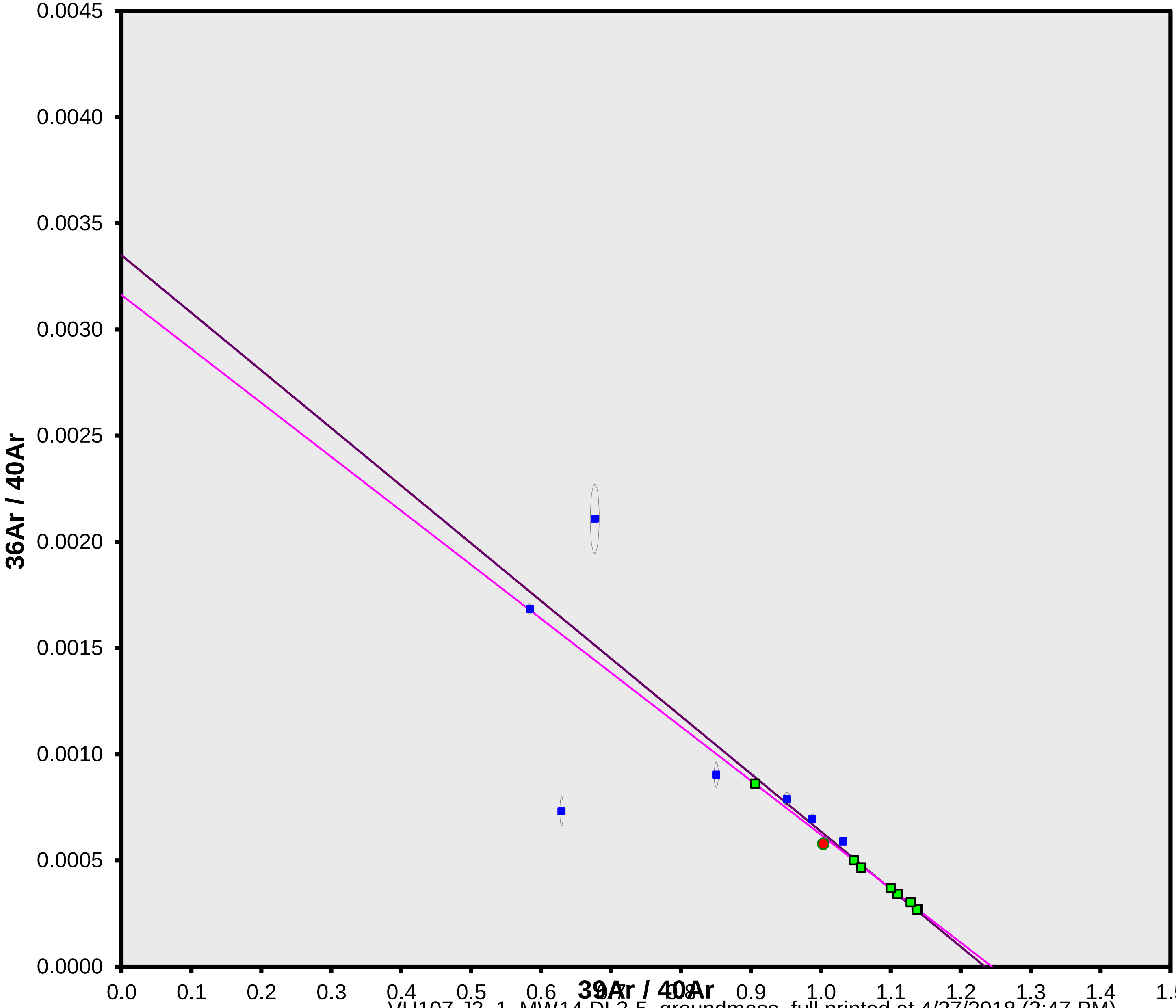

Ar-Ages in Ma

WEIGHTED PLATEAU
6.94 ± 0.03
TOTAL FUSION
7.07 ± 0.02
NORMAL ISOCHRON
6.89 ± 0.04
INVERSE ISOCHRON
6.89 ± 0.04

MSWD (PROBABILITY)
0.28 (95%)

SPREADING FACTOR
18.7%

40AR/36AR INTERCEPT
316.2 ± 10.9

Sample Info

groundmass
MW14 DL3-5
Klaudia Kuiper

IRR = VU107
J = 0.00468970 ± 0.00000469

RECALIBRATED AGE

Relative Abundances			36Ar [fA]	%1σ	37Ar [fA]	%1σ	38Ar [fA]	%1σ	39Ar [fA]	%1σ	40Ar [fA]	%1σ	40(r)/39(k) ± 2σ	Age ± 2σ (Ma)	40Ar(r) (%)	39Ar(k) (%)	K/Ca ± 2σ
17D17983	1.8 %		0.0817191	0.705	40.6703	0.826	0.606835	3.908	50.7202	0.080	146.4183	0.051	2.47130 ± 0.00841	6.98 ± 0.02	85.56	3.71	0.536 ± 0.009
17D17985	1.9 %		0.0646316	0.821	42.7496	0.813	0.683222	3.489	53.1865	0.078	145.7613	0.044	2.44227 ± 0.00751	6.89 ± 0.02	89.07	3.89	0.535 ± 0.009
17D17986	2.0 %		0.0483275	0.868	36.8168	0.920	0.543092	4.488	45.6801	0.085	122.5235	0.046	2.43051 ± 0.00735	6.86 ± 0.02	90.57	3.34	0.533 ± 0.010
17D17988	2.2 %		0.0418661	1.059	38.1756	0.883	0.558361	4.573	44.9693	0.083	118.8240	0.044	2.43162 ± 0.00757	6.86 ± 0.02	91.97	3.29	0.506 ± 0.009
17D17989	2.4 %		0.0627246	0.765	68.6237	0.610	0.894092	2.621	76.0216	0.072	198.1569	0.024	2.43147 ± 0.00533	6.86 ± 0.02	93.22	5.56	0.476 ± 0.006
17D17991	2.7 %		0.0639066	0.802	81.4622	0.559	0.946926	2.577	81.5610	0.070	211.3559	0.024	2.43627 ± 0.00527	6.88 ± 0.01	93.95	5.97	0.430 ± 0.005
17D17992	3.0 %		0.0715595	0.714	107.1128	0.495	1.127732	2.258	93.1702	0.069	240.3078	0.020	2.44082 ± 0.00489	6.89 ± 0.01	94.56	6.82	0.374 ± 0.004
17D17994	3.4 %	✓	0.0765584	0.669	132.9444	0.466	1.186873	2.086	98.2194	0.067	252.4340	0.020	2.44470 ± 0.00475	6.90 ± 0.01	95.03	7.19	0.317 ± 0.003
17D17995	3.9 %	✓	0.0935271	0.638	179.6709	0.438	1.328571	1.759	112.1744	0.067	287.8976	0.017	2.44501 ± 0.00479	6.90 ± 0.01	95.16	8.20	0.268 ± 0.002
17D17997	4.5 %	✓	0.1489508	0.507	302.6577	0.417	1.962109	1.199	164.0813	0.065	421.7298	0.012	2.44638 ± 0.00443	6.91 ± 0.01	95.06	12.00	0.233 ± 0.002
17D17998	5.2 %	✓	0.0823972	0.650	166.1651	0.447	1.059919	2.312	88.4690	0.070	228.3543	0.021	2.45306 ± 0.00527	6.92 ± 0.01	94.92	6.47	0.229 ± 0.002
17D18000	6.0 %	✓	0.0974701	0.631	163.8046	0.445	1.224939	1.958	102.0459	0.068	266.6900	0.019	2.45634 ± 0.00513	6.93 ± 0.01	93.89	7.46	0.268 ± 0.002
17D18001	6.9 %	✓	0.0746402	0.730	97.0868	0.518	1.004506	2.569	81.1317	0.071	213.5368	0.023	2.45246 ± 0.00551	6.92 ± 0.02	93.10	5.94	0.359 ± 0.004
17D18003	7.9 %	✓	0.0670332	0.777	61.4122	0.659	0.826239	2.976	68.5557	0.073	183.0515	0.027	2.44933 ± 0.00600	6.91 ± 0.02	91.68	5.02	0.480 ± 0.006
17D18004	9.0 %		0.0644105	0.856	41.5377	0.828	0.704223	3.415	55.6280	0.077	150.3430	0.037	2.41668 ± 0.00729	6.82 ± 0.02	89.37	4.07	0.576 ± 0.010
17D18006	10.3 %		0.0581275	0.821	31.7000	1.063	0.490015	4.700	41.0906	0.087	112.0649	0.046	2.36737 ± 0.00850	6.68 ± 0.02	86.76	3.01	0.557 ± 0.012
17D18007	11.6 %		0.0852551	0.665	54.1498	0.701	0.543004	4.264	42.2379	0.085	116.8025	0.046	2.26795 ± 0.00930	6.40 ± 0.03	81.94	3.09	0.335 ± 0.005
17D18008	12.5 %		0.0551902	0.855	43.0952	0.820	0.315761	7.595	22.9533	0.119	62.9437	0.070	2.17850 ± 0.01400	6.15 ± 0.04	79.34	1.68	0.229 ± 0.004
17D18010	13.4 %		0.0596918	0.728	55.9687	0.655	0.260857	8.903	18.7864	0.139	50.9796	0.087	2.00947 ± 0.01586	5.67 ± 0.04	73.90	1.37	0.144 ± 0.002
17D18011	14.6 %		0.0415734	0.949	47.4293	0.789	0.132450	17.515	10.1113	0.228	27.5758	0.159	1.88355 ± 0.02684	5.32 ± 0.08	68.85	0.74	0.091 ± 0.002
17D18012	16.0 %		0.0476716	0.894	72.6445	0.599	0.099452	23.631	7.5267	0.311	21.2276	0.208	1.71559 ± 0.03845	4.85 ± 0.11	60.43	0.55	0.044 ± 0.001
17D18014	17.6 %		0.0360721	0.989	57.5483	0.683	0.057879	41.840	3.8180	0.576	12.2909	0.353	1.62630 ± 0.06539	4.59 ± 0.18	50.00	0.28	0.028 ± 0.001
17D18015	19.3 %		0.0401029	0.948	72.9168	0.573	0.047015	49.324	2.9114	0.784	12.3209	0.356	2.16540 ± 0.09412	6.11 ± 0.27	50.30	0.21	0.017 ± 0.000
17D18017	21.0 %		0.0362705	1.041	72.7266	0.591	0.075230	30.581	2.0896	1.071	18.5573	0.233	6.64420 ± 0.19031	18.69 ± 0.53	73.06	0.15	0.012 ± 0.000
		Σ	1.5996775	0.155	2069.0696	0.125	16.679304	0.705	1367.1393	0.018	3622.1478	0.007					

Information on Analysis and Constants Used in Calculations

Project = **O-CONNOR (16-23)**
Sample = **MW14-DL2-3**
Material = **Groundmass**
Location = **Mozambique Ridge**
Region = **Indian Ocean**
Analyst = **Dan Miggins**
Irradiation = **17-OSU-01 (1B32-17)**
Position = **X: 0 | Y: 0 | Z/H: 50.93376 mm**
FCT-NM Age = **28.201 ± 0.023 Ma**
FCT-NM Reference = **Kuiper et al (2008)**
FCT-NM 40Ar/39Ar Ratio = **10.04942 ± 0.01015**
FCT-NM J-value = **0.00156401 ± 0.00000158**
Air Shot 40Ar/36Ar = **302.5270 ± 0.2844**
Air Shot MDF = **0.99418558 ± 0.00062340 (LIN)**
Experiment Type = **Incremental Heating**
Extraction Method = **Bulk Laser Heating**
Heating = **77 sec**
Isolation = **3.00 min**
Instrument = **ARGUS-VI-D**
Preferred Age = **Plateau Age**
Age Classification = **Crystallization Age**
IGSN = Undefined
Rock Class = Undefined
Lithology = Undefined
Lat-Lon = Undefined - Undefined

Age Equations = **Min et al. (2000)**
Negative Intensities = **Allowed**
Collector Calibrations = **36Ar**
Decay 40K = **5.530 ± 0.048 E-10 1/a**
Decay 39Ar = **2.940 ± 0.016 E-07 1/h**
Decay 37Ar = **8.230 ± 0.012 E-04 1/h**
Decay 36Cl = **2.257 ± 0.015 E-06 1/a**
Decay 40K(EC,β^+) = **0.580 ± 0.009 E-10 1/a**
Decay 40K(β^-) = **4.950 ± 0.043 E-10 1/a**
Atmospheric 40/36(a) = **295.50**
Atmospheric 38/36(a) = **0.1869**
Production 39/37(ca) = **0.0006756 ± 0.0000089**
Production 38/37(ca) = **0.0000718 ± 0.0000092**
Production 36/37(ca) = **0.0002663 ± 0.0000004**
Production 40/39(k) = **0.003823 ± 0.000102**
Production 38/39(k) = **0.012031 ± 0.000019**
Production 36/38(cl) = **262.80 ± 1.71**
Scaling Ratio K/Ca = **0.430**
Abundance Ratio 40K/K = **1.1700 ± 0.0100 E-04**
Atomic Weight K = **39.0983 ± 0.0001 g**

Results

Results	40(a)/36(a) ± 2σ	40(r)/39(k) ± 2σ	Age ± 2σ (Ma)	MSWD	39Ar(k) (%,n)	K/Ca ± 2σ
**Age Plateau**		2.44919 ± 0.00345	**6.91 ± 0.02**	3.26	52.27	0.267 ± 0.041
Error Mean		± 0.14%	**± 0.25%**	0%	7	
			Full External Error ± 0.16	2.15	2σ Confidence Limit	
			Analytical Error ± 0.01	1.8068	Error Magnification	
**Total Fusion Age**		2.42143 ± 0.00147	**6.84 ± 0.01**		24	0.284 ± 0.001
		± 0.06%	**± 0.21%**			
			Full External Error ± 0.15			
			Analytical Error ± 0.00			
**Normal Isochron**	**316.99 ± 31.52**	2.43851 ± 0.01575	**6.88 ± 0.05**	2.90	52.27	
Error Chron	**± 9.94%**	± 0.65%	**± 0.68%**	1%	7	
			Full External Error ± 0.16	2.26	2σ Confidence Limit	
			Analytical Error ± 0.04	1.7038	Error Magnification	
				1	Number of Iterations	
				0.0000069884	Convergence	
**Inverse Isochron**	**315.46 ± 31.97**	2.43944 ± 0.01594	**6.89 ± 0.05**	2.95	52.27	
Error Chron	**± 10.13%**	± 0.65%	**± 0.68%**	1%	7	
			Full External Error ± 0.16	2.26	2σ Confidence Limit	
			Analytical Error ± 0.04	1.7178	Error Magnification	
				3	Number of Iterations	
				0.0002935942	Convergence	
				4%	Spreading Factor	

Incremental Heating			36Ar(a) [fA]	37Ar(ca) [fA]	38Ar(cl) [fA]	39Ar(k) [fA]	40Ar(r) [fA]	Age ± 2σ (Ma)	40Ar(r) (%)	39Ar(k) (%)	K/Ca ± 2σ
17D17983	1.8 %		0.0708886	40.6703	0.0000000	50.6927	125.2769	6.98 ± 0.02	85.56	3.71	0.536 ± 0.009
17D17985	1.9 %		0.0532411	42.7496	0.0306628	53.1576	129.8253	6.89 ± 0.02	89.07	3.89	0.535 ± 0.009
17D17986	2.0 %		0.0385232	36.8168	0.0000000	45.6553	110.9653	6.86 ± 0.02	90.57	3.34	0.533 ± 0.010
17D17988	2.2 %		0.0316981	38.1756	0.0089806	44.9435	109.2854	6.86 ± 0.02	91.97	3.29	0.506 ± 0.009
17D17989	2.4 %		0.0444501	68.6237	0.0000000	75.9752	184.7314	6.86 ± 0.02	93.22	5.56	0.476 ± 0.006
17D17991	2.7 %		0.0422132	81.4622	0.0000000	81.5059	198.5703	6.88 ± 0.01	93.95	5.97	0.430 ± 0.005
17D17992	3.0 %		0.0430354	107.1128	0.0000000	93.0978	227.2349	6.89 ± 0.01	94.56	6.82	0.374 ± 0.004
17D17994	3.4 %	✓	0.0411553	132.9444	0.0000000	98.1296	239.8974	6.90 ± 0.01	95.03	7.19	0.317 ± 0.003
17D17995	3.9 %	✓	0.0456808	179.6709	0.0000000	112.0530	273.9706	6.90 ± 0.01	95.16	8.20	0.268 ± 0.002
17D17997	4.5 %	✓	0.0683531	302.6577	0.0000000	163.8768	400.9050	6.91 ± 0.01	95.06	12.00	0.233 ± 0.002
17D17998	5.2 %	✓	0.0381474	166.1651	0.0000000	88.3567	216.7440	6.92 ± 0.01	94.92	6.47	0.229 ± 0.002
17D18000	6.0 %	✓	0.0538489	163.8046	0.0000000	101.9352	250.3880	6.93 ± 0.01	93.89	7.46	0.268 ± 0.002
17D18001	6.9 %	✓	0.0487833	97.0868	0.0131114	81.0661	198.8114	6.92 ± 0.02	93.10	5.94	0.359 ± 0.004
17D18003	7.9 %	✓	0.0506791	61.4122	0.0000000	68.5142	167.8139	6.91 ± 0.02	91.68	5.02	0.480 ± 0.006
17D18004	9.0 %		0.0533444	41.5377	0.0223478	55.6000	134.3671	6.82 ± 0.02	89.37	4.07	0.576 ± 0.010
17D18006	10.3 %		0.0496858	31.7000	0.0000000	41.0691	97.2257	6.68 ± 0.02	86.76	3.01	0.557 ± 0.012
17D18007	11.6 %		0.0708313	54.1498	0.0181542	42.2013	95.7105	6.40 ± 0.03	81.94	3.09	0.335 ± 0.005
17D18008	12.5 %		0.0437080	43.0952	0.0286973	22.9242	49.9404	6.15 ± 0.04	79.34	1.68	0.229 ± 0.004
17D18010	13.4 %		0.0447827	55.9687	0.0229048	18.7486	37.6746	5.67 ± 0.04	73.90	1.37	0.144 ± 0.002
17D18011	14.6 %		0.0289425	47.4293	0.0023726	10.0792	18.9847	5.32 ± 0.08	68.85	0.74	0.091 ± 0.002
17D18012	16.0 %		0.0283264	72.6445	0.0000000	7.4777	12.8286	4.85 ± 0.11	60.43	0.55	0.044 ± 0.001
17D18014	17.6 %		0.0207460	57.5483	0.0044031	3.7791	6.1460	4.59 ± 0.18	50.00	0.28	0.028 ± 0.001
17D18015	19.3 %		0.0206844	72.9168	0.0034789	2.8622	6.1977	6.11 ± 0.27	50.30	0.21	0.017 ± 0.000
17D18017	21.0 %		0.0168948	72.7266	0.0423018	2.0404	13.5571	18.69 ± 0.53	73.06	0.15	0.012 ± 0.000
		Σ	1.0486438	2069.0696	0.1974154	1365.7415	3307.0523				

Information on Analysis

Project = **O-CONNOR (16-23)**
Sample = **MW14-DL2-3**
Material = **Groundmass**
Location = **Mozambique Ridge**
Region = **Indian Ocean**
Analyst = **Dan Miggins**
Irradiation = **17-OSU-01 (1B32-17)**
J = **0.00156401 ± 0.00000158**
FCT-NM = **28.201 ± 0.023 Ma**

Results	40(r)/39(k) ± 2σ		Age ± 2σ (Ma)		MSWD	39Ar(k) (%,n)	K/Ca ± 2σ
**Age Plateau**	2.44919	± 0.00345	**6.91**	**± 0.02**	3.26	52.27	0.267 ± 0.041
Error Mean		± 0.14%		**± 0.25%**	0%	7	
			Full External Error	± 0.16	2.15	2σ Confidence Limit	
			Analytical Error	± 0.01	1.8068	Error Magnification	
**Total Fusion Age**	2.42143	± 0.00147	**6.84**	**± 0.01**		24	0.284 ± 0.001
		± 0.06%		**± 0.21%**			
			Full External Error	± 0.15			
			Analytical Error	± 0.00			

Normal Isochron			39(k)/36(a) ± 2σ	40(a+r)/36(a) ± 2σ	r.i.
17D17983	1.8 %		715.10 ± 11.82	2062.74 ± 34.00	0.9933
17D17985	1.9 %		998.43 ± 20.28	2733.94 ± 55.42	0.9961
17D17986	2.0 %		1185.14 ± 26.49	3175.98 ± 70.86	0.9963
17D17988	2.2 %		1417.86 ± 40.56	3743.20 ± 106.95	0.9978
17D17989	2.4 %		1709.22 ± 38.00	4451.42 ± 98.79	0.9977
17D17991	2.7 %		1930.82 ± 48.33	4999.49 ± 124.98	0.9983
17D17992	3.0 %		2163.29 ± 53.52	5575.69 ± 137.74	0.9983
17D17994	3.4 %	✓	2384.37 ± 62.74	6124.57 ± 160.96	0.9986
17D17995	3.9 %	✓	2452.96 ± 68.40	6293.01 ± 175.28	0.9987
17D17997	4.5 %	✓	2397.50 ± 58.69	6160.71 ± 150.60	0.9985
17D17998	5.2 %	✓	2316.19 ± 69.91	5977.24 ± 180.23	0.9988
17D18000	6.0 %	✓	1892.99 ± 45.67	4945.33 ± 119.13	0.9983
17D18001	6.9 %	✓	1661.76 ± 38.40	4370.89 ± 100.84	0.9979
17D18003	7.9 %	✓	1351.92 ± 28.49	3606.81 ± 75.84	0.9973
17D18004	9.0 %		1042.28 ± 21.92	2814.36 ± 59.06	0.9967
17D18006	10.3 %		826.58 ± 16.23	2252.31 ± 44.09	0.9949
17D18007	11.6 %		595.80 ± 9.74	1646.75 ± 26.82	0.9929
17D18008	12.5 %		524.48 ± 11.63	1438.09 ± 31.76	0.9922
17D18010	13.4 %		418.66 ± 8.42	1136.78 ± 22.73	0.9865
17D18011	14.6 %		348.25 ± 9.93	951.45 ± 26.96	0.9808
17D18012	16.0 %		263.98 ± 8.41	748.39 ± 23.59	0.9719
17D18014	17.6 %		182.16 ± 6.87	591.75 ± 21.65	0.9334
17D18015	19.3 %		138.37 ± 5.75	595.13 ± 23.24	0.9078
17D18017	21.0 %		120.77 ± 6.25	1097.95 ± 51.68	0.9010

Results	40(a)/36(a) ± 2σ		40(r)/39(k) ± 2σ		Age ± 2σ (Ma)		MSWD
Normal Isochron<br>Error Chron	**316.99**	**± 31.52**<br>**± 9.94%**	2.43851	± 0.01575<br>± 0.65%	**6.88**	**± 0.05**<br>**± 0.68%**	2.90<br>1%
					Full External Error	± 0.16	
					Analytical Error	± 0.04	

Statistics				
	2σ Confidence Limit	2.26	Convergence	0.000006988430
	Error Magnification	1.7038	Number of Iterations	1
	Number of Data Points	7	Calculated Line	Weighted York-2

Inverse Isochron			39(k)/40(a+r) ± 2σ	36(a)/40(a+r) ± 2σ	r.i.
17D17983	1.8 %		0.3466775 ± 0.0006602	0.00048479 ± 0.00000799	0.0339
17D17985	1.9 %		0.3651987 ± 0.0006563	0.00036577 ± 0.00000742	0.0218
17D17986	2.0 %		0.3731561 ± 0.0007202	0.00031486 ± 0.00000702	0.0200
17D17988	2.2 %		0.3787833 ± 0.0007140	0.00026715 ± 0.00000763	0.0145
17D17989	2.4 %		0.3839723 ± 0.0005798	0.00022465 ± 0.00000499	0.0069
17D17991	2.7 %		0.3862030 ± 0.0005696	0.00020002 ± 0.00000500	0.0065
17D17992	3.0 %		0.3879855 ± 0.0005608	0.00017935 ± 0.00000443	0.0047
17D17994	3.4 %	✓	0.3893123 ± 0.0005474	0.00016328 ± 0.00000429	0.0043
17D17995	3.9 %	✓	0.3897914 ± 0.0005439	0.00015891 ± 0.00000443	0.0032
17D17997	4.5 %	✓	0.3891606 ± 0.0005165	0.00016232 ± 0.00000397	0.0020
17D17998	5.2 %	✓	0.3875014 ± 0.0005654	0.00016730 ± 0.00000504	0.0042
17D18000	6.0 %	✓	0.3827828 ± 0.0005407	0.00020221 ± 0.00000487	0.0044
17D18001	6.9 %	✓	0.3801869 ± 0.0005663	0.00022879 ± 0.00000528	0.0063
17D18003	7.9 %	✓	0.3748255 ± 0.0005846	0.00027725 ± 0.00000583	0.0091
17D18004	9.0 %		0.3703444 ± 0.0006311	0.00035532 ± 0.00000746	0.0157
17D18006	10.3 %		0.3669907 ± 0.0007238	0.00044399 ± 0.00000869	0.0218
17D18007	11.6 %		0.3618043 ± 0.0007026	0.00060726 ± 0.00000989	0.0267
17D18008	12.5 %		0.3647091 ± 0.0010112	0.00069537 ± 0.00001536	0.0321
17D18010	13.4 %		0.3682841 ± 0.0012133	0.00087968 ± 0.00001759	0.0463
17D18011	14.6 %		0.3660213 ± 0.0020387	0.00105103 ± 0.00002978	0.0643
17D18012	16.0 %		0.3527361 ± 0.0026509	0.00133621 ± 0.00004212	0.0733
17D18014	17.6 %		0.3078358 ± 0.0041891	0.00168991 ± 0.00006183	0.1001
17D18015	19.3 %		0.2325074 ± 0.0040653	0.00168030 ± 0.00006562	0.0745
17D18017	21.0 %		0.1099998 ± 0.0024692	0.00091079 ± 0.00004287	0.0206

Results	40(a)/36(a) ± 2σ		40(r)/39(k) ± 2σ		Age ± 2σ (Ma)		MSWD
**Inverse Isochron**<br>Error Chron	**315.46**	**± 31.97**<br>**± 10.13%**	2.43944	± 0.01594<br>± 0.65%	**6.89**	**± 0.05**<br>**± 0.68%**	2.95<br>1%
					Full External Error	± 0.16	
					Analytical Error	± 0.04	

Statistics					
Statistics	2σ Confidence Limit	2.26	Convergence	0.0002935942	
	Error Magnification	1.7178	Number of Iterations	3	
	Number of Data Points	7	Calculated Line	Weighted York-2	
	Spreading Factor	3.7%			

Degassing Patterns			36Ar(a) [fA]	%1σ	36Ar(c) [fA]	%1σ	36Ar(ca) [fA]	%1σ	36Ar(cl) [fA]	%1σ	37Ar(ca) [fA]	%1σ	38Ar(a) [fA]	%1σ	38Ar(c) [fA]	%1σ
17D17983	1.8 %		0.0708886	0.82	0.0000000	0.00	0.0108305	0.84	0.0000000	0.00	40.6703	0.83	0.0132491	0.82	0.0000000	0.00
17D17985	1.9 %		0.0532411	1.01	0.0000000	0.00	0.0113842	0.83	0.0000063	77.85	42.7496	0.81	0.0099508	1.01	0.0000000	0.00
17D17986	2.0 %		0.0385232	1.11	0.0000000	0.00	0.0098043	0.93	0.0000000	0.00	36.8168	0.92	0.0072000	1.11	0.0000000	0.00
17D17988	2.2 %		0.0316981	1.43	0.0000000	0.00	0.0101661	0.90	0.0000018	284.60	38.1756	0.88	0.0059244	1.43	0.0000000	0.00
17D17989	2.4 %		0.0444501	1.11	0.0000000	0.00	0.0182745	0.63	0.0000000	0.00	68.6237	0.61	0.0083077	1.11	0.0000000	0.00
17D17991	2.7 %		0.0422132	1.25	0.0000000	0.00	0.0216934	0.58	0.0000000	0.00	81.4622	0.56	0.0078896	1.25	0.0000000	0.00
17D17992	3.0 %		0.0430354	1.23	0.0000000	0.00	0.0285241	0.52	0.0000000	0.00	107.1128	0.49	0.0080433	1.23	0.0000000	0.00
17D17994	3.4 %	✓	0.0411553	1.31	0.0000000	0.00	0.0354031	0.49	0.0000000	0.00	132.9444	0.47	0.0076919	1.31	0.0000000	0.00
17D17995	3.9 %	✓	0.0456808	1.39	0.0000000	0.00	0.0478464	0.46	0.0000000	0.00	179.6709	0.44	0.0085377	1.39	0.0000000	0.00
17D17997	4.5 %	✓	0.0683531	1.22	0.0000000	0.00	0.0805977	0.44	0.0000000	0.00	302.6577	0.42	0.0127752	1.22	0.0000000	0.00
17D17998	5.2 %	✓	0.0381474	1.51	0.0000000	0.00	0.0442498	0.47	0.0000000	0.00	166.1651	0.45	0.0071298	1.51	0.0000000	0.00
17D18000	6.0 %	✓	0.0538489	1.20	0.0000000	0.00	0.0436212	0.47	0.0000000	0.00	163.8046	0.45	0.0100644	1.20	0.0000000	0.00
17D18001	6.9 %	✓	0.0487833	1.15	0.0000000	0.00	0.0258542	0.54	0.0000027	197.35	97.0868	0.52	0.0091176	1.15	0.0000000	0.00
17D18003	7.9 %	✓	0.0506791	1.05	0.0000000	0.00	0.0163541	0.68	0.0000000	0.00	61.4122	0.66	0.0094719	1.05	0.0000000	0.00
17D18004	9.0 %		0.0533444	1.05	0.0000000	0.00	0.0110615	0.84	0.0000046	107.75	41.5377	0.83	0.0099701	1.05	0.0000000	0.00
17D18006	10.3 %		0.0496858	0.98	0.0000000	0.00	0.0084417	1.07	0.0000000	0.00	31.7000	1.06	0.0092863	0.98	0.0000000	0.00
17D18007	11.6 %		0.0708313	0.81	0.0000000	0.00	0.0144201	0.72	0.0000037	127.68	54.1498	0.70	0.0132384	0.81	0.0000000	0.00
17D18008	12.5 %		0.0437080	1.10	0.0000000	0.00	0.0114763	0.83	0.0000059	83.61	43.0952	0.82	0.0081690	1.10	0.0000000	0.00
17D18010	13.4 %		0.0447827	1.00	0.0000000	0.00	0.0149045	0.67	0.0000047	101.45	55.9687	0.66	0.0083699	1.00	0.0000000	0.00
17D18011	14.6 %		0.0289425	1.41	0.0000000	0.00	0.0126304	0.80	0.0000005	978.10	47.4293	0.79	0.0054094	1.41	0.0000000	0.00
17D18012	16.0 %		0.0283264	1.56	0.0000000	0.00	0.0193452	0.62	0.0000000	0.00	72.6445	0.60	0.0052942	1.56	0.0000000	0.00
17D18014	17.6 %		0.0207460	1.80	0.0000000	0.00	0.0153251	0.70	0.0000009	550.17	57.5483	0.68	0.0038774	1.80	0.0000000	0.00
17D18015	19.3 %		0.0206844	1.92	0.0000000	0.00	0.0194178	0.59	0.0000007	666.94	72.9168	0.57	0.0038659	1.92	0.0000000	0.00
17D18017	21.0 %		0.0168948	2.34	0.0000000	0.00	0.0193671	0.61	0.0000087	54.42	72.7266	0.59	0.0031576	2.34	0.0000000	0.00
		Σ	1.0486438	0.25	0.0000000	0.00	0.5509932	0.13	0.0000404	40.27	2069.0696	0.13	0.1959915	0.25	0.0000000	0.00
		Σ							1.5996775	0.17	2069.0696	0.13				

Degassing Patterns	38Ar(k) [fA]	%1σ	38Ar(ca) [fA]	%1σ	38Ar(cl) [fA]	%1σ	39Ar(k) [fA]	%1σ	39Ar(ca) [fA]	%1σ	40Ar(r) [fA]	%1σ	40Ar(a) [fA]	%1σ	40Ar(c) [fA]	%1σ	40Ar(k) [fA]	%1σ
17D17983	0.609884	0.18	0.0029201	12.85	0.0000000	0.00	50.6927	0.08	0.0274769	1.56	125.2769	0.15	20.94759	0.82	0.0000000	0.00	0.1937983	2.66
17D17985	0.639539	0.18	0.0030694	12.85	0.0306628	77.86	53.1576	0.08	0.0288816	1.55	129.8253	0.13	15.73276	1.01	0.0000000	0.00	0.2032215	2.66
17D17986	0.549278	0.18	0.0026434	12.85	0.0000000	0.00	45.6553	0.08	0.0248734	1.61	110.9653	0.13	11.38361	1.11	0.0000000	0.00	0.1745400	2.66
17D17988	0.540715	0.18	0.0027410	12.85	0.0089806	284.60	44.9435	0.08	0.0257914	1.59	109.2854	0.13	9.36678	1.43	0.0000000	0.00	0.1718189	2.66
17D17989	0.914058	0.18	0.0049272	12.83	0.0000000	0.00	75.9752	0.07	0.0463622	1.45	184.7314	0.08	13.13502	1.11	0.0000000	0.00	0.2904533	2.66
17D17991	0.980598	0.17	0.0058490	12.83	0.0000000	0.00	81.5059	0.07	0.0550359	1.43	198.5703	0.08	12.47399	1.25	0.0000000	0.00	0.3115972	2.66
17D17992	1.120060	0.17	0.0076907	12.83	0.0000000	0.00	93.0978	0.07	0.0723654	1.41	227.2349	0.07	12.71695	1.23	0.0000000	0.00	0.3559131	2.66
17D17994	1.180597	0.17	0.0095454	12.83	0.0000000	0.00	98.1296	0.07	0.0898172	1.40	239.8974	0.07	12.16140	1.31	0.0000000	0.00	0.3751495	2.66
17D17995	1.348110	0.17	0.0129004	12.83	0.0000000	0.00	112.0530	0.07	0.1213856	1.39	273.9706	0.07	13.49866	1.39	0.0000000	0.00	0.4283788	2.66
17D17997	1.971602	0.17	0.0217308	12.83	0.0000000	0.00	163.8768	0.07	0.2044755	1.38	400.9050	0.06	20.19834	1.22	0.0000000	0.00	0.6265011	2.66
17D17998	1.063020	0.17	0.0119307	12.83	0.0000000	0.00	88.3567	0.07	0.1122611	1.39	216.7440	0.08	11.27257	1.51	0.0000000	0.00	0.3377878	2.66
17D18000	1.226382	0.17	0.0117612	12.83	0.0000000	0.00	101.9352	0.07	0.1106664	1.39	250.3880	0.08	15.91235	1.20	0.0000000	0.00	0.3896983	2.66
17D18001	0.975306	0.17	0.0069708	12.83	0.0131114	197.35	81.0661	0.07	0.0655919	1.42	198.8114	0.09	14.41548	1.15	0.0000000	0.00	0.3099155	2.66
17D18003	0.824294	0.18	0.0044094	12.84	0.0000000	0.00	68.5142	0.07	0.0414901	1.48	167.8139	0.10	14.97567	1.05	0.0000000	0.00	0.2619298	2.66
17D18004	0.668923	0.18	0.0029824	12.85	0.0223478	107.76	55.6000	0.08	0.0280629	1.56	134.3671	0.13	15.76327	1.05	0.0000000	0.00	0.2125586	2.66
17D18006	0.494103	0.18	0.0022761	12.86	0.0000000	0.00	41.0691	0.09	0.0214165	1.70	97.2257	0.16	14.68214	0.98	0.0000000	0.00	0.1570073	2.66
17D18007	0.507724	0.18	0.0038880	12.84	0.0181542	127.68	42.2013	0.09	0.0365836	1.49	95.7105	0.19	20.93064	0.81	0.0000000	0.00	0.1613355	2.66
17D18008	0.275801	0.20	0.0030942	12.85	0.0286973	83.62	22.9242	0.12	0.0291151	1.55	49.9404	0.30	12.91572	1.10	0.0000000	0.00	0.0876392	2.66
17D18010	0.225564	0.21	0.0040186	12.84	0.0229048	101.45	18.7486	0.14	0.0378125	1.47	37.6746	0.37	13.23328	1.00	0.0000000	0.00	0.0716758	2.66
17D18011	0.121263	0.28	0.0034054	12.84	0.0023726	978.10	10.0792	0.23	0.0320432	1.54	18.9847	0.67	8.55251	1.41	0.0000000	0.00	0.0385328	2.67
17D18012	0.089964	0.35	0.0052159	12.83	0.0000000	0.00	7.4777	0.31	0.0490786	1.45	12.8286	1.08	8.37044	1.56	0.0000000	0.00	0.0285871	2.68
17D18014	0.045467	0.60	0.0041320	12.84	0.0044031	550.18	3.7791	0.58	0.0388797	1.49	6.1460	1.92	6.13046	1.80	0.0000000	0.00	0.0144476	2.72
17D18015	0.034435	0.81	0.0052354	12.83	0.0034789	666.94	2.8622	0.80	0.0492626	1.44	6.1977	2.02	6.11224	1.92	0.0000000	0.00	0.0109420	2.78
17D18017	0.024549	1.11	0.0052218	12.83	0.0423018	54.43	2.0404	1.10	0.0491341	1.45	13.5571	0.92	4.99240	2.34	0.0000000	0.00	0.0078006	2.88
Σ	16.431235	0.04	0.1485592	3.22	0.1974154	40.27	1365.7415	0.02	1.3978634	0.35	3307.0523	0.02	309.87426	0.25	0.0000000	0.00	5.2212296	0.67
Σ					16.973202	0.47			1367.1393	0.02							3622.1478	0.03

**Additional Parameters**			40Ar/39Ar	1σ	37Ar/39Ar	1σ	36Ar/39Ar	1σ	Time (days)	37Ar (decay)	39Ar (decay)	40Ar (moles)
17D17983	1.8 %		2.886784	0.002745	0.801856	0.006658	0.001611	0.000011	125.760	12.018988	1.00088864	7.028E-12
17D17985	1.9 %		2.740570	0.002458	0.803768	0.006563	0.001215	0.000010	125.774	12.022286	1.00088874	6.997E-12
17D17986	2.0 %		2.682205	0.002584	0.805970	0.007449	0.001058	0.000009	125.781	12.023935	1.00088879	5.881E-12
17D17988	2.2 %		2.642339	0.002486	0.848925	0.007529	0.000931	0.000010	125.795	12.027234	1.00088889	5.704E-12
17D17989	2.4 %		2.606587	0.001964	0.902687	0.005548	0.000825	0.000006	125.802	12.028884	1.00088894	9.512E-12
17D17991	2.7 %		2.591385	0.001907	0.998789	0.005625	0.000784	0.000006	125.816	12.032184	1.00088903	1.015E-11
17D17992	3.0 %		2.579234	0.001859	1.149646	0.005743	0.000768	0.000006	125.823	12.033835	1.00088908	1.153E-11
17D17994	3.4 %	✓	2.570103	0.001802	1.353545	0.006367	0.000779	0.000005	125.837	12.037136	1.00088918	1.212E-11
17D17995	3.9 %	✓	2.566517	0.001785	1.601710	0.007105	0.000834	0.000005	125.844	12.038788	1.00088923	1.382E-11
17D17997	4.5 %	✓	2.570249	0.001700	1.844559	0.007786	0.000908	0.000005	125.858	12.042091	1.00088933	2.024E-11
17D17998	5.2 %	✓	2.581179	0.001878	1.878229	0.008490	0.000931	0.000006	125.865	12.043743	1.00088938	1.096E-11
17D18000	6.0 %	✓	2.613433	0.001841	1.605206	0.007231	0.000955	0.000006	125.878	12.047047	1.00088948	1.280E-11
17D18001	6.9 %	✓	2.631979	0.001956	1.196658	0.006258	0.000920	0.000007	125.885	12.048700	1.00088952	1.025E-11
17D18003	7.9 %	✓	2.670114	0.002078	0.895801	0.005937	0.000978	0.000008	125.899	12.052005	1.00088962	8.786E-12
17D18004	9.0 %		2.702648	0.002299	0.746705	0.006212	0.001158	0.000010	125.906	12.053659	1.00088967	7.216E-12
17D18006	10.3 %		2.727266	0.002686	0.771468	0.008231	0.001415	0.000012	125.920	12.056966	1.00088977	5.379E-12
17D18007	11.6 %		2.765351	0.002680	1.282020	0.009053	0.002018	0.000014	125.927	12.058620	1.00088982	5.607E-12
17D18008	12.5 %		2.742252	0.003795	1.877518	0.015552	0.002404	0.000021	125.934	12.060274	1.00088987	3.021E-12
17D18010	13.4 %		2.713645	0.004460	2.979218	0.019952	0.003177	0.000024	125.948	12.063583	1.00088997	2.447E-12
17D18011	14.6 %		2.727235	0.007574	4.690740	0.038540	0.004112	0.000040	125.955	12.065238	1.00089002	1.324E-12
17D18012	16.0 %		2.820293	0.010542	9.651509	0.065104	0.006334	0.000060	125.962	12.066893	1.00089006	1.019E-12
17D18014	17.6 %		3.219189	0.021728	15.072864	0.134620	0.009448	0.000108	125.976	12.070204	1.00089016	5.900E-13
17D18015	19.3 %		4.231922	0.036455	25.045142	0.243309	0.013774	0.000169	125.983	12.072025	1.00089022	5.914E-13
17D18017	21.0 %		8.880895	0.097386	34.804431	0.425899	0.017358	0.000259	125.997	12.075337	1.00089031	8.908E-13

Procedure Blanks		^{36}Ar ± 1σ (SE) [fA]	^{37}Ar ± 1σ (SE) [fA]	^{38}Ar ± 1σ (SE) [fA]	^{39}Ar ± 1σ (SE) [fA]	^{40}Ar ± 1σ (SE) [fA]
17D17983	1.8 %	0.0033500 ± 0.0001718	0.1236791 ± 0.0178072	0.0490958 ± 0.0167534	0.0308774 ± 0.0157593	0.9588893 ± 0.0397218
17D17985	1.9 %	0.0034622 ± 0.0001718	0.1301650 ± 0.0178072	0.0447722 ± 0.0167534	0.0232231 ± 0.0157593	0.9624827 ± 0.0397218
17D17986	2.0 %	0.0035124 ± 0.0001718	0.1323218 ± 0.0178072	0.0441505 ± 0.0167534	0.0208835 ± 0.0157593	0.9659382 ± 0.0397218
17D17988	2.2 %	0.0035997 ± 0.0001718	0.1347866 ± 0.0178072	0.0447497 ± 0.0167534	0.0182084 ± 0.0157593	0.9748284 ± 0.0397218
17D17989	2.4 %	0.0036363 ± 0.0001718	0.1352169 ± 0.0178072	0.0455401 ± 0.0167534	0.0175311 ± 0.0157593	0.9797927 ± 0.0397218
17D17991	2.7 %	0.0036949 ± 0.0001718	0.1347610 ± 0.0178072	0.0471871 ± 0.0167534	0.0167612 ± 0.0157593	0.9897469 ± 0.0397218
17D17992	3.0 %	0.0037166 ± 0.0001718	0.1339826 ± 0.0178072	0.0477423 ± 0.0167534	0.0164209 ± 0.0157593	0.9943967 ± 0.0397218
17D17994	3.4 %	0.0037452 ± 0.0001718	0.1315770 ± 0.0178072	0.0477236 ± 0.0167534	0.0153286 ± 0.0157593	1.0023543 ± 0.0397218
17D17995	3.9 %	0.0037522 ± 0.0001718	0.1300433 ± 0.0178072	0.0469771 ± 0.0167534	0.0144232 ± 0.0157593	1.0054523 ± 0.0397218
17D17997	4.5 %	0.0037522 ± 0.0001718	0.1265300 ± 0.0178072	0.0437403 ± 0.0167534	0.0116276 ± 0.0157593	1.0095252 ± 0.0397218
17D17998	5.2 %	0.0037457 ± 0.0001718	0.1246296 ± 0.0178072	0.0412064 ± 0.0167534	0.0096780 ± 0.0157593	1.0104206 ± 0.0397218
17D18000	6.0 %	0.0037211 ± 0.0001718	0.1207216 ± 0.0178072	0.0343604 ± 0.0167534	0.0046446 ± 0.0157593	1.0098931 ± 0.0397218
17D18001	6.9 %	0.0037038 ± 0.0001718	0.1187788 ± 0.0178072	0.0301339 ± 0.0167534	0.0015953 ± 0.0157593	1.0085209 ± 0.0397218
17D18003	7.9 %	0.0036612 ± 0.0001718	0.1150599 ± 0.0178072	0.0204491 ± 0.0167534	0.0053640 ± 0.0157593	1.0038494 ± 0.0397218
17D18004	9.0 %	0.0036370 ± 0.0001718	0.1133343 ± 0.0178072	0.0152055 ± 0.0167534	0.0091454 ± 0.0157593	1.0007311 ± 0.0397218
17D18006	10.3 %	0.0035856 ± 0.0001718	0.1102593 ± 0.0178072	0.0046137 ± 0.0167534	0.0168724 ± 0.0157593	0.9935446 ± 0.0397218
17D18007	11.6 %	0.0035597 ± 0.0001718	0.1089461 ± 0.0178072	0.0003908 ± 0.0167534	0.0205951 ± 0.0157593	0.9897877 ± 0.0397218
17D18008	12.5 %	0.0035347 ± 0.0001718	0.1078025 ± 0.0178072	0.0049361 ± 0.0167534	0.0240494 ± 0.0157593	0.9861653 ± 0.0397218
17D18010	13.4 %	0.0034904 ± 0.0001718	0.1060703 ± 0.0178072	0.0117252 ± 0.0167534	0.0295347 ± 0.0157593	0.9801855 ± 0.0397218
17D18011	14.6 %	0.0034727 ± 0.0001718	0.1054988 ± 0.0178072	0.0134532 ± 0.0167534	0.0312175 ± 0.0157593	0.9783130 ± 0.0397218
17D18012	16.0 %	0.0034593 ± 0.0001718	0.1051310 ± 0.0178072	0.0136906 ± 0.0167534	0.0319356 ± 0.0157593	0.9775449 ± 0.0397218
17D18014	17.6 %	0.0034492 ± 0.0001718	0.1050146 ± 0.0178072	0.0084254 ± 0.0167534	0.0296086 ± 0.0157593	0.9805307 ± 0.0397218
17D18015	19.3 %	0.0034555 ± 0.0001718	0.1052999 ± 0.0178072	0.0014648 ± 0.0167534	0.0256374 ± 0.0157593	0.9855114 ± 0.0397218
17D18017	21.0 %	0.0034944 ± 0.0001718	0.1064288 ± 0.0178072	0.0205694 ± 0.0167534	0.0121477 ± 0.0157593	1.0025501 ± 0.0397218

Intercept Values		36Ar ± 1σ (SE) [fA]	r2	Regression (type,n)	37Ar ± 1σ (SE) [fA]	r2	Regression (type,n)	38Ar ± 1σ (SE) [fA]	r2	Regression (type,n)	39Ar ± 1σ (SE) [fA]	r2	Regression (type,n)	40Ar ± 1σ (SE) [fA]	r2	Regression (type,n)
17D17983	1.8 %	0.0820800 ± 0.0004879	0.5118	EXP 150 of 150	3.2012189 ± 0.0160722	0.5517	EXP 149 of 150	0.5506834 ± 0.0163774	0.0223	EXP 150 of 150	50.3502298 ± 0.0195404	0.9963	EXP 150 of 150	147.377184 ± 0.063891	0.2550	EXP 150 of 150
17D17985	1.9 %	0.0657297 ± 0.0004548	0.5351	EXP 150 of 150	3.3637630 ± 0.0170590	0.6153	EXP 149 of 150	0.6305063 ± 0.0165444	0.1586	EXP 150 of 150	52.8076639 ± 0.0188434	0.9970	EXP 150 of 150	146.723766 ± 0.051015	0.0054	EXP 150 of 150
17D17986	2.0 %	0.0500722 ± 0.0003460	0.7161	EXP 150 of 150	2.8763057 ± 0.0173887	0.4779	EXP 150 of 150	0.4926274 ± 0.0172983	0.0243	EXP 149 of 150	45.3538231 ± 0.0204326	0.9951	EXP 150 of 150	123.489424 ± 0.040271	0.8382	EXP 150 of 150
17D17988	2.2 %	0.0439343 ± 0.0003773	0.6637	EXP 150 of 150	2.9840204 ± 0.0168271	0.5351	EXP 150 of 150	0.5071193 ± 0.0188643	0.0568	EXP 150 of 150	44.6503892 ± 0.0187533	0.9959	EXP 149 of 150	119.798865 ± 0.033856	0.8058	EXP 149 of 150
17D17989	2.4 %	0.0640666 ± 0.0004003	0.6462	EXP 150 of 150	5.4703249 ± 0.0185118	0.7452	EXP 150 of 150	0.8381568 ± 0.0159481	0.0683	EXP 150 of 150	75.4957520 ± 0.0208397	0.9982	EXP 150 of 150	199.136653 ± 0.025118	0.9876	EXP 150 of 150
17D17991	2.7 %	0.0652638 ± 0.0004353	0.5432	EXP 150 of 150	6.5176754 ± 0.0185823	0.8196	EXP 150 of 150	0.8887296 ± 0.0173050	0.0475	EXP 150 of 150	80.9988670 ± 0.0188073	0.9987	EXP 150 of 150	212.345643 ± 0.031826	0.9885	EXP 150 of 150
17D17992	3.0 %	0.0726586 ± 0.0004261	0.5934	EXP 150 of 150	8.6119517 ± 0.0176330	0.8838	EXP 150 of 150	1.0668776 ± 0.0187355	0.0871	EXP 150 of 150	92.5308226 ± 0.0223969	0.9986	EXP 150 of 150	241.302191 ± 0.027586	0.9950	EXP 150 of 150
17D17994	3.4 %	0.0775033 ± 0.0004227	0.5556	EXP 149 of 150	10.7205713 ± 0.0178719	0.9228	EXP 150 of 150	1.1253502 ± 0.0177778	0.1300	EXP 150 of 150	97.5473598 ± 0.0184089	0.9992	EXP 150 of 150	253.436349 ± 0.029291	0.9960	EXP 150 of 150
17D17995	3.9 %	0.0938582 ± 0.0004977	0.3900	EXP 150 of 150	14.5343424 ± 0.0178179	0.9548	EXP 150 of 150	1.2661468 ± 0.0158060	0.0840	EXP 150 of 150	111.4099486 ± 0.0229369	0.9990	EXP 150 of 150	288.903044 ± 0.029936	0.9974	EXP 150 of 150
17D17997	4.5 %	0.1472546 ± 0.0006049	0.0527	EXP 149 of 150	24.5690225 ± 0.0191426	0.9824	EXP 150 of 150	1.8955560 ± 0.0159361	0.3159	EXP 150 of 150	162.9725246 ± 0.0241714	0.9995	EXP 150 of 150	422.739349 ± 0.031239	0.9992	EXP 150 of 150
17D17998	5.2 %	0.0831289 ± 0.0004429	0.4749	EXP 150 of 150	13.4318580 ± 0.0189018	0.9410	EXP 149 of 150	1.0063890 ± 0.0174347	0.0998	EXP 149 of 150	87.8677656 ± 0.0216620	0.9986	EXP 150 of 150	229.364769 ± 0.027237	0.9933	EXP 149 of 150
17D18000	6.0 %	0.0976259 ± 0.0005143	0.3384	EXP 150 of 150	13.2395261 ± 0.0178143	0.9483	EXP 150 of 150	1.1763360 ± 0.0167019	0.1342	EXP 150 of 150	101.3588667 ± 0.0212669	0.9990	EXP 150 of 150	267.699925 ± 0.031518	0.9962	EXP 150 of 150
17D18001	6.9 %	0.0756138 ± 0.0004611	0.4418	EXP 150 of 150	7.7987397 ± 0.0185909	0.8521	EXP 150 of 150	0.9626926 ± 0.0191862	0.1533	EXP 150 of 150	80.5875470 ± 0.0212431	0.9984	EXP 150 of 150	214.545285 ± 0.028662	0.9904	EXP 149 of 150
17D18003	7.9 %	0.0682424 ± 0.0004420	0.5310	EXP 150 of 150	4.8917883 ± 0.0190411	0.6879	EXP 150 of 150	0.7961832 ± 0.0175702	0.0644	EXP 150 of 150	68.1026278 ± 0.0201280	0.9979	EXP 150 of 150	184.055382 ± 0.029569	0.9782	EXP 150 of 150
17D18004	9.0 %	0.0656914 ± 0.0004773	0.4070	EXP 150 of 150	3.2727120 ± 0.0168261	0.5431	EXP 150 of 150	0.6808299 ± 0.0168345	0.0788	EXP 150 of 150	55.2651782 ± 0.0185087	0.9973	EXP 150 of 150	151.343691 ± 0.039337	0.5120	EXP 150 of 150
17D18006	10.3 %	0.0595868 ± 0.0004018	0.4500	EXP 150 of 150	2.4731347 ± 0.0181348	0.3379	EXP 150 of 150	0.4797039 ± 0.0153986	0.0011	EXP 150 of 150	40.8326525 ± 0.0191637	0.9947	EXP 150 of 150	113.058401 ± 0.032259	0.8532	EXP 150 of 150
17D18007	11.6 %	0.0856963 ± 0.0004739	0.1521	EXP 150 of 150	4.3033836 ± 0.0179565	0.6699	EXP 150 of 150	0.5370814 ± 0.0155740	0.0081	EXP 150 of 150	41.9760041 ± 0.0186290	0.9953	EXP 150 of 150	117.792293 ± 0.035780	0.7077	EXP 150 of 150
17D18008	12.5 %	0.0567061 ± 0.0003987	0.3249	EXP 150 of 150	3.4032785 ± 0.0176161	0.5196	EXP 150 of 150	0.3170262 ± 0.0167631	0.0192	EXP 150 of 150	22.8238557 ± 0.0170225	0.9856	EXP 150 of 150	63.929885 ± 0.018876	0.9935	EXP 150 of 150
17D18010	13.4 %	0.0609987 ± 0.0003526	0.1629	EXP 149 of 150	4.4525968 ± 0.0153622	0.7298	EXP 150 of 150	0.2695494 ± 0.0156877	0.0080	EXP 150 of 150	18.6902806 ± 0.0170804	0.9789	EXP 150 of 150	51.959736 ± 0.019816	0.9939	EXP 150 of 150
17D18011	14.6 %	0.0435254 ± 0.0003233	0.4675	EXP 150 of 150	3.7570975 ± 0.0192248	0.5932	EXP 150 of 150	0.1443636 ± 0.0156540	0.0009	EXP 150 of 150	10.0748524 ± 0.0153354	0.9390	EXP 150 of 150	28.554067 ± 0.018536	0.9971	EXP 150 of 150
17D18012	16.0 %	0.0493872 ± 0.0003540	0.3783	EXP 149 of 150	5.8101575 ± 0.0191678	0.7648	EXP 150 of 150	0.1119863 ± 0.0160890	0.0017	EXP 150 of 150	7.5083477 ± 0.0163959	0.8715	EXP 150 of 150	22.205172 ± 0.019283	0.9973	EXP 150 of 150
17D18014	17.6 %	0.0382018 ± 0.0002840	0.5946	EXP 150 of 150	4.5797428 ± 0.0186792	0.6271	EXP 149 of 150	0.0656317 ± 0.0170938	0.0030	EXP 150 of 150	3.8220854 ± 0.0149137	0.6161	EXP 150 of 150	13.271423 ± 0.017333	0.9979	EXP 150 of 150
17D18015	19.3 %	0.0420914 ± 0.0003079	0.4652	EXP 149 of 150	5.8296438 ± 0.0163200	0.8204	EXP 150 of 150	0.0479329 ± 0.0156411	0.0005	EXP 150 of 150	2.9175837 ± 0.0162121	0.4069	EXP 149 of 150	13.306399 ± 0.018682	0.9976	EXP 150 of 150
17D18017	21.0 %	0.0384382 ± 0.0003080	0.5407	EXP 150 of 150	5.8114080 ± 0.0183198	0.7721	EXP 150 of 150	0.0537857 ± 0.0153738	0.0025	EXP 150 of 150	2.0877525 ± 0.0156377	0.1770	EXP 149 of 150	19.559882 ± 0.017254	0.9974	EXP 150 of 150

Project Info		Analyst	Irradiation	X-pos	Y-pos	Z/H-pos	Project	Experiment	Nmb
17D17983	1.8 %	Dan Miggins	17-OSU-01	0.00	0.00	50.93	Mozambique Ridge\O-Connor (16-23)	17D17979	01
17D17985	1.9 %	Dan Miggins	17-OSU-01	0.00	0.00	50.93	Mozambique Ridge\O-Connor (16-23)	17D17979	01
17D17986	2.0 %	Dan Miggins	17-OSU-01	0.00	0.00	50.93	Mozambique Ridge\O-Connor (16-23)	17D17979	01
17D17988	2.2 %	Dan Miggins	17-OSU-01	0.00	0.00	50.93	Mozambique Ridge\O-Connor (16-23)	17D17979	01
17D17989	2.4 %	Dan Miggins	17-OSU-01	0.00	0.00	50.93	Mozambique Ridge\O-Connor (16-23)	17D17979	01
17D17991	2.7 %	Dan Miggins	17-OSU-01	0.00	0.00	50.93	Mozambique Ridge\O-Connor (16-23)	17D17979	01
17D17992	3.0 %	Dan Miggins	17-OSU-01	0.00	0.00	50.93	Mozambique Ridge\O-Connor (16-23)	17D17979	01
17D17994	3.4 %	Dan Miggins	17-OSU-01	0.00	0.00	50.93	Mozambique Ridge\O-Connor (16-23)	17D17979	01
17D17995	3.9 %	Dan Miggins	17-OSU-01	0.00	0.00	50.93	Mozambique Ridge\O-Connor (16-23)	17D17979	01
17D17997	4.5 %	Dan Miggins	17-OSU-01	0.00	0.00	50.93	Mozambique Ridge\O-Connor (16-23)	17D17979	01
17D17998	5.2 %	Dan Miggins	17-OSU-01	0.00	0.00	50.93	Mozambique Ridge\O-Connor (16-23)	17D17979	01
17D18000	6.0 %	Dan Miggins	17-OSU-01	0.00	0.00	50.93	Mozambique Ridge\O-Connor (16-23)	17D17979	01
17D18001	6.9 %	Dan Miggins	17-OSU-01	0.00	0.00	50.93	Mozambique Ridge\O-Connor (16-23)	17D17979	01
17D18003	7.9 %	Dan Miggins	17-OSU-01	0.00	0.00	50.93	Mozambique Ridge\O-Connor (16-23)	17D17979	01
17D18004	9.0 %	Dan Miggins	17-OSU-01	0.00	0.00	50.93	Mozambique Ridge\O-Connor (16-23)	17D17979	01
17D18006	10.3 %	Dan Miggins	17-OSU-01	0.00	0.00	50.93	Mozambique Ridge\O-Connor (16-23)	17D17979	01
17D18007	11.6 %	Dan Miggins	17-OSU-01	0.00	0.00	50.93	Mozambique Ridge\O-Connor (16-23)	17D17979	01
17D18008	12.5 %	Dan Miggins	17-OSU-01	0.00	0.00	50.93	Mozambique Ridge\O-Connor (16-23)	17D17979	01
17D18010	13.4 %	Dan Miggins	17-OSU-01	0.00	0.00	50.93	Mozambique Ridge\O-Connor (16-23)	17D17979	01
17D18011	14.6 %	Dan Miggins	17-OSU-01	0.00	0.00	50.93	Mozambique Ridge\O-Connor (16-23)	17D17979	01
17D18012	16.0 %	Dan Miggins	17-OSU-01	0.00	0.00	50.93	Mozambique Ridge\O-Connor (16-23)	17D17979	01
17D18014	17.6 %	Dan Miggins	17-OSU-01	0.00	0.00	50.93	Mozambique Ridge\O-Connor (16-23)	17D17979	01
17D18015	19.3 %	Dan Miggins	17-OSU-01	0.00	0.00	50.93	Mozambique Ridge\O-Connor (16-23)	17D17979	01
17D18017	21.0 %	Dan Miggins	17-OSU-01	0.00	0.00	50.93	Mozambique Ridge\O-Connor (16-23)	17D17979	01

Sample Parameters		Sample	Material	Location	Standard Name	Standard (in Ma)	%1σ	Standard Reference	Standard 40Ar/39Ar	%1σ	J	%1σ	Air 40Ar/36Ar	%1σ	MDF (lin)	%1σ	Volume Ratio	Sensitivity (mol/volt)	Day	Month	Year	Hour	Min	Resist
17D17983	1.8 %	MW14-DL2-3	Groundmass	Mozambique Ridge	FCT-NM (1B32-17)	28.201	0.082	Kuiper et al (2008)	10.04942	0.101	0.00156401	0.101	302.527	0.094	0.9941856	0.063	1	4.8E-14	25	MAY	2017	9	54	1
17D17985	1.9 %	MW14-DL2-3	Groundmass	Mozambique Ridge	FCT-NM (1B32-17)	28.201	0.082	Kuiper et al (2008)	10.04942	0.101	0.00156401	0.101	302.527	0.094	0.9941856	0.063	1	4.8E-14	25	MAY	2017	10	14	1
17D17986	2.0 %	MW14-DL2-3	Groundmass	Mozambique Ridge	FCT-NM (1B32-17)	28.201	0.082	Kuiper et al (2008)	10.04942	0.101	0.00156401	0.101	302.527	0.094	0.9941856	0.063	1	4.8E-14	25	MAY	2017	10	24	1
17D17988	2.2 %	MW14-DL2-3	Groundmass	Mozambique Ridge	FCT-NM (1B32-17)	28.201	0.082	Kuiper et al (2008)	10.04942	0.101	0.00156401	0.101	302.527	0.094	0.9941856	0.063	1	4.8E-14	25	MAY	2017	10	44	1
17D17989	2.4 %	MW14-DL2-3	Groundmass	Mozambique Ridge	FCT-NM (1B32-17)	28.201	0.082	Kuiper et al (2008)	10.04942	0.101	0.00156401	0.101	302.527	0.094	0.9941856	0.063	1	4.8E-14	25	MAY	2017	10	54	1
17D17991	2.7 %	MW14-DL2-3	Groundmass	Mozambique Ridge	FCT-NM (1B32-17)	28.201	0.082	Kuiper et al (2008)	10.04942	0.101	0.00156401	0.101	302.527	0.094	0.9941856	0.063	1	4.8E-14	25	MAY	2017	11	14	1
17D17992	3.0 %	MW14-DL2-3	Groundmass	Mozambique Ridge	FCT-NM (1B32-17)	28.201	0.082	Kuiper et al (2008)	10.04942	0.101	0.00156401	0.101	302.527	0.094	0.9941856	0.063	1	4.8E-14	25	MAY	2017	11	24	1
17D17994	3.4 %	MW14-DL2-3	Groundmass	Mozambique Ridge	FCT-NM (1B32-17)	28.201	0.082	Kuiper et al (2008)	10.04942	0.101	0.00156401	0.101	302.527	0.094	0.9941856	0.063	1	4.8E-14	25	MAY	2017	11	44	1
17D17995	3.9 %	MW14-DL2-3	Groundmass	Mozambique Ridge	FCT-NM (1B32-17)	28.201	0.082	Kuiper et al (2008)	10.04942	0.101	0.00156401	0.101	302.527	0.094	0.9941856	0.063	1	4.8E-14	25	MAY	2017	11	54	1
17D17997	4.5 %	MW14-DL2-3	Groundmass	Mozambique Ridge	FCT-NM (1B32-17)	28.201	0.082	Kuiper et al (2008)	10.04942	0.101	0.00156401	0.101	302.527	0.094	0.9941856	0.063	1	4.8E-14	25	MAY	2017	12	14	1
17D17998	5.2 %	MW14-DL2-3	Groundmass	Mozambique Ridge	FCT-NM (1B32-17)	28.201	0.082	Kuiper et al (2008)	10.04942	0.101	0.00156401	0.101	302.527	0.094	0.9941856	0.063	1	4.8E-14	25	MAY	2017	12	24	1
17D18000	6.0 %	MW14-DL2-3	Groundmass	Mozambique Ridge	FCT-NM (1B32-17)	28.201	0.082	Kuiper et al (2008)	10.04942	0.101	0.00156401	0.101	302.527	0.094	0.9941856	0.063	1	4.8E-14	25	MAY	2017	12	44	1
17D18001	6.9 %	MW14-DL2-3	Groundmass	Mozambique Ridge	FCT-NM (1B32-17)	28.201	0.082	Kuiper et al (2008)	10.04942	0.101	0.00156401	0.101	302.527	0.094	0.9941856	0.063	1	4.8E-14	25	MAY	2017	12	54	1
17D18003	7.9 %	MW14-DL2-3	Groundmass	Mozambique Ridge	FCT-NM (1B32-17)	28.201	0.082	Kuiper et al (2008)	10.04942	0.101	0.00156401	0.101	302.527	0.094	0.9941856	0.063	1	4.8E-14	25	MAY	2017	13	14	1
17D18004	9.0 %	MW14-DL2-3	Groundmass	Mozambique Ridge	FCT-NM (1B32-17)	28.201	0.082	Kuiper et al (2008)	10.04942	0.101	0.00156401	0.101	302.527	0.094	0.9941856	0.063	1	4.8E-14	25	MAY	2017	13	24	1
17D18006	10.3 %	MW14-DL2-3	Groundmass	Mozambique Ridge	FCT-NM (1B32-17)	28.201	0.082	Kuiper et al (2008)	10.04942	0.101	0.00156401	0.101	302.527	0.094	0.9941856	0.063	1	4.8E-14	25	MAY	2017	13	44	1
17D18007	11.6 %	MW14-DL2-3	Groundmass	Mozambique Ridge	FCT-NM (1B32-17)	28.201	0.082	Kuiper et al (2008)	10.04942	0.101	0.00156401	0.101	302.527	0.094	0.9941856	0.063	1	4.8E-14	25	MAY	2017	13	54	1
17D18008	12.5 %	MW14-DL2-3	Groundmass	Mozambique Ridge	FCT-NM (1B32-17)	28.201	0.082	Kuiper et al (2008)	10.04942	0.101	0.00156401	0.101	302.527	0.094	0.9941856	0.063	1	4.8E-14	25	MAY	2017	14	4	1
17D18010	13.4 %	MW14-DL2-3	Groundmass	Mozambique Ridge	FCT-NM (1B32-17)	28.201	0.082	Kuiper et al (2008)	10.04942	0.101	0.00156401	0.101	302.527	0.094	0.9941856	0.063	1	4.8E-14	25	MAY	2017	14	24	1
17D18011	14.6 %	MW14-DL2-3	Groundmass	Mozambique Ridge	FCT-NM (1B32-17)	28.201	0.082	Kuiper et al (2008)	10.04942	0.101	0.00156401	0.101	302.527	0.094	0.9941856	0.063	1	4.8E-14	25	MAY	2017	14	34	1
17D18012	16.0 %	MW14-DL2-3	Groundmass	Mozambique Ridge	FCT-NM (1B32-17)	28.201	0.082	Kuiper et al (2008)	10.04942	0.101	0.00156401	0.101	302.527	0.094	0.9941856	0.063	1	4.8E-14	25	MAY	2017	14	44	1
17D18014	17.6 %	MW14-DL2-3	Groundmass	Mozambique Ridge	FCT-NM (1B32-17)	28.201	0.082	Kuiper et al (2008)	10.04942	0.101	0.00156401	0.101	302.527	0.094	0.9941856	0.063	1	4.8E-14	25	MAY	2017	15	4	1
17D18015	19.3 %	MW14-DL2-3	Groundmass	Mozambique Ridge	FCT-NM (1B32-17)	28.201	0.082	Kuiper et al (2008)	10.04942	0.101	0.00156401	0.101	302.527	0.094	0.9941856	0.063	1	4.8E-14	25	MAY	2017	15	15	1
17D18017	21.0 %	MW14-DL2-3	Groundmass	Mozambique Ridge	FCT-NM (1B32-17)	28.201	0.082	Kuiper et al (2008)	10.04942	0.101	0.00156401	0.101	302.527	0.094	0.9941856	0.063	1	4.8E-14	25	MAY	2017	15	35	1

Irradiation Constants		40/36(a)	%1σ	40/36(c)	%1σ	38/36(a)	%1σ	38/36(c)	%1σ	39/37(ca)	%1σ	38/37(ca)	%1σ	36/37(ca)	%1σ	40/39(k)	%1σ	38/39(k)	%1σ	36/38(cl)	%1σ	K/Ca	%1σ	K/Cl	%1σ	Ca/Cl	%1σ
17D17983	1.8 %	295.5	0	0.018	35	0.1869	0	1.493	3	0.000676	1.32	7.18E-05	12.82	0.000266	0.15	0.003823	2.66	0.012031	0.16	0	0	0.43	0	0	0	0	0
17D17985	1.9 %	295.5	0	0.018	35	0.1869	0	1.493	3	0.000676	1.32	7.18E-05	12.82	0.000266	0.15	0.003823	2.66	0.012031	0.16	0	0	0.43	0	0	0	0	0
17D17986	2.0 %	295.5	0	0.018	35	0.1869	0	1.493	3	0.000676	1.32	7.18E-05	12.82	0.000266	0.15	0.003823	2.66	0.012031	0.16	0	0	0.43	0	0	0	0	0
17D17988	2.2 %	295.5	0	0.018	35	0.1869	0	1.493	3	0.000676	1.32	7.18E-05	12.82	0.000266	0.15	0.003823	2.66	0.012031	0.16	0	0	0.43	0	0	0	0	0
17D17989	2.4 %	295.5	0	0.018	35	0.1869	0	1.493	3	0.000676	1.32	7.18E-05	12.82	0.000266	0.15	0.003823	2.66	0.012031	0.16	0	0	0.43	0	0	0	0	0
17D17991	2.7 %	295.5	0	0.018	35	0.1869	0	1.493	3	0.000676	1.32	7.18E-05	12.82	0.000266	0.15	0.003823	2.66	0.012031	0.16	0	0	0.43	0	0	0	0	0
17D17992	3.0 %	295.5	0	0.018	35	0.1869	0	1.493	3	0.000676	1.32	7.18E-05	12.82	0.000266	0.15	0.003823	2.66	0.012031	0.16	0	0	0.43	0	0	0	0	0
17D17994	3.4 %	295.5	0	0.018	35	0.1869	0	1.493	3	0.000676	1.32	7.18E-05	12.82	0.000266	0.15	0.003823	2.66	0.012031	0.16	0	0	0.43	0	0	0	0	0
17D17995	3.9 %	295.5	0	0.018	35	0.1869	0	1.493	3	0.000676	1.32	7.18E-05	12.82	0.000266	0.15	0.003823	2.66	0.012031	0.16	0	0	0.43	0	0	0	0	0
17D17997	4.5 %	295.5	0	0.018	35	0.1869	0	1.493	3	0.000676	1.32	7.18E-05	12.82	0.000266	0.15	0.003823	2.66	0.012031	0.16	0	0	0.43	0	0	0	0	0
17D17998	5.2 %	295.5	0	0.018	35	0.1869	0	1.493	3	0.000676	1.32	7.18E-05	12.82	0.000266	0.15	0.003823	2.66	0.012031	0.16	0	0	0.43	0	0	0	0	0
17D18000	6.0 %	295.5	0	0.018	35	0.1869	0	1.493	3	0.000676	1.32	7.18E-05	12.82	0.000266	0.15	0.003823	2.66	0.012031	0.16	0	0	0.43	0	0	0	0	0
17D18001	6.9 %	295.5	0	0.018	35	0.1869	0	1.493	3	0.000676	1.32	7.18E-05	12.82	0.000266	0.15	0.003823	2.66	0.012031	0.16	0	0	0.43	0	0	0	0	0
17D18003	7.9 %	295.5	0	0.018	35	0.1869	0	1.493	3	0.000676	1.32	7.18E-05	12.82	0.000266	0.15	0.003823	2.66	0.012031	0.16	0	0	0.43	0	0	0	0	0
17D18004	9.0 %	295.5	0	0.018	35	0.1869	0	1.493	3	0.000676	1.32	7.18E-05	12.82	0.000266	0.15	0.003823	2.66	0.012031	0.16	0	0	0.43	0	0	0	0	0
17D18006	10.3 %	295.5	0	0.018	35	0.1869	0	1.493	3	0.000676	1.32	7.18E-05	12.82	0.000266	0.15	0.003823	2.66	0.012031	0.16	0	0	0.43	0	0	0	0	0
17D18007	11.6 %	295.5	0	0.018	35	0.1869	0	1.493	3	0.000676	1.32	7.18E-05	12.82	0.000266	0.15	0.003823	2.66	0.012031	0.16	0	0	0.43	0	0	0	0	0
17D18008	12.5 %	295.5	0	0.018	35	0.1869	0	1.493	3	0.000676	1.32	7.18E-05	12.82	0.000266	0.15	0.003823	2.66	0.012031	0.16	0	0	0.43	0	0	0	0	0
17D18010	13.4 %	295.5	0	0.018	35	0.1869	0	1.493	3	0.000676	1.32	7.18E-05	12.82	0.000266	0.15	0.003823	2.66	0.012031	0.16	0	0	0.43	0	0	0	0	0
17D18011	14.6 %	295.5	0	0.018	35	0.1869	0	1.493	3	0.000676	1.32	7.18E-05	12.82	0.000266	0.15	0.003823	2.66	0.012031	0.16	0	0	0.43	0	0	0	0	0
17D18012	16.0 %	295.5	0	0.018	35	0.1869	0	1.493	3	0.000676	1.32	7.18E-05	12.82	0.000266	0.15	0.003823	2.66	0.012031	0.16	0	0	0.43	0	0	0	0	0
17D18014	17.6 %	295.5	0	0.018	35	0.1869	0	1.493	3	0.000676	1.32	7.18E-05	12.82	0.000266	0.15	0.003823	2.66	0.012031	0.16	0	0	0.43	0	0	0	0	0
17D18015	19.3 %	295.5	0	0.018	35	0.1869	0	1.493	3	0.000676	1.32	7.18E-05	12.82	0.000266	0.15	0.003823	2.66	0.012031	0.16	0	0	0.43	0	0	0	0	0
17D18017	21.0 %	295.5	0	0.018	35	0.1869	0	1.493	3	0.000676	1.32	7.18E-05	12.82	0.000266	0.15	0.003823	2.66	0.012031	0.16	0	0	0.43	0	0	0	0	0

17D17979.AGE >>> MW14-DL2-3 >>> MOZAMBIQUE RIDGE | O-CONNOR (16-23) PROJECT

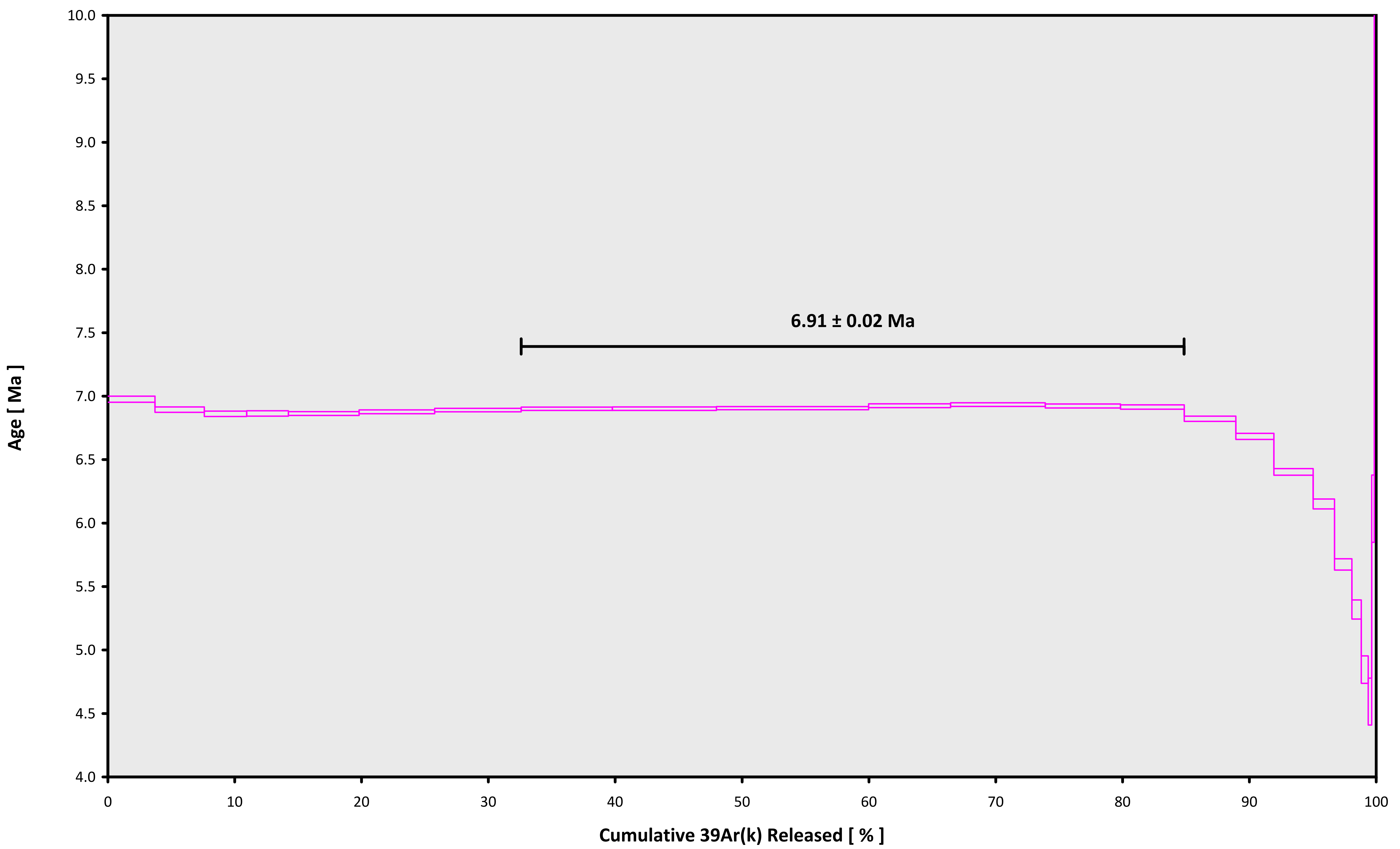

Ar-Ages in Ma

WEIGHTED PLATEAU
6.91 ± 0.02
TOTAL FUSION
6.84 ± 0.01
NORMAL ISOCHRON
6.88 ± 0.05
INVERSE ISOCHRON
6.89 ± 0.05

MSWD (PROBABILITY)
3.26 (0%)

Sample Info

Groundmass
Mozambique Ridge
Dan Miggins

IRR = 17-OSU-01 (1B32-17)
J = 0.00156401 ± 0.00000158

17D17979.AGE >>> MW14-DL2-3 >>> MOZAMBIQUE RIDGE | O-CONNOR (16-23) PROJECT

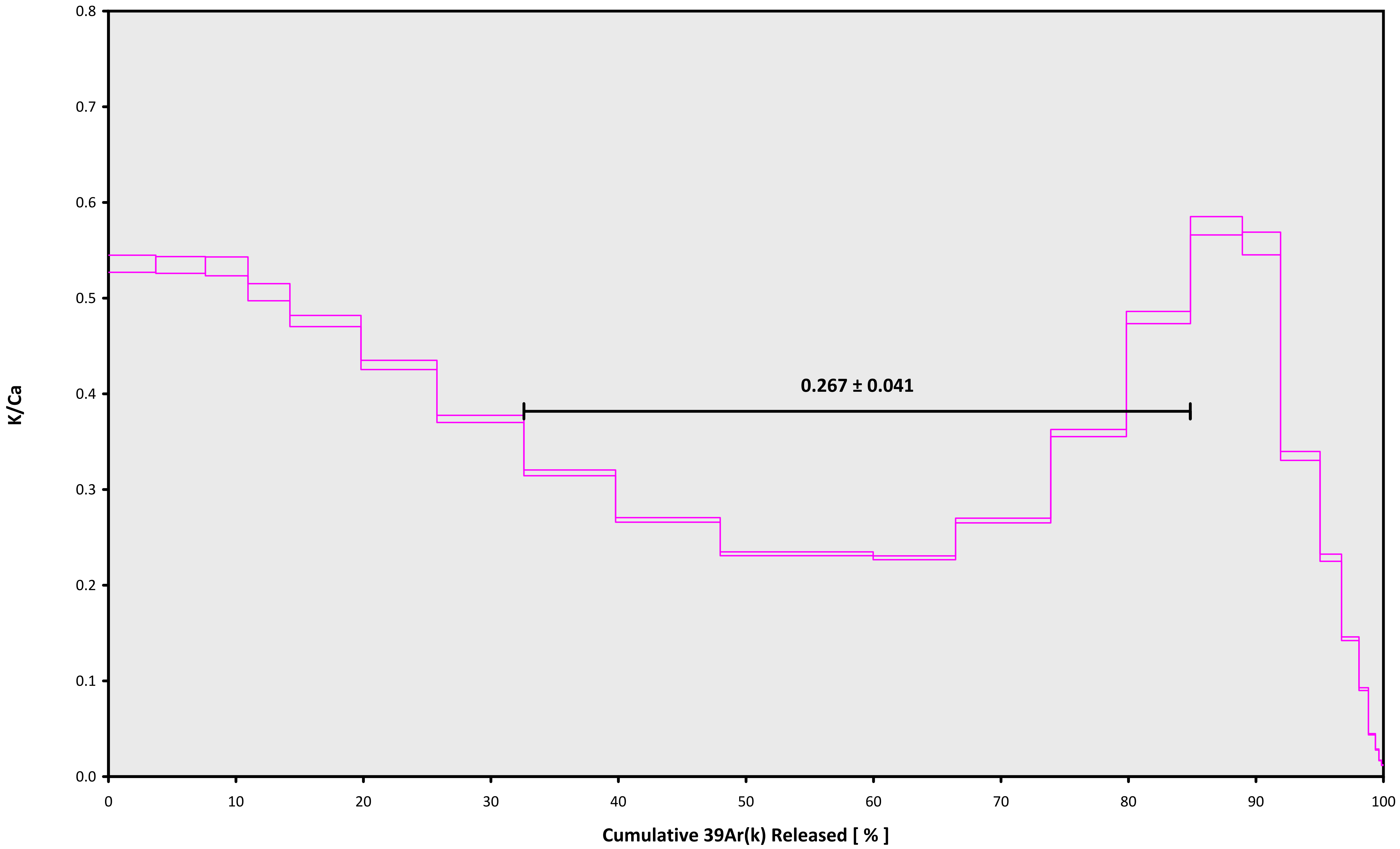

Ar-Ages in Ma

WEIGHTED PLATEAU
6.91 ± 0.02
TOTAL FUSION
6.84 ± 0.01
NORMAL ISOCHRON
6.88 ± 0.05
INVERSE ISOCHRON
6.89 ± 0.05

Sample Info

Groundmass
Mozambique Ridge
Dan Miggins

IRR = 17-OSU-01 (1B32-17)
J = 0.00156401 ± 0.00000158

17D17979.AGE >>> MW14-DL2-3 >>> MOZAMBIQUE RIDGE | O-CONNOR (16-23) PROJECT

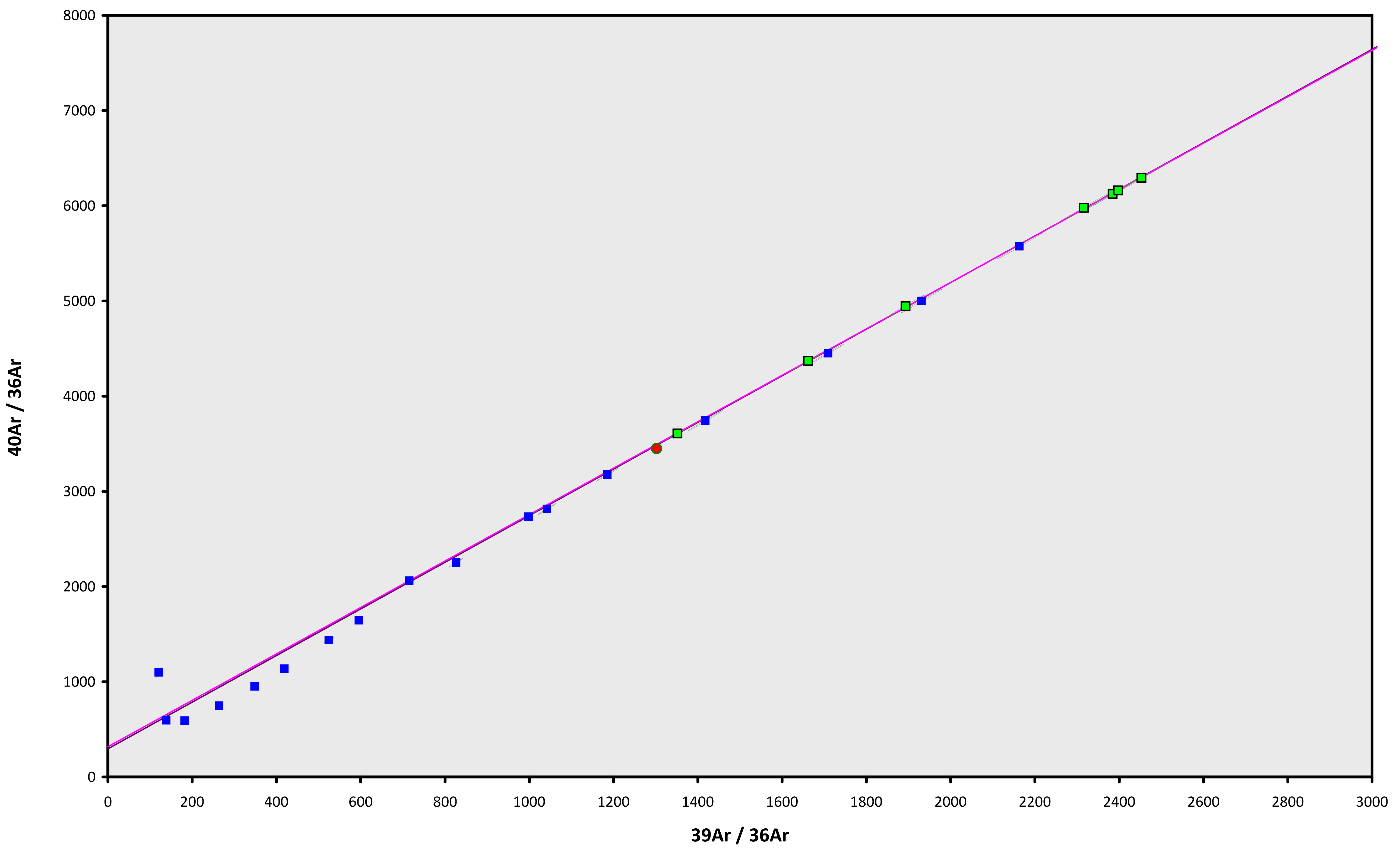

Ar-Ages in Ma

WEIGHTED PLATEAU
6.91 ± 0.02
TOTAL FUSION
6.84 ± 0.01
NORMAL ISOCHRON
6.88 ± 0.05
INVERSE ISOCHRON
6.89 ± 0.05

MSWD (PROBABILITY)
2.90 (1%)

40AR/36AR INTERCEPT
317.0 ± 31.5

Sample Info

Groundmass
Mozambique Ridge
Dan Miggins

IRR = 17-OSU-01 (1B32-17)
J = 0.00156401 ± 0.00000158

17D17979.AGE >>> MW14-DL2-3 >>> MOZAMBIQUE RIDGE | O-CONNOR (16-23) PROJECT

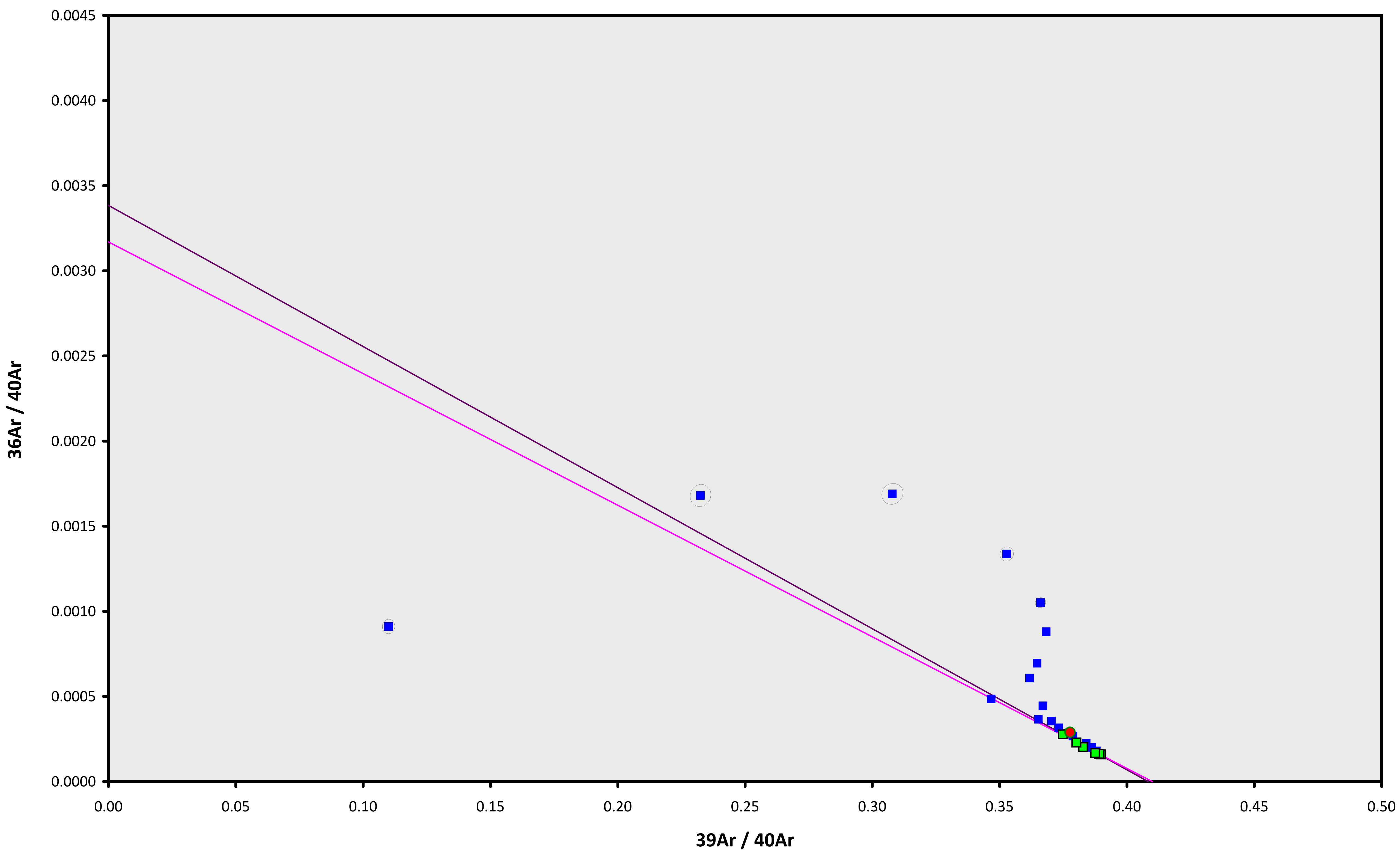

Ar-Ages in Ma

WEIGHTED PLATEAU
6.91 ± 0.02
TOTAL FUSION
6.84 ± 0.01
NORMAL ISOCHRON
6.88 ± 0.05
INVERSE ISOCHRON
6.89 ± 0.05

MSWD (PROBABILITY)
2.95 (1%)

SPREADING FACTOR
3.7%

40AR/36AR INTERCEPT
315.5 ± 32.0

Sample Info

Groundmass
Mozambique Ridge
Dan Miggins

IRR = 17-OSU-01 (1B32-17)
J = 0.00156401 ± 0.00000158

Incremental Heating			36Ar(a) [fA]	37Ar(ca) [fA]	38Ar(cl) [fA]	39Ar(k) [fA]	40Ar(r) [fA]	Age ± 2σ (Ma)	40Ar(r) (%)	39Ar(k) (%)	K/Ca ± 2σ
031_VU107-J-2	12 °C		0.0965643	30.3004	0.0000000	40.82729	34.81070	7.31 ± 0.08	54.67	6.43	0.579 ± 0.010
032_VU107-J-2	13 °C	4	0.0439433	54.1665	0.0000000	63.19633	52.05028	7.06 ± 0.05	79.80	9.95	0.502 ± 0.009
033_VU107-J-2	14 °C	4	0.0298013	81.7134	0.0000000	77.50585	63.24883	7.00 ± 0.04	87.59	12.20	0.408 ± 0.007
035_VU107-J-2	15 °C	4	0.0234960	104.9790	0.0000000	79.55701	64.69419	6.97 ± 0.04	90.13	12.52	0.326 ± 0.005
036_VU107-J-2	17 °C	4	0.0189446	106.5462	0.0000000	71.15459	58.02670	6.99 ± 0.04	91.03	11.20	0.287 ± 0.005
037_VU107-J-2	18 °C	4	0.0163882	86.4687	0.0000000	57.05554	46.46760	6.98 ± 0.05	90.39	8.98	0.284 ± 0.005
039_VU107-J-2	19 °C	4	0.0189657	72.7117	0.0000000	53.15155	43.23633	6.97 ± 0.04	88.34	8.37	0.314 ± 0.005
040_VU107-J-2	21 °C		0.0225855	54.9413	0.0025250	46.31879	37.19045	6.88 ± 0.04	84.57	7.29	0.363 ± 0.006
041_VU107-J-2	22 °C		0.0223360	41.5964	0.0121790	35.57192	27.98783	6.75 ± 0.06	80.69	5.60	0.368 ± 0.006
043_VU107-J-2	25 °C		0.0244584	52.5876	0.0225380	31.21206	23.89835	6.56 ± 0.08	76.53	4.91	0.255 ± 0.004
044_VU107-J-2	28 °C		0.0247921	72.4971	0.0239006	24.06260	17.71521	6.31 ± 0.10	70.47	3.79	0.143 ± 0.002
045_VU107-J-2	31 °C		0.0236020	91.7453	0.0238515	15.03000	11.44931	6.53 ± 0.18	61.86	2.37	0.070 ± 0.001
047_VU107-J-2	39 °C		0.0363465	141.3158	0.0206137	12.07775	8.92679	6.34 ± 0.29	45.11	1.90	0.037 ± 0.001
048_VU107-J-2	69 °C		0.1126433	188.6327	0.0095648	24.17711	21.39082	7.58 ± 0.21	38.86	3.81	0.055 ± 0.001
049_VU107-J-2	16 °C		0.0138561	160.3716	0.0145661	4.29672	2.95963	5.91 ± 0.80	41.68	0.68	0.012 ± 0.000
		Σ	0.5287231	1340.5737	0.1297386	635.19513	514.05302				

Information on Analysis

Sample = 031_VU107-J-2
Material = groundmass
Location = MW14 DL2-3
Analyst = Klaudia Kuiper
Project = VU107
Mass Discrimination Law = LIN
Irradiation = VU107
J = 0.00468970 ± 0.00000469
FCs = 28.201 ± 0.023 Ma

Results	40(r)/39(k)	± 2σ	Age (Ma)	± 2σ	MSWD	39Ar(k) (%,n)	K/Ca ± 2σ
Age Plateau	0.81588	± 0.00301<br>± 0.37%	6.99	± 0.03<br>± 0.42%	2.07<br>7%	63.23<br>6	0.326 ± 0.054
			Full External Error	± 0.15	2.26	2σ Confidence Limit	
			Analytical Error	± 0.03	1.4404	Error Magnification	
Total Fusion Age	0.80928	± 0.00218<br>± 0.27%	6.94	± 0.02<br>± 0.33%		15	0.204 ± 0.001
			Full External Error	± 0.15			
			Analytical Error	± 0.02			

Normal Isochron			39(k)/36(a) ± 2σ	40(a+r)/36(a) ± 2σ	r.i.
031_VU107-J-2	12 °C		422.80 ± 5.05	659.05 ± 7.55	0.9501
032_VU107-J-2	13 °C	4	1438.13 ± 32.02	1483.05 ± 32.69	0.9872
033_VU107-J-2	14 °C	4	2600.76 ± 83.83	2420.91 ± 77.69	0.9940
035_VU107-J-2	15 °C	4	3385.97 ± 157.17	3051.97 ± 141.38	0.9973
036_VU107-J-2	17 °C	4	3755.94 ± 193.17	3361.53 ± 172.59	0.9978
037_VU107-J-2	18 °C	4	3481.51 ± 202.09	3133.99 ± 181.67	0.9982
039_VU107-J-2	19 °C	4	2802.51 ± 107.42	2578.28 ± 98.52	0.9957
040_VU107-J-2	21 °C		2050.82 ± 60.38	1945.21 ± 56.96	0.9926
041_VU107-J-2	22 °C		1592.58 ± 51.51	1551.60 ± 49.92	0.9933
043_VU107-J-2	25 °C		1276.13 ± 46.49	1275.66 ± 46.17	0.9910
044_VU107-J-2	28 °C		970.58 ± 36.54	1013.11 ± 37.82	0.9901
045_VU107-J-2	31 °C		636.81 ± 28.54	783.66 ± 34.65	0.9851
047_VU107-J-2	39 °C		332.29 ± 12.36	544.16 ± 20.08	0.9901
048_VU107-J-2	69 °C		214.63 ± 3.79	488.46 ± 8.50	0.9788
049_VU107-J-2	16 °C		310.10 ± 30.35	512.16 ± 49.76	0.9917

Results	40(a)/36(a) ± 2σ	40(r)/39(k) ± 2σ	Age ± 2σ (Ma)	MSWD
Normal Isochron	320.57 ± 15.82 ± 4.93%	0.80761 ± 0.00633 ± 0.78%	6.92 ± 0.06 ± 0.81%	0.35 84%
			Full External Error ± 0.16	
			Analytical Error ± 0.05	

Statistics				
	2σ Confidence Limit	2.41	Convergence	0.000007879746
	Error Magnification	1.0000	Number of Iterations	68
	Number of Data Points	6	Calculated Line	Weighted York-2

Inverse Isochron			39(k)/40(a+r) ± 2σ	36(a)/40(a+r) ± 2σ	r.i.
031_VU107-J-2	12 °C		0.6415257 ± 0.0023906	0.00151733 ± 0.00001737	0.0295
032_VU107-J-2	13 °C	4	0.9697153 ± 0.0034371	0.00067429 ± 0.00001486	0.0184
033_VU107-J-2	14 °C	4	1.0742873 ± 0.0037913	0.00041307 ± 0.00001326	0.0150
035_VU107-J-2	15 °C	4	1.1094398 ± 0.0037717	0.00032766 ± 0.00001518	0.0090
036_VU107-J-2	17 °C	4	1.1173285 ± 0.0038279	0.00029748 ± 0.00001527	0.0073
037_VU107-J-2	18 °C	4	1.1108846 ± 0.0038405	0.00031908 ± 0.00001850	0.0074
039_VU107-J-2	19 °C	4	1.0869722 ± 0.0038422	0.00038786 ± 0.00001482	0.0128
040_VU107-J-2	21 °C		1.0542918 ± 0.0037748	0.00051408 ± 0.00001505	0.0169
041_VU107-J-2	22 °C		1.0264149 ± 0.0038239	0.00064450 ± 0.00002074	0.0124
043_VU107-J-2	25 °C		1.0003659 ± 0.0048706	0.00078391 ± 0.00002837	0.0185
044_VU107-J-2	28 °C		0.9580160 ± 0.0050717	0.00098706 ± 0.00003685	0.0107
045_VU107-J-2	31 °C		0.8126109 ± 0.0062693	0.00127607 ± 0.00005642	0.0080
047_VU107-J-2	39 °C		0.6106533 ± 0.0031821	0.00183769 ± 0.00006782	0.0128
048_VU107-J-2	69 °C		0.4394114 ± 0.0015896	0.00204726 ± 0.00003561	0.0303
049_VU107-J-2	16 °C		0.6054698 ± 0.0076013	0.00195252 ± 0.00018969	0.0062

Results	40(a)/36(a) ± 2σ	40(r)/39(k) ± 2σ	Age ± 2σ (Ma)	MSWD
Inverse Isochron	321.16 ± 15.79<br>± 4.92%	0.80740 ± 0.00632<br>± 0.78%	6.92 ± 0.06<br>± 0.81%<br>Full External Error ± 0.16<br>Analytical Error ± 0.05	0.35<br>84%

Statistics				
	2σ Confidence Limit	2.41	Convergence	0.0000299675
	Error Magnification	1.0000	Number of Iterations	3
	Number of Data Points	6	Calculated Line	Weighted York-2
	Spreading Factor	11.9%		

Relative Abundances			36Ar [fA]	%1σ	37Ar [fA]	%1σ	38Ar [fA]	%1σ	39Ar [fA]	%1σ	40Ar [fA]	%1σ	40(r)/39(k) ± 2σ	Age ± 2σ (Ma)	40Ar(r) (%)	39Ar(k) (%)	K/Ca ± 2σ
031_VU107-J-2	12 °C		0.1045636	0.520	30.3004	0.846	0.4871454	0.301	40.84768	0.178	63.67604	0.056	0.85263 ± 0.00890	7.31 ± 0.08	54.67	6.43	0.579 ± 0.010
032_VU107-J-2	13 °C	4	0.0582432	0.788	54.1665	0.847	0.7345823	0.299	63.23279	0.167	65.22433	0.059	0.82363 ± 0.00549	7.06 ± 0.05	79.80	9.95	0.502 ± 0.009
033_VU107-J-2	14 °C	4	0.0513736	0.823	81.7134	0.812	0.9053087	0.279	77.56085	0.164	72.21295	0.065	0.81605 ± 0.00472	7.00 ± 0.04	87.59	12.20	0.408 ± 0.007
035_VU107-J-2	15 °C	4	0.0512105	0.906	104.9790	0.799	0.9350753	0.258	79.62766	0.159	71.77759	0.059	0.81318 ± 0.00496	6.97 ± 0.04	90.13	12.52	0.326 ± 0.005
036_VU107-J-2	17 °C	4	0.0470728	0.827	106.5462	0.815	0.8451883	0.286	71.22629	0.162	63.74398	0.056	0.81550 ± 0.00497	6.99 ± 0.04	91.03	11.20	0.287 ± 0.005
037_VU107-J-2	18 °C	4	0.0392159	1.050	86.4687	0.816	0.6770374	0.276	57.11373	0.162	51.40952	0.060	0.81443 ± 0.00573	6.98 ± 0.05	90.39	8.98	0.284 ± 0.005
039_VU107-J-2	19 °C	4	0.0381616	0.797	72.7117	0.801	0.6384714	0.275	53.20049	0.164	48.94444	0.065	0.81345 ± 0.00502	6.97 ± 0.04	88.34	8.37	0.314 ± 0.005
040_VU107-J-2	21 °C		0.0370910	0.788	54.9413	0.846	0.5677029	0.267	46.35577	0.166	43.97340	0.066	0.80292 ± 0.00519	6.88 ± 0.04	84.57	7.29	0.363 ± 0.006
041_VU107-J-2	22 °C		0.0333226	1.019	41.5964	0.845	0.4471653	0.318	35.59992	0.176	34.68707	0.060	0.78680 ± 0.00675	6.75 ± 0.06	80.69	5.60	0.368 ± 0.006
043_VU107-J-2	25 °C		0.0383510	1.087	52.5876	0.842	0.4051264	0.315	31.24745	0.226	31.22748	0.090	0.76568 ± 0.00933	6.56 ± 0.08	76.53	4.91	0.255 ± 0.004
044_VU107-J-2	28 °C		0.0439414	0.952	72.4971	0.805	0.3199720	0.297	24.11139	0.254	25.13782	0.072	0.73621 ± 0.01218	6.31 ± 0.10	70.47	3.79	0.143 ± 0.002
045_VU107-J-2	31 °C		0.0478328	0.955	91.7453	0.815	0.2103138	0.384	15.09174	0.375	18.50886	0.082	0.76176 ± 0.02161	6.53 ± 0.18	61.86	2.37	0.070 ± 0.001
047_VU107-J-2	39 °C		0.0736626	0.745	141.3158	0.808	0.1737266	0.512	12.17286	0.246	19.78880	0.078	0.73911 ± 0.03348	6.34 ± 0.29	45.11	1.90	0.037 ± 0.001
048_VU107-J-2	69 °C		0.1624464	0.510	188.6327	0.817	0.3235829	0.308	24.30406	0.166	55.04239	0.069	0.88475 ± 0.02467	7.58 ± 0.21	38.86	3.81	0.055 ± 0.001
049_VU107-J-2	16 °C		0.0562004	0.917	160.3716	0.796	0.0692113	0.653	4.40465	0.597	7.10021	0.138	0.68881 ± 0.09401	5.91 ± 0.80	41.68	0.68	0.012 ± 0.000
		Σ	0.8826893	0.207	1340.5737	0.233	7.7396100	0.083	636.09733	0.050	672.45486	0.018					

Information on Analysis and Constants Used in Calculations

Sample = 031_VU107-J-2
Material = groundmass
Location = MW14 DL2-3
Analyst = Klaudia Kuiper
Project = VU107
Mass Discrimination Law = LIN
Irradiation = VU107
J = 0.00468970 ± 0.00000469
FCs = 28.201 ± 0.023 Ma
IGSN = Undefined
Preferred Age = Undefined
Classification = Undefined
Experiment Type = Undefined
Extraction Method = Undefined
Heating = 720 sec
Isolation = 18.00 min
Instrument = HELIX
Lithology = Undefined
Lat-Lon = Undefined - Undefined
Feature = Undefined

Age Equations = Min et al. (2000)
Negative Intensities = Allowed
Decay Constant 40K = 5.460 ± 0.053 E-10 1/a
Decay Constant 39Ar = 2.940 ± 0.016 E-07 1/h
Decay Constant 37Ar = 8.230 ± 0.012 E-04 1/h
Decay Constant 36Cl = 2.257 ± 0.015 E-06 1/a
Decay Activity 40K(EC,β⁺) = 3.310 ± 0.030 1/gs
Decay Activity 40K(β⁻) = 27.890 ± 0.150 1/gs
Atmospheric Ratio 40/36(a) = 298.56 ± 0.31
Atmospheric Ratio 38/36(a) = 0.1885 ± 0.0003
Production Ratio 39/37(ca) = 0.000673 ± 0.000004
Production Ratio 36/37(ca) = 0.000264 ± 0.000002
Production Ratio 40/39(k) = 0.000860 ± 0.000070
Production Ratio 38/39(k) = 0.012110 ± 0.000030
Production Ratio 36/38(cl) = 262.80 ± 1.71
Scaling Ratio K/Ca = 0.430
Abundance Ratio 40K/K = 1.1700 ± 0.0100 E-04
Atomic Weight K = 39.0983 ± 0.0001 g

Results

Results	40(a)/36(a) ± 2σ	40(r)/39(k) ± 2σ	Age ± 2σ (Ma)	MSWD	39Ar(k) (%,n)	K/Ca ± 2σ
Age Plateau		0.81588 ± 0.00301 ± 0.37%	6.99 ± 0.03 ± 0.42%	2.07 7%	63.23 6	0.326 ± 0.054
			Full External Error ± 0.15	2.26	2σ Confidence Limit	
			Analytical Error ± 0.03	1.4404	Error Magnification	
Total Fusion Age		0.80928 ± 0.00218 ± 0.27%	6.94 ± 0.02 ± 0.33%		15	0.204 ± 0.001
			Full External Error ± 0.15			
			Analytical Error ± 0.02			
Normal Isochron	320.57 ± 15.82 ± 4.93%	0.80761 ± 0.00633 ± 0.78%	6.92 ± 0.06 ± 0.81%	0.35 84%	63.23 6	
			Full External Error ± 0.16	2.41	2σ Confidence Limit	
			Analytical Error ± 0.05	1.0000	Error Magnification	
				68	Number of Iterations	
				0.0000078797	Convergence	
Inverse Isochron	321.16 ± 15.79 ± 4.92%	0.80740 ± 0.00632 ± 0.78%	6.92 ± 0.06 ± 0.81%	0.35 84%	63.23 6	
			Full External Error ± 0.16	2.41	2σ Confidence Limit	
			Analytical Error ± 0.05	1.0000	Error Magnification	
				3	Number of Iterations	
				0.0000299675	Convergence	
				12%	Spreading Factor	

Degassing Patterns			36Ar(a) [fA]	%1σ	36Ar(c) [fA]	%1σ	36Ar(ca) [fA]	%1σ	36Ar(cl) [fA]	%1σ	37Ar(ca) [fA]	%1σ	38Ar(a) [fA]	%1σ	38Ar(c) [fA]	%1σ
031_VU107-J-2	12 °C		0.0965643	0.57	0.0000000	0.00	0.0079993	1.06	0.0000000	0.00	30.3004	0.85	0.0182024	0.59	0.0000000	0.00
032_VU107-J-2	13 °C	4	0.0439433	1.10	0.0000000	0.00	0.0142999	1.06	0.0000000	0.00	54.1665	0.85	0.0082833	1.11	0.0000000	0.00
033_VU107-J-2	14 °C	4	0.0298013	1.60	0.0000000	0.00	0.0215723	1.03	0.0000000	0.00	81.7134	0.81	0.0056175	1.61	0.0000000	0.00
035_VU107-J-2	15 °C	4	0.0234960	2.32	0.0000000	0.00	0.0277145	1.02	0.0000000	0.00	104.9790	0.80	0.0044290	2.32	0.0000000	0.00
036_VU107-J-2	17 °C	4	0.0189446	2.57	0.0000000	0.00	0.0281282	1.04	0.0000000	0.00	106.5462	0.82	0.0035710	2.57	0.0000000	0.00
037_VU107-J-2	18 °C	4	0.0163882	2.90	0.0000000	0.00	0.0228277	1.04	0.0000000	0.00	86.4687	0.82	0.0030892	2.90	0.0000000	0.00
039_VU107-J-2	19 °C	4	0.0189657	1.91	0.0000000	0.00	0.0191959	1.03	0.0000000	0.00	72.7117	0.80	0.0035750	1.92	0.0000000	0.00
040_VU107-J-2	21 °C		0.0225855	1.46	0.0000000	0.00	0.0145045	1.06	0.0000011	102.93	54.9413	0.85	0.0042574	1.47	0.0000000	0.00
041_VU107-J-2	22 °C		0.0223360	1.61	0.0000000	0.00	0.0109814	1.06	0.0000051	17.89	41.5964	0.84	0.0042103	1.62	0.0000000	0.00
043_VU107-J-2	25 °C		0.0244584	1.81	0.0000000	0.00	0.0138831	1.06	0.0000095	8.96	52.5876	0.84	0.0046104	1.81	0.0000000	0.00
044_VU107-J-2	28 °C		0.0247921	1.87	0.0000000	0.00	0.0191392	1.03	0.0000101	6.64	72.4971	0.81	0.0046733	1.87	0.0000000	0.00
045_VU107-J-2	31 °C		0.0236020	2.21	0.0000000	0.00	0.0242208	1.04	0.0000101	5.28	91.7453	0.81	0.0044490	2.21	0.0000000	0.00
047_VU107-J-2	39 °C		0.0363465	1.84	0.0000000	0.00	0.0373074	1.03	0.0000087	5.40	141.3158	0.81	0.0068513	1.85	0.0000000	0.00
048_VU107-J-2	69 °C		0.1126433	0.87	0.0000000	0.00	0.0497990	1.04	0.0000040	15.84	188.6327	0.82	0.0212333	0.88	0.0000000	0.00
049_VU107-J-2	16 °C		0.0138561	4.86	0.0000000	0.00	0.0423381	1.02	0.0000061	4.21	160.3716	0.80	0.0026119	4.86	0.0000000	0.00
		∑	0.5287231	0.40	0.0000000	0.00	0.3539115	0.30	0.0000547	3.74	1340.5737	0.23	0.0996643	0.40	0.0000000	0.00
		∑							0.8826893	0.27	1340.5737	0.23				

Degassing Patterns	38Ar(k) [fA]	%1σ	38Ar(ca) [fA]	%1σ	38Ar(cl) [fA]	%1σ	39Ar(k) [fA]	%1σ	39Ar(ca) [fA]	%1σ	40Ar(r) [fA]	%1σ	40Ar(a) [fA]	%1σ	40Ar(c) [fA]	%1σ	40Ar(k) [fA]	%1σ
031_VU107-J-2	0.4944185	0.31	0.0000000	0.00	0.0000000	0.00	40.82729	0.18	0.0203922	1.01	34.81070	0.49	28.83023	0.58	0.0000000	0.00	0.0351115	8.10
032_VU107-J-2	0.7653076	0.30	0.0000000	0.00	0.0000000	0.00	63.19633	0.17	0.0364540	1.01	52.05028	0.29	13.11970	1.11	0.0000000	0.00	0.0543488	8.10
033_VU107-J-2	0.9385959	0.30	0.0000000	0.00	0.0000000	0.00	77.50585	0.16	0.0549931	0.98	63.24883	0.24	8.89747	1.61	0.0000000	0.00	0.0666550	8.10
035_VU107-J-2	0.9634354	0.30	0.0000000	0.00	0.0000000	0.00	79.55701	0.16	0.0706509	0.97	64.69419	0.26	7.01498	2.32	0.0000000	0.00	0.0684190	8.10
036_VU107-J-2	0.8616821	0.30	0.0000000	0.00	0.0000000	0.00	71.15459	0.16	0.0717056	0.98	58.02670	0.26	5.65609	2.57	0.0000000	0.00	0.0611929	8.10
037_VU107-J-2	0.6909426	0.30	0.0000000	0.00	0.0000000	0.00	57.05554	0.16	0.0581934	0.98	46.46760	0.31	4.89286	2.90	0.0000000	0.00	0.0490678	8.10
039_VU107-J-2	0.6436653	0.30	0.0000000	0.00	0.0000000	0.00	53.15155	0.16	0.0489350	0.97	43.23633	0.26	5.66239	1.91	0.0000000	0.00	0.0457103	8.10
040_VU107-J-2	0.5609206	0.30	0.0000000	0.00	0.0025250	102.93	46.31879	0.17	0.0369755	1.01	37.19045	0.28	6.74311	1.47	0.0000000	0.00	0.0398342	8.10
041_VU107-J-2	0.4307760	0.31	0.0000000	0.00	0.0121790	17.91	35.57192	0.18	0.0279944	1.01	27.98783	0.39	6.66864	1.61	0.0000000	0.00	0.0305919	8.10
043_VU107-J-2	0.3779780	0.34	0.0000000	0.00	0.0225380	9.01	31.21206	0.23	0.0353915	1.01	23.89835	0.57	7.30229	1.81	0.0000000	0.00	0.0268424	8.10
044_VU107-J-2	0.2913981	0.36	0.0000000	0.00	0.0239006	6.70	24.06260	0.25	0.0487906	0.98	17.71521	0.79	7.40191	1.87	0.0000000	0.00	0.0206938	8.10
045_VU107-J-2	0.1820133	0.45	0.0000000	0.00	0.0238515	5.36	15.03000	0.38	0.0617446	0.98	11.44931	1.37	7.04662	2.21	0.0000000	0.00	0.0129258	8.11
047_VU107-J-2	0.1462616	0.35	0.0000000	0.00	0.0206137	5.48	12.07775	0.25	0.0951055	0.98	8.92679	2.25	10.85162	1.85	0.0000000	0.00	0.0103869	8.10
048_VU107-J-2	0.2927848	0.30	0.0000000	0.00	0.0095648	15.87	24.17711	0.17	0.1269498	0.98	21.39082	1.38	33.63078	0.87	0.0000000	0.00	0.0207923	8.10
049_VU107-J-2	0.0520333	0.66	0.0000000	0.00	0.0145661	4.31	4.29672	0.61	0.1079301	0.97	2.95963	6.80	4.13688	4.86	0.0000000	0.00	0.0036952	8.12
∑	7.6922130	0.09	0.0000000	0.00	0.1297386	3.76	635.19513	0.05	0.9022061	0.28	514.05302	0.12	157.85558	0.40	0.0000000	0.00	0.5462678	2.39
∑					7.9216159	0.11			636.09733	0.05							672.45486	0.13

Additional Parameters			40Ar/39Ar	1σ	37Ar/39Ar	1σ	36Ar/39Ar	1σ	Time (days)	37Ar (decay)	39Ar (decay)	40Ar (moles)
031_VU107-J-2	12 °C		1.558865	0.002902	0.741790	0.006413	0.002560	0.000014	259.403	172.518508	1.00184170	6.368E-12
032_VU107-J-2	13 °C	4	1.031496	0.001826	0.856620	0.007393	0.000921	0.000007	259.424	172.591882	1.00184186	6.522E-12
033_VU107-J-2	14 °C	4	0.931049	0.001640	1.053539	0.008724	0.000662	0.000006	259.446	172.665286	1.00184201	7.221E-12
035_VU107-J-2	15 °C	4	0.901415	0.001529	1.318374	0.010740	0.000643	0.000006	259.487	172.805078	1.00184230	7.178E-12
036_VU107-J-2	17 °C	4	0.894950	0.001530	1.495883	0.012431	0.000661	0.000006	259.508	172.878573	1.00184245	6.374E-12
037_VU107-J-2	18 °C	4	0.900125	0.001553	1.513974	0.012587	0.000687	0.000007	259.530	172.952100	1.00184260	5.141E-12
039_VU107-J-2	19 °C	4	0.920000	0.001623	1.366749	0.011173	0.000717	0.000006	259.572	173.094498	1.00184290	4.894E-12
040_VU107-J-2	21 °C		0.948607	0.001695	1.185210	0.010224	0.000800	0.000006	259.592	173.165741	1.00184304	4.397E-12
041_VU107-J-2	22 °C		0.974358	0.001812	1.168441	0.010081	0.000936	0.000010	259.614	173.239389	1.00184320	3.469E-12
043_VU107-J-2	25 °C		0.999361	0.002429	1.682941	0.014667	0.001227	0.000014	259.656	173.382024	1.00184349	3.123E-12
044_VU107-J-2	28 °C		1.042570	0.002753	3.006758	0.025389	0.001822	0.000018	259.677	173.455765	1.00184364	2.514E-12
045_VU107-J-2	31 °C		1.226423	0.004712	6.079169	0.054542	0.003169	0.000033	259.699	173.529537	1.00184380	1.851E-12
047_VU107-J-2	39 °C		1.625649	0.004203	11.609088	0.098020	0.006051	0.000047	259.740	173.670028	1.00184408	1.979E-12
048_VU107-J-2	69 °C		2.264740	0.004076	7.761366	0.064696	0.006684	0.000036	259.761	173.743891	1.00184424	5.504E-12
049_VU107-J-2	16 °C		1.611978	0.009875	36.409596	0.362249	0.012759	0.000140	259.783	173.817786	1.00184439	7.100E-13

Procedure Blanks		36Ar [fA]	1σ	37Ar [fA]	1σ	38Ar [fA]	1σ	39Ar [fA]	1σ	40Ar [fA]	1σ
031_VU107-J-2	12 °C	0.0091067	0.0001280	0.0126994	0.0001361	0.0058109	0.0001072	0.1993215	0.0350331	2.7606120	0.0032607
032_VU107-J-2	13 °C	0.0091067	0.0001280	0.0126994	0.0001361	0.0058109	0.0001072	0.1993215	0.0350331	2.7606120	0.0032607
033_VU107-J-2	14 °C	0.0091067	0.0001280	0.0126994	0.0001361	0.0058109	0.0001072	0.1993215	0.0350331	2.7606120	0.0032607
035_VU107-J-2	15 °C	0.0095047	0.0001451	0.0126610	0.0000996	0.0061554	0.0001262	0.2345990	0.0214843	2.8611617	0.0054651
036_VU107-J-2	17 °C	0.0095047	0.0001451	0.0126610	0.0000996	0.0061554	0.0001262	0.2345990	0.0214843	2.8611617	0.0054651
037_VU107-J-2	18 °C	0.0095047	0.0001451	0.0126610	0.0000996	0.0061554	0.0001262	0.2345990	0.0214843	2.8611617	0.0054651
039_VU107-J-2	19 °C	0.0093501	0.0001023	0.0129290	0.0001113	0.0059677	0.0000629	0.2501847	0.0268808	2.7965944	0.0052220
040_VU107-J-2	21 °C	0.0093501	0.0001023	0.0129290	0.0001113	0.0059677	0.0000629	0.2501847	0.0268808	2.7965944	0.0052220
041_VU107-J-2	22 °C	0.0093501	0.0001023	0.0129290	0.0001113	0.0059677	0.0000629	0.2501847	0.0268808	2.7965944	0.0052220
043_VU107-J-2	25 °C	0.0095122	0.0000887	0.0126607	0.0001095	0.0060094	0.0000948	0.2023194	0.0469840	2.8118423	0.0054264
044_VU107-J-2	28 °C	0.0095122	0.0000887	0.0126607	0.0001095	0.0060094	0.0000948	0.2023194	0.0469840	2.8118423	0.0054264
045_VU107-J-2	31 °C	0.0095122	0.0000887	0.0126607	0.0001095	0.0060094	0.0000948	0.2023194	0.0469840	2.8118423	0.0054264
047_VU107-J-2	39 °C	0.0094910	0.0001415	0.0132192	0.0001467	0.0060789	0.0000943	0.2290960	0.0064235	2.7685235	0.0050313
048_VU107-J-2	69 °C	0.0094910	0.0001415	0.0132192	0.0001467	0.0060789	0.0000943	0.2290960	0.0064235	2.7685235	0.0050313
049_VU107-J-2	16 °C	0.0094910	0.0001415	0.0132192	0.0001467	0.0060789	0.0000943	0.2290960	0.0064235	2.7685235	0.0050313

Intercept Values		36Ar [fA]	1σ	r2		37Ar [fA]	1σ	r2		38Ar [fA]	1σ	r2		39Ar [fA]	1σ	r2		40Ar [fA]	1σ	r2	
031_VU107-J-2	12 °C	0.1067521	0.0002694	0.9500	LIN 15 of 15	0.1796	0.0005	0.9700	LIN 15 of 15	0.4768229	0.0010418	0.9900	LIN 15 of 15	40.29794	0.04735	1.0000	EXP 15 of 15	66.43665	0.03542	1.0000	EXP 15 of 15
032_VU107-J-2	13 °C	0.0634964	0.0003393	0.2700	LIN 15 of 15	0.3110	0.0009	0.9800	LIN 15 of 15	0.7160651	0.0015515	0.9900	LIN 15 of 15	62.27254	0.07512	1.0000	EXP 15 of 15	67.98495	0.03863	1.0000	EXP 14 of 15
033_VU107-J-2	14 °C	0.0570813	0.0003140	0.0500	LIN 15 of 15	0.4625	0.0009	0.9900	LIN 15 of 15	0.8811374	0.0016677	0.9900	LIN 15 of 15	76.33784	0.09258	1.0000	EXP 15 of 15	74.97356	0.04651	1.0000	EXP 15 of 15
035_VU107-J-2	15 °C	0.0573270	0.0003555	0.5400	LIN 15 of 15	0.5900	0.0009	0.9900	LIN 15 of 15	0.9102627	0.0014354	0.9900	LIN 15 of 15	78.40200	0.09441	1.0000	EXP 15 of 15	74.63875	0.04197	1.0000	EXP 15 of 15
036_VU107-J-2	17 °C	0.0534630	0.0002771	0.0200	LIN 15 of 15	0.5984	0.0013	0.9900	LIN 15 of 15	0.8233526	0.0016353	0.9900	LIN 15 of 15	70.15470	0.08615	1.0000	EXP 15 of 15	66.60514	0.03527	1.0000	EXP 15 of 15
037_VU107-J-2	18 °C	0.0461260	0.0003210	0.0000	LIN 15 of 15	0.4878	0.0011	0.9900	LIN 15 of 15	0.6607705	0.0012152	0.9900	LIN 15 of 15	56.30093	0.06800	1.0000	EXP 15 of 15	54.27068	0.03044	1.0000	EXP 15 of 15
039_VU107-J-2	19 °C	0.0449868	0.0002183	0.1900	LIN 15 of 15	0.4122	0.0006	0.9900	LIN 15 of 15	0.6232941	0.0011367	0.9900	LIN 15 of 15	52.47502	0.06237	1.0000	EXP 15 of 15	51.74103	0.03147	1.0000	EXP 15 of 15
040_VU107-J-2	21 °C	0.0439871	0.0002069	0.1800	LIN 15 of 15	0.3145	0.0010	0.9700	LIN 15 of 15	0.5548693	0.0009501	0.9900	LIN 15 of 15	45.75582	0.05408	1.0000	EXP 15 of 15	46.76999	0.02859	1.0000	EXP 15 of 15
041_VU107-J-2	22 °C	0.0404680	0.0002702	0.3500	LIN 15 of 15	0.2411	0.0007	0.9800	LIN 15 of 15	0.4383237	0.0010542	0.9800	LIN 15 of 15	35.19722	0.04289	1.0000	EXP 15 of 15	37.48366	0.02029	1.0000	EXP 15 of 15
043_VU107-J-2	25 °C	0.0453258	0.0003479	0.3900	LIN 15 of 15	0.3009	0.0009	0.9800	LIN 15 of 15	0.3977187	0.0009386	0.9800	LIN 15 of 15	30.87670	0.04068	1.0000	EXP 15 of 15	34.03932	0.02752	1.0000	EXP 15 of 15
044_VU107-J-2	28 °C	0.0505463	0.0003391	0.7600	LIN 15 of 15	0.4099	0.0007	0.9900	LIN 15 of 15	0.3153845	0.0006634	0.9900	LIN 15 of 15	23.87151	0.02912	1.0000	EXP 15 of 15	27.94966	0.01738	1.0000	EXP 15 of 15
045_VU107-J-2	31 °C	0.0541803	0.0003728	0.2500	LIN 14 of 15	0.5151	0.0011	0.9800	LIN 15 of 15	0.2093580	0.0006564	0.9600	LIN 15 of 15	15.01728	0.02579	0.9900	EXP 14 of 15	21.32070	0.01421	0.9900	EXP 14 of 15
047_VU107-J-2	39 °C	0.0782799	0.0003986	0.8600	LIN 15 of 15	0.7866	0.0015	0.9900	LIN 15 of 15	0.1740520	0.0007840	0.9200	LIN 15 of 15	12.17870	0.02615	0.9900	EXP 15 of 15	22.55732	0.01463	1.0000	EXP 15 of 15
048_VU107-J-2	69 °C	0.1611895	0.0004140	0.9700	LIN 15 of 15	1.0450	0.0023	0.9900	LIN 15 of 15	0.3189453	0.0007192	0.9800	LIN 15 of 15	24.08741	0.03106	1.0000	EXP 15 of 15	57.81091	0.03758	1.0000	EXP 15 of 15
049_VU107-J-2	16 °C	0.0619730	0.0004036	0.6700	LIN 15 of 15	0.8901	0.0012	1.0000	LIN 15 of 15	0.0729980	0.0004045	0.8300	LIN 15 of 15	4.55297	0.02462	0.9000	EXP 15 of 15	9.86873	0.00839	0.9900	EXP 15 of 15

Sample Parameters		Sample	Material	Location	Analyst	Temp	Standard (in Ma)	%1σ	J	%1σ	MDF	%1σ	Volume Ratio	Sensitivity (mol/volt)	Day	Month	Year	Hour	Min	Resist	Irradiation	Project	Experiment	Nmb	Standard Name
031_VU107-J-2	12 °C	031_VU107-J-2	groundmass	MW14 DL2-3	Klaudia Kuiper	11.7	28.201	0.08	0.0046897	0.1	0.983438	0.1	1	1E-13	18	NOV	2016	9	39	1	VU107	VU107	VU107-J-2	01	FCs
032_VU107-J-2	13 °C	032_VU107-J-2	groundmass	MW14 DL2-3	Klaudia Kuiper	13.1	28.201	0.08	0.0046897	0.1	0.983438	0.1	1	1E-13	18	NOV	2016	10	10	1	VU107	VU107	VU107-J-2	01	FCs
033_VU107-J-2	14 °C	033_VU107-J-2	groundmass	MW14 DL2-3	Klaudia Kuiper	14.3	28.201	0.08	0.0046897	0.1	0.983438	0.1	1	1E-13	18	NOV	2016	10	41	1	VU107	VU107	VU107-J-2	01	FCs
035_VU107-J-2	15 °C	035_VU107-J-2	groundmass	MW14 DL2-3	Klaudia Kuiper	15.3	28.201	0.08	0.0046897	0.1	0.983438	0.1	1	1E-13	18	NOV	2016	11	40	1	VU107	VU107	VU107-J-2	01	FCs
036_VU107-J-2	17 °C	036_VU107-J-2	groundmass	MW14 DL2-3	Klaudia Kuiper	16.5	28.201	0.08	0.0046897	0.1	0.983438	0.1	1	1E-13	18	NOV	2016	12	11	1	VU107	VU107	VU107-J-2	01	FCs
037_VU107-J-2	18 °C	037_VU107-J-2	groundmass	MW14 DL2-3	Klaudia Kuiper	17.6	28.201	0.08	0.0046897	0.1	0.983438	0.1	1	1E-13	18	NOV	2016	12	42	1	VU107	VU107	VU107-J-2	01	FCs
039_VU107-J-2	19 °C	039_VU107-J-2	groundmass	MW14 DL2-3	Klaudia Kuiper	18.9	28.201	0.08	0.0046897	0.1	0.983438	0.1	1	1E-13	18	NOV	2016	13	42	1	VU107	VU107	VU107-J-2	01	FCs
040_VU107-J-2	21 °C	040_VU107-J-2	groundmass	MW14 DL2-3	Klaudia Kuiper	20.5	28.201	0.08	0.0046897	0.1	0.983438	0.1	1	1E-13	18	NOV	2016	14	12	1	VU107	VU107	VU107-J-2	01	FCs
041_VU107-J-2	22 °C	041_VU107-J-2	groundmass	MW14 DL2-3	Klaudia Kuiper	22.4	28.201	0.08	0.0046897	0.1	0.983438	0.1	1	1E-13	18	NOV	2016	14	43	1	VU107	VU107	VU107-J-2	01	FCs
043_VU107-J-2	25 °C	043_VU107-J-2	groundmass	MW14 DL2-3	Klaudia Kuiper	24.6	28.201	0.08	0.0046897	0.1	0.983438	0.1	1	1E-13	18	NOV	2016	15	43	1	VU107	VU107	VU107-J-2	01	FCs
044_VU107-J-2	28 °C	044_VU107-J-2	groundmass	MW14 DL2-3	Klaudia Kuiper	27.6	28.201	0.08	0.0046897	0.1	0.983438	0.1	1	1E-13	18	NOV	2016	16	14	1	VU107	VU107	VU107-J-2	01	FCs
045_VU107-J-2	31 °C	045_VU107-J-2	groundmass	MW14 DL2-3	Klaudia Kuiper	31.2	28.201	0.08	0.0046897	0.1	0.983438	0.1	1	1E-13	18	NOV	2016	16	45	1	VU107	VU107	VU107-J-2	01	FCs
047_VU107-J-2	39 °C	047_VU107-J-2	groundmass	MW14 DL2-3	Klaudia Kuiper	38.7	28.201	0.08	0.0046897	0.1	0.983438	0.1	1	1E-13	18	NOV	2016	17	44	1	VU107	VU107	VU107-J-2	01	FCs
048_VU107-J-2	69 °C	048_VU107-J-2	groundmass	MW14 DL2-3	Klaudia Kuiper	68.6	28.201	0.08	0.0046897	0.1	0.983438	0.1	1	1E-13	18	NOV	2016	18	15	1	VU107	VU107	VU107-J-2	01	FCs
049_VU107-J-2	16 °C	049_VU107-J-2	groundmass	MW14 DL2-3	Klaudia Kuiper	16	28.201	0.08	0.0046897	0.1	0.983438	0.1	1	1E-13	18	NOV	2016	18	46	1	VU107	VU107	VU107-J-2	01	FCs

Irradiation Constants		40/36(a)	%1σ	40/36(c)	%1σ	38/36(a)	%1σ	38/36(c)	%1σ	39/37(ca)	%1σ	38/37(ca)	%1σ	36/37(ca)	%1σ	40/39(k)	%1σ	38/39(k)	%1σ	36/38(cl)	%1σ	K/Ca	%1σ	K/Cl	%1σ	Ca/Cl	%1σ
031_VU107-J-2	12 °C	298.56	0.104	0.018	35	0.1885	0.159	1.493	3	0.000673	0.55	0	0	0.000264	0.64	0.00086	8.1	0.01211	0.25	0	0	0.43	0	0	0	0	0
032_VU107-J-2	13 °C	298.56	0.104	0.018	35	0.1885	0.159	1.493	3	0.000673	0.55	0	0	0.000264	0.64	0.00086	8.1	0.01211	0.25	0	0	0.43	0	0	0	0	0
033_VU107-J-2	14 °C	298.56	0.104	0.018	35	0.1885	0.159	1.493	3	0.000673	0.55	0	0	0.000264	0.64	0.00086	8.1	0.01211	0.25	0	0	0.43	0	0	0	0	0
035_VU107-J-2	15 °C	298.56	0.104	0.018	35	0.1885	0.159	1.493	3	0.000673	0.55	0	0	0.000264	0.64	0.00086	8.1	0.01211	0.25	0	0	0.43	0	0	0	0	0
036_VU107-J-2	17 °C	298.56	0.104	0.018	35	0.1885	0.159	1.493	3	0.000673	0.55	0	0	0.000264	0.64	0.00086	8.1	0.01211	0.25	0	0	0.43	0	0	0	0	0
037_VU107-J-2	18 °C	298.56	0.104	0.018	35	0.1885	0.159	1.493	3	0.000673	0.55	0	0	0.000264	0.64	0.00086	8.1	0.01211	0.25	0	0	0.43	0	0	0	0	0
039_VU107-J-2	19 °C	298.56	0.104	0.018	35	0.1885	0.159	1.493	3	0.000673	0.55	0	0	0.000264	0.64	0.00086	8.1	0.01211	0.25	0	0	0.43	0	0	0	0	0
040_VU107-J-2	21 °C	298.56	0.104	0.018	35	0.1885	0.159	1.493	3	0.000673	0.55	0	0	0.000264	0.64	0.00086	8.1	0.01211	0.25	0	0	0.43	0	0	0	0	0
041_VU107-J-2	22 °C	298.56	0.104	0.018	35	0.1885	0.159	1.493	3	0.000673	0.55	0	0	0.000264	0.64	0.00086	8.1	0.01211	0.25	0	0	0.43	0	0	0	0	0
043_VU107-J-2	25 °C	298.56	0.104	0.018	35	0.1885	0.159	1.493	3	0.000673	0.55	0	0	0.000264	0.64	0.00086	8.1	0.01211	0.25	0	0	0.43	0	0	0	0	0
044_VU107-J-2	28 °C	298.56	0.104	0.018	35	0.1885	0.159	1.493	3	0.000673	0.55	0	0	0.000264	0.64	0.00086	8.1	0.01211	0.25	0	0	0.43	0	0	0	0	0
045_VU107-J-2	31 °C	298.56	0.104	0.018	35	0.1885	0.159	1.493	3	0.000673	0.55	0	0	0.000264	0.64	0.00086	8.1	0.01211	0.25	0	0	0.43	0	0	0	0	0
047_VU107-J-2	39 °C	298.56	0.104	0.018	35	0.1885	0.159	1.493	3	0.000673	0.55	0	0	0.000264	0.64	0.00086	8.1	0.01211	0.25	0	0	0.43	0	0	0	0	0
048_VU107-J-2	69 °C	298.56	0.104	0.018	35	0.1885	0.159	1.493	3	0.000673	0.55	0	0	0.000264	0.64	0.00086	8.1	0.01211	0.25	0	0	0.43	0	0	0	0	0
049_VU107-J-2	16 °C	298.56	0.104	0.018	35	0.1885	0.159	1.493	3	0.000673	0.55	0	0	0.000264	0.64	0.00086	8.1	0.01211	0.25	0	0	0.43	0	0	0	0	0

Geochronology laboratory
Vrije Universiteit Amsterdam, Amsterdam, The Netherlands

VU107-J2_1_CORR_MW14 DL2-3.AGE >>> 031_VU107-J-2 >>> VU107 PROJECT

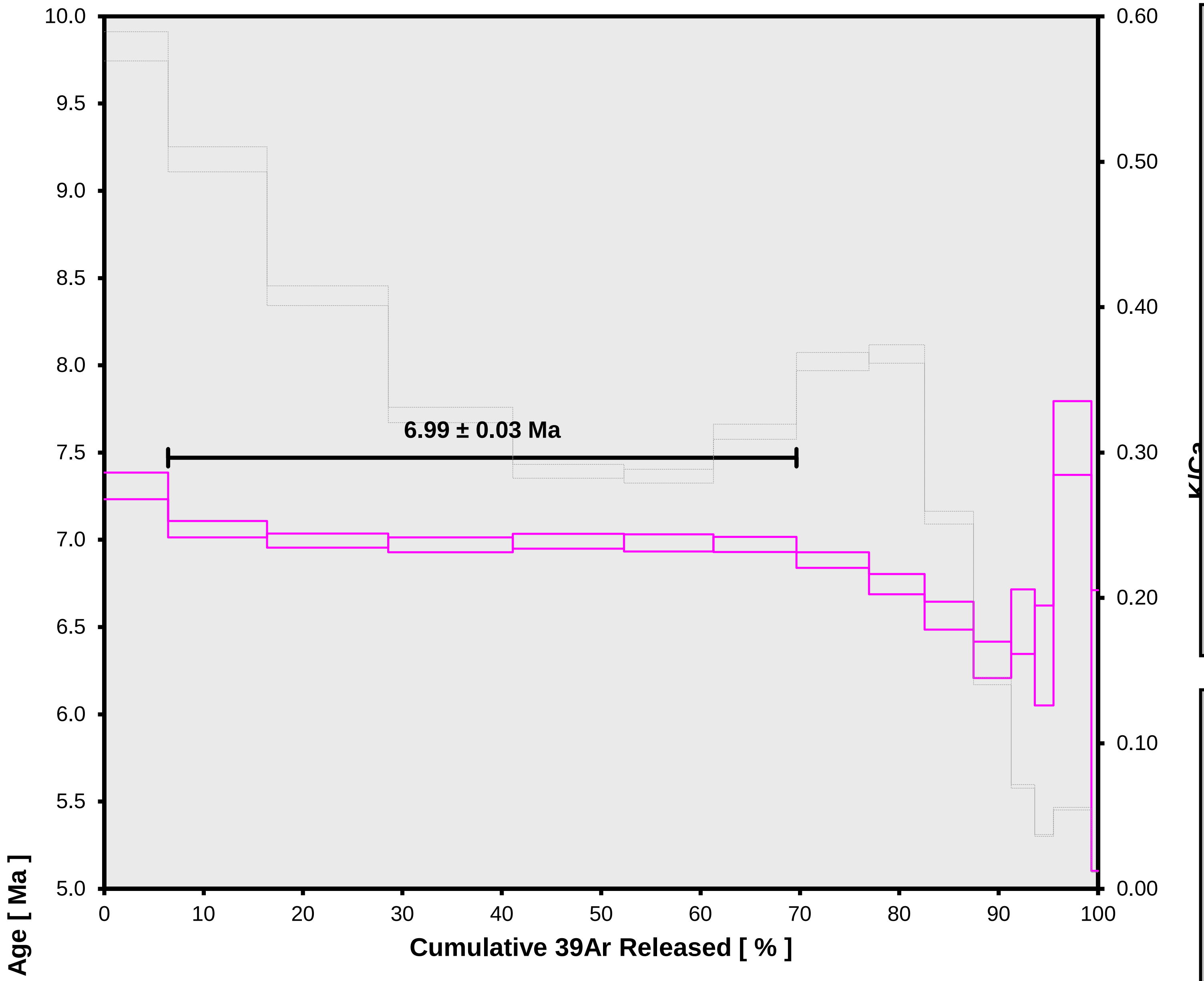

Ar-Ages in Ma

WEIGHTED PLATEAU
6.99 ± 0.03
TOTAL FUSION
6.94 ± 0.02
NORMAL ISOCHRON
6.92 ± 0.06
INVERSE ISOCHRON
6.92 ± 0.06

MSWD (PROBABILITY)
2.07 (7%)

Sample Info

groundmass
MW14 DL2-3
Klaudia Kuiper

IRR = VU107
J = 0.00468970 ± 0.00000469

RECALIBRATED AGE

VU107-J2_1_corr_MW14 DL2-3.full printed at 4/28/2018 (10:38 AM)
ArArCALC v2.5.2

Geochronology laboratory
Vrije Universiteit Amsterdam, Amsterdam, The Netherlands

VU107-J2_1_CORR_MW14 DL2-3.AGE >>> 031_VU107-J-2 >>> VU107 PROJECT

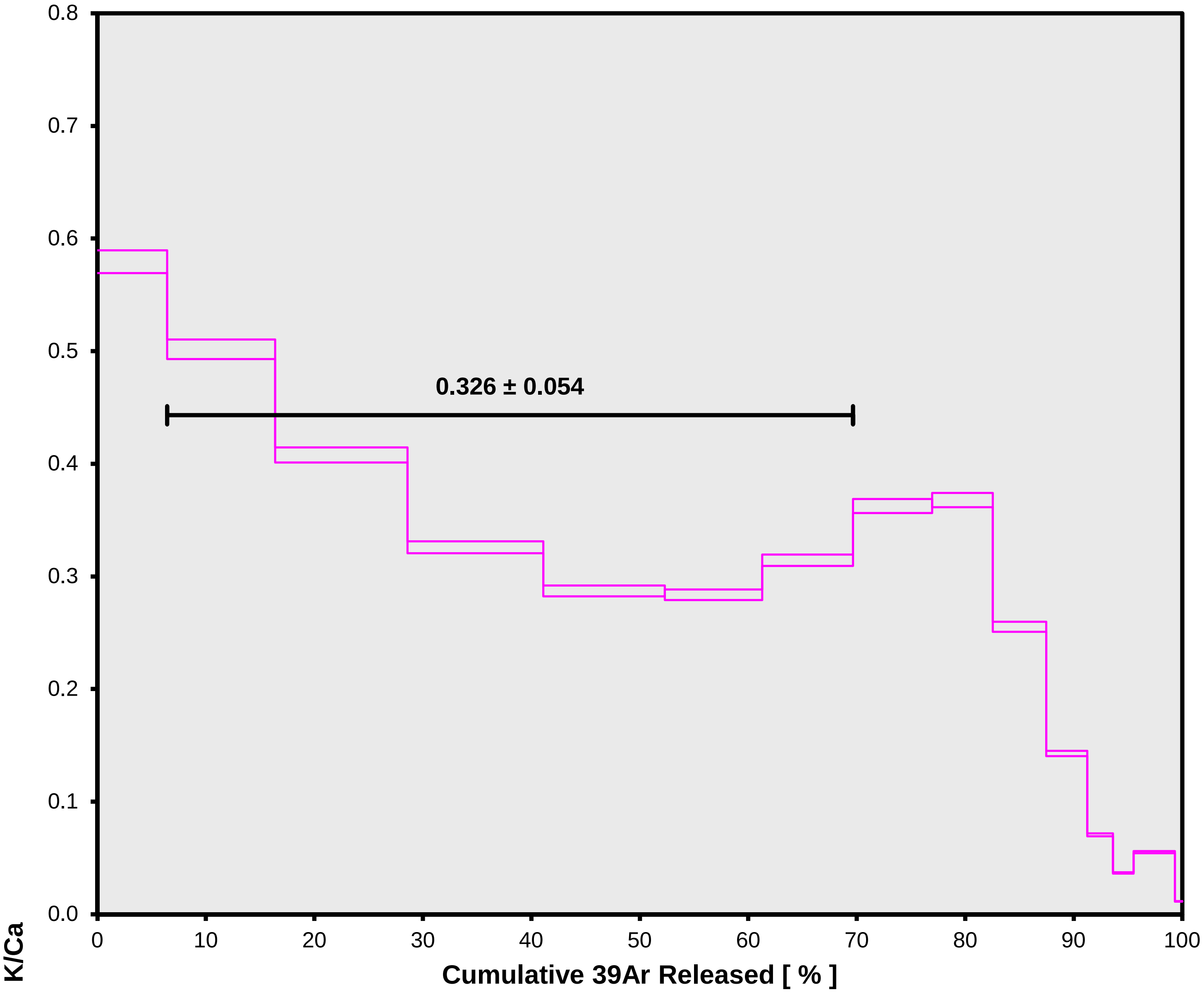

Ar-Ages in Ma

WEIGHTED PLATEAU
6.99 ± 0.03
TOTAL FUSION
6.94 ± 0.02
NORMAL ISOCHRON
6.92 ± 0.06
INVERSE ISOCHRON
6.92 ± 0.06

Sample Info

groundmass
MW14 DL2-3
Klaudia Kuiper

IRR = VU107
J = 0.00468970 ± 0.00000469

RECALIBRATED AGE

VU107-J2_1_corr_MW14 DL2-3.full printed at 4/28/2018 (10:38 AM)
ArArCALC v2.5.2

Geochronology laboratory
Vrije Universiteit Amsterdam, Amsterdam, The Netherlands

VU107-J2_1_CORR_MW14 DL2-3.AGE >>> 031_VU107-J-2 >>> VU107 PROJECT

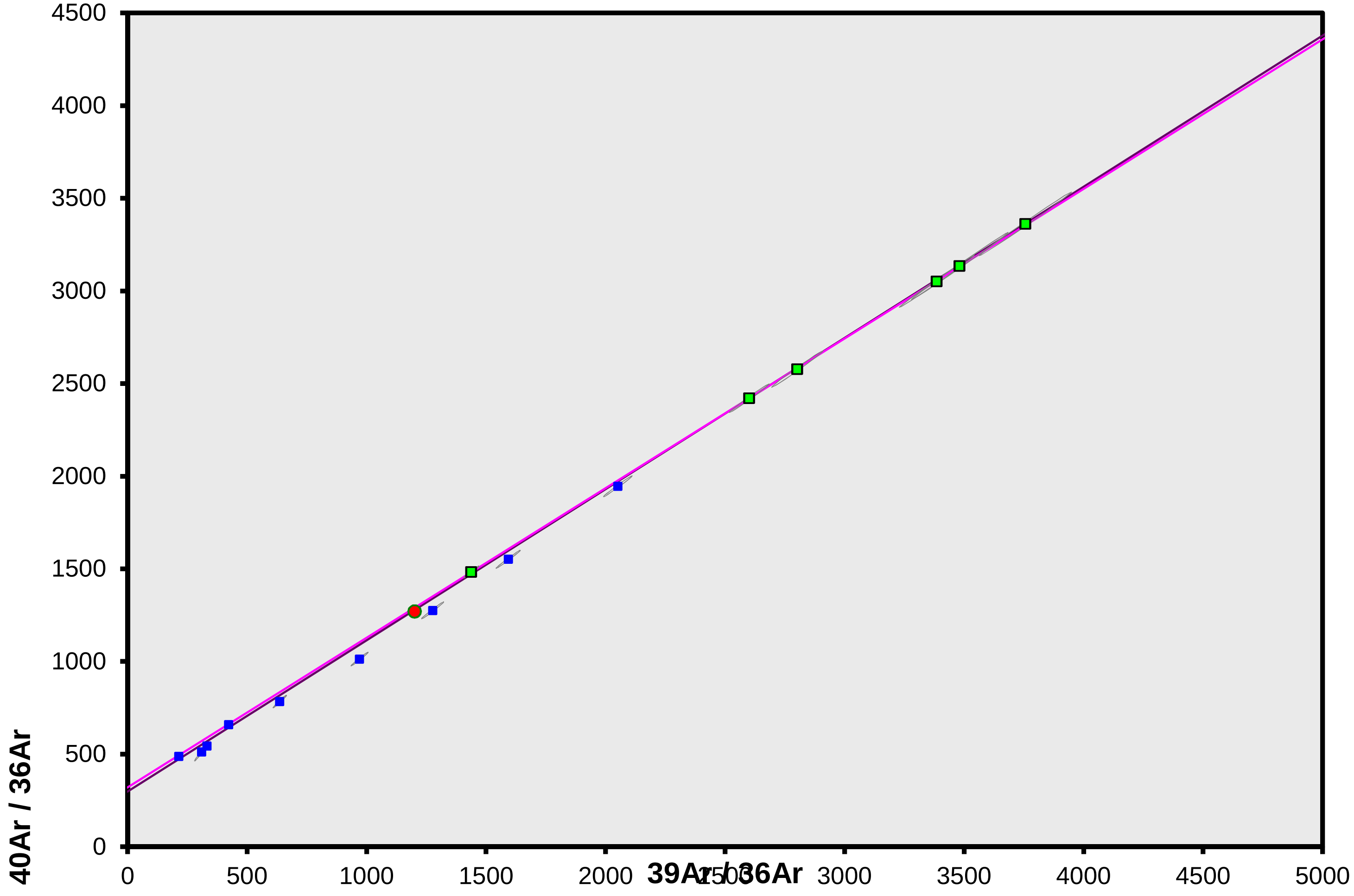

Ar-Ages in Ma

WEIGHTED PLATEAU
6.99 ± 0.03
TOTAL FUSION
6.94 ± 0.02
NORMAL ISOCHRON
6.92 ± 0.06
INVERSE ISOCHRON
6.92 ± 0.06

MSWD (PROBABILITY)
0.35 (84%)

40AR/36AR INTERCEPT
320.6 ± 15.8

Sample Info

groundmass
MW14 DL2-3
Klaudia Kuiper

IRR = VU107
J = 0.00468970 ± 0.00000469

RECALIBRATED AGE

VU107-J2_1_corr_MW14 DL2-3.full printed at 4/28/2018 (10:38 AM)
ArArCALC v2.5.2

Geochronology laboratory
Vrije Universiteit Amsterdam, Amsterdam, The Netherlands

VU107-J2_1_CORR_MW14 DL2-3.AGE >>> 031_VU107-J-2 >>> VU107 PROJECT

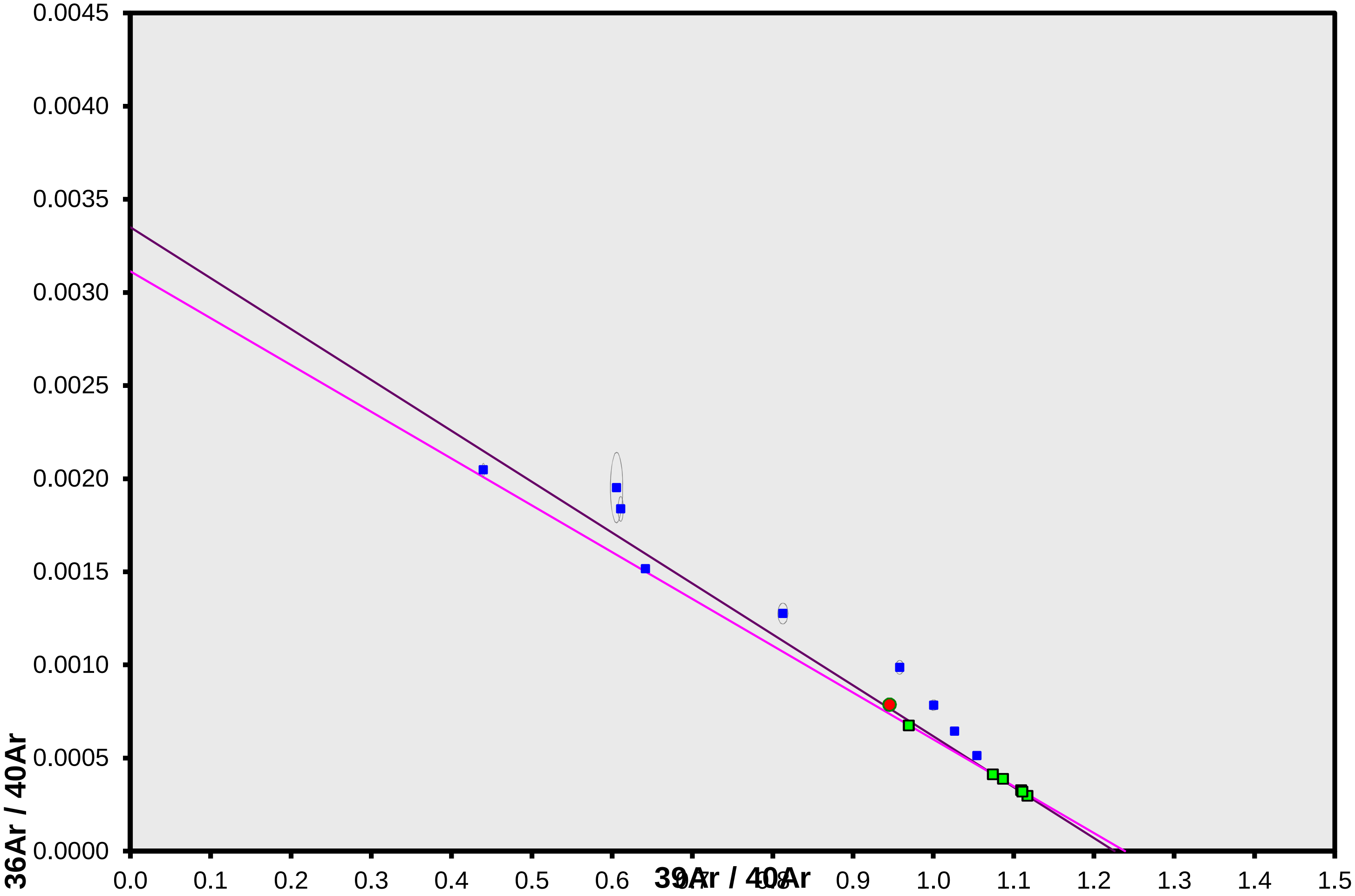

Ar-Ages in Ma

WEIGHTED PLATEAU
6.99 ± 0.03
TOTAL FUSION
6.94 ± 0.02
NORMAL ISOCHRON
6.92 ± 0.06
INVERSE ISOCHRON
6.92 ± 0.06

MSWD (PROBABILITY)
0.35 (84%)

SPREADING FACTOR
11.9%

40AR/36AR INTERCEPT
321.2 ± 15.8

Sample Info

groundmass
MW14 DL2-3
Klaudia Kuiper

IRR = VU107
J = 0.00468970 ± 0.00000469

RECALIBRATED AGE

VU107-J2_1_corr_MW14 DL2-3.full printed at 4/28/2018 (10:38 AM)
ArArCALC v2.5.2

**Relative Abundances**			36Ar [fA]	%1σ	37Ar [fA]	%1σ	38Ar [fA]	%1σ	39Ar [fA]	%1σ	40Ar [fA]	%1σ	40(r)/39(k) ± 2σ	Age ± 2σ (Ma)	40Ar(r) (%)	39Ar(k) (%)	K/Ca ± 2σ
17D18022	1.8 %	✓	0.1624643	0.617	73.9781	0.590	0.608230	3.935	49.91883	0.094	176.7300	0.134	2.52630 ± 0.02898	6.97 ± 0.08	71.29	6.16	0.290 ± 0.003
17D18024	1.9 %	✓	0.1040969	0.730	68.0583	0.613	0.539236	4.225	43.72542	0.100	140.7078	0.089	2.52055 ± 0.02144	6.95 ± 0.06	78.24	5.40	0.276 ± 0.003
17D18025	2.0 %	✓	0.0457268	0.994	37.6991	0.915	0.274794	8.872	22.73250	0.155	70.1696	0.088	2.52984 ± 0.02140	6.98 ± 0.06	81.87	2.81	0.259 ± 0.005
17D18027	2.2 %	✓	0.0371694	1.166	35.2333	0.992	0.258602	9.339	19.65543	0.172	59.5487	0.070	2.52831 ± 0.02185	6.97 ± 0.06	83.35	2.43	0.240 ± 0.005
17D18028	2.4 %	✓	0.0192018	1.825	19.1825	1.589	0.122978	19.029	10.52720	0.306	31.4786	0.114	2.51541 ± 0.03140	6.94 ± 0.09	84.02	1.30	0.236 ± 0.008
17D18030	2.7 %	✓	0.1056581	0.610	126.1494	0.485	0.776155	3.101	63.92361	0.085	187.2932	0.029	2.53029 ± 0.01254	6.98 ± 0.03	86.24	7.89	0.218 ± 0.002
17D18031	3.0 %	✓	0.0835332	0.689	119.2121	0.490	0.632168	3.601	53.04518	0.091	152.7419	0.047	2.53378 ± 0.01240	6.99 ± 0.03	87.86	6.55	0.191 ± 0.002
17D18033	3.4 %	✓	0.0750167	0.777	118.3012	0.494	0.540130	4.309	47.31766	0.096	134.8214	0.043	2.52408 ± 0.01288	6.96 ± 0.04	88.44	5.84	0.172 ± 0.002
17D18034	3.9 %	✓	0.0915798	0.696	153.2515	0.460	0.682610	3.488	56.50007	0.090	160.5568	0.032	2.52432 ± 0.01208	6.96 ± 0.03	88.67	6.97	0.158 ± 0.001
17D18036	4.5 %	✓	0.0971148	0.688	171.2963	0.447	0.701008	3.606	58.21056	0.088	164.9569	0.030	2.52171 ± 0.01208	6.96 ± 0.03	88.81	7.18	0.146 ± 0.001
17D18037	5.2 %	✓	0.0676602	0.805	120.3202	0.496	0.469318	4.952	38.86566	0.106	110.5660	0.048	2.52175 ± 0.01406	6.96 ± 0.04	88.46	4.79	0.139 ± 0.001
17D18039	6.0 %		0.1069866	0.672	169.3005	0.451	0.722722	3.285	57.81366	0.087	166.1890	0.030	2.49653 ± 0.01341	6.89 ± 0.04	86.68	7.13	0.147 ± 0.001
17D18040	6.9 %		0.1004400	0.618	142.1516	0.472	0.677882	3.468	51.60066	0.092	149.7375	0.039	2.47319 ± 0.01412	6.82 ± 0.04	85.07	6.37	0.156 ± 0.001
17D18042	7.9 %		0.1334753	0.579	169.1061	0.452	0.839834	2.820	62.28662	0.085	181.2544	0.028	2.40785 ± 0.01521	6.64 ± 0.04	82.59	7.68	0.158 ± 0.001
17D18043	9.0 %		0.1247253	0.615	162.8677	0.454	0.659964	3.605	47.46227	0.096	138.5660	0.044	2.31416 ± 0.01886	6.38 ± 0.05	79.08	5.85	0.125 ± 0.001
17D18045	10.3 %		0.1152054	0.578	166.3676	0.455	0.512328	4.216	34.65139	0.114	101.1154	0.044	2.19696 ± 0.02247	6.06 ± 0.06	75.04	4.27	0.089 ± 0.001
17D18046	11.6 %		0.1383861	0.529	239.8162	0.434	0.441190	5.259	30.72748	0.122	89.1621	0.047	2.04997 ± 0.02728	5.66 ± 0.08	70.27	3.78	0.055 ± 0.000
17D18047	12.5 %		0.0993283	0.629	184.8795	0.447	0.299199	8.295	19.18914	0.172	55.5763	0.063	1.98105 ± 0.03337	5.47 ± 0.09	67.96	2.36	0.044 ± 0.000
17D18049	13.4 %		0.0523424	0.934	103.8895	0.506	0.109563	20.554	9.60362	0.333	27.4828	0.123	1.96235 ± 0.04567	5.42 ± 0.13	68.07	1.18	0.039 ± 0.000
17D18050	14.6 %		0.0409968	1.039	77.5181	0.589	0.089869	26.162	7.55922	0.414	21.6472	0.158	1.91969 ± 0.05060	5.30 ± 0.14	66.57	0.93	0.042 ± 0.001
17D18051	16.0 %		0.0552012	0.846	116.6672	0.495	0.162291	14.874	7.77889	0.406	22.8480	0.149	1.85411 ± 0.05475	5.12 ± 0.15	62.49	0.95	0.028 ± 0.000
17D18053	17.6 %		0.0714004	0.755	159.2897	0.459	0.124813	18.791	8.84608	0.355	26.8391	0.126	1.89312 ± 0.05595	5.22 ± 0.15	61.64	1.08	0.024 ± 0.000
17D18054	19.3 %		0.0489501	0.920	109.8970	0.504	0.070183	33.321	5.54799	0.564	17.2544	0.201	1.87491 ± 0.07236	5.17 ± 0.20	59.48	0.68	0.021 ± 0.000
17D18056	21.0 %		0.0299011	1.280	63.0505	0.641	0.012647	196.219	3.54256	0.875	11.3873	0.305	1.92320 ± 0.09457	5.31 ± 0.26	59.11	0.43	0.024 ± 0.001
		Σ	2.0065609	0.150	2907.4832	0.106	10.327714	1.122	811.03168	0.025	2398.6303	0.015					

Information on Analysis and Constants Used in Calculations

Project = **O-CONNOR (16-23)**
Sample = **MW14-DL2-2**
Material = **Groundmass**
Location = **Mozambique Ridge**
Region = **Indian Ocean**
Analyst = **Dan Miggins**
Irradiation = **17-OSU-01 (1B38-17)**
Position = **X: 0 | Y: 0 | Z/H: 59.89021 mm**
FCT-NM Age = **28.201 ± 0.023 Ma**
FCT-NM Reference = **Kuiper et al (2008)**
FCT-NM 40Ar/39Ar Ratio = **10.28448 ± 0.01018**
FCT-NM J-value = **0.00152826 ± 0.00000151**
Air Shot 40Ar/36Ar = **302.7200 ± 0.4268**
Air Shot MDF = **0.99402969 ± 0.00067386 (LIN)**
Experiment Type = **Incremental Heating**
Extraction Method = **Bulk Laser Heating**
Heating = **77 sec**
Isolation = **3.00 min**
Instrument = **ARGUS-VI-D**
Preferred Age = **Plateau Age**
Age Classification = **Crystallization Age**
IGSN = Undefined
Rock Class = Undefined
Lithology = Undefined
Lat-Lon = Undefined - Undefined

Age Equations = **Min et al. (2000)**
Negative Intensities = **Allowed**
Collector Calibrations = **36Ar**
Decay 40K = **5.530 ± 0.048 E-10 1/a**
Decay 39Ar = **2.940 ± 0.016 E-07 1/h**
Decay 37Ar = **8.230 ± 0.012 E-04 1/h**
Decay 36Cl = **2.257 ± 0.015 E-06 1/a**
Decay 40K(EC,β^+) = **0.580 ± 0.009 E-10 1/a**
Decay 40K(β^-) = **4.950 ± 0.043 E-10 1/a**
Atmospheric 40/36(a) = **354.12 ± 3.99**
Atmospheric 38/36(a) = **0.1869**
Production 39/37(ca) = **0.0006756 ± 0.0000089**
Production 38/37(ca) = **0.0000718 ± 0.0000092**
Production 36/37(ca) = **0.0002663 ± 0.0000004**
Production 40/39(k) = **0.003823 ± 0.000102**
Production 38/39(k) = **0.012031 ± 0.000019**
Production 36/38(cl) = **262.80 ± 1.71**
Scaling Ratio K/Ca = **0.430**
Abundance Ratio 40K/K = **1.1700 ± 0.0100 E-04**
Atomic Weight K = **39.0983 ± 0.0001 g**

Results

Results	40(a)/36(a) ± 2σ		40(r)/39(k) ± 2σ		Age ± 2σ (Ma)		MSWD	39Ar(k) (%,n)	K/Ca ± 2σ
**Age Plateau**			2.52589	± 0.00465<br>± 0.18%	**6.97**	**± 0.02**<br>**± 0.27%**	0.40<br>95%	57.32<br>11	0.173 ± 0.025
					Full External Error	± 0.16	1.89	2σ Confidence Limit	
					Analytical Error	± 0.01	1.0000	Error Magnification	
**Total Fusion Age**			2.42154	± 0.00424<br>± 0.18%	**6.68**	**± 0.02**<br>**± 0.26%**		24	0.120 ± 0.000
					Full External Error	± 0.15			
					Analytical Error	± 0.01			
**Normal Isochron**	**354.70**	**± 7.97**<br>**± 2.25%**	2.52532	± 0.00989<br>± 0.39%	**6.97**	**± 0.03**<br>**± 0.44%**	0.71<br>70%	57.32<br>11	
					Full External Error	± 0.16	1.94	2σ Confidence Limit	
					Analytical Error	± 0.03	1.0000	Error Magnification	
							20	Number of Iterations	
							0.0000249057	Convergence	
**Inverse Isochron**	**354.12**	**± 7.97**<br>**± 2.25%**	2.52619	± 0.00990<br>± 0.39%	**6.97**	**± 0.03**<br>**± 0.44%**	0.71<br>70%	57.32<br>11	
					Full External Error	± 0.16	1.94	2σ Confidence Limit	
					Analytical Error	± 0.03	1.0000	Error Magnification	
							2	Number of Iterations	
							0.0003231990	Convergence	
							18%	Spreading Factor	

Notes
Excess Initial 40Ar/36Ar = 354.12 ± 1.13 (%SD).

**Incremental Heating**			36Ar(a) [fA]	37Ar(ca) [fA]	38Ar(cl) [fA]	39Ar(k) [fA]	40Ar(r) [fA]	Age ± 2σ (Ma)	40Ar(r) (%)	39Ar(k) (%)	K/Ca ± 2σ
17D18022	1.8 %	✓	0.1427640	73.9781	0.0000000	49.86885	125.9838	6.97 ± 0.08	71.29	6.16	0.290 ± 0.003
17D18024	1.9 %	✓	0.0859730	68.0583	0.0000000	43.67944	110.0961	6.95 ± 0.06	78.24	5.40	0.276 ± 0.003
17D18025	2.0 %	✓	0.0356875	37.6991	0.0000000	22.70703	57.4452	6.98 ± 0.06	81.87	2.81	0.259 ± 0.005
17D18027	2.2 %	✓	0.0277838	35.2333	0.0146915	19.63163	49.6348	6.97 ± 0.06	83.35	2.43	0.240 ± 0.005
17D18028	2.4 %	✓	0.0140935	19.1825	0.0000000	10.51424	26.4476	6.94 ± 0.09	84.02	1.30	0.236 ± 0.008
17D18030	2.7 %	✓	0.0720646	126.1494	0.0000000	63.83838	161.5297	6.98 ± 0.03	86.24	7.89	0.218 ± 0.002
17D18031	3.0 %	✓	0.0517870	119.2121	0.0000000	52.96464	134.2006	6.99 ± 0.03	87.86	6.55	0.191 ± 0.002
17D18033	3.4 %	✓	0.0435131	118.3012	0.0000000	47.23773	119.2319	6.96 ± 0.04	88.44	5.84	0.172 ± 0.002
17D18034	3.9 %	✓	0.0507689	153.2515	0.0000000	56.39653	142.3629	6.96 ± 0.03	88.67	6.97	0.158 ± 0.001
17D18036	4.5 %	✓	0.0514986	171.2963	0.0000000	58.09483	146.4981	6.96 ± 0.03	88.81	7.18	0.146 ± 0.001
17D18037	5.2 %	✓	0.0356190	120.3202	0.0000000	38.78437	97.8044	6.96 ± 0.04	88.46	4.79	0.139 ± 0.001
17D18039	6.0 %		0.0619009	169.3005	0.0048166	57.69928	144.0481	6.89 ± 0.04	86.68	7.13	0.147 ± 0.001
17D18040	6.9 %		0.0625776	142.1516	0.0363279	51.50462	127.3806	6.82 ± 0.04	85.07	6.37	0.156 ± 0.001
17D18042	7.9 %		0.0884294	169.1061	0.0631686	62.17237	149.7020	6.64 ± 0.04	82.59	7.68	0.158 ± 0.001
17D18043	9.0 %		0.0813406	162.8677	0.0633731	47.35223	109.5806	6.38 ± 0.05	79.08	5.85	0.125 ± 0.001
17D18045	10.3 %		0.0708870	166.3676	0.0715957	34.53899	75.8809	6.06 ± 0.06	75.04	4.27	0.089 ± 0.001
17D18046	11.6 %		0.0745144	239.8162	0.0423116	30.56546	62.6582	5.66 ± 0.08	70.27	3.78	0.055 ± 0.000
17D18047	12.5 %		0.0500852	184.8795	0.0472020	19.06424	37.7672	5.47 ± 0.09	67.96	2.36	0.044 ± 0.000
17D18049	13.4 %		0.0246766	103.8895	0.0000000	9.53343	18.7079	5.42 ± 0.13	68.07	1.18	0.039 ± 0.000
17D18050	14.6 %		0.0203537	77.5181	0.0000000	7.50684	14.4108	5.30 ± 0.14	66.57	0.93	0.042 ± 0.001
17D18051	16.0 %		0.0241211	116.6672	0.0567660	7.70007	14.2768	5.12 ± 0.15	62.49	0.95	0.028 ± 0.000
17D18053	17.6 %		0.0289810	159.2897	0.0028274	8.73846	16.5429	5.22 ± 0.15	61.64	1.08	0.024 ± 0.000
17D18054	19.3 %		0.0196845	109.8970	0.0000000	5.47375	10.2628	5.17 ± 0.20	59.48	0.68	0.021 ± 0.000
17D18056	21.0 %		0.0131107	63.0505	0.0000000	3.49996	6.7311	5.31 ± 0.26	59.11	0.43	0.024 ± 0.001
		Σ	1.2322155	2907.4832	0.4030805	809.06738	1959.1851				

Information on Analysis

Project = **O-CONNOR (16-23)**
Sample = **MW14-DL2-2**
Material = **Groundmass**
Location = **Mozambique Ridge**
Region = **Indian Ocean**
Analyst = **Dan Miggins**
Irradiation = **17-OSU-01 (1B38-17)**
J = **0.00152826 ± 0.00000151**
FCT-NM = **28.201 ± 0.023 Ma**

**Results**	40(r)/39(k) ± 2σ		Age ± 2σ (Ma)		MSWD	39Ar(k) (%,n)	K/Ca ± 2σ
**Age Plateau**	2.52589	± 0.00465<br>± 0.18%	**6.97**	**± 0.02**<br>**± 0.27%**	0.40<br>95%	57.32<br>11	0.173 ± 0.025
		Full External Error		± 0.16	1.89	2σ Confidence Limit	
		Analytical Error		± 0.01	1.0000	Error Magnification	
**Total Fusion Age**	2.42154	± 0.00424<br>± 0.18%	**6.68**	**± 0.02**<br>**± 0.26%**		24	0.120 ± 0.000
		Full External Error		± 0.15			
		Analytical Error		± 0.01			

Normal Isochron			39(k)/36(a) ± 2σ	40(a+r)/36(a) ± 2σ	r.i.
17D18022	1.8 %	✓	349.31 ± 4.99	1236.58 ± 17.81	0.9740
17D18024	1.9 %	✓	508.06 ± 9.14	1634.71 ± 29.38	0.9889
17D18025	2.0 %	✓	636.27 ± 16.67	1963.79 ± 51.20	0.9907
17D18027	2.2 %	✓	706.59 ± 22.70	2140.59 ± 68.43	0.9933
17D18028	2.4 %	✓	746.03 ± 38.36	2230.70 ± 114.00	0.9919
17D18030	2.7 %	✓	885.85 ± 16.46	2595.58 ± 48.04	0.9953
17D18031	3.0 %	✓	1022.74 ± 23.70	2945.52 ± 68.11	0.9960
17D18033	3.4 %	✓	1085.60 ± 30.25	3094.26 ± 86.06	0.9971
17D18034	3.9 %	✓	1110.85 ± 29.27	3158.25 ± 83.05	0.9973
17D18036	4.5 %	✓	1128.09 ± 30.82	3198.82 ± 87.23	0.9977
17D18037	5.2 %	✓	1088.87 ± 34.87	3099.97 ± 99.11	0.9974
17D18039	6.0 %		932.12 ± 22.65	2681.20 ± 65.01	0.9971
17D18040	6.9 %		823.05 ± 17.14	2389.68 ± 49.59	0.9954
17D18042	7.9 %		703.07 ± 12.80	2047.02 ± 37.12	0.9952
17D18043	9.0 %		582.15 ± 11.43	1701.30 ± 33.29	0.9942
17D18045	10.3 %		487.24 ± 9.67	1424.57 ± 28.10	0.9923
17D18046	11.6 %		410.20 ± 8.74	1195.01 ± 25.30	0.9924
17D18047	12.5 %		380.64 ± 10.22	1108.18 ± 29.54	0.9905
17D18049	13.4 %		386.34 ± 16.18	1112.24 ± 46.07	0.9853
17D18050	14.6 %		368.82 ± 16.38	1062.14 ± 46.44	0.9796
17D18051	16.0 %		319.23 ± 13.33	946.00 ± 38.84	0.9779
17D18053	17.6 %		301.52 ± 12.20	924.94 ± 36.89	0.9821
17D18054	19.3 %		278.07 ± 13.82	875.48 ± 42.48	0.9698
17D18056	21.0 %		266.95 ± 16.90	867.53 ± 52.98	0.9552

Results	40(a)/36(a) ± 2σ	40(r)/39(k) ± 2σ	Age ± 2σ (Ma)	MSWD
Normal Isochron	**354.70** **± 7.97** **± 2.25%**	2.52532 ± 0.00989 ± 0.39%	**6.97** **± 0.03** **± 0.44%**	0.71 70%
			Full External Error ± 0.16	
			Analytical Error ± 0.03	

Statistics					
	2σ Confidence Limit	1.94	Convergence	0.000024905704
	Error Magnification	1.0000	Number of Iterations	20
	Number of Data Points	11	Calculated Line	Weighted York-2

Inverse Isochron			39(k)/40(a+r) ± 2σ	36(a)/40(a+r) ± 2σ	r.i.
17D18022	1.8 %	✓	0.2824800 ± 0.0009243	0.00080868 ± 0.00001165	0.1524
17D18024	1.9 %	✓	0.3107953 ± 0.0008311	0.00061173 ± 0.00001099	0.0656
17D18025	2.0 %	✓	0.3240027 ± 0.0011567	0.00050922 ± 0.00001328	0.0337
17D18027	2.2 %	✓	0.3300896 ± 0.0012278	0.00046716 ± 0.00001493	0.0164
17D18028	2.4 %	✓	0.3344392 ± 0.0021882	0.00044829 ± 0.00002291	0.0156
17D18030	2.7 %	✓	0.3412920 ± 0.0006139	0.00038527 ± 0.00000713	0.0102
17D18031	3.0 %	✓	0.3472194 ± 0.0007147	0.00033950 ± 0.00000785	0.0186
17D18033	3.4 %	✓	0.3508426 ± 0.0007416	0.00032318 ± 0.00000899	0.0128
17D18034	3.9 %	✓	0.3517282 ± 0.0006748	0.00031663 ± 0.00000833	0.0083
17D18036	4.5 %	✓	0.3526567 ± 0.0006562	0.00031261 ± 0.00000852	0.0072
17D18037	5.2 %	✓	0.3512512 ± 0.0008158	0.00032258 ± 0.00001031	0.0123
17D18039	6.0 %		0.3476521 ± 0.0006401	0.00037297 ± 0.00000904	0.0080
17D18040	6.9 %		0.3444191 ± 0.0006901	0.00041847 ± 0.00000868	0.0147
17D18042	7.9 %		0.3434621 ± 0.0006142	0.00048852 ± 0.00000886	0.0097
17D18043	9.0 %		0.3421776 ± 0.0007241	0.00058779 ± 0.00001150	0.0191
17D18045	10.3 %		0.3420266 ± 0.0008381	0.00070197 ± 0.00001385	0.0161
17D18046	11.6 %		0.3432577 ± 0.0009008	0.00083681 ± 0.00001772	0.0158
17D18047	12.5 %		0.3434789 ± 0.0012670	0.00090238 ± 0.00002406	0.0163
17D18049	13.4 %		0.3473474 ± 0.0024829	0.00089908 ± 0.00003724	0.0207
17D18050	14.6 %		0.3472424 ± 0.0030962	0.00094150 ± 0.00004117	0.0256
17D18051	16.0 %		0.3374478 ± 0.0029467	0.00105708 ± 0.00004340	0.0249
17D18053	17.6 %		0.3259932 ± 0.0024851	0.00108115 ± 0.00004312	0.0210
17D18054	19.3 %		0.3176234 ± 0.0038542	0.00114223 ± 0.00005542	0.0275
17D18056	21.0 %		0.3077188 ± 0.0057648	0.00115270 ± 0.00007040	0.0325

Results	40(a)/36(a) ± 2σ		40(r)/39(k) ± 2σ		Age ± 2σ (Ma)		MSWD
**Inverse Isochron**	**354.12**	**± 7.97**<br>**± 2.25%**	2.52619	± 0.00990<br>± 0.39%	**6.97**	**± 0.03**<br>**± 0.44%**	0.71<br>70%
					Full External Error	± 0.16	
					Analytical Error	± 0.03	

Statistics				
2σ Confidence Limit	1.94	Convergence	0.0003231990
Error Magnification	1.0000	Number of Iterations	2
Number of Data Points	11	Calculated Line	Weighted York-2
Spreading Factor	17.7%		

Degassing Patterns			36Ar(a) [fA]	%1σ	36Ar(c) [fA]	%1σ	36Ar(ca) [fA]	%1σ	36Ar(cl) [fA]	%1σ	37Ar(ca) [fA]	%1σ	38Ar(a) [fA]	%1σ	38Ar(c) [fA]	%1σ	38Ar(k) [fA]	%1σ	38Ar(ca) [fA]	%1σ	38Ar(cl) [fA]	%1σ	39Ar(k) [fA]	%1σ	39Ar(ca) [fA]	%1σ	40Ar(r) [fA]	%1σ	40Ar(a) [fA]	%1σ	40Ar(c) [fA]	%1σ	40Ar(k) [fA]	%1σ
17D18022	1.8 %	✓	0.1427640	0.71	0.0000000	0.00	0.0197004	0.61	0.0000000	0.00	73.9781	0.59	0.0266826	0.71	0.0000000	0.00	0.5999721	0.19	0.0053116	12.83	0.0000000	0.00	49.86885	0.09	0.0499796	1.45	125.9838	0.57	50.55557	1.33	0.0000000	0.00	0.1906486	2.66
17D18024	1.9 %	✓	0.0859730	0.89	0.0000000	0.00	0.0181239	0.63	0.0000000	0.00	68.0583	0.61	0.0160683	0.89	0.0000000	0.00	0.5255073	0.19	0.0048866	12.83	0.0000000	0.00	43.67944	0.10	0.0459802	1.46	110.0961	0.41	30.44474	1.44	0.0000000	0.00	0.1669865	2.66
17D18025	2.0 %	✓	0.0356875	1.30	0.0000000	0.00	0.0100393	0.93	0.0000000	0.00	37.6991	0.92	0.0066700	1.30	0.0000000	0.00	0.2731883	0.22	0.0027068	12.85	0.0000000	0.00	22.70703	0.15	0.0254695	1.61	57.4452	0.39	12.63766	1.72	0.0000000	0.00	0.0868090	2.66
17D18027	2.2 %	✓	0.0277838	1.60	0.0000000	0.00	0.0093826	1.00	0.0000030	164.46	35.2333	0.99	0.0051928	1.60	0.0000000	0.00	0.2361881	0.24	0.0025298	12.86	0.0146915	164.46	19.63163	0.17	0.0238036	1.65	49.6348	0.40	9.83880	1.95	0.0000000	0.00	0.0750517	2.67
17D18028	2.4 %	✓	0.0140935	2.55	0.0000000	0.00	0.0051083	1.60	0.0000000	0.00	19.1825	1.59	0.0026341	2.55	0.0000000	0.00	0.1264968	0.35	0.0013773	12.92	0.0000000	0.00	10.51424	0.31	0.0129597	2.07	26.4476	0.54	4.99079	2.79	0.0000000	0.00	0.0401959	2.68
17D18030	2.7 %	✓	0.0720646	0.93	0.0000000	0.00	0.0335936	0.51	0.0000000	0.00	126.1494	0.48	0.0134689	0.93	0.0000000	0.00	0.7680396	0.18	0.0090575	12.83	0.0000000	0.00	63.83838	0.09	0.0852265	1.41	161.5297	0.23	25.51950	1.46	0.0000000	0.00	0.2440541	2.66
17D18031	3.0 %	✓	0.0517870	1.16	0.0000000	0.00	0.0317462	0.51	0.0000000	0.00	119.2121	0.49	0.0096790	1.16	0.0000000	0.00	0.6372175	0.18	0.0085594	12.83	0.0000000	0.00	52.96464	0.09	0.0805397	1.41	134.2006	0.23	18.33881	1.61	0.0000000	0.00	0.2024838	2.66
17D18033	3.4 %	✓	0.0435131	1.39	0.0000000	0.00	0.0315036	0.52	0.0000000	0.00	118.3012	0.49	0.0081326	1.39	0.0000000	0.00	0.5683171	0.19	0.0084940	12.83	0.0000000	0.00	47.23773	0.10	0.0799243	1.41	119.2319	0.24	15.40885	1.79	0.0000000	0.00	0.1805898	2.66
17D18034	3.9 %	✓	0.0507689	1.31	0.0000000	0.00	0.0408109	0.48	0.0000000	0.00	153.2515	0.46	0.0094887	1.31	0.0000000	0.00	0.6785067	0.18	0.0110035	12.83	0.0000000	0.00	56.39653	0.09	0.1035367	1.40	142.3629	0.22	17.97830	1.73	0.0000000	0.00	0.2156039	2.66
17D18036	4.5 %	✓	0.0514986	1.36	0.0000000	0.00	0.0456162	0.47	0.0000000	0.00	171.2963	0.45	0.0096251	1.36	0.0000000	0.00	0.6989389	0.18	0.0122991	12.83	0.0000000	0.00	58.09483	0.09	0.1157278	1.39	146.4981	0.22	18.23667	1.77	0.0000000	0.00	0.2220965	2.66
17D18037	5.2 %	✓	0.0356190	1.60	0.0000000	0.00	0.0320413	0.52	0.0000000	0.00	120.3202	0.50	0.0066572	1.60	0.0000000	0.00	0.4666148	0.19	0.0086390	12.83	0.0000000	0.00	38.78437	0.11	0.0812883	1.41	97.8044	0.26	12.61338	1.95	0.0000000	0.00	0.1482726	2.66
17D18039	6.0 %		0.0619009	1.21	0.0000000	0.00	0.0450847	0.48	0.0000010	494.63	169.3005	0.45	0.0115693	1.21	0.0000000	0.00	0.6941800	0.18	0.0121558	12.83	0.0048166	494.63	57.69928	0.09	0.1143794	1.40	144.0481	0.25	21.92035	1.65	0.0000000	0.00	0.2205843	2.66
17D18040	6.9 %		0.0625776	1.04	0.0000000	0.00	0.0378550	0.49	0.0000074	64.91	142.1516	0.47	0.0116958	1.04	0.0000000	0.00	0.6196521	0.18	0.0102065	12.83	0.0363279	64.91	51.50462	0.09	0.0960376	1.40	127.3806	0.27	22.15998	1.53	0.0000000	0.00	0.1969022	2.66
17D18042	7.9 %		0.0884294	0.91	0.0000000	0.00	0.0450329	0.48	0.0000129	37.65	169.1061	0.45	0.0165275	0.91	0.0000000	0.00	0.7479958	0.18	0.0121418	12.83	0.0631686	37.66	62.17237	0.08	0.1142481	1.40	149.7020	0.30	31.31463	1.45	0.0000000	0.00	0.2376850	2.66
17D18043	9.0 %		0.0813406	0.98	0.0000000	0.00	0.0433717	0.48	0.0000130	37.67	162.8677	0.45	0.0152026	0.98	0.0000000	0.00	0.5696947	0.19	0.0116939	12.83	0.0633731	37.68	47.35223	0.10	0.1100334	1.40	109.5806	0.40	28.80435	1.49	0.0000000	0.00	0.1810276	2.66
17D18045	10.3 %		0.0708870	0.99	0.0000000	0.00	0.0443037	0.48	0.0000147	30.28	166.3676	0.45	0.0132488	0.99	0.0000000	0.00	0.4155386	0.20	0.0119452	12.83	0.0715957	30.30	34.53899	0.11	0.1123980	1.40	75.8809	0.50	25.10250	1.50	0.0000000	0.00	0.1320426	2.66
17D18046	11.6 %		0.0745144	1.06	0.0000000	0.00	0.0638631	0.46	0.0000087	55.12	239.8162	0.43	0.0139267	1.06	0.0000000	0.00	0.3677331	0.20	0.0172188	12.83	0.0423116	55.13	30.56546	0.12	0.1620198	1.39	62.6582	0.65	26.38703	1.54	0.0000000	0.00	0.1168518	2.66
17D18047	12.5 %		0.0500852	1.33	0.0000000	0.00	0.0492334	0.47	0.0000097	52.73	184.8795	0.45	0.0093609	1.33	0.0000000	0.00	0.2293619	0.24	0.0132743	12.83	0.0472020	52.73	19.06424	0.17	0.1249046	1.39	37.7672	0.82	17.73617	1.74	0.0000000	0.00	0.0728826	2.67
17D18049	13.4 %		0.0246766	2.07	0.0000000	0.00	0.0276658	0.53	0.0000000	0.00	103.8895	0.51	0.0046121	2.07	0.0000000	0.00	0.1146967	0.37	0.0074593	12.83	0.0000000	0.00	9.53343	0.34	0.0701878	1.41	18.7079	1.11	8.73847	2.35	0.0000000	0.00	0.0364463	2.68
17D18050	14.6 %		0.0203537	2.18	0.0000000	0.00	0.0206431	0.61	0.0000000	0.00	77.5181	0.59	0.0038041	2.18	0.0000000	0.00	0.0903148	0.45	0.0055658	12.83	0.0000000	0.00	7.50684	0.42	0.0523712	1.45	14.4108	1.25	7.20766	2.45	0.0000000	0.00	0.0286987	2.69
17D18051	16.0 %		0.0241211	2.05	0.0000000	0.00	0.0310685	0.52	0.0000116	42.58	116.6672	0.49	0.0045082	2.05	0.0000000	0.00	0.0926395	0.44	0.0083767	12.83	0.0567660	42.59	7.70007	0.41	0.0788204	1.41	14.2768	1.42	8.54177	2.34	0.0000000	0.00	0.0294374	2.69
17D18053	17.6 %		0.0289810	1.99	0.0000000	0.00	0.0424189	0.48	0.0000006	831.32	159.2897	0.46	0.0054165	1.99	0.0000000	0.00	0.1051324	0.39	0.0114370	12.83	0.0028274	831.32	8.73846	0.36	0.1076161	1.40	16.5429	1.43	10.26273	2.29	0.0000000	0.00	0.0334071	2.68
17D18054	19.3 %		0.0196845	2.42	0.0000000	0.00	0.0292656	0.53	0.0000000	0.00	109.8970	0.50	0.0036790	2.42	0.0000000	0.00	0.0658546	0.59	0.0078906	12.83	0.0000000	0.00	5.47375	0.57	0.0742464	1.41	10.2628	1.84	6.97067	2.67	0.0000000	0.00	0.0209261	2.72
17D18056	21.0 %		0.0131107	3.04	0.0000000	0.00	0.0167904	0.66	0.0000000	0.00	63.0505	0.64	0.0024504	3.04	0.0000000	0.00	0.0421081	0.90	0.0045270	12.84	0.0000000	0.00	3.49996	0.89	0.0425969	1.47	6.7311	2.29	4.64276	3.24	0.0000000	0.00	0.0133804	2.80
		Σ	1.2322155	0.25	0.0000000	0.00	0.7742628	0.11	0.0000826	18.59	2907.4832	0.11	0.2303011	0.25	0.0000000	0.00	9.7338897	0.05	0.2087573	2.86	0.4030805	18.59	809.06738	0.03	1.9642956	0.31	1959.1851	0.08	436.35216	0.37	0.0000000	0.00	3.0930646	0.64
		Σ							2.0065609	0.16	2907.4832	0.11									10.576029	0.71			811.03168	0.03							2398.6303	0.10

Additional Parameters			40Ar/39Ar	1σ	37Ar/39Ar	1σ	36Ar/39Ar	1σ	Time (days)	37Ar (decay)	39Ar (decay)	40Ar (moles)
17D18022	1.8 %	✓	3.540348	0.005785	1.481967	0.008860	0.003255	0.000020	126.024	12.081633	1.00089050	8.483E-12
17D18024	1.9 %	✓	3.217987	0.004296	1.556494	0.009661	0.002381	0.000018	126.037	12.084948	1.00089060	6.754E-12
17D18025	2.0 %	✓	3.086754	0.005502	1.658378	0.015396	0.002012	0.000020	126.044	12.086605	1.00089065	3.368E-12
17D18027	2.2 %	✓	3.029630	0.005626	1.792550	0.018055	0.001891	0.000022	126.058	12.089922	1.00089075	2.858E-12
17D18028	2.4 %	✓	2.990218	0.009769	1.822187	0.029482	0.001824	0.000034	126.065	12.091580	1.00089080	1.511E-12
17D18030	2.7 %	✓	2.929954	0.002629	1.973439	0.009712	0.001653	0.000010	126.079	12.094898	1.00089089	8.990E-12
17D18031	3.0 %	✓	2.879468	0.002957	2.247368	0.011199	0.001575	0.000011	126.086	12.096557	1.00089094	7.332E-12
17D18033	3.4 %	✓	2.849283	0.003004	2.500148	0.012572	0.001585	0.000012	126.100	12.099876	1.00089104	6.471E-12
17D18034	3.9 %	✓	2.841710	0.002718	2.712413	0.012709	0.001621	0.000011	126.107	12.101536	1.00089109	7.707E-12
17D18036	4.5 %	✓	2.833797	0.002628	2.942702	0.013417	0.001668	0.000012	126.121	12.104856	1.00089119	7.918E-12
17D18037	5.2 %	✓	2.844826	0.003295	3.095797	0.015686	0.001741	0.000014	126.128	12.106516	1.00089124	5.307E-12
17D18039	6.0 %		2.874563	0.002638	2.928382	0.013458	0.001851	0.000013	126.142	12.109838	1.00089133	7.977E-12
17D18040	6.9 %		2.901852	0.002899	2.754840	0.013240	0.001946	0.000012	126.149	12.111499	1.00089138	7.187E-12
17D18042	7.9 %		2.910005	0.002594	2.714966	0.012497	0.002143	0.000013	126.163	12.114988	1.00089149	8.700E-12
17D18043	9.0 %		2.919498	0.003079	3.431519	0.015935	0.002628	0.000016	126.170	12.116650	1.00089154	6.651E-12
17D18045	10.3 %		2.918076	0.003561	4.801182	0.022502	0.003325	0.000020	126.184	12.119975	1.00089163	4.854E-12
17D18046	11.6 %		2.901705	0.003782	7.804615	0.035158	0.004504	0.000024	126.191	12.121637	1.00089168	4.280E-12
17D18047	12.5 %		2.896235	0.005303	9.634588	0.046145	0.005176	0.000034	126.198	12.123300	1.00089173	2.668E-12
17D18049	13.4 %		2.861716	0.010156	10.817752	0.065501	0.005450	0.000054	126.212	12.126626	1.00089183	1.319E-12
17D18050	14.6 %		2.863677	0.012684	10.254784	0.073801	0.005423	0.000061	126.219	12.128290	1.00089188	1.039E-12
17D18051	16.0 %		2.937178	0.012700	14.997925	0.095945	0.007096	0.000067	126.226	12.129954	1.00089193	1.097E-12
17D18053	17.6 %		3.034007	0.011426	18.006816	0.104444	0.008071	0.000067	126.240	12.133282	1.00089203	1.288E-12
17D18054	19.3 %		3.110021	0.018632	19.808423	0.149930	0.008823	0.000095	126.247	12.134946	1.00089208	8.282E-13
17D18056	21.0 %		3.214421	0.029777	17.798005	0.193054	0.008441	0.000131	126.260	12.138276	1.00089217	5.466E-13

Procedure Blanks		36Ar ± 1σ (SE) [fA]	37Ar ± 1σ (SE) [fA]	38Ar ± 1σ (SE) [fA]	39Ar ± 1σ (SE) [fA]	40Ar ± 1σ (SE) [fA]
17D18022	1.8 %	0.0039396 ± 0.0002054	0.1201230 ± 0.0180800	0.0102243 ± 0.0168176	0.0166887 ± 0.0261427	1.0486496 ± 0.0288350
17D18024	1.9 %	0.0039995 ± 0.0002054	0.1247946 ± 0.0180800	0.0121886 ± 0.0168176	0.0151578 ± 0.0261427	1.0542211 ± 0.0288350
17D18025	2.0 %	0.0040221 ± 0.0002054	0.1243783 ± 0.0180800	0.0110659 ± 0.0168176	0.0166574 ± 0.0261427	1.0539037 ± 0.0288350
17D18027	2.2 %	0.0040502 ± 0.0002054	0.1199130 ± 0.0180800	0.0063315 ± 0.0168176	0.0223231 ± 0.0261427	1.0488312 ± 0.0288350
17D18028	2.4 %	0.0040551 ± 0.0002054	0.1165228 ± 0.0180800	0.0033178 ± 0.0168176	0.0258422 ± 0.0261427	1.0447143 ± 0.0288350
17D18030	2.7 %	0.0040458 ± 0.0002054	0.1088490 ± 0.0180800	0.0027327 ± 0.0168176	0.0328826 ± 0.0261427	1.0347411 ± 0.0288350
17D18031	3.0 %	0.0040317 ± 0.0002054	0.1050441 ± 0.0180800	0.0053518 ± 0.0168176	0.0359541 ± 0.0261427	1.0293844 ± 0.0288350
17D18033	3.4 %	0.0039854 ± 0.0002054	0.0984700 ± 0.0180800	0.0089581 ± 0.0168176	0.0403225 ± 0.0261427	1.0190061 ± 0.0288350
17D18034	3.9 %	0.0039539 ± 0.0002054	0.0959994 ± 0.0180800	0.0097078 ± 0.0168176	0.0413670 ± 0.0261427	1.0143454 ± 0.0288350
17D18036	4.5 %	0.0038771 ± 0.0002054	0.0932129 ± 0.0180800	0.0087319 ± 0.0168176	0.0407922 ± 0.0261427	1.0068098 ± 0.0288350
17D18037	5.2 %	0.0038330 ± 0.0002054	0.0930157 ± 0.0180800	0.0069489 ± 0.0168176	0.0391177 ± 0.0261427	1.0041570 ± 0.0288350
17D18039	6.0 %	0.0037381 ± 0.0002054	0.0950848 ± 0.0180800	0.0008750 ± 0.0168176	0.0331034 ± 0.0261427	1.0014640 ± 0.0288350
17D18040	6.9 %	0.0036892 ± 0.0002054	0.0972898 ± 0.0180800	0.0032930 ± 0.0168176	0.0289057 ± 0.0261427	1.0015072 ± 0.0288350
17D18042	7.9 %	0.0035901 ± 0.0002054	0.1040372 ± 0.0180800	0.0139064 ± 0.0168176	0.0181778 ± 0.0261427	1.0046500 ± 0.0288350
17D18043	9.0 %	0.0035475 ± 0.0002054	0.1079995 ± 0.0180800	0.0195221 ± 0.0168176	0.0125213 ± 0.0261427	1.0075391 ± 0.0288350
17D18045	10.3 %	0.0034790 ± 0.0002054	0.1165177 ± 0.0180800	0.0308261 ± 0.0168176	0.0012773 ± 0.0261427	1.0156865 ± 0.0288350
17D18046	11.6 %	0.0034566 ± 0.0002054	0.1206462 ± 0.0180800	0.0360249 ± 0.0168176	0.0037670 ± 0.0261427	1.0207465 ± 0.0288350
17D18047	12.5 %	0.0034444 ± 0.0002054	0.1243528 ± 0.0180800	0.0405453 ± 0.0168176	0.0080093 ± 0.0261427	1.0262932 ± 0.0288350
17D18049	13.4 %	0.0034592 ± 0.0002054	0.1293151 ± 0.0180800	0.0462411 ± 0.0168176	0.0126397 ± 0.0261427	1.0381960 ± 0.0288350
17D18050	14.6 %	0.0034903 ± 0.0002054	0.1299034 ± 0.0180800	0.0466868 ± 0.0168176	0.0122217 ± 0.0261427	1.0441689 ± 0.0288350
17D18051	16.0 %	0.0035404 ± 0.0002054	0.1287350 ± 0.0180800	0.0449944 ± 0.0168176	0.0093892 ± 0.0261427	1.0498621 ± 0.0288350
17D18053	17.6 %	0.0037072 ± 0.0002054	0.1194629 ± 0.0180800	0.0334050 ± 0.0168176	0.0054931 ± 0.0261427	1.0593889 ± 0.0288350
17D18054	19.3 %	0.0038291 ± 0.0002054	0.1104518 ± 0.0180800	0.0225380 ± 0.0168176	0.0186122 ± 0.0261427	1.0626544 ± 0.0288350
17D18056	21.0 %	0.0041637 ± 0.0002054	0.0811859 ± 0.0180800	0.0119915 ± 0.0168176	0.0591255 ± 0.0261427	1.0645962 ± 0.0288350

**Intercept Values**		**36Ar ± 1σ (SE) [fA]**	**r2**	**Regression (type,n)**	**37Ar ± 1σ (SE) [fA]**	**r2**	**Regression (type,n)**	**38Ar ± 1σ (SE) [fA]**	**r2**	**Regression (type,n)**	**39Ar ± 1σ (SE) [fA]**	**r2**	**Regression (type,n)**	**40Ar ± 1σ (SE) [fA]**	**r2**	**Regression (type,n)**
17D18022	1.8 %	0.1603039 ± 0.0008390	0.6068	EXP 150 of 150	5.8935492 ± 0.0179455	0.7700	EXP 150 of 150	0.5907441 ± 0.0166030	0.0128	EXP 149 of 150	49.5939138 ± 0.0187224	0.9965	EXP 150 of 150	177.778684 ± 0.234788	0.7714	EXP 150 of 150
17D18024	1.9 %	0.1041879 ± 0.0006456	0.5543	EXP 150 of 150	5.4061468 ± 0.0174189	0.7403	EXP 149 of 150	0.5206100 ± 0.0149453	0.0507	EXP 150 of 150	43.4413511 ± 0.0182624	0.9957	EXP 150 of 150	141.762071 ± 0.121332	0.7931	EXP 150 of 150
17D18025	2.0 %	0.0480320 ± 0.0003669	0.8356	EXP 149 of 150	2.9389168 ± 0.0173361	0.4836	EXP 150 of 150	0.2604472 ± 0.0172416	0.0016	EXP 150 of 150	22.5935923 ± 0.0174482	0.9849	EXP 150 of 150	71.223549 ± 0.054926	0.9802	EXP 150 of 150
17D18027	2.2 %	0.0398240 ± 0.0003495	0.7770	EXP 150 of 150	2.7422390 ± 0.0184727	0.4628	EXP 150 of 150	0.2491833 ± 0.0169264	0.0103	EXP 150 of 150	19.5432421 ± 0.0164581	0.9822	EXP 150 of 150	60.597519 ± 0.029896	0.9903	EXP 150 of 150
17D18028	2.4 %	0.0225359 ± 0.0002626	0.8417	EXP 150 of 150	1.4415406 ± 0.0156441	0.1641	EXP 150 of 150	0.1181916 ± 0.0158674	0.0003	EXP 150 of 150	10.4809981 ± 0.0170609	0.9267	EXP 150 of 150	32.523329 ± 0.021388	0.9966	EXP 150 of 150
17D18030	2.7 %	0.1057368 ± 0.0005137	0.3599	EXP 150 of 150	10.1345796 ± 0.0190277	0.9046	EXP 150 of 150	0.7696216 ± 0.0167773	0.0835	EXP 150 of 150	63.5190224 ± 0.0195039	0.9978	EXP 150 of 150	188.327950 ± 0.045724	0.9462	EXP 150 of 150
17D18031	3.0 %	0.0844284 ± 0.0004644	0.5218	EXP 150 of 150	9.5737413 ± 0.0182951	0.8983	EXP 150 of 150	0.6299730 ± 0.0149146	0.0227	EXP 150 of 150	52.7181083 ± 0.0190613	0.9969	EXP 150 of 150	153.771272 ± 0.065493	0.2721	EXP 150 of 150
17D18033	3.4 %	0.0761854 ± 0.0004822	0.3389	EXP 150 of 150	9.5037262 ± 0.0189162	0.8953	EXP 150 of 150	0.5426398 ± 0.0156667	0.0005	EXP 150 of 150	47.0341489 ± 0.0186456	0.9962	EXP 150 of 150	135.840387 ± 0.050639	0.2024	EXP 150 of 150
17D18034	3.9 %	0.0920952 ± 0.0005245	0.3570	EXP 150 of 150	12.3413194 ± 0.0179941	0.9383	EXP 150 of 150	0.6841688 ± 0.0164208	0.0341	EXP 150 of 150	56.1547628 ± 0.0207548	0.9967	EXP 150 of 150	161.571183 ± 0.042832	0.8640	EXP 149 of 150
17D18036	4.5 %	0.0973455 ± 0.0005522	0.3013	EXP 150 of 150	13.8047424 ± 0.0163669	0.9605	EXP 150 of 150	0.7013704 ± 0.0184399	0.0087	EXP 150 of 150	57.8529633 ± 0.0190242	0.9975	EXP 150 of 150	165.963720 ± 0.040055	0.9332	EXP 150 of 150
17D18037	5.2 %	0.0689529 ± 0.0004473	0.4457	EXP 150 of 150	9.6677031 ± 0.0199693	0.8956	EXP 150 of 150	0.4706640 ± 0.0156236	0.0174	EXP 150 of 150	38.6387826 ± 0.0171950	0.9953	EXP 150 of 150	111.570177 ± 0.043986	0.6742	EXP 150 of 150
17D18039	6.0 %	0.1067077 ± 0.0005966	0.1605	EXP 150 of 150	13.6352876 ± 0.0178348	0.9511	EXP 150 of 150	0.7149684 ± 0.0163203	0.0777	EXP 149 of 150	57.4510823 ± 0.0171595	0.9979	EXP 150 of 150	167.190484 ± 0.039955	0.9413	EXP 150 of 150
17D18040	6.9 %	0.1003580 ± 0.0004942	0.2204	EXP 150 of 150	11.4297090 ± 0.0194418	0.9232	EXP 150 of 150	0.6664963 ± 0.0159994	0.0587	EXP 150 of 150	51.2764067 ± 0.0184131	0.9969	EXP 150 of 150	150.738973 ± 0.050561	0.6211	EXP 150 of 150
17D18042	7.9 %	0.1320538 ± 0.0006202	0.0757	EXP 150 of 150	13.6047386 ± 0.0182955	0.9482	EXP 150 of 150	0.8159009 ± 0.0162366	0.1353	EXP 150 of 150	61.8784974 ± 0.0176051	0.9981	EXP 150 of 150	182.259005 ± 0.041377	0.9560	EXP 150 of 150
17D18043	9.0 %	0.1235897 ± 0.0006275	0.0276	EXP 149 of 150	13.0932409 ± 0.0178182	0.9512	EXP 150 of 150	0.6325632 ± 0.0164035	0.0545	EXP 149 of 150	47.1499450 ± 0.0183350	0.9964	EXP 150 of 150	139.573507 ± 0.054117	0.0165	EXP 150 of 150
17D18045	10.3 %	0.1143588 ± 0.0005239	0.0166	EXP 150 of 150	13.3647136 ± 0.0186727	0.9453	EXP 150 of 150	0.4753858 ± 0.0131220	0.0506	EXP 150 of 150	34.4155001 ± 0.0175948	0.9937	EXP 150 of 150	102.131086 ± 0.033759	0.8676	EXP 150 of 150
17D18046	11.6 %	0.1366467 ± 0.0005645	0.0246	EXP 150 of 150	19.3096627 ± 0.0193546	0.9701	EXP 150 of 150	0.3998982 ± 0.0155656	0.0024	EXP 149 of 150	30.5134074 ± 0.0164019	0.9928	EXP 150 of 150	90.182841 ± 0.030055	0.9522	EXP 150 of 150
17D18047	12.5 %	0.0990433 ± 0.0005003	0.0046	EXP 149 of 150	14.8528387 ± 0.0187864	0.9526	EXP 150 of 150	0.2550819 ± 0.0178423	0.0018	EXP 150 of 150	19.0497967 ± 0.0149284	0.9837	EXP 150 of 150	56.602562 ± 0.020017	0.9939	EXP 150 of 150
17D18049	13.4 %	0.0538362 ± 0.0003997	0.3632	EXP 150 of 150	8.2845251 ± 0.0168606	0.8850	EXP 150 of 150	0.0620136 ± 0.0145679	0.0257	EXP 150 of 150	9.5252436 ± 0.0167935	0.9136	EXP 150 of 150	28.521020 ± 0.017864	0.9969	EXP 150 of 150
17D18050	14.6 %	0.0429478 ± 0.0003375	0.5200	EXP 150 of 150	6.1472993 ± 0.0192518	0.7926	EXP 150 of 150	0.0421097 ± 0.0160261	0.0071	EXP 149 of 150	7.4952546 ± 0.0160095	0.8692	EXP 150 of 150	22.691324 ± 0.018282	0.9971	EXP 150 of 150
17D18051	16.0 %	0.0566690 ± 0.0003720	0.3720	EXP 150 of 150	9.3173574 ± 0.0184894	0.8981	EXP 150 of 150	0.1153587 ± 0.0169114	0.0342	EXP 150 of 150	7.7162581 ± 0.0164971	0.8689	EXP 150 of 150	23.897852 ± 0.018212	0.9970	EXP 150 of 150
17D18053	17.6 %	0.0724267 ± 0.0004372	0.0861	EXP 150 of 150	12.7740701 ± 0.0187897	0.9387	EXP 149 of 150	0.0899182 ± 0.0159427	0.0030	EXP 150 of 150	8.7910234 ± 0.0159034	0.9087	EXP 150 of 150	27.898454 ± 0.017777	0.9967	EXP 150 of 150
17D18054	19.3 %	0.0509412 ± 0.0003589	0.4220	EXP 150 of 150	8.7838209 ± 0.0185132	0.8793	EXP 150 of 150	0.0468072 ± 0.0158456	0.0000	EXP 150 of 150	5.5286307 ± 0.0164301	0.7726	EXP 150 of 150	18.317032 ± 0.019213	0.9971	EXP 150 of 150
17D18056	21.0 %	0.0329421 ± 0.0002952	0.6461	EXP 150 of 150	5.0202702 ± 0.0173735	0.7496	EXP 149 of 150	0.0244872 ± 0.0178423	0.0011	EXP 150 of 150	3.5774382 ± 0.0160690	0.5421	EXP 150 of 150	12.451879 ± 0.019295	0.9973	EXP 150 of 150

Project Info		Analyst	Irradiation	X-pos	Y-pos	Z/H-pos	Project	Experiment	Nmb
17D18022	1.8 %	Dan Miggins	17-OSU-01	0.00	0.00	59.89	Mozambique Ridge\O-Connor (16-23)	17D18018	01
17D18024	1.9 %	Dan Miggins	17-OSU-01	0.00	0.00	59.89	Mozambique Ridge\O-Connor (16-23)	17D18018	01
17D18025	2.0 %	Dan Miggins	17-OSU-01	0.00	0.00	59.89	Mozambique Ridge\O-Connor (16-23)	17D18018	01
17D18027	2.2 %	Dan Miggins	17-OSU-01	0.00	0.00	59.89	Mozambique Ridge\O-Connor (16-23)	17D18018	01
17D18028	2.4 %	Dan Miggins	17-OSU-01	0.00	0.00	59.89	Mozambique Ridge\O-Connor (16-23)	17D18018	01
17D18030	2.7 %	Dan Miggins	17-OSU-01	0.00	0.00	59.89	Mozambique Ridge\O-Connor (16-23)	17D18018	01
17D18031	3.0 %	Dan Miggins	17-OSU-01	0.00	0.00	59.89	Mozambique Ridge\O-Connor (16-23)	17D18018	01
17D18033	3.4 %	Dan Miggins	17-OSU-01	0.00	0.00	59.89	Mozambique Ridge\O-Connor (16-23)	17D18018	01
17D18034	3.9 %	Dan Miggins	17-OSU-01	0.00	0.00	59.89	Mozambique Ridge\O-Connor (16-23)	17D18018	01
17D18036	4.5 %	Dan Miggins	17-OSU-01	0.00	0.00	59.89	Mozambique Ridge\O-Connor (16-23)	17D18018	01
17D18037	5.2 %	Dan Miggins	17-OSU-01	0.00	0.00	59.89	Mozambique Ridge\O-Connor (16-23)	17D18018	01
17D18039	6.0 %	Dan Miggins	17-OSU-01	0.00	0.00	59.89	Mozambique Ridge\O-Connor (16-23)	17D18018	01
17D18040	6.9 %	Dan Miggins	17-OSU-01	0.00	0.00	59.89	Mozambique Ridge\O-Connor (16-23)	17D18018	01
17D18042	7.9 %	Dan Miggins	17-OSU-01	0.00	0.00	59.89	Mozambique Ridge\O-Connor (16-23)	17D18018	01
17D18043	9.0 %	Dan Miggins	17-OSU-01	0.00	0.00	59.89	Mozambique Ridge\O-Connor (16-23)	17D18018	01
17D18045	10.3 %	Dan Miggins	17-OSU-01	0.00	0.00	59.89	Mozambique Ridge\O-Connor (16-23)	17D18018	01
17D18046	11.6 %	Dan Miggins	17-OSU-01	0.00	0.00	59.89	Mozambique Ridge\O-Connor (16-23)	17D18018	01
17D18047	12.5 %	Dan Miggins	17-OSU-01	0.00	0.00	59.89	Mozambique Ridge\O-Connor (16-23)	17D18018	01
17D18049	13.4 %	Dan Miggins	17-OSU-01	0.00	0.00	59.89	Mozambique Ridge\O-Connor (16-23)	17D18018	01
17D18050	14.6 %	Dan Miggins	17-OSU-01	0.00	0.00	59.89	Mozambique Ridge\O-Connor (16-23)	17D18018	01
17D18051	16.0 %	Dan Miggins	17-OSU-01	0.00	0.00	59.89	Mozambique Ridge\O-Connor (16-23)	17D18018	01
17D18053	17.6 %	Dan Miggins	17-OSU-01	0.00	0.00	59.89	Mozambique Ridge\O-Connor (16-23)	17D18018	01
17D18054	19.3 %	Dan Miggins	17-OSU-01	0.00	0.00	59.89	Mozambique Ridge\O-Connor (16-23)	17D18018	01
17D18056	21.0 %	Dan Miggins	17-OSU-01	0.00	0.00	59.89	Mozambique Ridge\O-Connor (16-23)	17D18018	01

Sample Parameters		Sample	Material	Location	Standard Name	Standard (in Ma)	%1σ	Standard Reference	Standard 40Ar/39Ar	%1σ	J	%1σ	Air 40Ar/36Ar	%1σ	MDF (lin)	%1σ	Volume Ratio	Sensitivity (mol/volt)	Day	Month	Year	Hour	Min	Resist
17D18022	1.8 %	MW14-DL2-2	Groundmass	Mozambique Ridge	FCT-NM (1B38-17)	28.201	0.082	Kuiper et al (2008)	10.28448	0.099	0.00152826	0.099	302.72	0.141	0.9940297	0.068	1	4.8E-14	25	MAY	2017	16	13	1
17D18024	1.9 %	MW14-DL2-2	Groundmass	Mozambique Ridge	FCT-NM (1B38-17)	28.201	0.082	Kuiper et al (2008)	10.28448	0.099	0.00152826	0.099	302.72	0.141	0.9940297	0.068	1	4.8E-14	25	MAY	2017	16	33	1
17D18025	2.0 %	MW14-DL2-2	Groundmass	Mozambique Ridge	FCT-NM (1B38-17)	28.201	0.082	Kuiper et al (2008)	10.28448	0.099	0.00152826	0.099	302.72	0.141	0.9940297	0.068	1	4.8E-14	25	MAY	2017	16	43	1
17D18027	2.2 %	MW14-DL2-2	Groundmass	Mozambique Ridge	FCT-NM (1B38-17)	28.201	0.082	Kuiper et al (2008)	10.28448	0.099	0.00152826	0.099	302.72	0.141	0.9940297	0.068	1	4.8E-14	25	MAY	2017	17	3	1
17D18028	2.4 %	MW14-DL2-2	Groundmass	Mozambique Ridge	FCT-NM (1B38-17)	28.201	0.082	Kuiper et al (2008)	10.28448	0.099	0.00152826	0.099	302.72	0.141	0.9940297	0.068	1	4.8E-14	25	MAY	2017	17	13	1
17D18030	2.7 %	MW14-DL2-2	Groundmass	Mozambique Ridge	FCT-NM (1B38-17)	28.201	0.082	Kuiper et al (2008)	10.28448	0.099	0.00152826	0.099	302.72	0.141	0.9940297	0.068	1	4.8E-14	25	MAY	2017	17	33	1
17D18031	3.0 %	MW14-DL2-2	Groundmass	Mozambique Ridge	FCT-NM (1B38-17)	28.201	0.082	Kuiper et al (2008)	10.28448	0.099	0.00152826	0.099	302.72	0.141	0.9940297	0.068	1	4.8E-14	25	MAY	2017	17	43	1
17D18033	3.4 %	MW14-DL2-2	Groundmass	Mozambique Ridge	FCT-NM (1B38-17)	28.201	0.082	Kuiper et al (2008)	10.28448	0.099	0.00152826	0.099	302.72	0.141	0.9940297	0.068	1	4.8E-14	25	MAY	2017	18	3	1
17D18034	3.9 %	MW14-DL2-2	Groundmass	Mozambique Ridge	FCT-NM (1B38-17)	28.201	0.082	Kuiper et al (2008)	10.28448	0.099	0.00152826	0.099	302.72	0.141	0.9940297	0.068	1	4.8E-14	25	MAY	2017	18	13	1
17D18036	4.5 %	MW14-DL2-2	Groundmass	Mozambique Ridge	FCT-NM (1B38-17)	28.201	0.082	Kuiper et al (2008)	10.28448	0.099	0.00152826	0.099	302.72	0.141	0.9940297	0.068	1	4.8E-14	25	MAY	2017	18	33	1
17D18037	5.2 %	MW14-DL2-2	Groundmass	Mozambique Ridge	FCT-NM (1B38-17)	28.201	0.082	Kuiper et al (2008)	10.28448	0.099	0.00152826	0.099	302.72	0.141	0.9940297	0.068	1	4.8E-14	25	MAY	2017	18	43	1
17D18039	6.0 %	MW14-DL2-2	Groundmass	Mozambique Ridge	FCT-NM (1B38-17)	28.201	0.082	Kuiper et al (2008)	10.28448	0.099	0.00152826	0.099	302.72	0.141	0.9940297	0.068	1	4.8E-14	25	MAY	2017	19	3	1
17D18040	6.9 %	MW14-DL2-2	Groundmass	Mozambique Ridge	FCT-NM (1B38-17)	28.201	0.082	Kuiper et al (2008)	10.28448	0.099	0.00152826	0.099	302.72	0.141	0.9940297	0.068	1	4.8E-14	25	MAY	2017	19	13	1
17D18042	7.9 %	MW14-DL2-2	Groundmass	Mozambique Ridge	FCT-NM (1B38-17)	28.201	0.082	Kuiper et al (2008)	10.28448	0.099	0.00152826	0.099	302.72	0.141	0.9940297	0.068	1	4.8E-14	25	MAY	2017	19	34	1
17D18043	9.0 %	MW14-DL2-2	Groundmass	Mozambique Ridge	FCT-NM (1B38-17)	28.201	0.082	Kuiper et al (2008)	10.28448	0.099	0.00152826	0.099	302.72	0.141	0.9940297	0.068	1	4.8E-14	25	MAY	2017	19	44	1
17D18045	10.3 %	MW14-DL2-2	Groundmass	Mozambique Ridge	FCT-NM (1B38-17)	28.201	0.082	Kuiper et al (2008)	10.28448	0.099	0.00152826	0.099	302.72	0.141	0.9940297	0.068	1	4.8E-14	25	MAY	2017	20	4	1
17D18046	11.6 %	MW14-DL2-2	Groundmass	Mozambique Ridge	FCT-NM (1B38-17)	28.201	0.082	Kuiper et al (2008)	10.28448	0.099	0.00152826	0.099	302.72	0.141	0.9940297	0.068	1	4.8E-14	25	MAY	2017	20	14	1
17D18047	12.5 %	MW14-DL2-2	Groundmass	Mozambique Ridge	FCT-NM (1B38-17)	28.201	0.082	Kuiper et al (2008)	10.28448	0.099	0.00152826	0.099	302.72	0.141	0.9940297	0.068	1	4.8E-14	25	MAY	2017	20	24	1
17D18049	13.4 %	MW14-DL2-2	Groundmass	Mozambique Ridge	FCT-NM (1B38-17)	28.201	0.082	Kuiper et al (2008)	10.28448	0.099	0.00152826	0.099	302.72	0.141	0.9940297	0.068	1	4.8E-14	25	MAY	2017	20	44	1
17D18050	14.6 %	MW14-DL2-2	Groundmass	Mozambique Ridge	FCT-NM (1B38-17)	28.201	0.082	Kuiper et al (2008)	10.28448	0.099	0.00152826	0.099	302.72	0.141	0.9940297	0.068	1	4.8E-14	25	MAY	2017	20	54	1
17D18051	16.0 %	MW14-DL2-2	Groundmass	Mozambique Ridge	FCT-NM (1B38-17)	28.201	0.082	Kuiper et al (2008)	10.28448	0.099	0.00152826	0.099	302.72	0.141	0.9940297	0.068	1	4.8E-14	25	MAY	2017	21	4	1
17D18053	17.6 %	MW14-DL2-2	Groundmass	Mozambique Ridge	FCT-NM (1B38-17)	28.201	0.082	Kuiper et al (2008)	10.28448	0.099	0.00152826	0.099	302.72	0.141	0.9940297	0.068	1	4.8E-14	25	MAY	2017	21	24	1
17D18054	19.3 %	MW14-DL2-2	Groundmass	Mozambique Ridge	FCT-NM (1B38-17)	28.201	0.082	Kuiper et al (2008)	10.28448	0.099	0.00152826	0.099	302.72	0.141	0.9940297	0.068	1	4.8E-14	25	MAY	2017	21	34	1
17D18056	21.0 %	MW14-DL2-2	Groundmass	Mozambique Ridge	FCT-NM (1B38-17)	28.201	0.082	Kuiper et al (2008)	10.28448	0.099	0.00152826	0.099	302.72	0.141	0.9940297	0.068	1	4.8E-14	25	MAY	2017	21	54	1

**Irradiation Constants**		40/36(a)	%1σ	40/36(c)	%1σ	38/36(a)	%1σ	38/36(c)	%1σ	39/37(ca)	%1σ	38/37(ca)	%1σ	36/37(ca)	%1σ	40/39(k)	%1σ	38/39(k)	%1σ	36/38(cl)	%1σ	K/Ca	%1σ	K/Cl	%1σ	Ca/Cl	%1σ
17D18022	1.8 %	354.12	1.126	0.018	35	0.1869	0	1.493	3	0.000676	1.32	7.18E-05	12.82	0.000266	0.15	0.003823	2.66	0.012031	0.16	0	0	0.43	0	0	0	0	0
17D18024	1.9 %	354.12	1.126	0.018	35	0.1869	0	1.493	3	0.000676	1.32	7.18E-05	12.82	0.000266	0.15	0.003823	2.66	0.012031	0.16	0	0	0.43	0	0	0	0	0
17D18025	2.0 %	354.12	1.126	0.018	35	0.1869	0	1.493	3	0.000676	1.32	7.18E-05	12.82	0.000266	0.15	0.003823	2.66	0.012031	0.16	0	0	0.43	0	0	0	0	0
17D18027	2.2 %	354.12	1.126	0.018	35	0.1869	0	1.493	3	0.000676	1.32	7.18E-05	12.82	0.000266	0.15	0.003823	2.66	0.012031	0.16	0	0	0.43	0	0	0	0	0
17D18028	2.4 %	354.12	1.126	0.018	35	0.1869	0	1.493	3	0.000676	1.32	7.18E-05	12.82	0.000266	0.15	0.003823	2.66	0.012031	0.16	0	0	0.43	0	0	0	0	0
17D18030	2.7 %	354.12	1.126	0.018	35	0.1869	0	1.493	3	0.000676	1.32	7.18E-05	12.82	0.000266	0.15	0.003823	2.66	0.012031	0.16	0	0	0.43	0	0	0	0	0
17D18031	3.0 %	354.12	1.126	0.018	35	0.1869	0	1.493	3	0.000676	1.32	7.18E-05	12.82	0.000266	0.15	0.003823	2.66	0.012031	0.16	0	0	0.43	0	0	0	0	0
17D18033	3.4 %	354.12	1.126	0.018	35	0.1869	0	1.493	3	0.000676	1.32	7.18E-05	12.82	0.000266	0.15	0.003823	2.66	0.012031	0.16	0	0	0.43	0	0	0	0	0
17D18034	3.9 %	354.12	1.126	0.018	35	0.1869	0	1.493	3	0.000676	1.32	7.18E-05	12.82	0.000266	0.15	0.003823	2.66	0.012031	0.16	0	0	0.43	0	0	0	0	0
17D18036	4.5 %	354.12	1.126	0.018	35	0.1869	0	1.493	3	0.000676	1.32	7.18E-05	12.82	0.000266	0.15	0.003823	2.66	0.012031	0.16	0	0	0.43	0	0	0	0	0
17D18037	5.2 %	354.12	1.126	0.018	35	0.1869	0	1.493	3	0.000676	1.32	7.18E-05	12.82	0.000266	0.15	0.003823	2.66	0.012031	0.16	0	0	0.43	0	0	0	0	0
17D18039	6.0 %	354.12	1.126	0.018	35	0.1869	0	1.493	3	0.000676	1.32	7.18E-05	12.82	0.000266	0.15	0.003823	2.66	0.012031	0.16	0	0	0.43	0	0	0	0	0
17D18040	6.9 %	354.12	1.126	0.018	35	0.1869	0	1.493	3	0.000676	1.32	7.18E-05	12.82	0.000266	0.15	0.003823	2.66	0.012031	0.16	0	0	0.43	0	0	0	0	0
17D18042	7.9 %	354.12	1.126	0.018	35	0.1869	0	1.493	3	0.000676	1.32	7.18E-05	12.82	0.000266	0.15	0.003823	2.66	0.012031	0.16	0	0	0.43	0	0	0	0	0
17D18043	9.0 %	354.12	1.126	0.018	35	0.1869	0	1.493	3	0.000676	1.32	7.18E-05	12.82	0.000266	0.15	0.003823	2.66	0.012031	0.16	0	0	0.43	0	0	0	0	0
17D18045	10.3 %	354.12	1.126	0.018	35	0.1869	0	1.493	3	0.000676	1.32	7.18E-05	12.82	0.000266	0.15	0.003823	2.66	0.012031	0.16	0	0	0.43	0	0	0	0	0
17D18046	11.6 %	354.12	1.126	0.018	35	0.1869	0	1.493	3	0.000676	1.32	7.18E-05	12.82	0.000266	0.15	0.003823	2.66	0.012031	0.16	0	0	0.43	0	0	0	0	0
17D18047	12.5 %	354.12	1.126	0.018	35	0.1869	0	1.493	3	0.000676	1.32	7.18E-05	12.82	0.000266	0.15	0.003823	2.66	0.012031	0.16	0	0	0.43	0	0	0	0	0
17D18049	13.4 %	354.12	1.126	0.018	35	0.1869	0	1.493	3	0.000676	1.32	7.18E-05	12.82	0.000266	0.15	0.003823	2.66	0.012031	0.16	0	0	0.43	0	0	0	0	0
17D18050	14.6 %	354.12	1.126	0.018	35	0.1869	0	1.493	3	0.000676	1.32	7.18E-05	12.82	0.000266	0.15	0.003823	2.66	0.012031	0.16	0	0	0.43	0	0	0	0	0
17D18051	16.0 %	354.12	1.126	0.018	35	0.1869	0	1.493	3	0.000676	1.32	7.18E-05	12.82	0.000266	0.15	0.003823	2.66	0.012031	0.16	0	0	0.43	0	0	0	0	0
17D18053	17.6 %	354.12	1.126	0.018	35	0.1869	0	1.493	3	0.000676	1.32	7.18E-05	12.82	0.000266	0.15	0.003823	2.66	0.012031	0.16	0	0	0.43	0	0	0	0	0
17D18054	19.3 %	354.12	1.126	0.018	35	0.1869	0	1.493	3	0.000676	1.32	7.18E-05	12.82	0.000266	0.15	0.003823	2.66	0.012031	0.16	0	0	0.43	0	0	0	0	0
17D18056	21.0 %	354.12	1.126	0.018	35	0.1869	0	1.493	3	0.000676	1.32	7.18E-05	12.82	0.000266	0.15	0.003823	2.66	0.012031	0.16	0	0	0.43	0	0	0	0	0

17D18018.AGE >>> MW14-DL2-2 >>> MOZAMBIQUE RIDGE | O-CONNOR (16-23) PROJECT

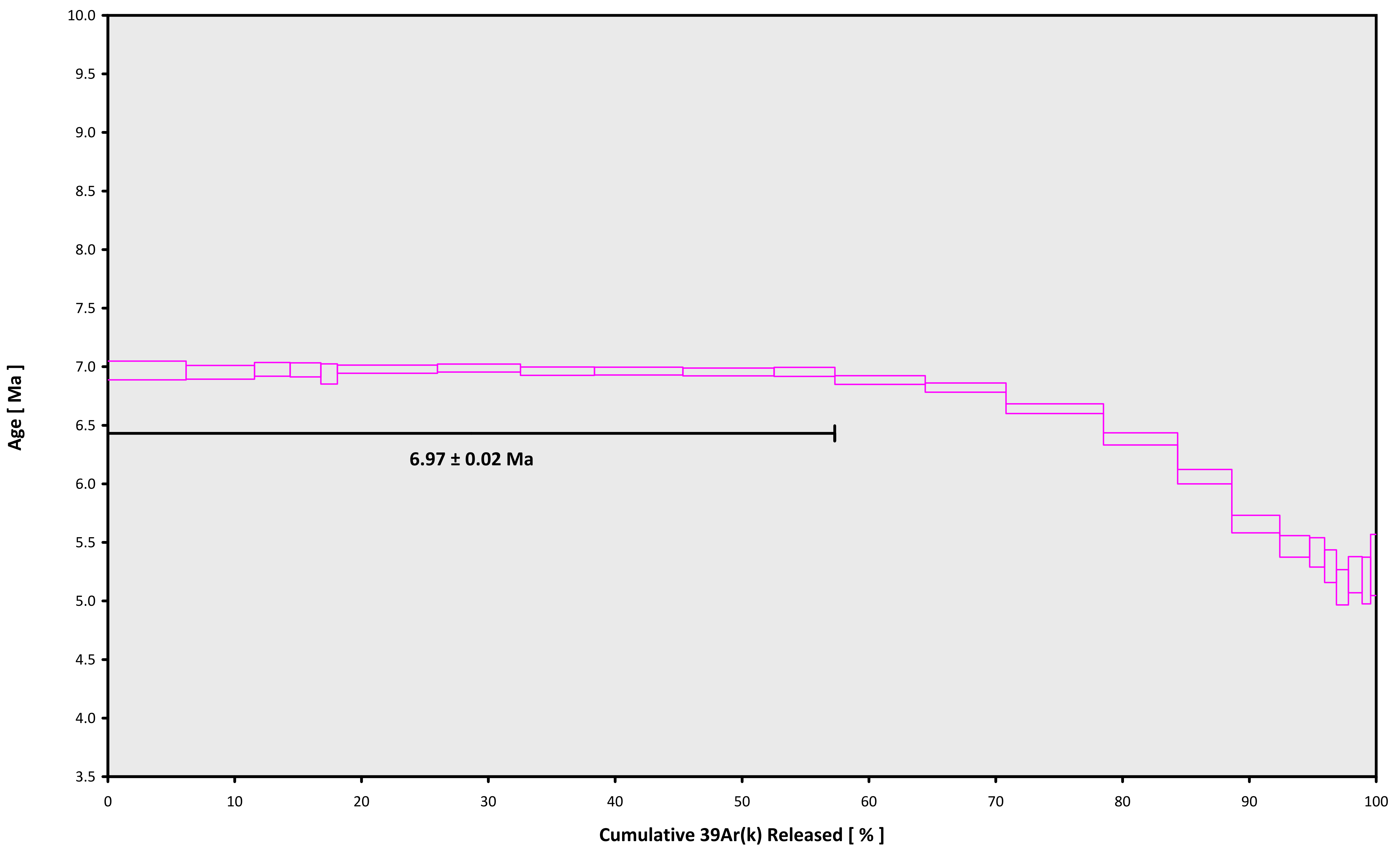

Ar-Ages in Ma

WEIGHTED PLATEAU
6.97 ± 0.02
TOTAL FUSION
6.68 ± 0.02
NORMAL ISOCHRON
6.97 ± 0.03
INVERSE ISOCHRON
6.97 ± 0.03

MSWD (PROBABILITY)
0.40 (95%)

Sample Info

Groundmass
Mozambique Ridge
Dan Miggins

IRR = 17-OSU-01 (1B38-17)
J = 0.00152826 ± 0.00000151

17D18018.AGE >>> MW14-DL2-2 >>> MOZAMBIQUE RIDGE | O-CONNOR (16-23) PROJECT

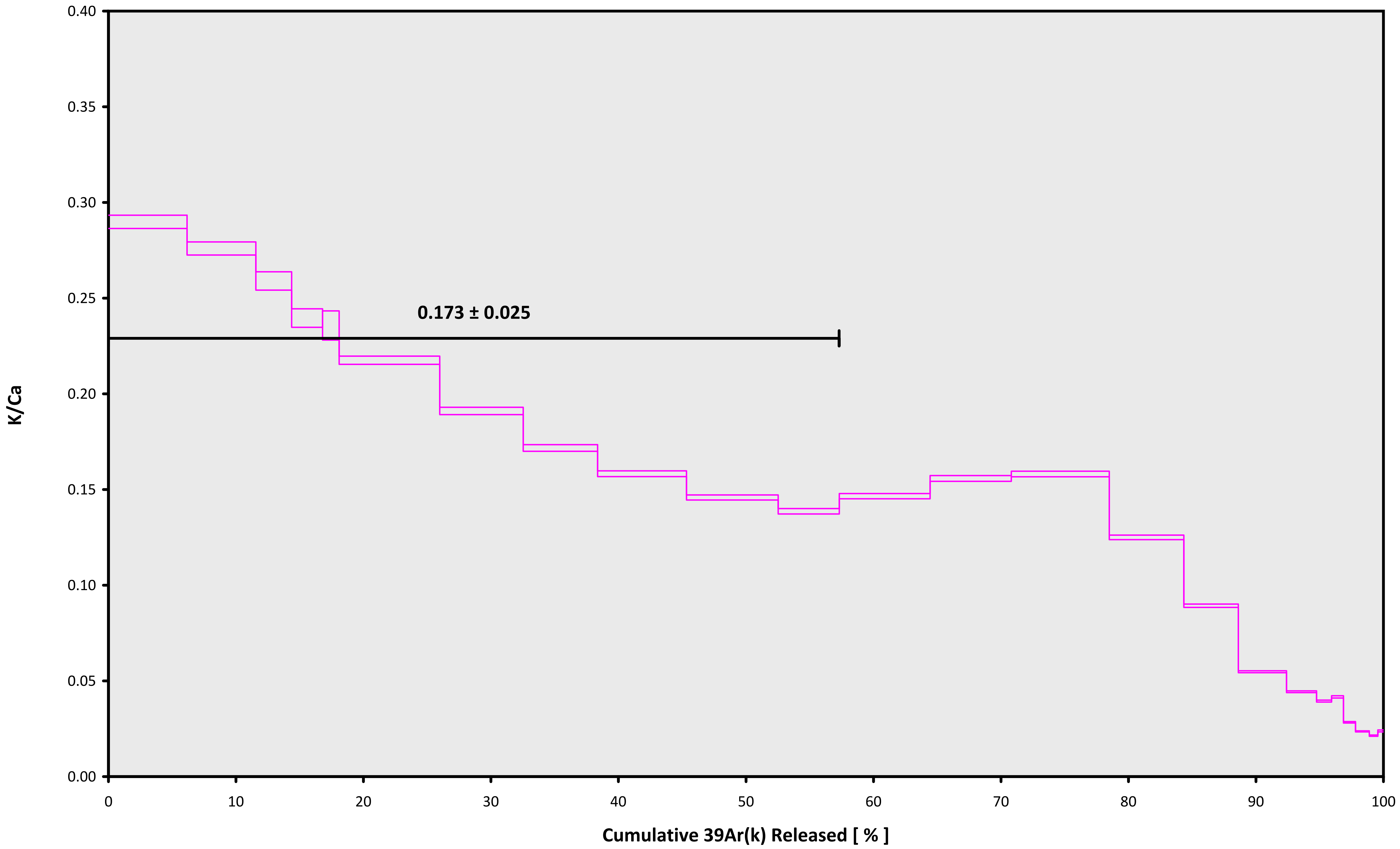

Ar-Ages in Ma

WEIGHTED PLATEAU
6.97 ± 0.02
TOTAL FUSION
6.68 ± 0.02
NORMAL ISOCHRON
6.97 ± 0.03
INVERSE ISOCHRON
6.97 ± 0.03

Sample Info

Groundmass
Mozambique Ridge
Dan Miggins

IRR = 17-OSU-01 (1B38-17)
J = 0.00152826 ± 0.00000151

17D18018.AGE >>> MW14-DL2-2 >>> MOZAMBIQUE RIDGE | O-CONNOR (16-23) PROJECT

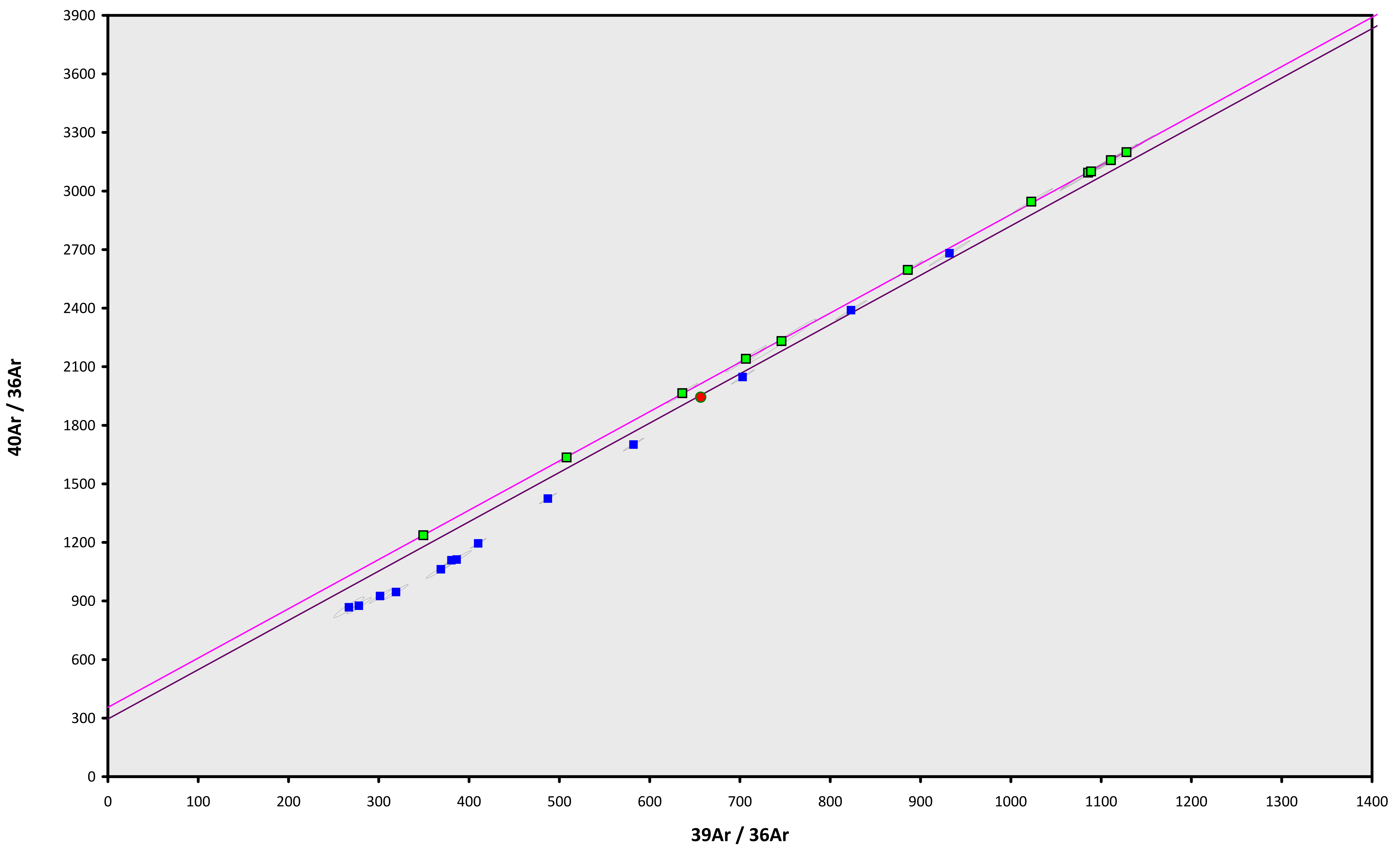

Ar-Ages in Ma

WEIGHTED PLATEAU
6.97 ± 0.02
TOTAL FUSION
6.68 ± 0.02
NORMAL ISOCHRON
6.97 ± 0.03
INVERSE ISOCHRON
6.97 ± 0.03

MSWD (PROBABILITY)
0.71 (70%)

40AR/36AR INTERCEPT
354.7 ± 8.0

Sample Info

Groundmass
Mozambique Ridge
Dan Miggins

IRR = 17-OSU-01 (1B38-17)
J = 0.00152826 ± 0.00000151

17D18018.AGE >>> MW14-DL2-2 >>> MOZAMBIQUE RIDGE | O-CONNOR (16-23) PROJECT

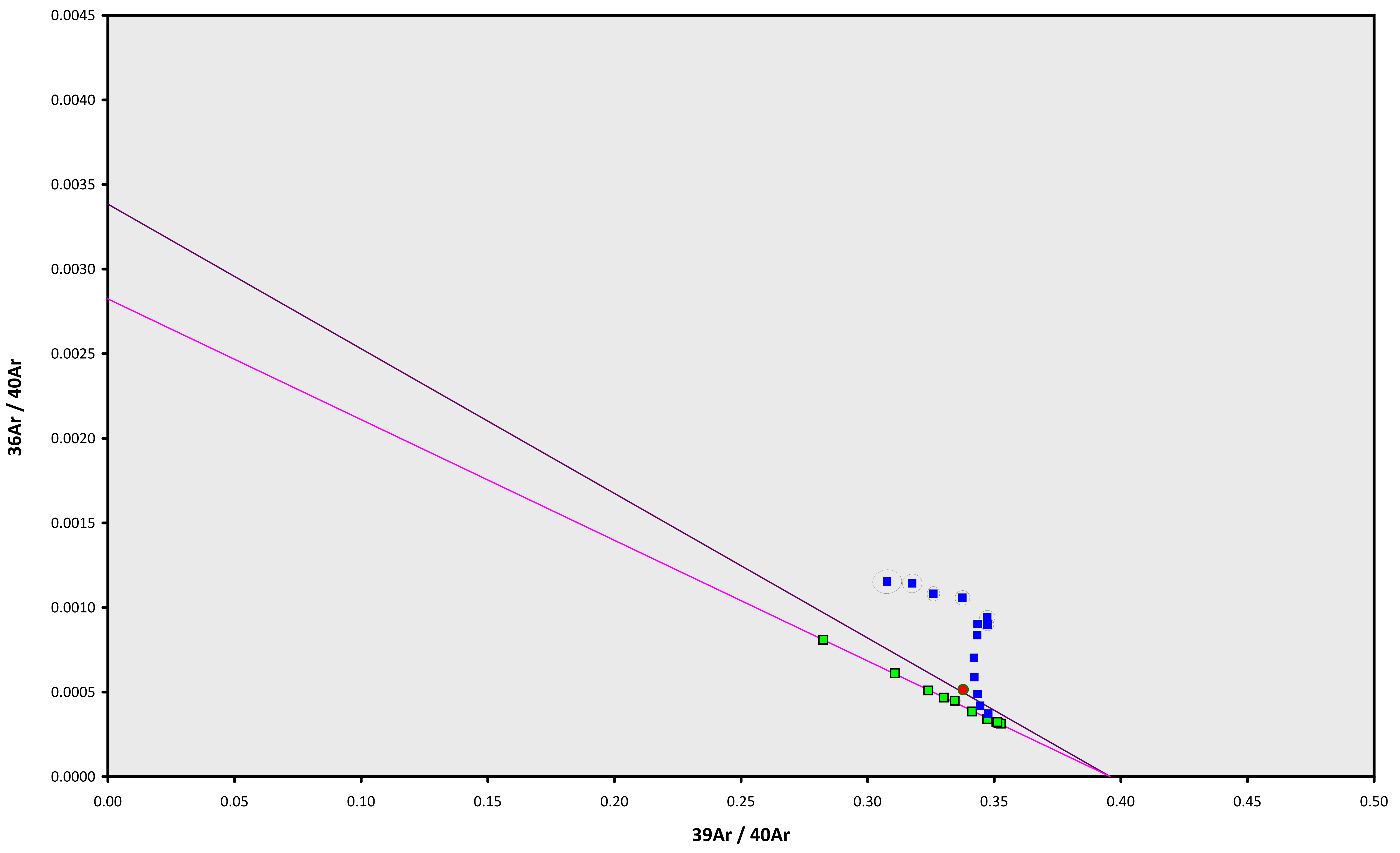

Ar-Ages in Ma

WEIGHTED PLATEAU
6.97 ± 0.02
TOTAL FUSION
6.68 ± 0.02
NORMAL ISOCHRON
6.97 ± 0.03
INVERSE ISOCHRON
6.97 ± 0.03

MSWD (PROBABILITY)
0.71 (70%)

SPREADING FACTOR
17.7%

40AR/36AR INTERCEPT
354.1 ± 8.0

Sample Info

Groundmass
Mozambique Ridge
Dan Miggins

IRR = 17-OSU-01 (1B38-17)
J = 0.00152826 ± 0.00000151

Incremental Heating			36Ar(a) [fA]	37Ar(ca) [fA]	38Ar(cl) [fA]	39Ar(k) [fA]	40Ar(r) [fA]	Age ± 2σ (Ma)	40Ar(r) (%)	39Ar(k) (%)	K/Ca ± 2σ
011_VU107-J-1	12 °C		0.2958851	34.9015	0.0000000	25.07765	24.92931	8.52 ± 0.32	22.00	8.59	0.309 ± 0.005
012_VU107-J-1	13 °C		0.0844360	49.1647	0.0000000	30.34999	26.85728	7.59 ± 0.11	51.56	10.40	0.265 ± 0.005
013_VU107-J-1	14 °C	4	0.0616547	72.5992	0.0000000	35.21543	30.27562	7.37 ± 0.09	62.15	12.07	0.209 ± 0.004
015_VU107-J-1	15 °C	4	0.0418076	85.5391	0.0000000	33.86943	29.08497	7.36 ± 0.08	69.92	11.61	0.170 ± 0.003
016_VU107-J-1	17 °C	4	0.0361941	78.4697	0.0033017	29.07523	24.42990	7.20 ± 0.09	69.28	9.96	0.159 ± 0.003
017_VU107-J-1	18 °C	4	0.0378311	78.9429	0.0123056	28.60286	23.75533	7.12 ± 0.09	67.73	9.80	0.156 ± 0.003
019_VU107-J-1	19 °C		0.0352459	64.3145	0.0151668	23.37092	19.10342	7.01 ± 0.10	64.44	8.01	0.156 ± 0.003
020_VU107-J-1	21 °C		0.0357881	63.7975	0.0227754	23.08901	18.20826	6.76 ± 0.13	62.98	7.91	0.156 ± 0.003
021_VU107-J-1	22 °C		0.0432599	66.0451	0.0302630	18.43942	14.23808	6.62 ± 0.16	52.40	6.32	0.120 ± 0.002
023_VU107-J-1	25 °C		0.0380105	87.1221	0.0313777	15.97360	11.65948	6.26 ± 0.16	50.65	5.47	0.079 ± 0.001
024_VU107-J-1	28 °C		0.0378179	116.9947	0.0315070	11.60080	7.85324	5.81 ± 0.31	41.00	3.98	0.043 ± 0.001
025_VU107-J-1	31 °C		0.0370329	110.9901	0.0228761	6.66849	4.35136	5.60 ± 0.43	28.23	2.29	0.026 ± 0.001
027_VU107-J-1	39 °C		0.0364656	129.2578	0.0196035	5.05897	3.31232	5.62 ± 0.59	23.32	1.73	0.017 ± 0.000
028_VU107-J-1	69 °C		0.0210744	114.1181	0.0112148	2.60625	1.69498	5.58 ± 1.02	21.22	0.89	0.010 ± 0.000
029_VU107-J-1	16 °C		0.0256379	102.4006	0.0103187	2.78866	1.88433	5.79 ± 1.25	19.75	0.96	0.012 ± 0.000
		∑	0.8681416	1254.6576	0.2107102	291.78670	241.63786				

Information on Analysis

Sample = 011_VU107-J-1
Material = groundmass
Location = MW14 DL2-2
Analyst = Klaudia Kuiper
Project = VU107
Mass Discrimination Law = LIN
Irradiation = VU107
J = 0.00468970 ± 0.00000469
FCs = 28.201 ± 0.023 Ma

Results	40(r)/39(k)	± 2σ		Age (Ma)	± 2σ	MSWD	39Ar(k) (%,n)	K/Ca ± 2σ
Age Plateau<br>Error Mean	0.84748	± 0.01424<br>± 1.68%		7.26	± 0.12<br>± 1.69%	7.94<br>0%	43.44<br>4	0.168 ± 0.021
			Full External Error		± 0.20	2.63	2σ Confidence Limit	
			Analytical Error		± 0.12	2.8176	Error Magnification	
Total Fusion Age	0.82813	± 0.00546<br>± 0.66%		7.10	± 0.05<br>± 0.69%		15	0.100 ± 0.000
			Full External Error		± 0.16			
			Analytical Error		± 0.05			

Normal Isochron			39(k)/36(a) ± 2σ	40(a+r)/36(a) ± 2σ	r.i.
011_VU107-J-1	12 °C		84.75 ± 0.98	382.81 ± 3.99	0.8881
012_VU107-J-1	13 °C		359.44 ± 5.22	616.64 ± 8.77	0.9728
013_VU107-J-1	14 °C	4	571.17 ± 10.55	789.61 ± 14.31	0.9754
015_VU107-J-1	15 °C	4	810.13 ± 20.65	994.25 ± 25.16	0.9901
016_VU107-J-1	17 °C	4	803.31 ± 20.55	973.53 ± 24.72	0.9897
017_VU107-J-1	18 °C	4	756.07 ± 18.84	926.49 ± 22.88	0.9888
019_VU107-J-1	19 °C		663.08 ± 16.86	840.56 ± 21.25	0.9889
020_VU107-J-1	21 °C		645.16 ± 20.37	807.34 ± 25.28	0.9884
021_VU107-J-1	22 °C		426.25 ± 10.96	627.69 ± 15.94	0.9845
023_VU107-J-1	25 °C		420.24 ± 10.73	605.30 ± 14.95	0.9638
024_VU107-J-1	28 °C		306.75 ± 11.43	506.22 ± 18.39	0.9734
025_VU107-J-1	31 °C		180.07 ± 5.91	416.06 ± 12.40	0.9056
027_VU107-J-1	39 °C		138.73 ± 4.50	389.39 ± 12.50	0.9858
028_VU107-J-1	69 °C		123.67 ± 6.40	378.99 ± 18.67	0.9463
029_VU107-J-1	16 °C		108.77 ± 6.48	372.06 ± 19.51	0.8756

Results	40(a)/36(a) ± 2σ		40(r)/39(k) ± 2σ		Age ± 2σ (Ma)		MSWD
Normal Isochron<br>Error Chron	334.01	± 74.30<br>± 22.24%	0.79763	± 0.10427<br>± 13.07%	6.84	± 0.89<br>± 13.05%	7.99<br>0%
					Full External Error	± 0.90	
					Analytical Error	± 0.89	

Statistics				
	2σ Confidence Limit	3.00	Convergence	0.000007391869
	Error Magnification	2.8267	Number of Iterations	29
	Number of Data Points	4	Calculated Line	Weighted York-2

Inverse Isochron			39(k)/40(a+r) ± 2σ	36(a)/40(a+r) ± 2σ	r.i.
011_VU107-J-1	12 °C		0.2213995 ± 0.0011764	0.00261224 ± 0.00002723	0.0303
012_VU107-J-1	13 °C		0.5829085 ± 0.0019626	0.00162170 ± 0.00002307	0.0291
013_VU107-J-1	14 °C	4	0.7233581 ± 0.0029489	0.00126645 ± 0.00002295	0.0240
015_VU107-J-1	15 °C	4	0.8148146 ± 0.0029117	0.00100579 ± 0.00002545	0.0177
016_VU107-J-1	17 °C	4	0.8251566 ± 0.0030247	0.00102719 ± 0.00002608	0.0197
017_VU107-J-1	18 °C	4	0.8160544 ± 0.0030407	0.00107934 ± 0.00002665	0.0158
019_VU107-J-1	19 °C		0.7888534 ± 0.0029784	0.00118968 ± 0.00003007	0.0344
020_VU107-J-1	21 °C		0.7991172 ± 0.0038320	0.00123864 ± 0.00003879	0.0225
021_VU107-J-1	22 °C		0.6790745 ± 0.0030599	0.00159315 ± 0.00004045	0.0167
023_VU107-J-1	25 °C		0.6942666 ± 0.0047223	0.00165206 ± 0.00004081	0.0148
024_VU107-J-1	28 °C		0.6059712 ± 0.0051699	0.00197543 ± 0.00007177	0.0059
025_VU107-J-1	31 °C		0.4327971 ± 0.0060266	0.00240350 ± 0.00007164	0.0053
027_VU107-J-1	39 °C		0.3562779 ± 0.0019415	0.00256809 ± 0.00008243	0.0220
028_VU107-J-1	69 °C		0.3263134 ± 0.0054632	0.00263860 ± 0.00012996	0.0145
029_VU107-J-1	16 °C		0.2923502 ± 0.0084189	0.00268775 ± 0.00014091	0.0078

Results	40(a)/36(a) ± 2σ	40(r)/39(k) ± 2σ	Age ± 2σ (Ma)	MSWD
Inverse Isochron<br>Error Chron	332.07 ± 74.98<br>± 22.58%	0.80114 ± 0.10238<br>± 12.78%	6.87 ± 0.88<br>± 12.76%<br>Full External Error ± 0.89<br>Analytical Error ± 0.88	8.13<br>0%

Statistics				
	2σ Confidence Limit	3.00	Convergence	0.0001136412
	Error Magnification	2.8506	Number of Iterations	3
	Number of Data Points	4	Calculated Line	Weighted York-2
	Spreading Factor	8.2%		

Relative Abundances			36Ar [fA]	%1σ	37Ar [fA]	%1σ	38Ar [fA]	%1σ	39Ar [fA]	%1σ	40Ar [fA]	%1σ	40(r)/39(k) ± 2σ	Age ± 2σ (Ma)	40Ar(r) (%)	39Ar(k) (%)	K/Ca ± 2σ
011_VU107-J-1	12 °C		0.3050991	0.500	34.9015	0.827	0.3455436	0.362	25.10114	0.257	113.29034	0.065	0.99408 ± 0.03796	8.52 ± 0.32	22.00	8.59	0.309 ± 0.005
012_VU107-J-1	13 °C		0.0974154	0.597	49.1647	0.874	0.3695274	0.309	30.38308	0.157	52.09258	0.059	0.88492 ± 0.01239	7.59 ± 0.11	51.56	10.40	0.265 ± 0.005
013_VU107-J-1	14 °C	4	0.0808209	0.641	72.5992	0.863	0.4291763	0.290	35.26429	0.192	48.71354	0.066	0.85973 ± 0.01024	7.37 ± 0.09	62.15	12.07	0.209 ± 0.004
015_VU107-J-1	15 °C	4	0.0643899	0.735	85.5391	0.816	0.4171610	0.315	33.92699	0.167	41.59616	0.063	0.85874 ± 0.00990	7.36 ± 0.08	69.92	11.61	0.170 ± 0.003
016_VU107-J-1	17 °C	4	0.0569115	0.711	78.4697	0.823	0.3622253	0.295	29.12804	0.170	35.26102	0.067	0.84023 ± 0.01001	7.20 ± 0.09	69.28	9.96	0.159 ± 0.003
017_VU107-J-1	18 °C	4	0.0586772	0.705	78.9429	0.813	0.3658174	0.265	28.65599	0.176	35.07479	0.060	0.83052 ± 0.01031	7.12 ± 0.09	67.73	9.80	0.156 ± 0.003
019_VU107-J-1	19 °C		0.0522314	0.783	64.3145	0.802	0.3048325	0.340	23.41420	0.165	29.64654	0.090	0.81740 ± 0.01193	7.01 ± 0.10	64.44	8.01	0.156 ± 0.003
020_VU107-J-1	21 °C		0.0526402	1.006	63.7975	0.856	0.3091293	0.300	23.13195	0.221	28.91300	0.092	0.78861 ± 0.01509	6.76 ± 0.13	62.98	7.91	0.156 ± 0.003
021_VU107-J-1	22 °C		0.0607085	0.847	66.0451	0.886	0.2617189	0.355	18.48387	0.214	27.16961	0.069	0.77215 ± 0.01824	6.62 ± 0.16	52.40	6.32	0.120 ± 0.002
023_VU107-J-1	25 °C		0.0610239	0.660	87.1221	0.820	0.2319831	0.440	16.03224	0.330	23.02162	0.079	0.72992 ± 0.01837	6.26 ± 0.16	50.65	5.47	0.079 ± 0.001
024_VU107-J-1	28 °C		0.0687177	0.886	116.9947	0.806	0.1791214	0.485	11.67954	0.418	19.15412	0.068	0.67696 ± 0.03592	5.81 ± 0.31	41.00	3.98	0.043 ± 0.001
025_VU107-J-1	31 °C		0.0663439	0.695	110.9901	0.808	0.1106122	0.384	6.74319	0.685	15.41363	0.074	0.65253 ± 0.05042	5.60 ± 0.43	28.23	2.29	0.026 ± 0.001
027_VU107-J-1	39 °C		0.0705979	0.664	129.2578	0.797	0.0877413	0.384	5.14596	0.249	14.20385	0.098	0.65474 ± 0.06939	5.62 ± 0.59	23.32	1.73	0.017 ± 0.000
028_VU107-J-1	69 °C		0.0512063	0.806	114.1181	0.817	0.0467490	0.670	2.68306	0.795	7.98921	0.173	0.65035 ± 0.11967	5.58 ± 1.02	21.22	0.89	0.010 ± 0.000
029_VU107-J-1	16 °C		0.0526760	1.158	102.4006	0.809	0.0489221	0.655	2.85758	1.395	9.54117	0.171	0.67571 ± 0.14547	5.79 ± 1.25	19.75	0.96	0.012 ± 0.000
		Σ	1.1994600	0.199	1254.6576	0.221	3.8702607	0.094	292.63109	0.063	501.08117	0.022					

Information on Analysis and Constants Used in Calculations

Sample = 011_VU107-J-1
Material = groundmass
Location = MW14 DL2-2
Analyst = Klaudia Kuiper
Project = VU107
Mass Discrimination Law = LIN
Irradiation = VU107
J = 0.00468970 ± 0.00000469
FCs = 28.201 ± 0.023 Ma
IGSN = Undefined
Preferred Age = Undefined
Classification = Undefined
Experiment Type = Undefined
Extraction Method = Undefined
Heating = 720 sec
Isolation = 18.00 min
Instrument = HELIX
Lithology = Undefined
Lat-Lon = Undefined - Undefined
Feature = Undefined

Age Equations = Min et al. (2000)
Negative Intensities = Allowed
Decay Constant 40K = 5.460 ± 0.053 E-10 1/a
Decay Constant 39Ar = 2.940 ± 0.016 E-07 1/h
Decay Constant 37Ar = 8.230 ± 0.012 E-04 1/h
Decay Constant 36Cl = 2.257 ± 0.015 E-06 1/a
Decay Activity 40K(EC,β⁺) = 3.310 ± 0.030 1/gs
Decay Activity 40K(β⁻) = 27.890 ± 0.150 1/gs
Atmospheric Ratio 40/36(a) = 298.56 ± 0.31
Atmospheric Ratio 38/36(a) = 0.1885 ± 0.0003
Production Ratio 39/37(ca) = 0.000673 ± 0.000004
Production Ratio 36/37(ca) = 0.000264 ± 0.000002
Production Ratio 40/39(k) = 0.000860 ± 0.000070
Production Ratio 38/39(k) = 0.012110 ± 0.000030
Production Ratio 36/38(cl) = 262.80 ± 1.71
Scaling Ratio K/Ca = 0.430
Abundance Ratio 40K/K = 1.1700 ± 0.0100 E-04
Atomic Weight K = 39.0983 ± 0.0001 g

Results

Results	40(a)/36(a) ± 2σ	40(r)/39(k) ± 2σ	Age ± 2σ (Ma)	MSWD	39Ar(k) (%,n)	K/Ca ± 2σ
**Age Plateau** Error Mean		0.84748 ± 0.01424 ± 1.68%	7.26 ± 0.12 ± 1.69%	7.94 0%	43.44 4	0.168 ± 0.021
			Full External Error ± 0.20	2.63	2σ Confidence Limit	
			Analytical Error ± 0.12	2.8176	Error Magnification	
**Total Fusion Age**		0.82813 ± 0.00546 ± 0.66%	7.10 ± 0.05 ± 0.69%		15	0.100 ± 0.000
			Full External Error ± 0.16			
			Analytical Error ± 0.05			
**Normal Isochron** Error Chron	334.01 ± 74.30 ± 22.24%	0.79763 ± 0.10427 ± 13.07%	6.84 ± 0.89 ± 13.05%	7.99 0%	43.44 4	
			Full External Error ± 0.90	3.00	2σ Confidence Limit	
			Analytical Error ± 0.89	2.8267	Error Magnification	
				29	Number of Iterations	
				0.0000073919	Convergence	
**Inverse Isochron** Error Chron	332.07 ± 74.98 ± 22.58%	0.80114 ± 0.10238 ± 12.78%	6.87 ± 0.88 ± 12.76%	8.13 0%	43.44 4	
			Full External Error ± 0.89	3.00	2σ Confidence Limit	
			Analytical Error ± 0.88	2.8506	Error Magnification	
				3	Number of Iterations	
				0.0001136412	Convergence	
				8%	Spreading Factor	

Degassing Patterns			36Ar(a) [fA]	%1σ	36Ar(c) [fA]	%1σ	36Ar(ca) [fA]	%1σ	36Ar(cl) [fA]	%1σ	37Ar(ca) [fA]	%1σ	38Ar(a) [fA]	%1σ	38Ar(c) [fA]	%1σ
011_VU107-J-1	12 °C		0.2958851	0.52	0.0000000	0.00	0.0092140	1.05	0.0000000	0.00	34.9015	0.83	0.0557743	0.54	0.0000000	0.00
012_VU107-J-1	13 °C		0.0844360	0.71	0.0000000	0.00	0.0129795	1.08	0.0000000	0.00	49.1647	0.87	0.0159162	0.73	0.0000000	0.00
013_VU107-J-1	14 °C	4	0.0616547	0.90	0.0000000	0.00	0.0191662	1.07	0.0000000	0.00	72.5992	0.86	0.0116219	0.92	0.0000000	0.00
015_VU107-J-1	15 °C	4	0.0418076	1.26	0.0000000	0.00	0.0225823	1.04	0.0000000	0.00	85.5391	0.82	0.0078807	1.27	0.0000000	0.00
016_VU107-J-1	17 °C	4	0.0361941	1.27	0.0000000	0.00	0.0207160	1.04	0.0000014	51.83	78.4697	0.82	0.0068226	1.28	0.0000000	0.00
017_VU107-J-1	18 °C	4	0.0378311	1.23	0.0000000	0.00	0.0208409	1.03	0.0000052	13.42	78.9429	0.81	0.0071312	1.24	0.0000000	0.00
019_VU107-J-1	19 °C		0.0352459	1.26	0.0000000	0.00	0.0169790	1.03	0.0000064	9.91	64.3145	0.80	0.0066439	1.27	0.0000000	0.00
020_VU107-J-1	21 °C		0.0357881	1.56	0.0000000	0.00	0.0168425	1.07	0.0000096	6.55	63.7975	0.86	0.0067461	1.57	0.0000000	0.00
021_VU107-J-1	22 °C		0.0432599	1.27	0.0000000	0.00	0.0174359	1.09	0.0000127	4.43	66.0451	0.89	0.0081545	1.28	0.0000000	0.00
023_VU107-J-1	25 °C		0.0380105	1.23	0.0000000	0.00	0.0230002	1.04	0.0000132	4.52	87.1221	0.82	0.0071650	1.24	0.0000000	0.00
024_VU107-J-1	28 °C		0.0378179	1.82	0.0000000	0.00	0.0308866	1.03	0.0000133	3.84	116.9947	0.81	0.0071287	1.82	0.0000000	0.00
025_VU107-J-1	31 °C		0.0370329	1.49	0.0000000	0.00	0.0293014	1.03	0.0000096	3.49	110.9901	0.81	0.0069807	1.50	0.0000000	0.00
027_VU107-J-1	39 °C		0.0364656	1.60	0.0000000	0.00	0.0341240	1.02	0.0000083	2.48	129.2578	0.80	0.0068738	1.61	0.0000000	0.00
028_VU107-J-1	69 °C		0.0210744	2.46	0.0000000	0.00	0.0301272	1.04	0.0000047	3.98	114.1181	0.82	0.0039725	2.46	0.0000000	0.00
029_VU107-J-1	16 °C		0.0256379	2.62	0.0000000	0.00	0.0270338	1.03	0.0000043	5.95	102.4006	0.81	0.0048327	2.62	0.0000000	0.00
		Σ	0.8681416	0.30	0.0000000	0.00	0.3312296	0.28	0.0000887	1.95	1254.6576	0.22	0.1636447	0.30	0.0000000	0.00
		Σ							1.1994600	0.23	1254.6576	0.22				

Degassing Patterns	38Ar(k) [fA]	%1σ	38Ar(ca) [fA]	%1σ	38Ar(cl) [fA]	%1σ	39Ar(k) [fA]	%1σ	39Ar(ca) [fA]	%1σ	40Ar(r) [fA]	%1σ	40Ar(a) [fA]	%1σ	40Ar(c) [fA]	%1σ	40Ar(k) [fA]	%1σ
011_VU107-J-1	0.3036903	0.36	0.0000000	0.00	0.0000000	0.00	25.07765	0.26	0.0234887	0.99	24.92931	1.89	88.33946	0.53	0.0000000	0.00	0.0215668	8.10
012_VU107-J-1	0.3675384	0.30	0.0000000	0.00	0.0000000	0.00	30.34999	0.16	0.0330879	1.03	26.85728	0.68	25.20920	0.72	0.0000000	0.00	0.0261010	8.10
013_VU107-J-1	0.4264588	0.32	0.0000000	0.00	0.0000000	0.00	35.21543	0.19	0.0488593	1.02	30.27562	0.56	18.40763	0.91	0.0000000	0.00	0.0302853	8.10
015_VU107-J-1	0.4101588	0.30	0.0000000	0.00	0.0000000	0.00	33.86943	0.17	0.0575678	0.98	29.08497	0.55	12.48207	1.27	0.0000000	0.00	0.0291277	8.10
016_VU107-J-1	0.3521010	0.30	0.0000000	0.00	0.0033017	51.83	29.07523	0.17	0.0528101	0.99	24.42990	0.57	10.80612	1.27	0.0000000	0.00	0.0250047	8.10
017_VU107-J-1	0.3463807	0.31	0.0000000	0.00	0.0123056	13.45	28.60286	0.18	0.0531286	0.98	23.75533	0.60	11.29486	1.24	0.0000000	0.00	0.0245985	8.10
019_VU107-J-1	0.2830218	0.30	0.0000000	0.00	0.0151668	9.95	23.37092	0.17	0.0432837	0.97	19.10342	0.71	10.52303	1.26	0.0000000	0.00	0.0200990	8.10
020_VU107-J-1	0.2796079	0.33	0.0000000	0.00	0.0227754	6.62	23.08901	0.22	0.0429357	1.02	18.20826	0.93	10.68489	1.57	0.0000000	0.00	0.0198565	8.10
021_VU107-J-1	0.2233014	0.33	0.0000000	0.00	0.0302630	4.52	18.43942	0.21	0.0444484	1.04	14.23808	1.16	12.91567	1.27	0.0000000	0.00	0.0158579	8.10
023_VU107-J-1	0.1934404	0.41	0.0000000	0.00	0.0313777	4.61	15.97360	0.33	0.0586331	0.99	11.65948	1.21	11.34840	1.24	0.0000000	0.00	0.0137373	8.11
024_VU107-J-1	0.1404857	0.49	0.0000000	0.00	0.0315070	3.95	11.60080	0.42	0.0787374	0.98	7.85324	2.62	11.29090	1.82	0.0000000	0.00	0.0099767	8.11
025_VU107-J-1	0.0807554	0.74	0.0000000	0.00	0.0228761	3.61	6.66849	0.69	0.0746963	0.98	4.35136	3.80	11.05653	1.49	0.0000000	0.00	0.0057349	8.13
027_VU107-J-1	0.0612641	0.36	0.0000000	0.00	0.0196035	2.64	5.05897	0.25	0.0869905	0.97	3.31232	5.29	10.88718	1.61	0.0000000	0.00	0.0043507	8.10
028_VU107-J-1	0.0315617	0.86	0.0000000	0.00	0.0112148	4.09	2.60625	0.82	0.0768015	0.99	1.69498	9.16	6.29199	2.46	0.0000000	0.00	0.0022414	8.14
029_VU107-J-1	0.0337707	1.45	0.0000000	0.00	0.0103187	6.02	2.78866	1.43	0.0689156	0.98	1.88433	10.67	7.65444	2.62	0.0000000	0.00	0.0023982	8.23
Σ	3.5335370	0.10	0.0000000	0.00	0.2107102	1.97	291.78670	0.06	0.8443846	0.27	241.63786	0.32	259.19237	0.30	0.0000000	0.00	0.2509366	2.40
Σ					3.9078919	0.14			292.63109	0.06							501.08117	0.22

Additional Parameters			40Ar/39Ar	1σ	37Ar/39Ar	1σ	36Ar/39Ar	1σ	Time (days)	37Ar (decay)	39Ar (decay)	40Ar (moles)
011_VU107-J-1	12 °C		4.513355	0.011980	1.390434	0.012046	0.012155	0.000068	258.982	171.090426	1.00183873	1.133E-11
012_VU107-J-1	13 °C		1.714526	0.002883	1.618162	0.014365	0.003206	0.000020	259.003	171.160844	1.00183888	5.209E-12
013_VU107-J-1	14 °C	4	1.381385	0.002811	2.058717	0.018208	0.002292	0.000015	259.024	171.233640	1.00183903	4.871E-12
015_VU107-J-1	15 °C	4	1.226049	0.002186	2.521270	0.020993	0.001898	0.000014	259.066	171.374624	1.00183932	4.160E-12
016_VU107-J-1	17 °C	4	1.210552	0.002214	2.693958	0.022644	0.001954	0.000014	259.087	171.445159	1.00183947	3.526E-12
017_VU107-J-1	18 °C	4	1.223995	0.002275	2.754849	0.022926	0.002048	0.000015	259.108	171.518076	1.00183962	3.507E-12
019_VU107-J-1	19 °C		1.266178	0.002385	2.746817	0.022506	0.002231	0.000018	259.150	171.659293	1.00183992	2.965E-12
020_VU107-J-1	21 °C		1.249917	0.002991	2.757982	0.024373	0.002276	0.000023	259.172	171.732301	1.00184007	2.891E-12
021_VU107-J-1	22 °C		1.469909	0.003303	3.573122	0.032572	0.003284	0.000029	259.193	171.805340	1.00184022	2.717E-12
023_VU107-J-1	25 °C		1.435958	0.004866	5.434180	0.048022	0.003806	0.000028	259.234	171.944436	1.00184051	2.302E-12
024_VU107-J-1	28 °C		1.639973	0.006948	10.017069	0.090936	0.005884	0.000058	259.256	172.017565	1.00184066	1.915E-12
025_VU107-J-1	31 °C		2.285807	0.015738	16.459590	0.174256	0.009839	0.000096	259.277	172.090725	1.00184082	1.541E-12
027_VU107-J-1	39 °C		2.760195	0.007396	25.118307	0.209813	0.013719	0.000097	259.319	172.232414	1.00184111	1.420E-12
028_VU107-J-1	69 °C		2.977653	0.024229	42.532883	0.484984	0.019085	0.000216	259.340	172.303302	1.00184126	7.989E-13
029_VU107-J-1	16 °C		3.338902	0.046926	35.834770	0.577835	0.018434	0.000334	259.361	172.376584	1.00184141	9.541E-13

Procedure Blanks		36Ar [fA]	1σ	37Ar [fA]	1σ	38Ar [fA]	1σ	39Ar [fA]	1σ	40Ar [fA]	1σ
011_VU107-J-1	12 °C	0.0096788	0.0001132	0.0121369	0.0000913	0.0061327	0.0001035	0.2153791	0.0058224	2.9155395	0.0056492
012_VU107-J-1	13 °C	0.0096788	0.0001132	0.0121369	0.0000913	0.0061327	0.0001035	0.2153791	0.0058224	2.9155395	0.0056492
013_VU107-J-1	14 °C	0.0096788	0.0001132	0.0121369	0.0000913	0.0061327	0.0001035	0.2153791	0.0058224	2.9155395	0.0056492
015_VU107-J-1	15 °C	0.0095560	0.0001311	0.0124240	0.0001547	0.0062651	0.0001203	0.2303195	0.0161037	2.8869061	0.0043034
016_VU107-J-1	17 °C	0.0095560	0.0001311	0.0124240	0.0001547	0.0062651	0.0001203	0.2303195	0.0161037	2.8869061	0.0043034
017_VU107-J-1	18 °C	0.0095560	0.0001311	0.0124240	0.0001547	0.0062651	0.0001203	0.2303195	0.0161037	2.8869061	0.0043034
019_VU107-J-1	19 °C	0.0094880	0.0001873	0.0124973	0.0001287	0.0060000	0.0000975	0.2019398	0.0062571	2.8829288	0.0053294
020_VU107-J-1	21 °C	0.0094880	0.0001873	0.0124973	0.0001287	0.0060000	0.0000975	0.2019398	0.0062571	2.8829288	0.0053294
021_VU107-J-1	22 °C	0.0094880	0.0001873	0.0124973	0.0001287	0.0060000	0.0000975	0.2019398	0.0062571	2.8829288	0.0053294
023_VU107-J-1	25 °C	0.0091710	0.0001391	0.0125903	0.0001253	0.0059457	0.0001276	0.2105255	0.0438790	2.8156003	0.0039253
024_VU107-J-1	28 °C	0.0091710	0.0001391	0.0125903	0.0001253	0.0059457	0.0001276	0.2105255	0.0438790	2.8156003	0.0039253
025_VU107-J-1	31 °C	0.0091710	0.0001391	0.0125903	0.0001253	0.0059457	0.0001276	0.2105255	0.0438790	2.8156003	0.0039253
027_VU107-J-1	39 °C	0.0089608	0.0001526	0.0129043	0.0001257	0.0057682	0.0000993	0.2234395	0.0053739	2.7509641	0.0065072
028_VU107-J-1	69 °C	0.0089608	0.0001526	0.0129043	0.0001257	0.0057682	0.0000993	0.2234395	0.0053739	2.7509641	0.0065072
029_VU107-J-1	16 °C	0.0089608	0.0001526	0.0129043	0.0001257	0.0057682	0.0000993	0.2234395	0.0053739	2.7509641	0.0065072

Intercept Values		36Ar [fA]	1σ	r2		37Ar [fA]	1σ	r2	
011_VU107-J-1	12 °C	0.2945918	0.0007640	0.9700	LIN 15 of 15	0.2060	0.0005	0.9800	LIN 15 of 15
012_VU107-J-1	13 °C	0.1006490	0.0003686	0.8400	LIN 15 of 15	0.2851	0.0011	0.9600	LIN 15 of 15
013_VU107-J-1	14 °C	0.0851524	0.0003465	0.8300	LIN 15 of 15	0.4151	0.0015	0.9700	LIN 15 of 15
015_VU107-J-1	15 °C	0.0696857	0.0003382	0.5900	LIN 15 of 15	0.4868	0.0011	0.9800	LIN 15 of 15
016_VU107-J-1	17 °C	0.0627021	0.0002752	0.7800	LIN 15 of 15	0.4474	0.0011	0.9800	LIN 15 of 15
017_VU107-J-1	18 °C	0.0643510	0.0002809	0.8300	LIN 15 of 15	0.4498	0.0010	0.9900	LIN 15 of 15
019_VU107-J-1	19 °C	0.0582636	0.0002617	0.8300	LIN 15 of 15	0.3686	0.0006	0.9900	LIN 15 of 15
020_VU107-J-1	21 °C	0.0586454	0.0004084	0.6300	LIN 15 of 15	0.3656	0.0012	0.9700	LIN 15 of 15
021_VU107-J-1	22 °C	0.0661799	0.0003723	0.6900	LIN 15 of 15	0.3778	0.0015	0.9600	LIN 15 of 15
023_VU107-J-1	25 °C	0.0661574	0.0002543	0.8900	LIN 15 of 15	0.4941	0.0012	0.9900	LIN 15 of 15
024_VU107-J-1	28 °C	0.0733422	0.0004803	0.7600	LIN 15 of 15	0.6590	0.0012	0.9900	LIN 15 of 15
025_VU107-J-1	31 °C	0.0711254	0.0003134	0.8600	LIN 15 of 15	0.6255	0.0012	0.9900	LIN 15 of 15
027_VU107-J-1	39 °C	0.0748878	0.0003020	0.8800	LIN 15 of 15	0.7262	0.0010	0.9900	LIN 15 of 15
028_VU107-J-1	69 °C	0.0567792	0.0002910	0.8500	LIN 15 of 15	0.6424	0.0015	0.9900	LIN 15 of 15
029_VU107-J-1	16 °C	0.0581516	0.0005082	0.5600	LIN 15 of 15	0.5775	0.0011	0.9900	LIN 15 of 15

Intercept Values	38Ar [fA]	1σ	r2		39Ar [fA]	1σ	r2		40Ar [fA]	1σ	r2	
011_VU107-J-1	0.3402325	0.0009966	0.9700	LIN 15 of 15	24.85628	0.05818	0.9900	EXP 15 of 15	116.20588	0.07310	1.0000	EXP 15 of 15
012_VU107-J-1	0.3634220	0.0008251	0.9800	LIN 15 of 15	30.04138	0.03587	1.0000	EXP 15 of 15	55.00812	0.03013	1.0000	EXP 15 of 15
013_VU107-J-1	0.4210954	0.0008493	0.9900	LIN 15 of 15	34.83308	0.05664	1.0000	EXP 14 of 15	51.62908	0.03182	1.0000	EXP 15 of 15
015_VU107-J-1	0.4096105	0.0009617	0.9800	LIN 15 of 15	33.53524	0.04149	1.0000	EXP 15 of 15	44.48307	0.02587	1.0000	EXP 15 of 15
016_VU107-J-1	0.3564941	0.0007371	0.9900	LIN 15 of 15	28.82427	0.03590	1.0000	EXP 15 of 15	38.14792	0.02338	1.0000	EXP 15 of 15
017_VU107-J-1	0.3599673	0.0005902	0.9900	LIN 15 of 15	28.36088	0.03745	1.0000	EXP 14 of 15	37.96170	0.02058	1.0000	EXP 15 of 15
019_VU107-J-1	0.3007370	0.0007968	0.9800	LIN 15 of 15	23.18681	0.02964	1.0000	EXP 15 of 15	32.52947	0.02625	1.0000	EXP 15 of 15
020_VU107-J-1	0.3048915	0.0006524	0.9900	LIN 15 of 15	22.90973	0.04435	0.9900	EXP 15 of 15	31.79593	0.02594	1.0000	EXP 15 of 15
021_VU107-J-1	0.2590512	0.0007311	0.9800	LIN 15 of 15	18.34688	0.03375	0.9900	EXP 15 of 15	30.05254	0.01795	1.0000	LIN 14 of 15
023_VU107-J-1	0.2302459	0.0008662	0.9600	LIN 15 of 15	15.94878	0.02278	1.0000	EXP 15 of 15	25.83722	0.01767	1.0000	EXP 15 of 15
024_VU107-J-1	0.1791349	0.0007519	0.9500	LIN 15 of 15	11.67589	0.01560	1.0000	EXP 15 of 15	21.96972	0.01234	1.0000	EXP 15 of 15
025_VU107-J-1	0.1128946	0.0003238	0.9700	LIN 15 of 15	6.83006	0.00918	1.0000	EXP 15 of 15	18.22923	0.01071	0.9900	EXP 15 of 15
027_VU107-J-1	0.0906037	0.0002580	0.9600	LIN 15 of 15	5.27503	0.01022	0.9900	EXP 15 of 15	16.95481	0.01229	0.9700	EXP 15 of 15
028_VU107-J-1	0.0509690	0.0002708	0.8300	LIN 15 of 15	2.85729	0.02007	0.7300	EXP 15 of 15	10.74017	0.01220	0.9500	EXP 15 of 15
029_VU107-J-1	0.0530701	0.0002773	0.8700	LIN 15 of 15	3.02861	0.03866	0.5600	EXP 15 of 15	12.29213	0.01498	0.8300	EXP 15 of 15

Sample Parameters		Sample	Material	Location	Analyst	Temp	Standard (in Ma)	%1σ	J	%1σ	MDF	%1σ	Volume Ratio	Sensitivity (mol/volt)	Day	Month	Year	Hour	Min	Resist	Irradiation	Project	Experiment	Nmb	Standard Name
011_VU107-J-1	12 °C	011_VU107-J-1	groundmass	MW14 DL2-2	Klaudia Kuiper	11.7	28.201	0.08	0.0046897	0.1	0.983438	0.1	1	1E-13	17	NOV	2016	23	33	1	VU107	VU107	VU107-J-1	01	FCs
012_VU107-J-1	13 °C	012_VU107-J-1	groundmass	MW14 DL2-2	Klaudia Kuiper	13.1	28.201	0.08	0.0046897	0.1	0.983438	0.1	1	1E-13	18	NOV	2016	0	3	1	VU107	VU107	VU107-J-1	01	FCs
013_VU107-J-1	14 °C	013_VU107-J-1	groundmass	MW14 DL2-2	Klaudia Kuiper	14.3	28.201	0.08	0.0046897	0.1	0.983438	0.1	1	1E-13	18	NOV	2016	0	34	1	VU107	VU107	VU107-J-1	01	FCs
015_VU107-J-1	15 °C	015_VU107-J-1	groundmass	MW14 DL2-2	Klaudia Kuiper	15.3	28.201	0.08	0.0046897	0.1	0.983438	0.1	1	1E-13	18	NOV	2016	1	34	1	VU107	VU107	VU107-J-1	01	FCs
016_VU107-J-1	17 °C	016_VU107-J-1	groundmass	MW14 DL2-2	Klaudia Kuiper	16.5	28.201	0.08	0.0046897	0.1	0.983438	0.1	1	1E-13	18	NOV	2016	2	4	1	VU107	VU107	VU107-J-1	01	FCs
017_VU107-J-1	18 °C	017_VU107-J-1	groundmass	MW14 DL2-2	Klaudia Kuiper	17.6	28.201	0.08	0.0046897	0.1	0.983438	0.1	1	1E-13	18	NOV	2016	2	35	1	VU107	VU107	VU107-J-1	01	FCs
019_VU107-J-1	19 °C	019_VU107-J-1	groundmass	MW14 DL2-2	Klaudia Kuiper	18.9	28.201	0.08	0.0046897	0.1	0.983438	0.1	1	1E-13	18	NOV	2016	3	35	1	VU107	VU107	VU107-J-1	01	FCs
020_VU107-J-1	21 °C	020_VU107-J-1	groundmass	MW14 DL2-2	Klaudia Kuiper	20.5	28.201	0.08	0.0046897	0.1	0.983438	0.1	1	1E-13	18	NOV	2016	4	6	1	VU107	VU107	VU107-J-1	01	FCs
021_VU107-J-1	22 °C	021_VU107-J-1	groundmass	MW14 DL2-2	Klaudia Kuiper	22.4	28.201	0.08	0.0046897	0.1	0.983438	0.1	1	1E-13	18	NOV	2016	4	37	1	VU107	VU107	VU107-J-1	01	FCs
023_VU107-J-1	25 °C	023_VU107-J-1	groundmass	MW14 DL2-2	Klaudia Kuiper	24.6	28.201	0.08	0.0046897	0.1	0.983438	0.1	1	1E-13	18	NOV	2016	5	36	1	VU107	VU107	VU107-J-1	01	FCs
024_VU107-J-1	28 °C	024_VU107-J-1	groundmass	MW14 DL2-2	Klaudia Kuiper	27.6	28.201	0.08	0.0046897	0.1	0.983438	0.1	1	1E-13	18	NOV	2016	6	7	1	VU107	VU107	VU107-J-1	01	FCs
025_VU107-J-1	31 °C	025_VU107-J-1	groundmass	MW14 DL2-2	Klaudia Kuiper	31.2	28.201	0.08	0.0046897	0.1	0.983438	0.1	1	1E-13	18	NOV	2016	6	38	1	VU107	VU107	VU107-J-1	01	FCs
027_VU107-J-1	39 °C	027_VU107-J-1	groundmass	MW14 DL2-2	Klaudia Kuiper	38.7	28.201	0.08	0.0046897	0.1	0.983438	0.1	1	1E-13	18	NOV	2016	7	38	1	VU107	VU107	VU107-J-1	01	FCs
028_VU107-J-1	69 °C	028_VU107-J-1	groundmass	MW14 DL2-2	Klaudia Kuiper	68.6	28.201	0.08	0.0046897	0.1	0.983438	0.1	1	1E-13	18	NOV	2016	8	8	1	VU107	VU107	VU107-J-1	01	FCs
029_VU107-J-1	16 °C	029_VU107-J-1	groundmass	MW14 DL2-2	Klaudia Kuiper	16	28.201	0.08	0.0046897	0.1	0.983438	0.1	1	1E-13	18	NOV	2016	8	39	1	VU107	VU107	VU107-J-1	01	FCs

Irradiation Constants		40/36(a)	%1σ	40/36(c)	%1σ	38/36(a)	%1σ	38/36(c)	%1σ	39/37(ca)	%1σ	38/37(ca)	%1σ	36/37(ca)	%1σ	40/39(k)	%1σ	38/39(k)	%1σ	36/38(cl)	%1σ	K/Ca	%1σ	K/Cl	%1σ	Ca/Cl	%1σ
011_VU107-J-1	12 °C	298.56	0.104	0.018	35	0.1885	0.159	1.493	3	0.000673	0.55	0	0	0.000264	0.64	0.00086	8.1	0.01211	0.25	0	0	0.43	0	0	0	0	0
012_VU107-J-1	13 °C	298.56	0.104	0.018	35	0.1885	0.159	1.493	3	0.000673	0.55	0	0	0.000264	0.64	0.00086	8.1	0.01211	0.25	0	0	0.43	0	0	0	0	0
013_VU107-J-1	14 °C	298.56	0.104	0.018	35	0.1885	0.159	1.493	3	0.000673	0.55	0	0	0.000264	0.64	0.00086	8.1	0.01211	0.25	0	0	0.43	0	0	0	0	0
015_VU107-J-1	15 °C	298.56	0.104	0.018	35	0.1885	0.159	1.493	3	0.000673	0.55	0	0	0.000264	0.64	0.00086	8.1	0.01211	0.25	0	0	0.43	0	0	0	0	0
016_VU107-J-1	17 °C	298.56	0.104	0.018	35	0.1885	0.159	1.493	3	0.000673	0.55	0	0	0.000264	0.64	0.00086	8.1	0.01211	0.25	0	0	0.43	0	0	0	0	0
017_VU107-J-1	18 °C	298.56	0.104	0.018	35	0.1885	0.159	1.493	3	0.000673	0.55	0	0	0.000264	0.64	0.00086	8.1	0.01211	0.25	0	0	0.43	0	0	0	0	0
019_VU107-J-1	19 °C	298.56	0.104	0.018	35	0.1885	0.159	1.493	3	0.000673	0.55	0	0	0.000264	0.64	0.00086	8.1	0.01211	0.25	0	0	0.43	0	0	0	0	0
020_VU107-J-1	21 °C	298.56	0.104	0.018	35	0.1885	0.159	1.493	3	0.000673	0.55	0	0	0.000264	0.64	0.00086	8.1	0.01211	0.25	0	0	0.43	0	0	0	0	0
021_VU107-J-1	22 °C	298.56	0.104	0.018	35	0.1885	0.159	1.493	3	0.000673	0.55	0	0	0.000264	0.64	0.00086	8.1	0.01211	0.25	0	0	0.43	0	0	0	0	0
023_VU107-J-1	25 °C	298.56	0.104	0.018	35	0.1885	0.159	1.493	3	0.000673	0.55	0	0	0.000264	0.64	0.00086	8.1	0.01211	0.25	0	0	0.43	0	0	0	0	0
024_VU107-J-1	28 °C	298.56	0.104	0.018	35	0.1885	0.159	1.493	3	0.000673	0.55	0	0	0.000264	0.64	0.00086	8.1	0.01211	0.25	0	0	0.43	0	0	0	0	0
025_VU107-J-1	31 °C	298.56	0.104	0.018	35	0.1885	0.159	1.493	3	0.000673	0.55	0	0	0.000264	0.64	0.00086	8.1	0.01211	0.25	0	0	0.43	0	0	0	0	0
027_VU107-J-1	39 °C	298.56	0.104	0.018	35	0.1885	0.159	1.493	3	0.000673	0.55	0	0	0.000264	0.64	0.00086	8.1	0.01211	0.25	0	0	0.43	0	0	0	0	0
028_VU107-J-1	69 °C	298.56	0.104	0.018	35	0.1885	0.159	1.493	3	0.000673	0.55	0	0	0.000264	0.64	0.00086	8.1	0.01211	0.25	0	0	0.43	0	0	0	0	0
029_VU107-J-1	16 °C	298.56	0.104	0.018	35	0.1885	0.159	1.493	3	0.000673	0.55	0	0	0.000264	0.64	0.00086	8.1	0.01211	0.25	0	0	0.43	0	0	0	0	0

Geochronology laboratory
Vrije Universiteit Amsterdam, Amsterdam, The Netherlands

VU107-J1_1_CORR_MW14 DL2-2.AGE >>> 011_VU107-J-1 >>> VU107 PROJECT

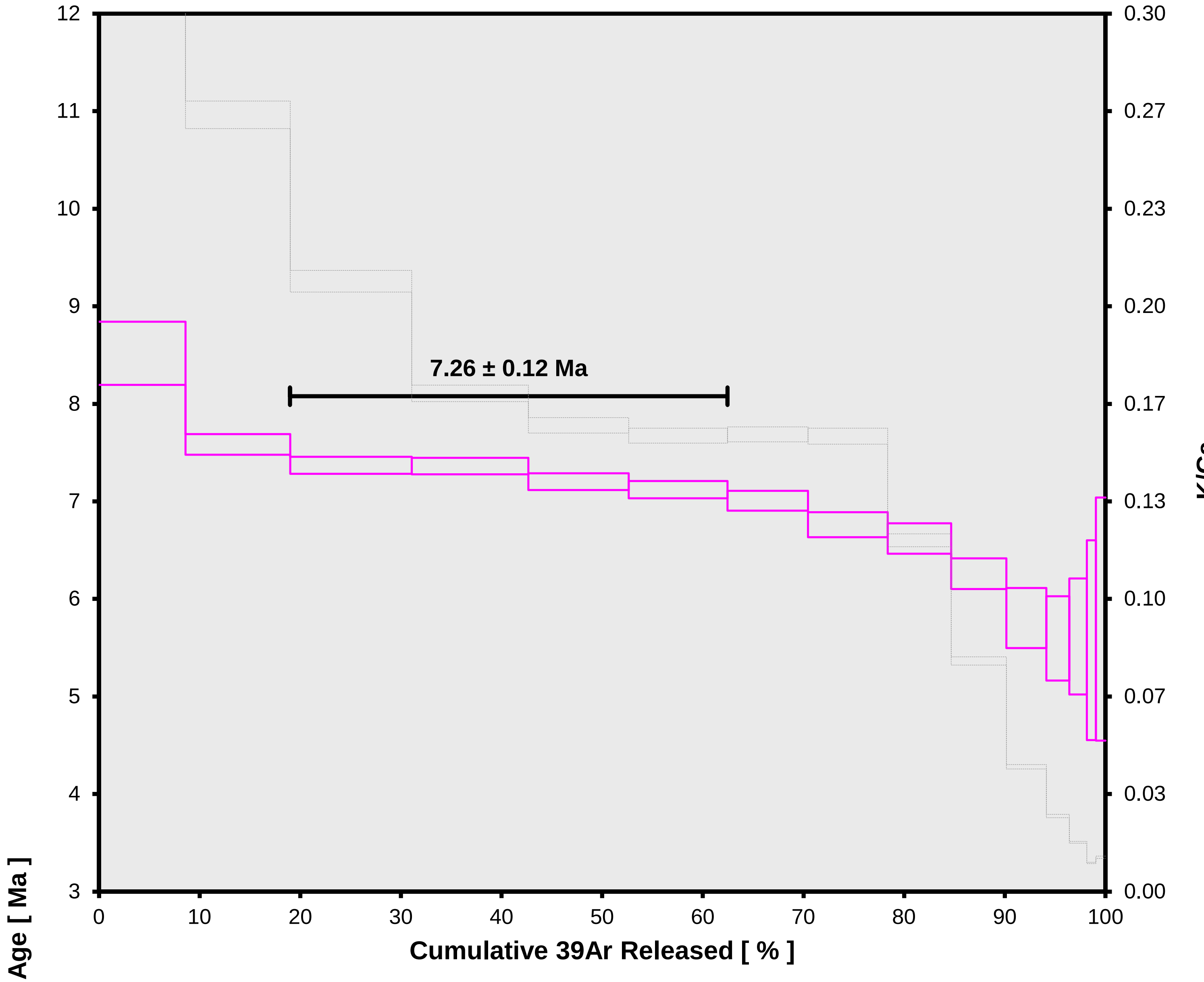

Ar-Ages in Ma

WEIGHTED PLATEAU
7.26 ± 0.12
TOTAL FUSION
7.10 ± 0.05
NORMAL ISOCHRON
6.84 ± 0.89
INVERSE ISOCHRON
6.87 ± 0.88

MSWD (PROBABILITY)
7.94 (0%)

Sample Info

groundmass
MW14 DL2-2
Klaudia Kuiper

IRR = VU107
J = 0.00468970 ± 0.00000469

RECALIBRATED AGE

VU107-J1_1_corr_MW14 DL2-2.full printed at 4/27/2018 (2:40 PM)
ArArCALC v2.5.2

Geochronology laboratory
Vrije Universiteit Amsterdam, Amsterdam, The Netherlands

VU107-J1_1_CORR_MW14 DL2-2.AGE >>> 011_VU107-J-1 >>> VU107 PROJECT

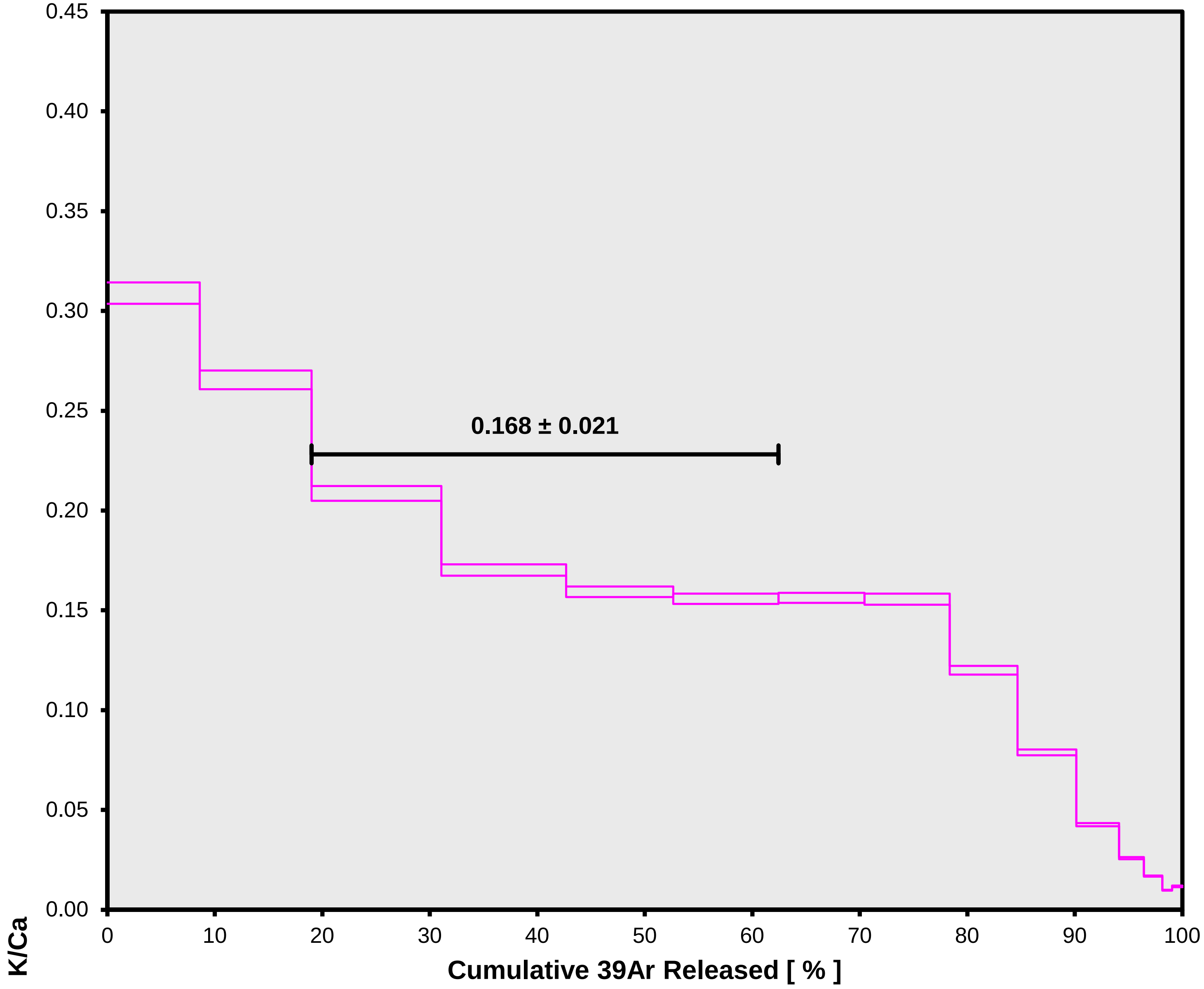

Ar-Ages in Ma

WEIGHTED PLATEAU
7.26 ± 0.12
TOTAL FUSION
7.10 ± 0.05
NORMAL ISOCHRON
6.84 ± 0.89
INVERSE ISOCHRON
6.87 ± 0.88

Sample Info

groundmass
MW14 DL2-2
Klaudia Kuiper

IRR = VU107
J = 0.00468970 ± 0.00000469

RECALIBRATED AGE

VU107-J1_1_corr_MW14 DL2-2.full printed at 4/27/2018 (2:40 PM)
ArArCALC v2.5.2

Geochronology laboratory
Vrije Universiteit Amsterdam, Amsterdam, The Netherlands

VU107-J1_1_CORR_MW14 DL2-2.AGE >>> 011_VU107-J-1 >>> VU107 PROJECT

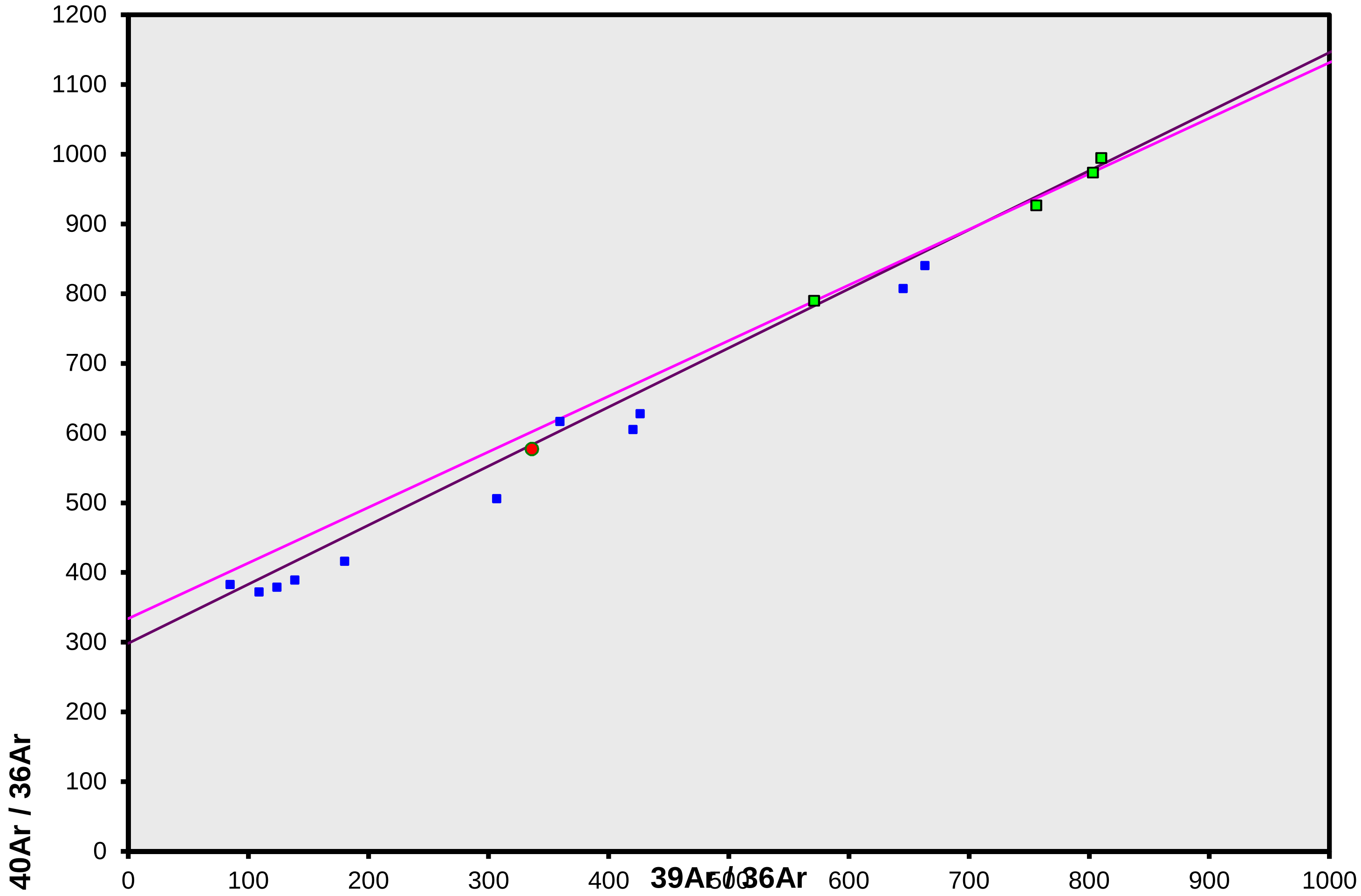

Ar-Ages in Ma

WEIGHTED PLATEAU
7.26 ± 0.12
TOTAL FUSION
7.10 ± 0.05
NORMAL ISOCHRON
6.84 ± 0.89
INVERSE ISOCHRON
6.87 ± 0.88

MSWD (PROBABILITY)
7.99 (0%)

40AR/36AR INTERCEPT
334.0 ± 74.3

Sample Info

groundmass
MW14 DL2-2
Klaudia Kuiper

IRR = VU107
J = 0.00468970 ± 0.00000469

RECALIBRATED AGE

VU107-J1_1_CORR_MW14 DL2-2.AGE >>> 011_VU107-J-1 >>> VU107 PROJECT

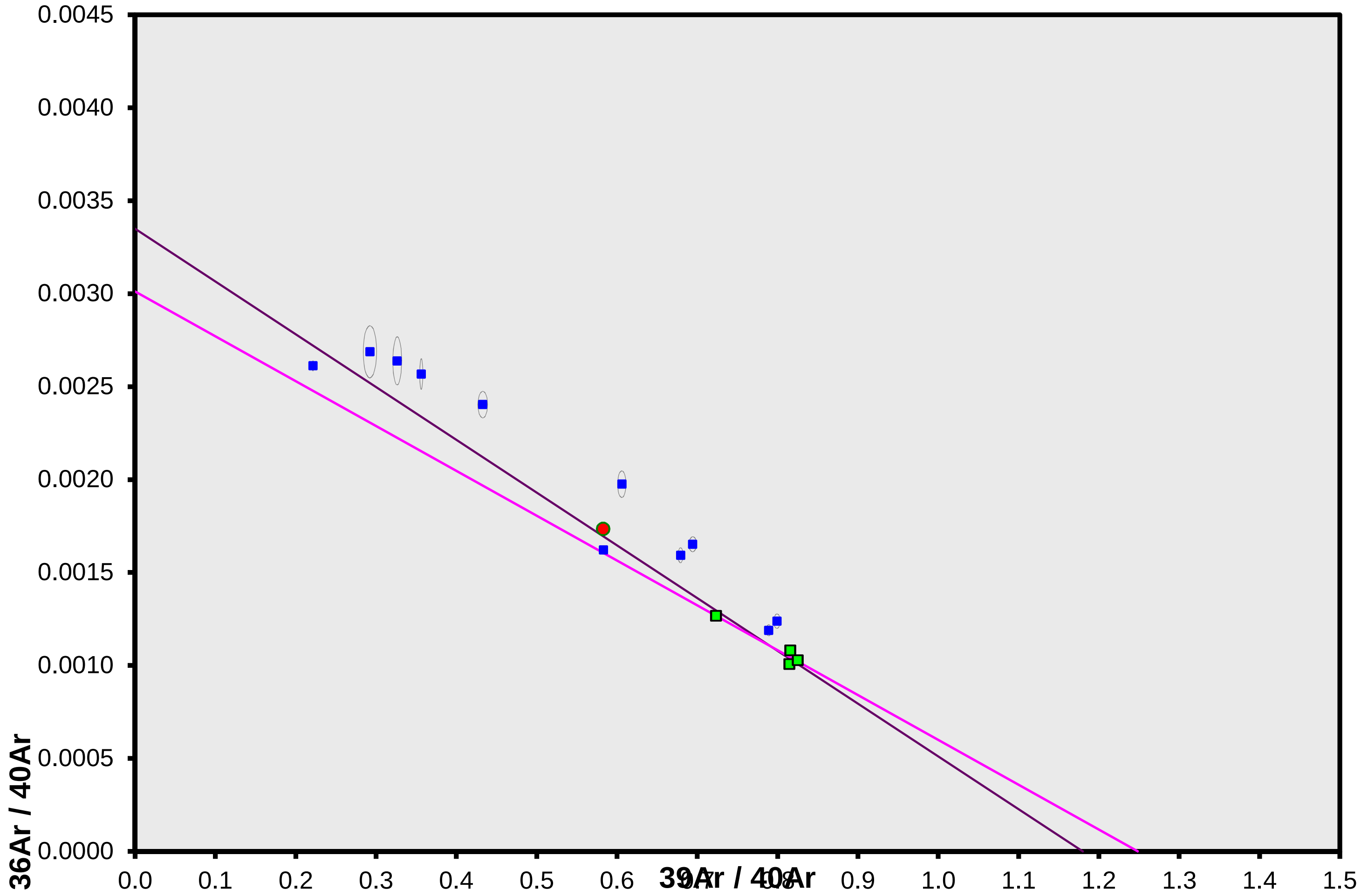

Ar-Ages in Ma

WEIGHTED PLATEAU
7.26 ± 0.12
TOTAL FUSION
7.10 ± 0.05
NORMAL ISOCHRON
6.84 ± 0.89
INVERSE ISOCHRON
6.87 ± 0.88

MSWD (PROBABILITY)
8.13 (0%)

SPREADING FACTOR
8.2%

40AR/36AR INTERCEPT
332.1 ± 75.0

Sample Info

groundmass
MW14 DL2-2
Klaudia Kuiper

IRR = VU107
J = 0.00468970 ± 0.00000469

RECALIBRATED AGE
