# Supplementary material for: Superplume mantle tracked isotopically the length of Africa from the Indian Ocean to the Red Sea
Source: Nat Commun. 2019 Dec 2;10:5493. doi: 10.1038/s41467-019-13181-7 (PMC6889401; doi:10.1038/s41467-019-13181-7)
Supplement: Supplementary file 6 — Supplementary Data 3 [file 41467_2019_13181_MOESM6_ESM.pdf]

Supplementary Data 3. Geochemical analyses of Mozambique Ridge dome samples

Sample	MW14DL2-3	MW14DL2-2	MW14DL3-5	BHVO2A	BHVO2B	BHVO2
Rock Type	Basanite	Basanite	Basanite/ Alkali Basalt			mean
Lat (°S) on/off-bottom	26° 8.12'/26° 7.83'	26° 8.12'/26° 7.83'	26° 7.80'/26° 7.84'			
Long (°E) on/off-bottom	034° 45.23'/034° 45.44'	034° 45.23'/034° 45.44'	034° 45.50'/034° 45.45'			
Depth (m.b.s.l)	925-929	925-929	943-956			
Cruise	SO230	SO230	SO230			
SiO_2	44.00	42.82	45.05			
TiO_2	3.36	3.29	3.40			
Al_2O_3	12.60	12.01	12.62			
Fe_2O_3	14.18	14.03	14.03			
MnO	0.187	0.181	0.194			
MgO	6.91	8.79	6.00			
CaO	11.89	12.25	12.03			
Na_2O	2.88	2.51	2.91			
K_2O	1.08	0.790	1.23			
P_2O_5	1.30	1.48	0.951			
LOI	1.28	1.50	1.25			
Total	99.66	99.66	99.66			
Li	10.890	13.860	6.826	4.691	4.494	4.592
Sc	26.820	26.600	26.970	30.766	30.892	30.829
V	329.100	306.900	331.800	300.077	306.359	303.218
Cr	357.700	361.700	377.300	270.846	277.331	274.089
Co	53.760	56.860	52.890	43.145	43.832	43.488
Ni	124.600	186.700	123.500	122.893	125.159	124.026
Cu	64.550	60.250	75.870	123.925	124.423	124.174
Zn	118.600	118.400	117.400	96.114	98.652	97.383
Ga	32.290	30.680	34.360	21.400	21.426	21.413
Rb	17.170	9.168	25.720	8.982	8.824	8.903
Sr	701.200	694.000	637.800	391.753	389.588	390.670
Y	25.520	25.050	25.100	26.174	26.162	26.168
Zr	209.100	200.600	208.300	162.305	163.850	163.077
Nb	54.140	52.020	52.940	17.818	17.828	17.823
Mo	1.666	1.473	1.710	3.720	3.783	3.721
Sn	2.037	1.987	2.029	1.662	1.663	1.662
Cs	0.131	0.066	0.360	0.112	0.113	0.113
Ba	488.500	456.200	480.200	124.461	125.015	124.738
La	40.320	40.230	40.610	14.481	14.542	14.511
Ce	83.880	82.260	83.240	35.850	35.917	35.883
Pr	9.546	9.313	9.399	5.021	5.036	5.029
Nd	38.660	37.810	38.090	23.202	23.308	23.255
Sm	8.477	8.264	8.341	5.987	6.013	6.000
Eu	2.844	2.775	2.799	1.967	1.975	1.971
Gd	7.713	7.648	7.714	5.973	6.015	5.994
Tb	1.103	1.078	1.088	0.911	0.915	0.913
Dy	6.074	5.959	5.988	5.271	5.304	5.287
Ho	1.058	1.034	1.040	0.950	0.955	0.952
Er	2.696	2.628	2.625	2.451	2.464	2.457
Tm	0.342	0.335	0.335	0.321	0.324	0.323
Yb	2.067	2.025	2.025	1.914	1.922	1.918
Lu	0.274	0.266	0.265	0.272	0.271	0.271
Hf	5.172	5.085	5.141	4.234	4.286	4.260
Ta	2.820	2.765	2.802	1.124	1.133	1.129
W	0.694	0.765	0.817	0.240	0.236	0.237
Tl	0.198	0.142	0.319	0.053	0.047	0.045
Pb	4.426	2.528	2.559	1.669	1.758	1.714
Th	4.895	4.667	4.674	1.163	1.176	1.169
U	2.255	1.818	1.569	0.396	0.404	0.400
$^{87}Sr/^{86}Sr_m$	0.703151	0.703150	0.703155			
±2s	0.000004	0.000004	0.000005			
$^{87}Rb/^{86}Sr$	0.070	0.023	0.078			
$^{87}Sr/^{86}Sr_{in}$	0.703144	0.703148	0.703147			
$^{143}Nd/^{144}Nd_m$	0.512883	0.512885	0.512878			
$\pm 2\sigma$	0.000005	0.000005	0.000005			
$^{147}Sm/^{144}Nd$	0.170	0.165	0.170			
$^{143}Nd/^{144}Nd_{in}$	0.512875	0.512877	0.512870			
$^{206}Pb/^{204}Pb_m$	19.5425	19.5650	19.5531			
$\pm 2\sigma$	0.0003	0.0003	0.0004			
$^{207}Pb/^{204}Pb_m$	15.6317	15.6357	15.6314			
±2s	0.0003	0.0003	0.0003			
$^{208}Pb/^{204}Pb_m$	39.3660	39.3993	39.3766			
$\pm 2\sigma$	0.0008	0.0007	0.0008			
$^{238}U/^{204}Pb$	19.23	19.28	20.87			
$^{235}U/^{204}Pb$	0.14	0.14	0.15			
$^{232}Th/^{204}Pb$	55.11	91.62	53.26			
$^{206}Pb/^{204}Pb_{in}$	19.5216	19.5440	19.5304			
$^{207}Pb/^{204}Pb_{in}$	15.6307	15.6347	15.6304			
$^{208}Pb/^{204}Pb_{in}$	39.3469	39.3676	39.3581			
